# SARS-CoV-2 positivity in offspring and timing of mother-to-child transmission: living systematic review and meta-analysis

**DOI:** 10.1136/bmj-2021-067696

**Published:** 2022-03-16

**Authors:** John Allotey, Shaunak Chatterjee, Tania Kew, Andrea Gaetano, Elena Stallings, Silvia Fernández-García, Magnus Yap, Jameela Sheikh, Heidi Lawson, Dyuti Coomar, Anushka Dixit, Dengyi Zhou, Rishab Balaji, Megan Littmoden, Yasmin King, Luke Debenham, Anna Clavé Llavall, Kehkashan Ansari, Gurimaan Sandhu, Adeolu Banjoko, Kate Walker, Keelin O’Donoghue, Madelon van Wely, Elizabeth van Leeuwen, Elena Kostova, Heinke Kunst, Asma Khalil, Vanessa Brizuela, Nathalie Broutet, Edna Kara, Caron Rahn Kim, Anna Thorson, Olufemi T Oladapo, Javier Zamora, Mercedes Bonet, Lynne Mofenson, Shakila Thangaratinam

**Affiliations:** 1 WHO Collaborating Centre for Global Women’s Health, Institute of Metabolism and Systems Research, University of Birmingham, Birmingham, UK; 2 Birmingham Medical School, University of Birmingham, Birmingham, UK; 3 Clinical Biostatistics Unit, Hospital Universitario Ramón y Cajal (IRYCIS), Madrid, Spain; 4 CIBER Epidemiology and Public Health (CIBERESP), Madrid, Spain; 5 University of Nottingham, Nottingham, UK; 6 University College Cork, Cork, Ireland; 7 Netherlands Satellite of the Cochrane Gynaecology and Fertility Group, Amsterdam University Medical Centre, Amsterdam, Netherlands; 8 Department of Obstetrics and Gynaecology, Amsterdam University Medical Centre, Amsterdam, Netherlands; 9 Blizard Institute, Queen Mary University of London, London, UK; 10 Barts Health NHS Trust, London, UK; 11 St George’s University London, London, UK; 12 UNDP/UNFPA/UNICEF/WHO/World Bank Special Programme of Research, Development and Research Training in Human Reproduction (HRP), Department of Sexual and Reproductive Health and Research, World Health Organization, Geneva, Switzerland; 13 Elizabeth Glaser Paediatric AIDS Foundation, Washington DC, USA; 14 Birmingham Women’s and Children’s NHS Foundation Trust, Birmingham, UK

## Abstract

**Objectives:**

To assess the rates of SARS-CoV-2 positivity in babies born to mothers with SARS-CoV-2 infection, the timing of mother-to-child transmission and perinatal outcomes, and factors associated with SARS-CoV-2 status in offspring.

**Design:**

Living systematic review and meta-analysis.

**Data sources:**

Major databases between 1 December 2019 and 3 August 2021.

**Study selection:**

Cohort studies of pregnant and recently pregnant women (including after abortion or miscarriage) who sought hospital care for any reason and had a diagnosis of SARS-CoV-2 infection, and also provided data on offspring SARS-CoV-2 status and risk factors for positivity. Case series and case reports were also included to assess the timing and likelihood of mother-to-child transmission in SARS-CoV-2 positive babies.

**Data extraction:**

Two reviewers independently extracted data and assessed study quality. A random effects model was used to synthesise data for rates, with associations reported using odds ratios and 95% confidence intervals. Narrative syntheses were performed when meta-analysis was inappropriate. The World Health Organization classification was used to categorise the timing of mother-to-child transmission (in utero, intrapartum, early postnatal).

**Results:**

472 studies (206 cohort studies, 266 case series and case reports; 28 952 mothers, 18 237 babies) were included. Overall, 1.8% (95% confidence interval 1.2% to 2.5%; 140 studies) of the 14 271 babies born to mothers with SARS-CoV-2 infection tested positive for the virus with reverse transcriptase polymerase chain reaction (RT-PCR). Of the 592 SARS-CoV-2 positive babies with data on the timing of exposure and type and timing of tests, 14 had confirmed mother-to-child transmission: seven in utero (448 assessed), two intrapartum (18 assessed), and five during the early postnatal period (70 assessed). Of the 800 SARS-CoV-2 positive babies with outcome data, 20 were stillbirths, 23 were neonatal deaths, and eight were early pregnancy losses; 749 babies were alive at the end of follow-up. Severe maternal covid-19 (odds ratio 2.4, 95% confidence interval 1.3 to 4.4), maternal death (14.1, 4.1 to 48.0), maternal admission to an intensive care unit (3.5, 1.7 to 6.9), and maternal postnatal infection (5.0, 1.2 to 20.1) were associated with SARS-CoV-2 positivity in offspring. Positivity rates using RT-PCR varied between regions, ranging from 0.1% (95% confidence interval 0.0% to 0.3%) in studies from North America to 5.7% (3.2% to 8.7%) in studies from Latin America and the Caribbean.

**Conclusion:**

SARS-CoV-2 positivity rates were found to be low in babies born to mothers with SARS-CoV-2 infection. Evidence suggests confirmed vertical transmission of SARS-CoV-2, although this is likely to be rare. Severity of maternal covid-19 appears to be associated with SARS-CoV-2 positivity in offspring.

**Systematic review registration:**

PROSPERO CRD42020178076.

**Readers’ note:**

This article is a living systematic review that will be updated to reflect emerging evidence. Updates may occur for up to two years from the date of original publication.

## Introduction

Maternal infection with SARS-CoV-2 has raised concerns about the potential for mother-to-child transmission.^1^ Although there is robust evidence on the magnitude and modes of SARS-CoV-2 transmission in the general population and the prevalence of test positivity,^2^ little is known about the burden of SARS-CoV-2 positivity in babies born to infected women. Existing studies vary widely in the reported rates of SARS-CoV-2 test positivity and definition and timing of transmission from exposure to the virus in utero or during the intrapartum and postnatal periods.^3^ ^4^ ^5^ ^6^

To confirm infection and accurately determine when transmission of SARS-CoV-2 occurs, appropriately timed and repeated tests are needed in relevant samples.^7^ ^8^ Detection of SARS-COV-2 in specimens from fetuses (eg, placental, amniotic fluid) or offspring (eg, nasopharyngeal or faecal) using reverse transcriptase polymerase chain reaction (RT-PCR) alone is not sufficient to diagnose fetal infection.^9^ ^10^ ^11^ The accuracy of anti-SARS-CoV-2 IgM assays for serological diagnosis of congenital infection also varies.^12^ ^13^ Furthermore, as timing and route of infection may affect clinical outcomes, we need to be able to differentiate between intrapartum transmission of the virus and infection acquired soon after birth through contact with mother, caregivers, healthcare workers, or the neonate’s environment.^11^

The clinical outcomes in SARS-CoV-2 positive babies and those with confirmed vertical infection also need to be ascertained. It is unknown whether maternal factors such as severe covid-19, timing of infection, mode of delivery, breastfeeding, and postnatal contact with offspring are associated with SARS-CoV-2 positivity in babies. We undertook a systematic review to assess the rates of SARS-CoV-2 positivity in babies born to mothers with SARS-CoV-2 infection, the timing of mother-to-child transmission, perinatal outcomes in positive babies, and factors associated with SARS-CoV-2 positivity in offspring.

## Methods

Our systematic review is based on a prospective living protocol (PROSPERO CRD42020178076; registered 22 April 2020). In this paper, we focus on mother-to-child transmission using the preferred reporting items for systematic reviews and meta-analyses (PRISMA) recommendations (see supplementary appendix 1).

### Search strategy

We searched major databases, preprint servers, and websites that serve as repositories for covid-19 studies, including Medline, Embase, Cochrane database, WHO COVID-19 database, Living Overview of the Evidence platform, China National Knowledge Infrastructure (CNKI), and Wanfang databases (1 December 2019 to 3 August 2021) for studies (cohort, case series, and case report) on SARS-CoV-2 infection in pregnant and recently pregnant women (including after abortion or miscarriage). No language restrictions were applied. Our searches were coordinated with the EPPI-Centre, the WHO (World Health Organization) Library, and the Cochrane Gynaecology and Fertility group (see supplementary appendix 2).

### Study selection

Sixteen reviewers contributed to study selection. Two independent reviewers assessed each study using a two stage process. In the first stage, the titles and abstracts of all citations were screened and the full texts examined for inclusion in the second stage. Disagreements between reviewers were resolved through discussion with a third reviewer (ST, JA, or ES). To assess SARS-CoV-2 positivity rates in offspring, we included cohort studies of pregnant and recently pregnant women who sought hospital care for any reason and had a diagnosis of SARS-CoV-2 infection, and where SARS-CoV-2 status was ascertained in the fetus or neonate using RT-PCR (neonatal pharyngeal, rectal, or faecal swabs, neonatal or cord blood, fetal tissue, placental samples, or amniotic fluid) or serological tests (anti-SARS-CoV-2 IgM), or both. We defined cohort studies as those that sampled consecutive women, who were followed-up to ascertain the SARS-CoV-2 status of their offspring within the first 30 days after birth.^14^ Unless specified otherwise, we use the term babies and offspring to denote both fetuses and neonates.

In addition to the cohort studies, we included case series and case reports to assess the timing of mother-to-child transmission and likelihood of infection. We also included cohort studies of pregnant and recently pregnant women with a diagnosis of SARS-CoV-2 infection that reported on maternal and perinatal risk factors such as maternal severe covid-19, admission to an intensive care unit, and death; timing of exposure to the virus (antenatal versus postnatal, third versus first or second trimester); intrapartum factors (<37 weeks preterm versus term), mode of delivery (immediate versus delayed cord clamping); postnatal care (skin-to-skin contact versus none; not separated versus separated at birth, breastfed versus not breastfed), and SARS-CoV-2 status of offspring.

### Quality assessment and data extraction

We assessed the internal and external validity of non-comparative cohorts using the tool by Hoy et al.^15^ For internal validity, we considered studies to be at low risk of bias if data were collected from clinical records or research case report forms (data collection), clearly defined outcomes (case definition), confirmed SARS-CoV-2 infection using laboratory based tests (instrument validity), used same mode of data collection in all participants (ascertainment bias), and had sufficient follow-up, with appropriate numerator and denominator. For external validity, we considered studies to be at low risk of bias if they were representative of the national population for relevant variables (population), representative of the target population (sampling frame), undertook a census (selection bias), and the response rate of individuals with and without the outcome was more than 75% (non-response bias). We assessed the methodological quality of the comparative cohort studies using the Newcastle Ottawa scale for selection, comparability, and outcome ascertainment bias outcome.^16^

Using a pre-piloted form, six independent reviewers in two sets extracted data on study design, number of pregnant women with SARS-CoV-2 infection, type of SARS-CoV-2 test in mothers and babies (RT-PCR, IgM), maternal characteristics (stage of pregnancy at diagnosis, severity of covid-19 as defined by authors), mode of delivery, type of samples tested (neonatal nasopharyngeal, rectal, or faecal swabs, neonatal or cord blood, fetal tissue, placenta, amniotic fluid, vaginal fluid, breast milk), and timing of sample collection. We also extracted data on the clinical outcomes of all SARS-CoV-2 positive fetuses and neonates when available, including early pregnancy outcomes of miscarriage and abortion. A detailed deduplication process was used to cross check data against other studies published by the same authors or those that included women from the same institutions. We contacted study authors for unpublished information and to query duplication of data.

### Data analysis

We summarised the SARS-CoV-2 positivity rates in offspring identified by RT-PCR or anti-SARS-CoV-2 IgM assays, or both, as a proportion of all babies born to mothers with SARS-CoV-2 infection in cohort studies. After transforming data using Freeman-Tukey double arcsine transformation, we used DerSimonian and Laird random effects meta-analysis to calculate rates and corresponding 95% confidence intervals. Heterogeneity was reported as I^2^ and τ^2^ estimates. Sensitivity analysis for SARS-CoV-2 positivity rates in babies was done by restricting the analysis to studies at low risk of bias, babies tested at less than 24 hours after birth, and babies born to women with SARS-CoV-2 infection diagnosed antenatally. The rates of SARS-CoV-2 positivity were also evaluated by subgroups of studies involving babies and mothers from various World Bank regions.

We ascertained the timing of mother-to-child transmission based on the World Health Organization classification in all studies (cohort, case series, case reports) that reported SARS-CoV-2 positive babies and provided information on the timing of exposure (antenatal, postnatal) and test timings in the babies (see supplementary appendix 3).^17^ Each baby with a positive test result was placed in mutually exclusive categories for likelihood of infection: confirmed (definite infection), possible (evidence suggestive of infection but not confirmatory), unlikely (infection cannot be ruled out), and indeterminate (tests required to define classification have not been performed) for in utero, intrapartum, or early postnatal transmission. In addition to the specifications in the WHO criteria, we categorised babies to have confirmed or possible in utero infection if they had a positive test result in the first 24 hours after birth and did not have a test between 24 and 48 hours but had a repeat positive test result from a sterile (confirmed) or non-sterile (possible) sample after 48 hours and before seven days, with no negative test results before the repeat positive test result. We also added one further “indeterminate” category for intrapartum transmission: when babies had a negative test result or no test in the first 24 hours after birth and a single anti-SARS-CoV-2 IgM positive result at 7-14 days with no confirmatory test; and a further “indeterminate” category for postpartum transmission: when babies had a negative test result in the first 48 hours after birth with a single positive non-sterile sample after 48 hours or IgM result at more than 14 days with no or negative confirmatory test result.

To summarise the associations between maternal and perinatal characteristics and SARS-CoV-2 status in exposed babies, we pooled comparative dichotomous data as odds ratios and 95% confidence intervals using random effects meta-analysis. When meta-analysis was considered inappropriate because of excessive clinical or statistical heterogeneity or when SARS-CoV-2 positive offspring were selectively reported in the cohort studies, we used a narrative descriptive approach to summarise the evidence, such as for clinical outcomes in test positive babies and test positivity in various biological samples. All statistical analyses were performed using Stata (version 16).

### Patient and public involvement

This study is supported by Katie’s Team, a dedicated patient and public involvement group in women’s health. The team was involved in the interpretation and reporting of this living systematic review through participation in virtual meetings. Findings will be made available on our website in a format more suitable for patients and members of the public (www.birmingham.ac.uk/research/who-collaborating-centre/pregcov/index.aspx).

## Results

**Fig 1:**
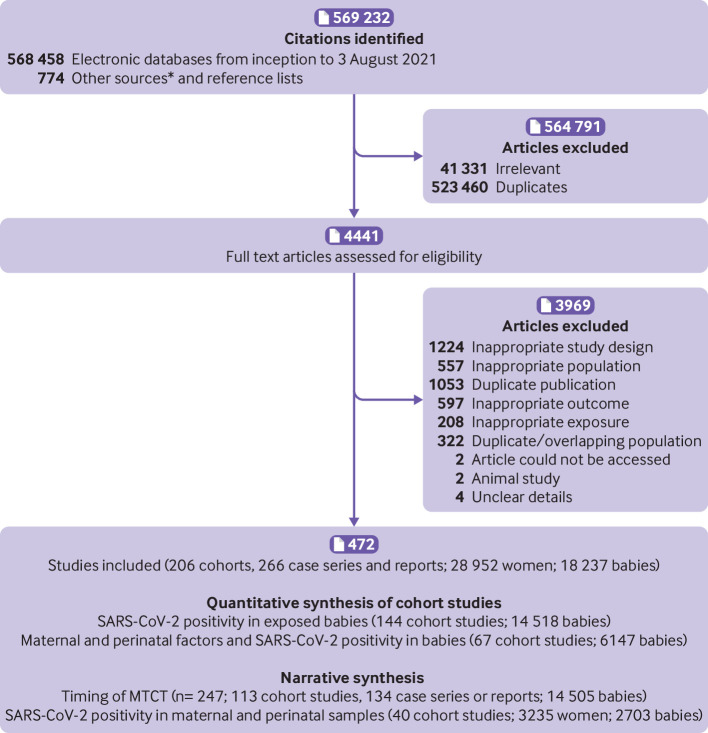
Study selection process in systematic review: SARS-CoV-2 positivity in babies born to mothers with covid-19 and timing of mother-to-child transmission. *See supplementary figure for details of other sources

Overall, we included 472 studies (206 cohort studies, 266 case series and case reports; 28 952 mothers, 18 237 babies) from 569 232 identified articles (fig 1). None of the studies were conducted during the emergence of any SARS-CoV-2 variants of concern. Overall, 144 cohort studies reported on SARS-CoV-2 positivity status in 14 518 exposed babies. A total of 988 babies tested positive for SARS-CoV-2 across all study designs (247 studies; 113 cohorts, 134 case series or case reports). Sixty seven comparative cohorts (with 6147 mother-baby dyads) reported on various maternal and perinatal factors and SARS-CoV-2 positivity in offspring. In 144 cohort studies, SARS-CoV-2 testing of various maternal and perinatal biological samples (placenta, amniotic fluid, maternal vaginal fluid, stool samples, and breast milk) were reported in a proportion of participants (3235 mothers, 2703 babies).

### Characteristics of included studies

Most of the included studies were from the World Bank regions of Europe and Central Asia (145/472, 31%) and North America (87/472, 18%), followed by East Asia and Pacific (73/472, 15%), Middle East and North Africa (60/472, 13%), Latin America and the Caribbean (51/472, 11%), and South Asia (51/472, 11%), and five studies were from Sub-Saharan Africa (5/472, 1%). Maternal infection was confirmed by laboratory tests in 99% (467/472) of the studies. The most common test to ascertain infection in offspring was RT-PCR in 97% of cohort studies (140/144); 10% (15/144) of cohort studies used either anti-SARS-CoV-2 IgM alone or with RT-PCR (see supplementary appendix 4).

### Quality of included cohort studies

Our internal validity assessment of the non-comparative cohorts showed a low risk of bias for data collection in 98% (194/197) of the studies, 62% (122/197) for case definition, 100% (197/197) for measurement, 97% (192/197) for differential verification, 60% (119/197) for adequate follow-up, and 92% (182/197) for appropriate numerator and denominator. For external validity, the studies had low risk of bias for representativeness in 9% (17/197) of the studies, 24% (47/197) for sampling, 88% (173/197) for selection, and 98% (194/197) for non-response. The overall risk of bias (Newcastle Ottawa scale) for the included comparative cohort studies was low in 99% (66/67) of studies; 97% (65/67) had low risk of bias for study selection, 28% (19/67) for comparability of cohorts, and 78% (52/67) for outcome assessment (see supplementary appendix 5).

### SARS-CoV-2 positivity in exposed babies in cohort studies

**Fig 2:**
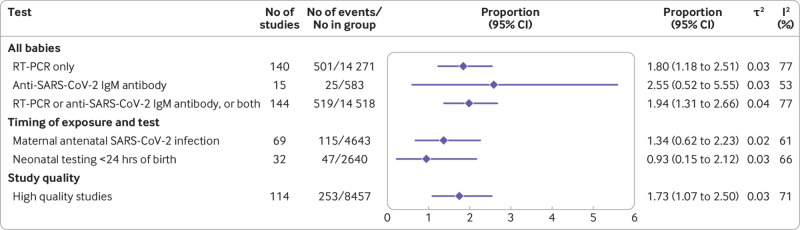
Rates of SARS-CoV-2 positivity in babies (including fetuses) born to mothers seeking hospital care for any reason and having active or recently diagnosed SARS-CoV-2 infection. RT-PCR=reverse transcriptase polymerase chain reaction

SARS-CoV-2 positivity using RT-PCR was observed in 1.8% (95% confidence interval 1.2% to 2.5%) of all babies (n=14 271) born to mothers with a diagnosis of SARS-CoV-2 infection (140 cohort studies); 1.9% (1.3% to 2.7%) tested positive when studies used either RT-PCR or anti-SARS-CoV-2 IgM tests (144 studies, 14 518 babies). Anti-SARS-CoV-2 specific IgM antibodies were shown in 2.6% (95% confidence interval 0.5% to 5.6%) of exposed babies who were tested (15 studies, 583 babies) (fig 2). In sensitivity analysis, the SARS-CoV-2 RT-PCR positivity rate limited to high quality studies was 1.7% (95% confidence interval 1.1% to 2.5%) in babies born to mothers with SARS-CoV-2 infection, a finding similar to that of the main analysis. When the analysis was limited to babies of mothers with a diagnosis of SARS-CoV-2 infection in the antenatal period, the positivity rate was 1.3% (0.6% to 2.2%); 0.9% (0.2% to 2.1%) when limited to babies tested in the first 24 hours after birth (fig 2). In the subgroup analyses, SARS-CoV-2 positivity rates by RT-PCR in offspring varied between regions, ranging from 0.1% (0.0% to 0.3%) in studies from North America to 5.7% (3.2% to 8.7%) in studies from Latin America and the Caribbean (see supplementary appendix 6).

### Timing of mother-to-child transmission

**Fig 3:**
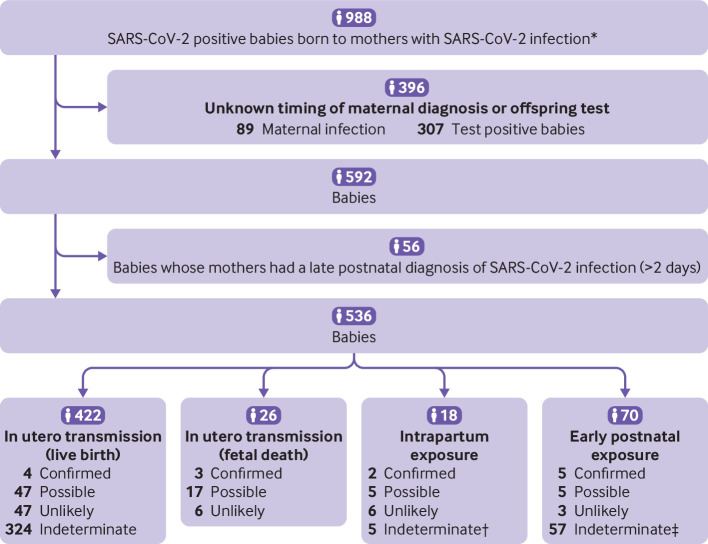
Flowchart showing inclusion of babies classified by timing of SARS-CoV-2 mother-to-child transmission using the World Health Organization classification system. *Clinical and laboratory diagnosis. †Category added to existing WHO classification. ‡Babies with positive serology at days 7-14, but no confirmatory test done. §Includes 29 babies with negative test ≤48 hours, then positive test >48 hours with no further or negative confirmatory test

Of the 14 505 babies born to mothers with SARS-CoV-2 infection across all studies (cohorts, case series, case reports), data were sufficient to apply the WHO classification system for timing of exposure and likelihood of infection in 592 babies, including 448 babies exposed in utero, 18 exposed intrapartum, and 70 exposed in the early postnatal period (fig 3). After exclusion of 56 babies where maternal SARS-CoV-2 infection was diagnosed late (>2 days postnatally), 14 of the 536 babies (including fetuses) were categorised as having confirmed infection (4/422 live births with in utero transmission, 3/26 fetal deaths or miscarriages with in utero transmission, 2/18 intrapartum, and 5/70 early postnatal exposure), and 74 as possible infection (47/422 live births with in utero transmission, 17/26 fetal deaths or miscarriages with in utero transmission, 5/18 intrapartum, and 5/70 early postnatal exposure) (fig 3). The likelihood of infection was classified as indeterminate for 386 babies, mainly owing to the lack of repeat confirmatory testing within the prespecified time points. Table 1 and supplementary appendix 7 provide the maternal and perinatal characteristics and SARS-CoV-2 test results of the babies with confirmed and possible vertical infection, respectively.

**Table 1:** Maternal and perinatal characteristics of babies (including fetuses) with confirmed in utero, intrapartum, and early postnatal transmission of SARS-CoV-2

	Maternal characteristics	Mode of delivery	Measures to prevent SARS-CoV-2 MTCT	Tests for SARS-CoV-2 MTCT			Fetal and neonatal characteristics
				Initial test	Further tests		
**Confirmed in utero MTCT**							
Live births:							
Behling 2020	Age 36 years; asymptomatic for covid-19; SARS-CoV-2 IgG positive on postnatal day 10	Operative vaginal delivery	None reported	Placenta RT-PCR positive	At autopsy, SARS-CoV-2 found in neonatal organs by nested RT-PCR		Gestational age 39 weeks; body weight 2600 g; admitted to neonatal intensive care unit owing to symptoms; died on day 4 after birth
Correia 2020	Age 40 years; pre-eclampsia and previous risk of preterm delivery; symptoms of covid-19; RT-PCR positive on nasopharyngeal swab at 34 weeks before delivery; stool RT-PCR positive	Caesarean section	Delivery in negative pressure room; no skin-to-skin contact	Blood sample and nasopharyngeal swab RT-PCR positive at 30 minutes	Deep tracheal aspirate RT-PCR positive at 48 hours, and days 9, 15, and 19; blood IgM and IgG initially negative on days 3, 7, and 11, and then positive on day 15; stool PCR positive on day 7		Gestational age 34 weeks; body weight 1510 g; Apgar score 1 and 5 minutes: 8 and 9; required positive airway pressure ventilation, admitted to neonatal intensive care unit; alive
Lima 2020*	Age 27 years, gravida 2 (para not reported); no comorbidities; flu-like symptoms at 29 weeks; rapid serological test IgM positive and IgG positive at 32 weeks	Caesarean section	Mother wore N95 mask during delivery in isolated operative room; immediate mother-baby separation; breastfed from day 7	Blood sample and nasopharyngeal swab RT-PCR positive at one hour; cord blood IgM negative but IgG positive; peripheral blood at birth IgM negative but IgG positive; placenta and amniotic fluid RT-PCR negative; chorion RT-PCR inconclusive	Blood sample and nasopharyngeal swab RT-PCR positive on day 5; nasopharyngeal swab negative on days 13 and 14		Gestational age 33 weeks; body weight 2400 g; Apgar score 1 and 5 minutes: 7 and 9; fetal echocardiogram at 32 weeks showed high risk of cardiac tamponade, leading to emergency caesarean section; prophylactic steroids given for fetal lung maturation; bag mask ventilation at birth, then transferred to neonatal intensive care unit; computed tomography scan showed some lung changes; became unstable on day 3 and was intubated; pericardial drain inserted; extubated on day 7; alive on discharge
NG DCE 2021	Age 39 years; primigravida; fever and cough; signs of pneumonia on chest radiograph; nasopharyngeal swab RT-PCR positive at 29 gestational weeks	Preterm labour, spontaneous vaginal delivery	Mother wore surgical mask during delivery; mother-baby separation at birth	Nasopharyngeal swab RT-PCR positive at 2 hours; blood sample IgM and IgG negative at birth	Tracheal aspirate RT-PCR positive at 26 hours; blood sample IgM and IgG positive on day 14		Gestational age 29 weeks; body weight 1100 g; Apgar score 1 and 5 minutes: 9 and 9; symptoms present; respiratory distress, required non-invasive continuous positive airway pressure ventilation; bilateral ground glass opacities on computed tomography scan; alive
Fetal death:							
Rodrigues 2020	Age 19 years; no medical history; nasopharyngeal swab RT-PCR positive just before delivery; asymptomatic	Vaginal delivery (stillbirth)	None	Fetal tissues RT-PCR positive at autopsy			No fetal heartbeat at 34 weeks; small for gestational age (third centile); body weight 1460 g
Valdespino-Vazquez 2020 (twins)	Age 28 years; gravida 4 para 3; fever, headache, arthralgia, fatigue at 13 weeks, and also dark vaginal bleeding; nasopharyngeal swab initially RT-PCR negative but became positive	Vaginal delivery (miscarriage)	None reported	Fetal organs RT-PCR and immunofluorescence positive in both fetuses; fetus 1 electron microscopy positive in lung	Placenta RT-PCR positive; electron microscopy positive; immunofluorescence positive in both placentas		Gestational age 13 weeks; diamniotic twin pregnancy, no heartbeat in both at 13 weeks. Twin 1: length 12, body weight 37 g. Twin 2: severely macerated
**Confirmed intrapartum MTCT**							
Zeng 2020 (baby 1; baby 2)	Nasopharyngeal swab RT-PCR positive just before delivery; fever and pneumonia (computed tomography scan); not admitted to intensive care unit; no information on maternal characteristics	Caesarean section	Mother-baby separation at birth; not breastfed	Amniotic fluid and cord blood RT-PCR negative	Nasopharyngeal swab RT-PCR positive on days 2 and 4		Gestational age 40 weeks. Baby 1: body weight 3250 g; lethargy, fever; pneumonia on chest radiograph; admitted to neonatal intensive care unit; alive. Baby 2: body weight 3360 g; lethargy, vomiting, fever; pneumonia on chest radiograph; alive
**Confirmed early postpartum MTCT**							
Bastug 2020	Age 20 years; gravida 2 para 2, covid-19 diagnosed at 39 weeks, asymptomatic; nasopharyngeal swab RT-PCR positive just before delivery; breast milk RT-PCR positive	Vaginal delivery	Mother wore mask during delivery and when expressing breast milk; neonate separated from mother after birth and given expressed breast milk	Nasopharyngeal swab RT-PCR negative on day 1		Peripheral blood RT-PCR positive on day 4	Gestational age 39 weeks; body weight 2980 g; admitted to neonatal intensive care unit; asymptomatic; alive
Demirjian 2020	Age 34 years; gravida 3 para 2, covid-19 diagnosed at 38 weeks; severe symptoms of increasing dyspnoea requiring intubation in intensive care unit; sputum RT-PCR positive just before delivery; maternal blood RT-PCR positive	Caesarean section	Mother-baby separation at birth; formula fed exclusively	Nasopharyngeal swab and rectal, peripheral blood, and cerebrospinal fluid samples RT-PCR negative on day 1		Nasopharyngeal swab RT-PCR positive on days 4 and 8; rectal and peripheral blood samples RT-PCR positive on day 7 (rectal sample RT-PCR negative on day 4 and peripheral blood sample RT-PCR negative on day 5	Gestational age 39 weeks; body weight 4170 g; Apgar score 1, 5, and 10 minutes: 5, 9, and 9; fever, coryza, and mild tachypnoea; alive
Gordon 2020	Age 36 years; gravida 3 para 0; covid-19 diagnosed at 32 weeks; cough, high fever, and lymphopenia; nasopharyngeal swab RT-PCR positive just before delivery	Caesarean section	Mother wore mask during delivery; neonate separated from mother after birth	Nasopharyngeal swab RT-PCR negative on day 1		Nasopharyngeal swab RT-PCR positive on days 4 and 14 (with further positive test results on days 21 and 29)	Gestational age 32 weeks; body weight 2150 g; alive; radiography findings consistent with surfactant deficiency lung disease
Komiazyk 2020	Age 28 years; asymptomatic; nasopharyngeal swab RT-PCR positive just before delivery (results known after delivery)	Vaginal delivery	Skin-to-skin contact; mother-baby separation later when maternal RT-PCR result was known	Nasopharyngeal swab RT-PCR negative on day 1		Nasopharyngeal swab RT-PCR positive on days 5 and 10	Gestational age 40 weeks; Apgar score 10; asymptomatic; alive
Yu 2020	Gravida 1 para 0, symptomatic, covid-19 diagnosed at 38 weeks; nasopharyngeal swab RT-PCR positive on postnatal day 1	Caesarean section	Neonate in room with mother; not breastfed	Cord blood sample RT-PCR negative		Nasopharyngeal swab RT-PCR positive on days 7 and 15	Gestational age 38 weeks; body weight 3600 g; Apgar score “normal”; fever; chest radiograph showed diffuse consolidation; alive

MTCT=mother-to-child-transmission (according to World Health Organization classification); RT-PCR=reverse transcriptase polymerase chain reaction.

* Classified as “confirmed” in utero as tests were repeated <24 hours, despite not meeting WHO criteria of positive test result at 24-48 hours.

### Outcomes of SARS-CoV-2 positive babies

Outcomes were reported for 800 SARS-CoV-2 positive babies (247 studies including cohort, case series, case reports); 749 babies were alive at the end of follow-up, and eight early pregnancy losses, 20 stillbirths, and 23 neonatal deaths occurred (table 2). Of the seven babies with confirmed in utero infection, three were alive at end of follow-up, one died after delivery, and one was stillborn, and early pregnancy loss occurred in a set of twin fetuses. Both babies with confirmed intrapartum infection and the five with early postnatal infection were alive at the end of follow-up (table 1). Fifty eight babies with symptoms (58/146) were born preterm, and gestational age was not known in another 18 babies. Of the 121 SARS-CoV-2 positive babies in whom radiological findings were reported (as defined by the authors), abnormalities were seen in 71, including 28 preterm babies (see supplementary appendix 8).

**Table 2:** Outcomes in SARS-CoV-2 positive babies born to mothers with covid-19 in all studies (cohort, case series, and case reports). Values are numbers (percentages) unless stated otherwise

Outcome in offspring	Test positive term babies (≥37 weeks)					Test positive preterm babies and early pregnancy (<37 weeks)						Test positive babies (gestation not known)				All SARS-CoV-2 positive babies			
	Mild maternal disease (n=152)	Severe maternal disease* (n=10)	Severity not known (n=55)	Total (n=217)		Mild maternal disease (n=95)		Severe maternal disease* (n=26)	Severity not known (n=22)	Total (n=143)		Mild maternal disease (n=95)	Severity not known (n=533)	Total (n=628)		Mild maternal disease (n=342)	Severe maternal disease* (n=36)	Severity not known (n=610)	Total (n=988)
Alive at end of follow-up	145 (71)	10 (5)	50 (24)	205		61 (62)	21 (21)		17 (17)	99		86 (19)	359 (81)	445		292 (40)	31 (41)	426 (57)	749
Miscarriage or abortion						8 (100)	-		-	8						8 (100)	-	-	8
Stillbirth	-	-	-	-		16 (84)	2 (11)		1 (5)	19		-	1 (100)	1		16 (80)	2 (10)	2 (10)	20
Neonatal death	4 (100)	-	-	4		8 (62)	2 (15)		3 (23)	13		1 (17)	5 (83)	6		13 (57)	2 (9)	8 (35)	23
Not known	3 (38)	-	5 (63)	8		2 (50)	1 (25)		1 (25)	4		8 (5)	168 (95)	176		13 (7)	1 (0.5)	174 (93)	188

* Either one of severe symptoms of covid-19, admission to intensive care unit, or maternal death.

### Maternal and perinatal factors associated with SARS-CoV-2 positivity in offspring

We found a significant association between maternal factors such as severe covid-19 (odds ratio 2.36, 95% confidence interval 1.28 to 4.36, I^2^=10%; 22 studies, 2842 women), maternal admission to the intensive care unit (3.46, 1.74 to 6.91, I^2^=0%; 19 studies, 2851 women), maternal death (14.09, 4.14 to 47.97, I^2^=0%; 7 studies, 725 women), and postnatal diagnosis of SARS-CoV-2 infection in the mother (4.99, 1.24 to 20.13, I^2^=65%; 12 studies, 750 women) and SARS-CoV-2 positive status in the babies (table 3). No associations were shown between SARS-CoV-2 positivity in babies and the trimester of maternal infection (third versus first or second trimester), preterm birth, mode of delivery, breastfeeding, or mother-baby separation at birth (table 3).

**Table 3:** Maternal and perinatal factors associated with SARS-CoV-2 positivity in offspring

Risk factors	No of studies	No of mother-baby dyads	No of test positive babies*/No with risk factors	No of test positive babies*/No without risk factors	Odds ratio (95% CI)	I^2^ (%)
**Maternal factors**						
Severe covid-19	22	2842	18/331	125/2511	2.36 (1.28 to 4.36)	10
Maternal death	7	725	6/15	28/710	14.09 (4.14 to 47.97)	0
Admission to ICU	19	2851	7/92	123/2759	3.46 (1.74 to 6.91)	0
**Timing of maternal infection**						
Postnatal *v* antenatal	12	750	19/122	54/628	4.99 (1.24 to 20.13)	65
3rd *v* 1st or 2nd trimester	13	1422	104/1403	2/19	0.29 (0.08 to 1.10)	0
**Intrapartum factors**						
Preterm *v* term	40	4126	55/618	203/3508	1.47 (0.99 to 2.17)	2
Mode of delivery	49	4814	159/2429	99/2385	1.38 (0.97 to 1.95)	18
**Postnatal care**						
Not separated at birth *v* separated	11	1617	42/658	48/959	1.37 (0.47 to 3.98)	64
Breastfed *v* not breastfed	13	1545	43/783	39/762	0.74 (0.34 to 1.62)	29

ICU=intensive care unit; CI=confidence interval.

* Reverse transcriptase polymerase chain reaction.

### SARS-CoV-2 positivity in maternal and perinatal biological samples

In addition to testing infants for SARS-CoV-2, evidence was found for SARS-CoV-2 positivity in additional maternal and perinatal biological samples tested in cohort studies: from placental tissue in 59 women (355 tested, 13 studies), placental RT-PCR swabs in four woman (225 tested, 13 studies), amniotic fluid in 11 women (476 tested, 18 studies), vaginal fluid in two women (315 tested, 9 studies), and breast milk in eight women (328 tested, 14 studies) (see supplementary appendix 9). Data were inadequate to assess the SARS-CoV-2 positivity status in newborn babies of women with positive placental, amniotic fluid, or other biological samples. When studies of all designs were included, RT-PCR positivity was found in 89 placental samples (n=538 tested), 14 amniotic fluid samples (n=390), five maternal vaginal fluid samples (n=182), 20 babies’ stool specimens (n=95), and seven breast milk samples (n=422).

## Discussion

This living systematic review and meta-analysis found that less than 2% of babies born to mothers seeking hospital care for any reason and with a diagnosis of SARS-CoV-2 infection also test positive for SARS-CoV-2; the rates are lower (1%) when limited to babies with antenatal or intrapartum exposure to the virus. We found evidence for confirmed mother-to-child-transmission through in utero, intrapartum, and early postnatal exposure; but the overall risk is likely to be low. Severity of maternal covid-19 and postnatal maternal infection seem to be associated with SARS-CoV-2 positivity in offspring, and not trimester of maternal infection, gestation at birth, mode of delivery, breastfeeding, or mother-baby separation at birth. SARS-CoV-2 RNA is detected in amniotic fluid, placenta, vaginal fluid, and breast milk, but detection of virus in these biological specimens may not necessarily indicate infection in the baby.

### Strengths and limitations of this review

We carried out a comprehensive review on SARS-CoV-2 positivity rates in babies born to mothers with the infection and assessed the timing of exposure and likelihood of infection. As this is a living systematic review, we will be able to rapidly update the findings as new evidence emerges. We only included cohort studies for estimating the rates of SARS-CoV-2 positivity in offspring, unlike some of the published systematic reviews, which combined cohort and case-control studies with case series^18^ ^19^ ^20^ or reported positivity in babies as neonatal infection.^20^ ^21^ Our extensive sensitivity and subgroup analyses enabled us to assess the robustness of our findings according to the timing of maternal infection and testing in babies, and across regions. We used the detailed WHO classification system to ascertain the timing of transmission of SARS-CoV-2 from mother to baby and to confirm infection status, and we included data from any study that reported on babies with a positive test result. We assessed the various maternal and perinatal factors that may be associated with SARS-CoV-2 positivity in babies. Our extensive de-duplication process minimised the risk of double counting data.

Our findings were limited by heterogeneity in populations, tests, and outcomes. For example, severe and mild covid-19 were variably defined in the studies. Severe disease included severe symptoms, admission to an intensive care unit, and need for extracorporeal membrane oxygenation, and mild symptoms included asymptomatic women. Since almost all mothers in the studies had a recent diagnosis of SARS-CoV-2 infection, our findings are not applicable to those with infection in early pregnancy who recovered. Similarly, the types and timing of tests used in mothers and babies and their accuracy varied. Several studies did not provide details on the timing of perinatal exposure to SARS-CoV-2, or on the timing of tests, which hindered our ability to determine the timing of mother-to-child transmission of SARS-CoV-2. Even when the babies were tested, confirmatory tests were often not performed, further limiting our ability to use the WHO classification system to confirm infection status. Clinical outcomes of the babies born to mothers with SARS-CoV-2 infection were inconsistently reported, making it challenging to ascertain if the complications including stillbirths and neonatal deaths were related to SARS-CoV-2 or other clinical factors.

### SARS-CoV-2 positivity in offspring and timing of mother-to-child transmission

With low SARS-CoV-2 positivity rates in offspring, and only a small proportion of those found positive likely to have confirmed mother-to-child transmission, we expect an overall low risk of SARS-CoV-2 transmission to babies. Some studies used anti-SARS-CoV-2 IgM antibody testing to diagnose neonatal infection. But there are concerns about the accuracy of IgM antibody tests to diagnose vertical infection, and often a confirmatory IgM test was not performed.^12^ The low SARS-CoV-2 positivity rates in offspring in studies from Europe and North America could reflect the policy of universal maternal screening for SARS-CoV-2, resulting in inclusion of women with mild disease.^22^ ^23^ Since SARS-CoV-2 positivity in offspring was associated with severity of maternal disease, regions with mostly symptomatic testing of pregnant women were more likely to include women with severe disease, which may be reflected in the higher reported SARS-CoV-2 positivity rates in offspring in those regions. It is unlikely that change in SARS-CoV-2 virus variant would explain this difference, as the data collected from primary studies were all before the earliest identification of the alpha variant.

A previous systematic review that pooled data from all studies, including case series and reports without a formal meta-analysis, reported 70% of the 122 positive babies to have postpartum infections and 9% to have confirmed in utero and intrapartum infection, using the Shah classification.^18^ But we categorised fewer babies to have confirmed infection using the more stringent WHO criteria. We also refrained from providing the findings of confirmed infection as a proportion of all positive babies, because the selective reporting of SARS-CoV-2 positive babies in the studies affects the reliability of rate estimates.

The observed association between severe maternal disease and test positivity in offspring could be linked to detection of viral RNA in the blood associated with disease severity.^24^ ^25^ But to date, no clear evidence links the severity of maternal disease to the shedding of SARS-CoV-2, although the duration of shedding appears to be prolonged in individuals with severe covid-19.^26^ The observed association between postnatal diagnosis of maternal SARS-CoV-2 and neonates who test positive could also be attributed to horizontal transmission from the mother, caregivers, or health workers, or from the neonate’s environment. Appropriate measures to reduce the risk of horizontal transmission should be followed if infection is suspected, such as improved ventilation, adequate masks and mask wearing, hand hygiene, and use of protective clothing during contact with the baby.

We did not find any association between breastfeeding practice and SARS-CoV-2 positivity in neonates, consistent with rare findings of RT-PCR positivity in breast milk samples.^27^ Although we found evidence of SARS-CoV-2 positivity in various biological samples that could be associated with the potential for vertical infection—such as amniotic fluid, placenta, and vaginal secretions, finding a pathogen in such samples does not necessarily correlate with infection of the fetus.^9^ ^10^ ^11^ Studies did not always report whether the maternal or fetal side of the placenta was swabbed, making it difficult to accurately determine placental infection.

### Relevance for clinical practice and research

Our review provides estimates on the expected burden of SARS-CoV-2 positive test results in exposed babies in clinical practice who will require further testing and monitoring. Evidence was found of vertical transmission of SARS-CoV-2 through in utero and intrapartum routes, although the absolute number of confirmed cases is low. SARS-CoV-2 positivity in babies is likely to be higher when their mothers have severe covid-19, and relevant testing should be considered in these babies. Current evidence does not support caesarean sections, mother-baby separation at birth, or formula feeding as interventions for avoiding SARS-CoV-2 transmission to babies.

Healthcare professionals need to perform further tests in fetuses and babies with a positive result to robustly confirm infection occurred and classify timing of mother-to-child transmission using appropriate samples according to WHO guidance. To reduce the proportion of babies in whom we cannot confirm vertical transmission despite their initial positive status, repeat tests are needed at various time points in appropriate samples. Further research is needed to assess factors contributing to regional variations, such as different strategies for screening, severity of maternal disease, and emerging variants. Further data are needed on the SARS-CoV-2 positive status of the various biological samples that could be potentially associated with SARS-CoV-2 mother-to-child transmission, and the relationship of sample positivity to fetal or neonatal infection. It is important to consider the changing landscape of the covid-19 pandemic, including the prevalence of covid-19 in various regions, impact of vaccination, and the effects of known and emerging SARS-CoV-2 variants on mother-to-child transmission. Our living systematic review is well placed to update these findings on the growing evidence base.

### Conclusion

The overall rates of SARS-CoV-2 positivity in babies born to mothers with SARS-CoV-2 infection is low. Evidence was found for confirmed vertical transmission of the virus, although the absolute numbers are low. Severe maternal covid-19 may be associated with SARS-CoV-2 positivity in babies, but not vaginal delivery, breastfeeding, or mother-baby contact after birth.

**What is already known on this topic**

In pregnant women with SARS-CoV-2 infection, the virus and viral fragments have been detected in maternal blood, placenta, amniotic fluid, and breast milk, suggesting the potential for mother-to-child transmission
Primary studies and systematic reviews provide varied estimates for the rates of neonatal SARS-CoV-2 infection or positivity, or both
Current classification systems categorise the timing of SARS-CoV-2 mother-to-child transmission based on timing of exposure to the virus and type and timing of tests in offspring
People with severe covid-19 have high viral load

**What this study adds**

The overall rates of SARS-CoV-2 positivity in babies born to mothers with infection is low (<2%)
Evidence confirms mother-to-child transmission of SARS-CoV-2 through in utero, intrapartum, and early postpartum transmission, but vertical transmission is likely to be rare
Maternal factors such as severe covid-19, death, admission to intensive care unit, and postnatal infection seem to be associated with SARS-CoV-2 positivity in offspring

## Data Availability

No additional data available.

## Web extra

Extra material supplied by authors

Supplementary information: additional material

Supplementary information: figure 1 showing full list of other sources

## Notes

Contributors: ST, MB, and JA conceptualised the study. MY, SC, TK, ACL, AD, DZ, RB, SL, JS, HL, ML, YK, LD, GS, AB, LM, and KA selected the studies. JA, RC, TK, ES, MY, and SF extracted the data. JZ and AGG conducted the analyses. JA and SC are joint first authors. All authors contributed to writing the manuscript and approved the final version. ST, JA, LM, and JZ are the guarantors. The corresponding author attests that all listed authors meet authorship criteria and that no others meeting the criteria have been omitted.

The lead authors (ST, JA, LM, and JZ) affirms that the manuscript is an honest, accurate, and transparent account of the study being reported; that no important aspects of the study have been omitted; and that any discrepancies from the study as planned (and, if relevant, registered) have been explained.

Dissemination to participants and related patient and public communities: The PregCOV-19 Living Systematic Review Group will disseminate the findings through a dedicated website (www.birmingham.ac.uk/research/who-collaborating-centre/pregcov/index.aspx), and through social media.

Provenance and peer review: Not commissioned; externally peer reviewed.
