# SARS-CoV-2 positivity in offspring and timing of mother-to-child transmission: living systematic review and meta-analysis

**DOI:** 10.1136/bmj-2021-067696

**Published:** 2022-03-16

**Authors:** John Allotey, Tania Kew, Silvia Fernández-García, Andrea Gaetano-Gil, Magnus Yap, Jameela Sheikh, Megan Littmoden, Oluwadamilola Akande, Halimah Khalil, Maurie Kumaran, Kathryn Barry, Shruti Attarde, Dharshini Sambamoorthi, Anoushka Ramkumar, Heidi Lawson, Millie Manning, Sophie Maddock, Ankita Gupta, Meghnaa Hebbar, Alya Khashaba, Kehkashan Ansari, Adeolu Banjoko, Kate Walker, Keelin O’Donoghue, Madelon van Wely, Elizabeth van Leeuwen, Elena Kostova, Heinke Kunst, Asma Khalil, Vanessa Brizuela, Edna Kara, Caron Rahn Kim, Anna Thorson, Olufemi T Oladapo, Javier Zamora, Mercedes Bonet, Lynne Mofenson, Shakila Thangaratinam

**Affiliations:** 1 WHO Collaborating Centre for Global Women’s Health, Institute of Metabolism and Systems Research, University of Birmingham, Birmingham, B15 2TT, UK; 2 Birmingham Medical School, University of Birmingham, Birmingham, UK; 3 Clinical Biostatistics Unit, Hospital Universitario Ramón y Cajal (IRYCIS), Madrid, Spain; 4 CIBER Epidemiology and Public Health (CIBERESP), Madrid, Spain; 5 University of Nottingham, Nottingham, UK; 6 University College Cork, Cork, Ireland; 7 Netherlands Satellite of the Cochrane Gynaecology and Fertility Group, Amsterdam University Medical Centre, Amsterdam, Netherlands; 8 Department of Obstetrics and Gynaecology, Amsterdam University Medical Centre, Amsterdam, Netherlands; 9 Blizard Institute, Queen Mary University of London, London, UK; 10 Barts Health NHS Trust, London, UK; 11 St George’s University London, London, UK; 12 UNDP/UNFPA/UNICEF/WHO/World Bank Special Programme of Research, Development and Research Training in Human Reproduction (HRP), Department of Sexual and Reproductive Health and Research, World Health Organization, Geneva, Switzerland; 13 Elizabeth Glaser Paediatric AIDS Foundation, Washington, DC, USA; 14 Birmingham Women’s and Children’s NHS Foundation Trust, Birmingham, UK

## Abstract

**Objectives:**

To assess the rates of SARS-CoV-2 positivity in babies born to mothers with SARS-CoV-2 infection, the timing of mother-to-child transmission and perinatal outcomes, and factors associated with SARS-CoV-2 status in offspring.

**Design:**

Living systematic review and meta-analysis.

**Data sources:**

Major databases between 1 December 2019 and 25 April 2022.

**Study selection:**

Cohort studies of pregnant and recently pregnant women (including after abortion or miscarriage) who sought hospital care for any reason and had a diagnosis of SARS-CoV-2 infection, and also provided data on offspring SARS-CoV-2 status and risk factors for positivity. Case series and case reports were also included to assess the timing and likelihood of mother-to-child transmission in SARS-CoV-2 positive babies.

**Data extraction:**

Two reviewers independently extracted data and assessed study quality. A random effects model was used to synthesise data for rates, with associations reported using odds ratios and 95% confidence intervals. Narrative syntheses were performed when meta-analysis was inappropriate. The World Health Organization classification was used to categorise the timing of mother-to-child transmission (in utero, intrapartum, early postnatal).

**Results:**

643 studies (343 cohort studies, 300 case series and case reports; 44 552 mothers, 30 822 babies) were included. Overall, 2.7% (95% confidence interval 2.1% to 3.5%; 210 studies, 24 040 babies) born to mothers with SARS-CoV-2 infection tested positive for the virus with reverse transcriptase polymerase chain reaction (RT-PCR). Of the 1107 SARS-CoV-2 positive babies with data on the timing of exposure and type and timing of tests, 32 had confirmed mother-to-child transmission: 20 in utero (857 assessed), three intrapartum (35 assessed), and nine during the early postnatal period (144 assessed). Of the 1213 SARS-CoV-2 positive babies with outcome data, 64 were stillbirths, 36 were neonatal deaths, and nine were early pregnancy losses; 1104 babies were alive at the end of follow-up. Severe maternal covid-19 (odds ratio 3.5, 95% confidence interval 1.5 to 8.1), maternal death (14.1, 4.1 to 48.0), maternal postnatal infection (5.0, 1.2 to 20.1), caesarean section (1.4, 1.1 to 1.8), and preterm delivery (1.5, 1.2 to 1.9) were associated with SARS-CoV-2 positivity in offspring. Positivity rates in offspring using RT-PCR varied between regions, ranging from 0.1% (95% confidence interval 0.0% to 0.5%) in studies from North America to 8.5% (4.6% to 13.3%) in studies from Latin America and the Caribbean.

**Conclusion:**

SARS-CoV-2 positivity rates are low in babies born to mothers with SARS-CoV-2 infection. Evidence confirms vertical transmission of SARS-CoV-2, although this is rare. Severity of maternal covid-19 is associated with SARS-CoV-2 positivity in offspring.

**Systematic review registration:**

PROSPERO CRD42020178076.

**Readers’ note:**

This article is a living systematic review that will be updated to reflect emerging evidence. Updates may occur for up to two years from the date of original publication. This version is update 1 of the original article published on 16 March 2022 (*BMJ* 2022;376:e067696), and previous updates can be found as data supplements (https://www.bmj.com/content/376/bmj-2021-067696/related#datasupp).

## Introduction

Maternal infection with SARS-CoV-2 has raised concerns about the potential for mother-to-child transmission of the virus.^1^ Although there is robust evidence on the magnitude and modes of SARS-CoV-2 transmission in the general population and the prevalence of test positivity,^2^ little is known about the burden of SARS-CoV-2 positivity in babies born to infected women. Existing primary studies vary widely in the reported rates of SARS-CoV-2 test positivity and the definition and timing of transmission from exposure to the virus in utero or during the intrapartum and postnatal periods.^3^ ^4^ ^5^ ^6^ Our earlier published systematic review on SARS-CoV-2 positivity in offspring and timing of mother-to-child transmission reported low positivity rates, and although evidence suggested vertical transmission of the virus, transmission was thought to be rare.^7^ Further information is needed on the rates of mother-to-child transmission of the virus following the emergence of SARS-CoV-2 variants of concern, and the roll out of vaccinations globally.^8^ ^9^

To confirm infection and accurately determine when transmission of SARS-CoV-2 occurs, appropriately timed and repeated tests are needed in relevant samples.^10^ ^11^ Detection of SARS-COV-2 in specimens from the placenta, amniotic fluid, or neonate (eg, non-sterile specimens such as nasopharyngeal or faecal) using reverse transcriptase polymerase chain reaction (RT-PCR) alone is not sufficient to diagnose fetal infection.^12^ ^13^ ^14^ The accuracy of anti-SARS-CoV-2 IgM assays for serological diagnosis of congenital infection also varies.^15^ ^16^ Furthermore, as timing and route of infection may affect clinical outcomes, we need to be able to differentiate between intrapartum transmission of the virus and infection acquired soon after birth through contact with mother, caregivers, healthcare workers, or the neonate’s environment.^14^

The clinical outcomes in SARS-CoV-2 positive babies and those with confirmed vertical infection also need to be ascertained. The extent to which maternal factors such as severe covid-19, timing of infection, mode of delivery, breastfeeding, and postnatal contact with offspring are associated with SARS-CoV-2 positivity in babies is needs to be known to inform maternal care. 

In our living systematic review, we assess the rates of SARS-CoV-2 positivity in babies born to mothers with SARS-CoV-2 infection, the timing of mother-to-child transmission, perinatal outcomes in positive babies, and factors associated with SARS-CoV-2 positivity in offspring. In this update, we address the above unknowns, and additionally investigate the association between SARS-CoV-2 variants and mother-to-child transmission of the virus.

## Methods

Our systematic review is based on a prospective living protocol (PROSPERO CRD42020178076; registered 22 April 2020). In this paper, we focus on mother-to-child transmission using the preferred reporting items for systematic reviews and meta-analyses (PRISMA) recommendations (see supplementary appendix 1).

### Search strategy

We searched major databases, preprint servers, and websites that serve as repositories for covid-19 studies, including Medline, Embase, Cochrane database, WHO COVID-19 database, Living Overview of the Evidence platform, China National Knowledge Infrastructure (CNKI), and Wanfang databases for studies (cohort, case series, and case report) on SARS-CoV-2 infection in pregnant and recently pregnant women (including after abortion or miscarriage). For this update of the living systematic review, we included studies from searches up to 25 April 2022. No language restrictions were applied. Our searches were coordinated with the EPPI-Centre, the WHO (World Health Organization) Library, and the Cochrane Gynaecology and Fertility group (see supplementary appendix 2).

### Study selection

Eighteen reviewers contributed to study selection. Two independent reviewers assessed each study using a two stage process. In the first stage, the titles and abstracts of all citations were screened, and the full texts examined for inclusion in the second stage. Disagreements between reviewers were resolved through discussion with a third reviewer (ST, JA, or SF-G). To assess SARS-CoV-2 positivity rates in offspring, we included cohort studies of pregnant and recently pregnant women who sought hospital care for any reason and had a diagnosis of SARS-CoV-2 infection, and where SARS-CoV-2 status was ascertained in the fetus or neonate using RT-PCR (neonatal pharyngeal, rectal, or faecal swabs, neonatal or cord blood, fetal tissue, placental samples, or amniotic fluid) or serological tests (anti-SARS-CoV-2 IgM), or both. We defined cohort studies as those that sampled consecutive women, who were followed-up to ascertain the SARS-CoV-2 status of their offspring within the first 30 days after birth.^17^ Unless specified otherwise, we use the term babies and offspring to denote both fetuses and neonates.

In addition to the cohort studies, we included case series and case reports to assess the timing of mother-to-child transmission and likelihood of infection. To evaluate maternal risk factors for offspring SARS-CoV-2 positivity, we included cohort studies of pregnant and recently pregnant women with a diagnosis of SARS-CoV-2 infection that reported on maternal and perinatal risk factors such as maternal severe covid-19, admission to an intensive care unit, and death; timing of exposure to the virus (antenatal *v* postnatal; third *v* first or second trimester); intrapartum factors (<37 weeks preterm *v* term); mode of delivery (caesarean section *v* vaginal birth); timing of cord clamping (immediate *v* delayed)); postnatal care (skin-to-skin contact *v* none; not separated *v* separated at birth; breastfed versus not breastfed); and SARS-CoV-2 infection status of the offspring. In this update, we also extracted information about the SARS-CoV-2 variant and SARS-CoV-2 status of offspring born to mothers who were infected.

### Quality assessment and data extraction

We assessed the internal and external validity of non-comparative cohorts using the tool by Hoy et al.^18^ For internal validity, we considered studies to be at low risk of bias if data were collected from clinical records or research case report forms (data collection), clearly defined outcomes (case definition), confirmed SARS-CoV-2 infection using laboratory based tests (instrument validity), used same mode of data collection in all participants (ascertainment bias), and had sufficient follow-up, with appropriate numerator and denominator. For external validity, we considered studies to be at low risk of bias if they were representative of the national population for relevant variables (population), representative of the target population (sampling frame), undertook a census (selection bias), and the response rate of individuals with and without the outcome was more than 75% (non-response bias). We assessed the methodological quality of the comparative cohort studies using the Newcastle Ottawa scale for selection, comparability, and outcome ascertainment bias outcome.^19^

Using a pre-piloted form, six independent reviewers in two sets extracted data on study design, number of pregnant women with SARS-CoV-2 infection, type of SARS-CoV-2 test in mothers and babies (RT-PCR, IgM), maternal characteristics (including stage of pregnancy at diagnosis), severity of covid-19 (as defined by authors), mode of delivery, type of samples tested (neonatal nasopharyngeal, rectal, or faecal swabs, neonatal or cord blood, fetal tissue, placenta, amniotic fluid, vaginal fluid, breast milk), timing of sample collection, and SARS-CoV-2 predominant variant of concern^20^ (as reported by authors, or mapped by the dominant variant in the country during the study period using https://covariants.org/).^9^ We also extracted data on the clinical outcomes of all SARS-CoV-2 positive fetuses and neonates when available, including early pregnancy outcomes of miscarriage and abortion. A detailed deduplication process was used to cross check data against other studies published by the same authors or those that included women from the same institutions. We contacted study authors for unpublished information and to query duplication of data.

### Data analysis

We summarised the SARS-CoV-2 positivity rates in offspring identified by RT-PCR or anti-SARS-CoV-2 IgM assays, or both, as a proportion of all babies born to mothers with SARS-CoV-2 infection in cohort studies. After transforming data using Freeman-Tukey double arcsine transformation, we used DerSimonian and Laird random effects meta-analysis to calculate rates and corresponding 95% confidence intervals. Heterogeneity was reported as I^2^ and τ^2^ estimates. Sensitivity analysis for SARS-CoV-2 positivity rates in babies was done by restricting the analysis to studies at low risk of bias, babies tested at less than 24 h after birth, and babies born to mothers with SARS-CoV-2 infection diagnosed antenatally. The rates of SARS-CoV-2 positivity were also evaluated by subgroups of studies involving babies and mothers from various World Bank regions and by predominant SARS-CoV-2 variants of concern.

We ascertained the timing of mother-to-child transmission based on the World Health Organization classification in all studies (cohort, case series, case reports) that reported SARS-CoV-2 positive babies and provided information on the timing of exposure (antenatal, postnatal) and test timings in the babies (see supplementary appendix 3).^21^ Each baby with a positive test result was placed in mutually exclusive categories for likelihood of infection: confirmed (definite infection), possible (evidence suggestive of infection but not confirmatory), unlikely (infection cannot be ruled out), and indeterminate (tests required to define classification have not been performed) for in utero, intrapartum, or early postnatal transmission. In addition to the specifications in the WHO criteria, we categorised babies to have confirmed or possible in utero infection if they had a positive test result in the first 24 hours after birth and did not have a test between 24 h and 48 h but had a repeat positive test result from a sterile (confirmed) or non-sterile (possible) sample after 48 h and before seven days, with no negative test results before the repeat positive test result. We also added one further “indeterminate” category for intrapartum transmission: when babies had a negative test result or no test in the first 24 h after birth and a single anti-SARS-CoV-2 IgM positive result at 7-14 days with no confirmatory test; and a further “indeterminate” category for postpartum transmission: when babies had a negative test result in the first 48 h after birth with a single positive non-sterile sample after 48 h or IgM result at more than 14 days with no or negative confirmatory test result (see supplementary appendix 3 for revised classification).

To summarise the associations between maternal and perinatal characteristics and SARS-CoV-2 status in exposed babies, we pooled comparative dichotomous data as odds ratios and 95% confidence intervals using random effects meta-analysis. When meta-analysis was considered inappropriate because of excessive clinical or statistical heterogeneity or when SARS-CoV-2 positive offspring were selectively reported in the cohort studies, we used a narrative descriptive approach to summarise the evidence, such as for clinical outcomes in test positive babies and test positivity in various biological samples. All statistical analyses were performed using Stata (version 16).

### Patient and public involvement

This study is supported by Katie’s Team (https://www.elly.org.uk/copy-of-research) and The Hilda’s (https://www.dhlnetwork.com/news), dedicated patient and public involvement groups in women’s health. The team members were involved in the interpretation and reporting of this living systematic review through participation in virtual meetings. Findings will be made available on our website in a format more suitable for patients and members of the public (www.birmingham.ac.uk/research/who-collaborating-centre/pregcov/index.aspx).

## Results

**Fig 1:**
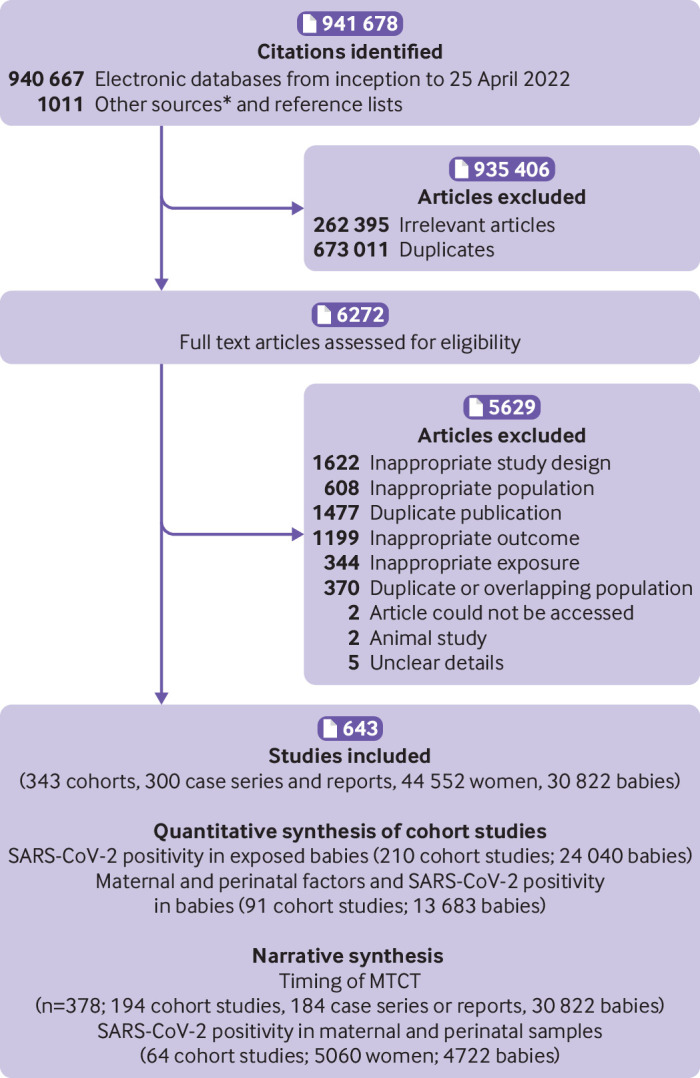
Study selection process in systematic review: SARS-CoV-2 positivity in babies born to mothers with covid-19 and timing of MTCT (mother-to-child transmission). *See supplementary figure for details of other sources

Overall, we included 643 studies (343 cohort studies, 300 case series and case reports; 44 552 mothers, 30 882 babies) from 941 678 identified articles (fig 1). In 554 studies, women had infections prior to the emergence of any SARS-CoV-2 variants of concern (wild type), six studies during predominance of variants of interest or variants under monitoring, 18 studies during predominance of the alpha variant, two studies each during predominance of the beta and gamma variants, and five studies during predominance of the delta variant. None of the studies was conducted during predominance of the omicron variant, and 56 studies did not report the predominant variant, or provide information required to identify the dominant variant in the country during the study period. A total of 210 cohort studies reported on SARS-CoV-2 positivity status in 24 040 exposed babies. Overall, 1567 babies tested positive for SARS-CoV-2 across all study designs (378 studies; 194 cohorts, 184 case series or case reports). Ninety one comparative cohorts (with 13 683 mother-baby dyads) reported on various maternal and perinatal factors and SARS-CoV-2 positivity in offspring. In 246 cohort studies, SARS-CoV-2 testing of various maternal and perinatal biological samples (placenta, amniotic fluid, maternal vaginal fluid, stool samples, and breast milk) were reported in a proportion of participants (5060 mothers, 4722 babies).

### Characteristics of included studies

Most of the included studies were from the World Bank regions of Europe and Central Asia (215/643, 33.5%), followed by North America (118/643, 18.5%), South Asia (84/643, 13%), East Asia and Pacific (80/643, 12.5%), and Middle East and North Africa (80/643, 12.5%), Latin America and the Caribbean (58/643, 9%), and eight studies were from Sub-Saharan Africa (8/643, 1%). Maternal infection was confirmed by laboratory tests in 99% (634/643) of the studies. The most common test to ascertain infection in offspring was RT-PCR in 97% of cohort studies (209/215); 11% (24/215) of cohort studies used either anti-SARS-CoV-2 IgM alone or with RT-PCR (see supplementary appendix 4).

### Quality of included cohort studies

Our internal validity assessment of the non-comparative cohorts showed a low risk of bias for data collection in 81% (265/326) of the studies, 63% (206/326) for case definition, 100% (326/326) for measurement, 98% (320/326) for differential verification, 71% (230/326) for adequate follow-up, and 87% (285/326) for appropriate numerator and denominator. For external validity, the studies had low risk of bias for representativeness in 6% (18/326) of the studies, 24% (79/326) for sampling, 91% (296/326) for selection, and 98% (319/326) for non-response. The overall risk of bias (Newcastle Ottawa scale) for the included comparative cohort studies was low in 98% (89/91) of studies; 97% (88/91) had low risk of bias for study selection, 32% (29/91) for comparability of cohorts, and 79% (72/91) for outcome assessment (see supplementary appendix 5).

### SARS-CoV-2 positivity in exposed babies in cohort studies

**Fig 2:**
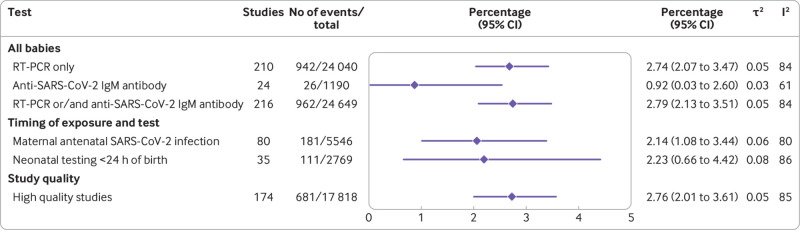
Rates of SARS-CoV-2 positivity in babies (including fetuses) born to mothers seeking hospital care for any reason and having active or recently diagnosed SARS-CoV-2 infection. RT-PCR=reverse transcriptase polymerase chain reaction

SARS-CoV-2 positivity using RT-PCR was observed in 2.7% (95% confidence interval 2.1% to 3.5%) of all babies (n=24 040) born to mothers with a diagnosis of SARS-CoV-2 infection (210 cohort studies); 2.8% (2.1% to 3.5%) tested positive when studies used either RT-PCR or anti-SARS-CoV-2 IgM tests (216 studies, 24 649 babies). Anti-SARS-CoV-2 specific IgM antibodies were shown in 0.9% (95% confidence interval 0.0% to 2.6%) of exposed babies who were tested (24 studies, 1190 babies) (fig 2). In sensitivity analysis, the SARS-CoV-2 RT-PCR positivity rate limited to high quality studies was 2.8% (95% confidence interval 2.0% to 3.6%) in babies born to mothers with SARS-CoV-2 infection, a finding similar to that of the main analysis. When the analysis was limited to babies of mothers with a diagnosis of SARS-CoV-2 infection in the antenatal period, the positivity rate was 2.1% (1.1% to 3.4%); 2.2% (0.7% to 4.4%) when limited to babies tested in the first 24 h after birth (fig 2). In the subgroup analyses, SARS-CoV-2 positivity rates by RT-PCR in offspring varied between regions, ranging from 0.1% (0.0% to 0.5%) in studies from North America to 8.5% (4.6% to 13.3%) in studies from Latin America and the Caribbean. Offspring rates of SARS-CoV-2 positivity by RT-PCR also varied by predominance of SARS-CoV-2 variants of concern. Most studies were conducted before predominance of a variant of concern with positivity rate of 2.5% (1.9% to 3.2%). Positivity rates for the variants of concern ranged from 1.7% (0.3% to 9%) in a study with women who were infected during predominance of gamma variant, to 4.3% (2.5% to 7.4%) in a study during predominance of the delta variant. Positivity rates were higher (13.7% (0.0% to 42.3%)) when the predominant variant during the study period was unknown (see supplementary appendix 6).

### Timing of mother-to-child transmission

**Fig 3:**
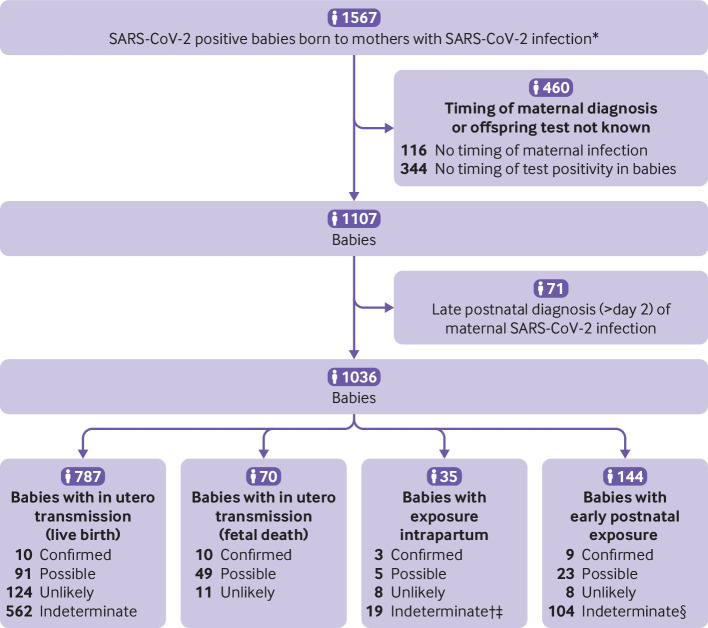
Flowchart showing inclusion of babies classified by timing of SARS-CoV-2 mother-to-child transmission using the World Health Organization classification system. *Clinical and laboratory diagnosis. †Category added to existing WHO classification. ‡Babies with positive serology at days 7-14, but no confirmatory test done. §Includes 29 babies with negative test ≤48 h, then positive test >48 h with no further or negative confirmatory test

Of the 28 350 babies born to mothers with SARS-CoV-2 infection across all studies (cohorts, case series, case reports), data were sufficient to apply the WHO classification system for timing of exposure and likelihood of mother-to-child transmission in 1107 babies with a positive test result, including 857 babies with a first positive test at <24 h, 35 babies with a negative test result at <24 h but positive at 24-48 h, and 144 babies with a negative test at <48 h and a positive test at >48 h (fig 3). After exclusion of 71 babies where maternal SARS-CoV-2 infection was diagnosed late (>2 days postnatally), 32 of the 1036 babies (including fetuses) were categorised as having confirmed infection (10/787 live births with testing consistent with in utero transmission, 10/70 fetal deaths or miscarriages with in utero transmission, 3/35 intrapartum, and 9/144 early postnatal infection), and 168 as possible infection (91/787 live births with testing consistent with in utero transmission, 49/70 fetal deaths or miscarriages with in utero transmission, 5/35 intrapartum, and 23/144 early postnatal infection) (fig 3). The likelihood of mother-to-child transmission was classified as indeterminate for 685 babies, mainly owing to the lack of repeat confirmatory testing within the prespecified time points. Table 1 22 23 24 25 26 27 28 29 30 31 32 33 34 35 36 37 38 39 40 and table 2 33 41 42 43 44 45 46 and supplementary appendix 7 provide the maternal and perinatal characteristics and SARS-CoV-2 test results of the babies with confirmed and possible vertical infection, respectively.

**Table 1:** Maternal and perinatal characteristics of live births with confirmed in utero, intrapartum, and early postnatal transmission of SARS-CoV-2^22^ ^23^ ^24^ ^25^ ^26^ ^27^ ^28^ ^29^ ^30^ ^31^ ^32^ ^33^ ^34^ ^35^ ^36^ ^37^ ^38^ ^39^ ^40^

Author, year	Maternal characteristics	Mode of delivery	Measures to prevent SARS-CoV-2 MTCT		Tests for SARS-CoV-2 MTCT				Fetal and neonatal characteristics	
					Initial test		Further tests			
**Livebirths**										
Correia CR, 2020	Age 40 years, pre-eclampsia and previous risk of preterm delivery Symptoms of covid-19 NP PCR + at 34 weeks before delivery Stool PCR+	Caesarean section	Delivery in negative pressure room No skin-to-skin contact		Blood PCR+ at 0.5 h NP PCR+ at 0.5 h		Deep tracheal aspirate PCR+ at 48 h, and on D9, D15, D19 Blood IgM and IgG initially negative on D3, D7, and D11, and then positive on D15 Stool PCR+ on D7		34 weeks gestational age 1510 g birthweight Apgar: 8, 9 Required positive airway pressure ventilation, admitted to NICU Alive	
Disse SC, 2021 (baby 1 of a case of triplets; baby 3 classified as possible in utero)*	Age 36 years, G2P1 Moderate respiratory symptoms NP PCR+ at 27 weeks	Caesarean section	Not reported		Placental tissue IHC+ NP PCR+ at 0 h Blood PCR and IgG negative at 0 h		Tracheal aspirate PCR+ on D3, D10, D14 NP PCR+ on D3, D5, and week 3; negative in week 4		Trichorionic triplets 28 weeks gestational age 1150 g birthweight Apgar: 8, 9, 9 Admitted to NICU on non-invasive intermittent positive pressure ventilation Alive	
Disse SC, 2021 (baby 2)	Age 36 years, G2P1 Moderate respiratory symptoms NP PCR+ at 27 weeks	Caesarean section	Not reported		Placental tissue IHC+ NP PCR neg at 0 h Blood PCR and IgG negative at 0 h		Tracheal aspirate PCR+ on D2 NP PCR neg on D2 NP PCR+ on D5, D10, D14; and negative in weeks 3 and 4		Trichorionic triplets 28 weeks gestational age 930 g birthweight Apgar: 8, 9, 9 Admitted to NICU on non-invasive intermittent positive pressure ventilation Alive	
Ferreira MFC, 2022*	Symptoms of coryza, hyposmia and ageusia NP PCR+	Caesarean section	Neonate transferred to isolation NICU in a transport incubator		Placenta PCR+ Bronchoalveolar aspirate PCR+ at <24 h Cord IgM and IgG negative		Bronchoalveolar aspirate PCR+ on D5, and negative on D15		33 weeks gestational age CT chest showed a viral pattern of infection in the lungs Alive	
Isidro EMM, 2021*	Asymptomatic NP PCR+ at 28 weeks	Vaginal delivery	Neonate admitted to an individual room in NICU under contact and droplet isolation measures		NP PCR+ at 2 h		Bronchoalveolar lavage PCR+ on D3 NP PCR+ on D3, D7, D12, D15, and D21 Blood IgM and IgG negative on D1, D12, D15, and D33 Blood PCR negative on D10 Faecal sample PCR+ on D10		29 weeks gestational age 1455 g birthweight Apgar: 6, 8 Neonate required intubation at birth CXR showed bilateral reticular interstitial pattern suggesting neonatal respiratory distress syndrome Alive	
Lima ARO, 2020*	Age 27 years, G2 (para not reported), no comorbidities Flu-like symptoms at 29 weeks Rapid serological test IgM+ and IgG+ at 32 weeks	Caesarean section	Mother wore N95 mask during delivery in isolated operative room Neonate immediately separated from mother Breastfed from D7		Blood and NP swab PCR+ at 1 h Cord blood IgM negative but IgG+ Peripheral blood at birth IgM negative but IgG+ Placenta and amniotic fluid PCR negative Chorion PCR inconclusive		Blood PCR+ on D5 NP PCR+ on D5, the negative on D13 and D14		33 weeks gestational age 2400 g birthweight Apgar: 7, 9 Fetal echocardiogram at 32 weeks showed high risk of cardiac tamponade, leading to emergency caesarean section Prophylactic steroids given for fetal lung maturation Bag-mask ventilation at birth, then transferred to NICU CT scan showed some lung changes On D3, became unstable and intubated Pericardial drain inserted Extubated on D7 Alive on discharge	
Ng DCE, 2021	Age 39 years, primigravid Symptoms of fever and cough Signs of pneumonia on CXR NP PCR+ at 29 GW	Preterm labour, spontaneous vaginal delivery	Mother wore surgical mask during delivery Separated from mother at birth		NP PCR+ at 2 h Blood IgM and IgG negative at birth		Tracheal aspirate PCR + at 26 h Blood IgM and IgG + on D14		29 weeks gestational age 1100 g birthweight APGAR: 9, 9 Symptomatic, respiratory distress, required non-invasive CPAP ventilation Bilateral ground glass opacities on CT scan Alive	
Reagan-Steiner S, 2022	Age 34 years, G4P3 Pre-eclampsia Asymptomatic NP PCR+	Caesarean section	Neonate placed under airborne, contact and droplet precautions in NICU		Placental tissue PCR+		NP PCR+ at 24 h and 72 h Fetal tissue PCR+ at >D4		25 weeks gestational age 670 g birthweight APGAR: 1, 4, 7 CXR showed widespread bilateral airspace consolidation Died on D4 of life due to acute bradycardic event and respiratory acidosis	
Vivanti A, 2021	Age 29 years, nulliparous CT chest showed moderate pneumonia NP PCR+	Caesarean section	Not reported		Placenta PCR+ Amniotic fluid PCR+		Bronchoalveolar lavage PCR+ on D1 NP PCR+ at <48 h		33 weeks gestational age 2130 g birthweight AGPAR: 2, 5 Neonate required invasive ventilation and oxygen for mild perinatal asphyxia Alive	
Yangin Ergon E, 2021*	Age 34 years NP PCR+ Symptoms of fever Chest CT consistent with bilateral covid-19 pneumonia	Caesarean section	Neonate monitored in an isolated negative pressure room		NP PCR+ at 0 h and 24 h		Tracheal aspirate PCR+ on D3, D8, D11, D17 Blood IgM and IgG+ on D5		34 weeks gestational age 2460 g birthweight APGAR: 4, 7 CXR showed bilateral ground-glass opacities	
**Confirmed intrapartum MTCT**										
Urban A, 2021	Age 24 years, primigravid NP PCR+ at 39 weeks	Caesarean section	Mother wore surgical mask during caesarean section, neonate immediately isolated from mother		NP PCR negative at 5 h		NP PCR+ at 48 h, on D5, D8, D13, D18 NP PCR+ negative on D24		39 weeks gestational age 3430 g birthweight Alive	
Zeng L, 2020 (twin 1)	Nasopharyngeal PCR+ just before delivery Fever and pneumonia (CT scan), not admitted to ICU No information on maternal characteristics	Caesarean section	Neonate separated from mother after birth Not breastfed		Amniotic fluid PCR negative Cord blood PCR negative		NP PCR+ on D2 of life Confirmed with NP PCR+ on D4		40 weeks gestational age 3250 g birthweight Lethargy and fever Pneumonia on chest x ray Admitted to NICU alive	
Zeng L, 2020 (twin 2)		Caesarean section	Neonate separated from mother after birth Not breastfed		Amniotic fluid PCR negative Cord blood PCR negative		NP PCR+ on D2 of life Confirmed with NP PCR+ on D4		40 weeks gestational age 3360 g birthweight Lethargy, vomiting and fever Pneumonia on chest x-ray	
**Confirmed early postpartum MTCT**										
Bastug A, 2020	Age 20 years, G2P2, diagnosed at 39 weeks, asymptomatic Mother NP PCR+ just before delivery Breast milk PCR+	Vaginal delivery		Mother wore mask during delivery and when expressing breast milk Neonate separated from mother after birth and consumed expressed breast milk		NP PCR negative on D1		Peripheral blood PCR+ on D4 of life		39 weeks gestational age 2980 g birthweight Admitted to NICU Asymptomatic Alive	
Demirjian A, 2020	Age 34 years, G3P2, 38 weeks Mother had severe symptoms of increasing dyspnoea requiring intubation (ICU) Sputum PCR+ just before delivery Maternal blood PCR+	Caesarean section		Neonate separated from mother after birth, and exclusively formula fed		NP and rectal PCR negative on D1, negative peripheral blood on D1 CSF PCR negative on D1		NP PCR+ on D4 Rectal PCR+ on D7 and NP PCR+ on D8 (note: rectal PCR negative on D4) Peripheral blood PCR+ on D7 (but was PCR negative on D5)		39 weeks gestational age 4170 g birthweight APGAR: 5,9,9 Symptomatic: fever, coryza and mild tachypnoea Alive	
Gordon M, 2020	Age 36 years, G3P0 with infection diagnosed at 32 weeks Symptomatic with cough, high fever and lymphopaenia Mother NP PCR+ just before delivery	Caesarean section		Mother wore a mask during delivery Neonate separated from mother after birth		NP PCR negative on D1		NP PCR+ on D4, confirmed with NP PCR+ on D14 (with further NP PCR+ on D21 and D29)		32 weeks gestational age 2150 g birthweight Radiography: findings consistent with surfactant deficiency lung disease. Alive	
Gupta V, 2022	NP PCR+	Not reported		NP PCR+		NP PCR negative at 24 h		NP PCR+ on D5 and D7		Asymptomatic Alive	
Ibrahim CPH, 2021 (Triplet 2; triplets 1 and 3 classified as possible in utero)	Age 23 years, G3P2 NP PCR+ at 29 weeks	Caesarean section		Neonate admitted to isolation rooms on NICU		NP PCR negative at 24 h		NP PCR+ at 72 h, D6, D9, D12, D15 NP PCR negative on D18; positive on D19; negative on D48		Triplets 29 weeks gestational age 1270 g birthweight Neonate developed respiratory distress syndrome Alive	
Komiazyk M, 2020	Age 28 years, asymptomatic Mother NP PCR+ just before delivery (results known after delivery)	Vaginal delivery		Skin-to-skin contact after birth Separated later when mother’s PCR result known		NP PCR negative on D1		NP PCR+ on D5 of life, confirmed with NP PCR+ on D10		40 weeks gestational age APGAR: 10 Asymptomatic Alive	
Ong TG, 2021	NP PCR+	Not reported		Neonate immediately isolated in NICU after birth without physical contact with parents		NP PCR negative on D1		NP PCR negative on D3 NP PCR+ on D5 and D12		37 weeks gestational age 2980 g birthweight Hypoxic ischaemic encephalopathy Alive	
Vigil-Vazquez S, 2022	Symptoms of dyspnoea and fever NP PCR+	Not reported		Not reported		NP PCR negative on D1		NP PCR+ on D16 and D30		Alive	
Yu ZY, 2020	G1P0, symptomatic, 38 weeks Mother NP PCR+ on D1PN	Caesarean section		Neonate roomed in with mother Not breastfed		Cord blood PCR negative		NP PCR+ on D7 of life and confirmed with NP PCR+ on D15		3600 g birthweight APGAR: “normal” Symptomatic with fever Chest x ray showed diffuse consolidation Alive	

MTCT=mother-to-child-transmission (according to World Health Organization classification); NP RT-PCR= nasopharyngeal reverse transcriptase polymerase chain reaction; Dx=day number; G2P1=gravida 2, parity 1; NICU=neonatal intensive care unit; IHC=Immunohistochemistry; IUFD=intra-uterine fetal death; D1PN=1 day after birth.

* Classified as “confirmed” in utero as tests were repeated <24 h, despite not meeting WHO criteria of positive test result at 24-48 h (appendix 3).

**Table 2:** Maternal and perinatal characteristics of fetal death with confirmed in utero mother-to-child transmission of SARS-CoV-2^33^ ^41^ ^42^ ^43^ ^44^ ^45^ ^46^

Author, year	Maternal characteristics	Mode of delivery	Measures to prevent SARS-CoV-2 MTCT	Tests for SARS-CoV-2 MTCT		Fetal and neonatal characteristics
				Initial test	Further tests	
Babal P, 2021	Age 32 years; NP PCR+	Vaginal delivery	Not reported	Fetal tissues PCR+	Placental tissue PCR+, IHC+, ISH+ Umbilical cord tissue PCR+	28 weeks gestational age 3315 g birthweight
Lesieur E, 2021	Age 40 years, G3P2; severe symptoms of cough and fever; NP PCR+ at 23 weeks	Vaginal delivery	Not reported	Fetal tissues PCR+	Placental tissue PCR+ and IHC+	24 weeks gestational age 528 g birthweight
Patane L, 2022 (twin 1)	Age 35 years; NP PCR+	Vaginal delivery (stillbirth)	Not reported	Fetal tissues PCR+	Placental tissue PCR+, IHC+, ISH+ Cord PCR+	21 weeks gestational age
Patane L, 2022 (twin 2)	Age 35 years; NP PCR+	Vaginal delivery (stillbirth)		Fetal tissues PCR+	Placental tissue PCR+, IHC+, ISH+ Cord PCR+	21 weeks gestational age
Rodrigues M, 2020	Age 19 years, no past medical history; nasopharyngeal PCR+ just before delivery; symptomatic	Vaginal delivery (stillbirth)	Not reported	Fetal tissues PCR+ on autopsy		No fetal heartbeat at 34 weeks, small for gestational age (third centile) 1460 g birthweight
Valdespino-Vazquez, MY 2020 (twins)	Age 28 years, G4P3 Fever, headache, arthralgia, fatigue at 13 weeks Also had dark vaginal bleeding NP PCR initially negative but became positive	Vaginal delivery (miscarriage)	Not reported	Fetal organs PCR+, immunofluorescence+, in both fetuses Fetus A EM+ in lung	Placenta PCR+, EM+, immunofluorescence+ in both placentas	13 weeks gestational age Diamniotic twin pregnancy, both found with no heartbeat at 13 weeks Fetus A was 12 cm in length and 37 g Fetus B severely macerated
Vivanti A, 2021	Age 27 years, G2P1 NP PCR+ at 31 weeks	IUFD at 32 weeks	Not reported	Fetal tissue PCR+	Placental tissue PCR+ NP PCR+ at <48 h	32 weeks gestational age 2248 g birthweight
Zaigham M, 2022 (baby 1)	Age 31 years, G2P1 NP PCR+ at 34 weeks	IUFD at 35 weeks	Not reported	Fetal tissue PCR+	Placental tissue PCR+ NP swab PCR+	35 weeks gestational age 2200 g birthweight
Zaigham M, 2022 (baby 2)	Age 25 years, G1P0 NP PCR+ at 33 weeks	IUFD at 34 weeks	Not reported	Fetal tissue PCR+	Placental tissue PCR+	34 weeks gestational age 2190 g birthweight

Mother-to-child transmission was according to World Health Organization classification. NP RT-PCR=nasopharyngeal reverse transcriptase polymerase chain reaction; NICU=neonatal intensive care unit; IHC=immunohistochemistry; ISH=in situ hybridisation; GxPx= Gravida number, parity number; IUFD=intra-uterine fetal death; Dx=day number; EM= electron microscopy.

* Classified as “confirmed” in utero as tests were repeated <24 h, despite not meeting WHO criteria of positive test result at 24-48 h (appendix 3).

### Outcomes of SARS-CoV-2 positive babies

Outcomes were reported for 1213 SARS-CoV-2 positive babies 378 studies including cohort, case series, case reports); 1104 babies were alive at the end of follow-up, nine early pregnancy losses, 64 stillbirths, and 36 neonatal deaths occurred (table 3). Of the twenty babies with confirmed in utero infection, nine were alive at end of follow-up, one died after delivery, and eight were stillborn, and early pregnancy loss occurred in a set of twin fetuses. All three babies with confirmed intrapartum infection and the nine with early postnatal infection were alive at the end of follow-up (table 1 22 23 24 25 26 27 28 29 30 31 32 33 34 35 36 37 38 39 40). Eighty eight babies with symptoms (88/208) were born preterm, and gestational age was not known in another 39 babies. Of the 147 SARS-CoV-2 positive babies in whom radiological findings were reported (as defined by the authors), abnormalities suggestive of covid 19 related pneumonia were seen in 87, including 41 preterm babies (see supplementary appendix 8).

**Table 3:** Outcomes in SARS-CoV-2 positive babies born to mothers with covid-19 in all studies by severity of maternal disease (cohort, case series, and case reports). Values are numbers (percentages) unless stated otherwise

Offspring outcome	Term babies with a positive test result (≥ 37 weeks)					Preterm babies and early pregnancy with a positive test result (<37 weeks)					Babies with a positive test result (gestation not known)					All babies with a positive test result			
	Mild (n=185)	Severe (n=10)	Severity not known (n=84)	Total (n=279)		Mild (n=154)	Severe (n=26)	Severity not known (n=60)	Total (n=240)		Mild (n=112)	Severe (n=33)	Severity not known (n=903)	Total (n=1048)		Mil (n=451)	Severe (n=69)	Severity not known (n=1047)	Total (n=1567)
Alive at end of follow-up	173 (69)	10 (4)	67 (27)	250		96 (62)	21 (13)	39 (25)	156		90 (13)	1 (0)	607 (87)	698		359 (32)	32 (3)	713 (65)	1104
Miscarriage or abortion	—	—	—	0		9 (100)	—	—	9		—	—	—	0		9 (100)	—	—	9
Stillbirth	1 (50)	—	1 (50)	2		33 (80)	2 (5)	6 (15)	41		9 (43)	1 (5)	11 (52)	21		43 (67)	3 (5)	18 (28)	64
Neonatal death	5 (100)	—	—	5		11 (55)	2 (10)	7 (35)	20		2 (18)	—	9 (82)	11		18 (50)	2 (6)	16 (44)	36
Not known	6 (27)	—	16 (73)	22		5 (36)	1 (7)	8 (57)	14		11 (3)	31 (10)	276 (87)	318		22 (6)	32 (9)	300 (85)	354

* Either one of severe symptoms of covid-19, admission to intensive care unit, or maternal death.

### Maternal and perinatal factors associated with SARS-CoV-2 positivity in offspring

We found a significant association between maternal factors such as severe covid-19 (odds ratio 3.53, 95% confidence interval 1.54 to 8.10, I^2^=69%; 20 studies, 5545 women), maternal death (14.09, 4.14 to 47.97, I^2^=0%; 7 studies, 725 women), postnatal diagnosis of SARS-CoV-2 infection in the mother (4.99, 1.24 to 20.13, I^2^=65%; 12 studies, 750 women), caesarean section (1.36; 1.05 to 1.77, I^2^=26%; 58 studies, 11 139 women), preterm birth (1.47; 1.16 to 1.85, I^2^=0%; 48 studies, 9148 women), and SARS-CoV-2 positive status in the babies (table 4). Postnatal care, such as skin-to-skin contact was associated with a reduction in SARS-CoV-2 positive status in the babies (0.42; 0.25 to 0.70, I^2^=0%; 3 studies, 1101 women) (table 4). No associations were seen between SARS-CoV-2 positivity in babies and maternal admission to the intensive care unit, the trimester of maternal infection (third versus first or second trimester), breastfeeding or mother-baby separation at birth. (table 4). Subgroup analysis by variants of concern showed most studies were recruited before predominance of any variants of concern (supplementary appendix 9).

**Table 4:** Maternal and perinatal factors associated with SARS-CoV-2 positive test results in offspring

Risk factors	No of studies	No of mother-baby dyads	No of test positive babies*/No with risk factors	No of test positive babies*/No without risk factors	Odds ratio (95% CI)	I^2^ (%)
**Maternal factors**						
Severe covid-19	20	5545	68/758	222/4787	3.53 (1.54 to 8.10)	69
Maternal death	7	725	6/15	28 710	14.09 (4.14 to 47.97)	0
Admission to ICU	18	3349	12/135	325/3214	2.11 (0.93 to 4.79)	44
**Timing of maternal infection**						
Postnatal *v* antenatal	12	750	19/122	54/628	4.99 (1.24 to 20.13)	65
3^rd^ *v* 1^st^ or 2^nd^ trimester	9	4780	177/4392	8/388	0.64 (0.20 to 2.08)	45
**Intrapartum factors**						
Preterm *v* term	48	9148	119/1454	431/7694	1.47 (1.16 to 1.85)	0
Caesarean section *v* vaginal birth	58	11 139	331/5005	243/6134	1.36 (1.05 to 1.77)	2
**Postnatal care **						
Not separated at birth *v* separated	11	2051	72/894	73/1157	1.48 (0.68 to 3.19)	58
Breastfed *v* not breastfed	19	2618	164/1733	69/885	0.78 (0.43 to 1.43)	44
Skin-to-skin *v* not skin-to-skin	3	1101	24/546	61/555	0.42 (0.25 to 0.70)	0

ICU=intensive care unit; CI=confidence interval.

* Reverse transcriptase polymerase chain reaction.

### SARS-CoV-2 positivity in maternal and perinatal biological samples

In addition to testing infants for SARS-CoV-2, evidence was found for SARS-CoV-2 positivity in other maternal and perinatal biological samples tested in cohort studies: from placental tissue in 99 women (957 tested, 16 studies), placental RT-PCR swabs in four women (259 tested, 15 studies), amniotic fluid in 11 women (780 tested, 26 studies), vaginal fluid in nine women (564 tested,16 studies), and breast milk in 11 women (774 tested, 23 studies) (see supplementary appendix 10). Data were inadequate to assess the SARS-CoV-2 positivity status in newborn babies of women with positive placental, amniotic fluid, or other biological samples. When studies of all designs were included, RT-PCR positivity was found in 171 placental samples (n=1293 tested), 25 amniotic fluid samples (n=826), 12 maternal vaginal fluid samples (n=581), 23 babies’ stool specimens (n=503), and fifteen breast milk samples (n=818).

## Discussion

This update of our living systematic review includes 171 more primary studies (15 600 women, 12 585 babies) than our original review, and 50% more pregnant women with SARS-CoV-2 infection. We found that less than 3% of babies born to mothers seeking hospital care for any reason and with a diagnosis of SARS-CoV-2 infection also tested positive for SARS-CoV-2; the rates were lower (2%) when limited to babies of mothers with antenatal SARS-CoV-2 infection. Confirmed mother-to-child-transmission was noted through in utero, intrapartum, and early postnatal exposure; but the overall risk is low. In addition to severity of maternal covid-19 disease and postnatal maternal infection, which were found to be associated with offspring SARS-CoV-2 positivity in our original review, low gestation at birth and caesarean section were also associated with offspring SARS-CoV-2 positivity in in this update. The risk of a positive test result from offspring was reduced in mothers with skin-to-skin contact compared with no contact, which is usually offered only to mothers who are clinically stable and less likely to have severe covid-19. We did not find any association between SARS-CoV-2 positivity in offspring and trimester of maternal infection, breastfeeding, or mother-baby separation at birth. However, the number of studies reporting on early SARS-CoV-2 exposure were low. SARS-CoV-2 RNA was detected in amniotic fluid, placenta, vaginal fluid, and breast milk, but detection of virus in these biological specimens may not necessarily indicate infection in the baby. Few studies included mothers infected after the emergence of identified SARS-CoV-2 variants. 

### Strengths and limitations of this review

We carried out a comprehensive review on SARS-CoV-2 positivity rates in babies born to mothers with the infection and assessed the timing of exposure and likelihood of infection. We only included cohort studies for estimating the rates of SARS-CoV-2 positivity in offspring, unlike some of the published systematic reviews, which combined cohort and case-control studies with case series^47^ ^48^ ^49^ or reported positivity in babies as neonatal infection.^49^ ^50^ Our extensive sensitivity and subgroup analyses enabled us to assess the robustness of our findings according to the timing of maternal infection and testing in babies, across regions, and included SARS-CoV-2 variants as they emerged. We used the WHO classification system to ascertain the timing of transmission of SARS-CoV-2 from mother to baby and to confirm infection status, and we included data from any study that reported on babies with a positive test result. We were also able to consult with experts on the revision of the WHO classification to confirm our updates. We assessed the various maternal and perinatal factors that may be associated with SARS-CoV-2 positivity in babies. Our extensive de-duplication process minimised the risk of double counting data. This update has allowed us to seamlessly incorporate new evidence, as well as consider the evolving nature of the pandemic such as the impact of SARS-CoV-2 variants on offspring positivity. 

Our review has limitations. We aimed to rapidly update findings since our first review. As a result of the very large number of published studies, requiring intense effort to remove duplicate studies and participants, as well as the need to contact authors for information to classify positive babies, more time was required to prepare and analyse the data than anticipated. Many published primary studies have not kept pace with the evolving nature of the pandemic, resulting in very few studies reporting on women who were infected after the emergence of SARS-CoV-2 variants of concern. Our findings were also limited by heterogeneity in populations, tests, and outcomes. For example, mild and severe covid-19 were variably defined in the studies. Severe disease included severe symptoms, admission to an intensive care unit, and need for extracorporeal membrane oxygenation, and mild symptoms included asymptomatic women. Since almost all women in the studies had a recent diagnosis of SARS-CoV-2 infection, our findings are not applicable to those with infection in early pregnancy who recovered. Similarly, the types and timing of tests used in mothers and babies and their accuracy varied. Several studies did not provide details on the timing of perinatal exposure to SARS-CoV-2, or on the timing of tests, which hindered our ability to determine the timing of mother-to-child transmission of SARS-CoV-2. Even when the babies were tested, confirmatory tests were often not performed, further limiting our ability to use the WHO classification system to confirm infection status. Clinical outcomes of the babies born to mothers with SARS-CoV-2 infection were inconsistently reported, making it challenging to ascertain if the complications including stillbirths and neonatal deaths were related to SARS-CoV-2, other clinical factors or subject to publication bias. We were unable to consider the impact of vaccination status of pregnant women on our findings because of the paucity of available information in studies. Similarly, our subgroup analysis by predominant SARS-CoV-2 variant was limited to analysis of positivity rates and risk factors, and not to timing of mother to child transmission because of the paucity of data. Furthermore, we were unable to limit our analysis for association between postnatal care and offspring SARS-CoV-2 positivity to women with peripartum or postnatal infection because of poor reporting in studies, which could dilute any association between these risk factors and offspring SARS-CoV-2 positivity.

### SARS-CoV-2 positivity in offspring and timing of mother-to-child transmission

Our overall findings in this update have remained consistent with our original review. SARS-CoV-2 positivity rates in offspring remain low, and only a small proportion of those infants who had a positive result were from confirmed mother-to-child transmission. Some studies used anti-SARS-CoV-2 IgM antibody testing to diagnose neonatal infection. However, there are concerns about the accuracy of IgM antibody tests to diagnose vertical infection, and often a confirmatory IgM test was not performed.^15^ Compared with findings in our original review, we noted a smaller percentage of babies tested showed evidence of anti-SARS-CoV-2 specific IgM antibodies in this update, while babies tested in the first 24 h after birth had higher SARS-CoV-2 positivity rates. However, these rates remained below the overall positivity rates across studies. The low SARS-CoV-2 positivity rates in offspring in studies from Europe and North America could reflect the policy of universal maternal screening for SARS-CoV-2, resulting in inclusion of women with mild disease.^51^ ^52^ Since SARS-CoV-2 positivity in offspring was associated with severity of maternal disease, regions with mostly symptomatic testing of pregnant women were more likely to include women with severe disease, which may be reflected in the higher reported SARS-CoV-2 positivity rates in offspring in those regions. Subgroup analysis by SARS-CoV-2 virus variant was limited by the paucity of data reported. 

A previous systematic review that pooled data from all studies, including case series and reports without a formal meta-analysis, reported 70% of the 122 positive babies to have postpartum infections and 9% to have confirmed in utero and intrapartum infection, using the Shah classification.^47^ But we categorised fewer babies to have confirmed infection using the more stringent WHO criteria. We also refrained from providing the findings of confirmed infection as a proportion of all positive babies, because the selective reporting of SARS-CoV-2 positive babies in the studies affects the reliability of rate estimates.

The observed association between severe maternal disease and test ositivety in offspring could be linked to the prolonged detection of viral RNA in the mothers blood associated with disease severity.^53^ ^54^ But to date, no clear evidence links the severity of maternal disease to the shedding of SARS-CoV-2, although the duration of shedding appears to be prolonged in individuals with severe covid-19.^55^ The observed association between postnatal diagnosis of maternal SARS-CoV-2 and neonates who test positive could also be attributed to horizontal transmission from the mother, caregivers, or health workers, or from the neonate’s environment. Appropriate measures to reduce the risk of horizontal transmission should be followed if infection is suspected, such as improved ventilation, adequate personal protective equipment including protective masks and mask wearing, hand hygiene, and use of protective clothing during contact with the baby. 

In this update, we found an association between preterm birth and caesarean sections with SARS-CoV-2 positivity in offspring. This likely reflects severity of the disease in the mother or fetal distress, resulting in interventional approaches such as iatrogenic preterm delivery (as opposed to spontaneous preterm delivery) or caesarean section by healthcare givers. Other studies have shown that SARS-CoV-2 infection during pregnancy is associated with preterm birth and caesarean section.^56^ Our review also identified a reduction in risk of test positivity in offspring of mothers with a recent diagnosis of SARS-CoV-2 infection and who were allowed skin-to-skin contact with their neonates. Although few studies reported on skin-to-skin contact, and there was paucity of information within studies on infection control practices followed by mothers to reduce risk of horizontal transmission, it is reassuring to see that there is no increased risk of test positivity in offspring of mothers allowed skin-to-skin contact. It is also likely that only mothers with milder illness are allowed skin-to-skin contact with their babies, which is itself an indication of severity of SARS-CoV-2 infection. 

We did not find any association between breastfeeding practice in mothers with a recent diagnosis of SARS-CoV-2 infection and SARS-CoV-2 positivity in neonates, consistent with rare findings of RT-PCR positivity in breast milk samples.^57^ Although we found evidence of SARS-CoV-2 positivity in various biological samples that could be associated with the potential for vertical infection—such as amniotic fluid, placenta, and vaginal secretions, finding a pathogen in such samples does not necessarily correlate with infection of the fetus.^12^ ^13^ ^14^ Studies did not always report whether the maternal or fetal side of the placenta was swabbed, making it difficult to accurately determine placental infection.

### Relevance for clinical practice and research

Our review provides estimates on the burden of SARS-CoV-2 positive test results in exposed babies in clinical practice who will require further testing and monitoring. Evidence confirms vertical transmission of SARS-CoV-2 through in utero and intrapartum routes, although the absolute number of confirmed cases is low. SARS-CoV-2 positivity in babies is higher when their mothers have severe covid-19, and relevant testing should be considered in these babies. Very few studies reported on variants and therefore we had limited data to assess their impact on mother to child transmission and positivity rates. Current evidence does not support routine caesarean sections, mother-baby separation at birth, or formula feeding as interventions for avoiding SARS-CoV-2 transmission to babies in mothers with a recent diagnosis of SARS-CoV-2 infection.

Healthcare professionals need to perform further tests in fetuses and babies with a positive result to robustly confirm infection occurred and classify timing of mother-to-child transmission using appropriate samples according to WHO guidance. To reduce the proportion of babies in whom vertical transmission cannot be confirmed despite their initial positive status, repeat tests are needed at various time points in appropriate samples. Further research is needed to assess factors contributing to regional variations, such as different strategies for screening, emerging variants, and vaccination status. Further data are needed on the SARS-CoV-2 positive status of the various biological samples that could be potentially associated with SARS-CoV-2 mother-to-child transmission, and the relationship of sample positivity to fetal or neonatal infection. Future reviews will need to consider the changing landscape of the covid-19 pandemic, including the prevalence of covid-19 in various regions, impact of vaccination, and the effects of known and emerging SARS-CoV-2 variants on mother-to-child transmission. In the absence of individual participant data for synthesis, aggregate data review needs to capture these trends by reporting data according to time of participant recruitment. 

### Conclusion

The overall rates of SARS-CoV-2 positivity in babies born to mothers with SARS-CoV-2 infection is low. Evidence was found for confirmed vertical transmission of the virus, although the absolute numbers are low. Severe maternal covid-19 was associated with SARS-CoV-2 positivity in babies, but not vaginal birth, keeping the baby with the mother after birth, or breastfeeding.

**What is already known on this topic**

In pregnant women with SARS-CoV-2 infection, the virus and viral fragments have been detected in maternal blood, placenta, amniotic fluid, and breast milk, suggesting the potential for mother-to-child transmission
Primary studies and systematic reviews provide varied estimates for the rates of neonatal SARS-CoV-2 infection or positivity, or both
Current classification systems categorise the timing of SARS-CoV-2 mother-to-child transmission based on timing of exposure to the virus and type and timing of tests in offspring
People with severe covid-19 have high viral load

**What this study adds**

The overall rates of SARS-CoV-2 positivity in babies born to mothers with infection is low (<3%)
Evidence confirms mother-to-child transmission of SARS-CoV-2 through in utero, intrapartum, and early postpartum transmission, but vertical transmission is likely to be rare
Maternal factors such as severe covid-19, death, preterm birth, caesarean section, and postnatal infection, were associated with SARS-CoV-2 positivity in offspring
The increased risks with preterm birth and caesarean section could be attributed to iatrogenic interventions reflecting severe maternal covid-19
Breast feeding and keeping the baby with the mother after birth does not appear to increase the risk of SARS-CoV-2 positivity in the baby

## Data Availability

No additional data available.

## Web extra

Extra material supplied by authors

## Notes

Contributors: ST, MB, and JA conceptualised the study. TK, MY, JS, ML, OA, HK, MK, KB, SA, DS, AR, HL, MM, SM, AG, MH, AK, KA, and AB selected the studies. JA, TK, MY, SF-G, OA, HK, and MK extracted the data. JZ and AG-G conducted the analyses. JA and TK are joint first authors. All authors contributed to writing the manuscript and approved the final version. ST, JA, LM, and JZ are the guarantors. The corresponding author attests that all listed authors meet authorship criteria and that no others meeting the criteria have been omitted.

The lead authors (ST, JA, LM, and JZ) affirms that the manuscript is an honest, accurate, and transparent account of the study being reported; that no important aspects of the study have been omitted; and that any discrepancies from the study as planned (and, if relevant, registered) have been explained.

Dissemination to participants and related patient and public communities: The PregCOV-19 Living Systematic Review Consortium will disseminate the findings through a dedicated website (www.birmingham.ac.uk/research/who-collaborating-centre/pregcov/index.aspx), and through social media.

Provenance and peer review: Not commissioned; externally peer reviewed.
