# Supplementary material for: SARS-CoV-2 positivity in offspring and timing of mother-to-child transmission: living systematic review and meta-analysis
Source: BMJ. 2022 Mar 16;376:e067696. doi: 10.1136/bmj-2021-067696 (PMC8924705; doi:10.1136/bmj-2021-067696)
Supplement: Supplementary file 1 — Supplementary information: Additional material [file allj067696.ww.pdf]

PRISMA 2009 Checklist

Section/topic	#	Checklist item	Reported on page #
**TITLE**			
Title	1	Identify the report as a systematic review, meta-analysis, or both.	1
**ABSTRACT**			
Structured summary	2	Provide a structured summary including, as applicable: background; objectives; data sources; study eligibility criteria, participants, and interventions; study appraisal and synthesis methods; results; limitations; conclusions and implications of key findings; systematic review registration number.	4-5
**INTRODUCTION**			
Rationale	3	Describe the rationale for the review in the context of what is already known.	6
Objectives	4	Provide an explicit statement of questions being addressed with reference to participants, interventions, comparisons, outcomes, and study design (PICOS).	7
**METHODS**			
Protocol and registration	5	Indicate if a review protocol exists, if and where it can be accessed (e.g., Web address), and, if available, provide registration information including registration number.	7
Eligibility criteria	6	Specify study characteristics (e.g., PICOS, length of follow-up) and report characteristics (e.g., years considered, language, publication status) used as criteria for eligibility, giving rationale.	7-8
Information sources	7	Describe all information sources (e.g., databases with dates of coverage, contact with study authors to identify additional studies) in the search and date last searched.	7
Search	8	Present full electronic search strategy for at least one database, including any limits used, such that it could be repeated.	7
Study selection	9	State the process for selecting studies (i.e., screening, eligibility, included in systematic review, and, if applicable, included in the meta-analysis).	7-8
Data collection process	10	Describe method of data extraction from reports (e.g., piloted forms, independently, in duplicate) and any processes for obtaining and confirming data from investigators.	8-9
Data items	11	List and define all variables for which data were sought (e.g., PICOS, funding sources) and any assumptions and simplifications made.	9
Risk of bias in individual studies	12	Describe methods used for assessing risk of bias of individual studies (including specification of whether this was done at the study or outcome level), and how this information is to be used in any data synthesis.	8-9
Summary measures	13	State the principal summary measures (e.g., risk ratio, difference in means).	9-10
Synthesis of results	14	Describe the methods of handling data and combining results of studies, if done, including measures of consistency (e.g., I^2) for each meta-analysis.	9-10

PRISMA 2009 Checklist

Section/topic	#	Checklist item	Reported on page #
Risk of bias across studies	15	Specify any assessment of risk of bias that may affect the cumulative evidence (e.g., publication bias, selective reporting within studies).	10-11
Additional analyses	16	Describe methods of additional analyses (e.g., sensitivity or subgroup analyses, meta-regression), if done, indicating which were pre-specified.	10-11
**RESULTS**			
Study selection	17	Give numbers of studies screened, assessed for eligibility, and included in the review, with reasons for exclusions at each stage, ideally with a flow diagram.	11
Study characteristics	18	For each study, present characteristics for which data were extracted (e.g., study size, PICOS, follow-up period) and provide the citations.	11-12
Risk of bias within studies	19	Present data on risk of bias of each study and, if available, any outcome level assessment (see item 12).	12
Results of individual studies	20	For all outcomes considered (benefits or harms), present, for each study: (a) simple summary data for each intervention group (b) effect estimates and confidence intervals, ideally with a forest plot.	12-15
Synthesis of results	21	Present results of each meta-analysis done, including confidence intervals and measures of consistency.	12-14
Risk of bias across studies	22	Present results of any assessment of risk of bias across studies (see Item 15).	12-13
Additional analysis	23	Give results of additional analyses, if done (e.g., sensitivity or subgroup analyses, meta-regression [see Item 16]).	12-13
**DISCUSSION**			
Summary of evidence	24	Summarize the main findings including the strength of evidence for each main outcome; consider their relevance to key groups (e.g., healthcare providers, users, and policy makers).	15
Limitations	25	Discuss limitations at study and outcome level (e.g., risk of bias), and at review-level (e.g., incomplete retrieval of identified research, reporting bias).	16
Conclusions	26	Provide a general interpretation of the results in the context of other evidence, and implications for future research.	17-19
**FUNDING**			
Funding	27	Describe sources of funding for the systematic review and other support (e.g., supply of data); role of funders for the systematic review.	20-21

From: Moher D, Liberati A, Tetzlaff J, Altman DG, The PRISMA Group (2009). Preferred Reporting Items for Systematic Reviews and Meta-Analyses: The PRISMA Statement. PLoS Med 6(7): e1000097. doi:10.1371/journal.pmed1000097

For more information, visit: **www.prisma-statement.org**.

Appendix: Details of search strategies used to include studies in the living systematic review on COVID-19 in pregnant and recently pregnant women

1. Cochrane Gynaecology and Fertility

Pubmed

Item	Term
1	pregnancy/
2	pregnan*.tw.
3	neonatal.tw.
4	perinatal.tw.
5	mothers/.
6	mother.tw.
7	maternal.tw.
8	obstetric.tw.
9	infant, newborn/
10	infant.tw.
11	newborn.tw.
12	child*.tw.
13	or/1-12
14	COVID-19.tw.
15	COVID-2019.tw.
16	severe acute respiratory syndrome coronavirus 2.tw.
17	2019-nCoV.tw.
18	SARS-CoV-2.tw.
19	2019nCoV.tw
20	or/14-19
21	coronavirus.tw.
22	2019/12.pd
23	2020.pd.
24	or/22-23
25	21 and 24
24	or/20-25
25	13 and 24

Google Scholar and Google
Using the following text words (pregnancy OR neonatal OR perinatal OR maternal OR obstetric OR newborn) AND (COVID-19 or SARS-Cov-2)

2. EPPI Centre

The MEDLINE search strategy is the OVID Expert Search as developed by Wolters Kluwer and available at http://tools.ovid.com/coronavirus/

MEDLINE search strategy

1 exp Coronavirus/
2 exp Coronavirus Infections/
3 (coronavirus* or corona virus* or OC43 or NL63 or 229E or HKU1 or HCoV* or ncov* or covid* or sars-cov* or sarscov* or Sars-coronavirus* or Severe Acute Respiratory Syndrome Coronavirus*).mp.
4 (or/1-3) and ((20191* or 202*).dp. or 20190101:20301231.(ep).)
5 4 not (SARS or SARS-CoV or MERS or MERS-CoV or Middle East respiratory syndrome or camel* or dromedar* or equine or coronary or coronal or covidence* or covidien or influenza virus or HIV or bovine or calves or TGEV or feline or porcine or BCoV or PED or PEDV or PDCoV or FIPV or FCoV or SADS-CoV or canine or CCov or zoonotic or avian influenza or H1N1 or H5N1 or H5N6 or IBV or murine corona*).mp.
6 ((pneumonia or covid* or coronavirus* or corona virus* or ncov* or 2019-ncov or sars*).mp. or exp pneumonia/) and Wuhan.mp.
7 (2019-ncov or ncov19 or ncov-19 or 2019-novel CoV or sars-cov2 or sars-cov-2 or sarscov2 or sarscov-2 or Sars-coronavirus2 or Sars-coronavirus-2 or SARS-like coronavirus* or coronavirus-19 or covid19 or covid-19 or covid 2019 or ((novel or new or nouveau) adj2 (CoV on nCoV or covid or coronavirus* or corona virus or Pandemi*2)) or ((covid or covid19 or covid-19) and pandemic*2) or (coronavirus* and pneumonia)).mp.
8 COVID-19.rx,px,ox. or severe acute respiratory syndrome coronavirus 2.os.
9 ("32240632" or "32236488" or "32268021" or "32267941" or "32169616" or "32267649" or "32267499" or "32267344" or "32248853" or "32246156" or "32243118" or "32240583" or "32237674" or "32234725" or "32173381" or "32227595" or "32185863" or "32221979" or "32213260" or "32205350" or "32202721" or "32197097" or "32196032" or "32188729" or "32176889" or "32088947" or "32277065" or "32273472" or "32273444" or "32145185" or "31917786" or "32267384" or "32265186" or "32253187" or "32265567" or "32231286" or "32105468" or "32179788" or "32152361" or "32152148" or "32140676" or "32053580" or "32029604" or "32127714" or "32047315" or "32020111" or "32267950" or "32249952" or "32172715").ui.
10 or/6-9
11 5 or 10

The Embase search strategy as at 21st April 2020

1 exp Coronavirus Infections/
2 exp coronavirinae/
3 (coronavirus* or corona virus* or OC43 or NL63 or 229E or HKU1 or HCoV* or ncov* or covid* or sars-cov* or sarscov* or Sars-coronavirus* or Severe Acute Respiratory Syndrome Coronavirus*).mp.
4 or/1-3
5 4 not (SARS or SARS-CoV or MERS or MERS-CoV or Middle East respiratory syndrome or camel* or dromedar* or equine or coronary or coronal or covidence* or covidien or

influenza virus or HIV or bovine or calves or TGEV or feline or porcine or BCoV or PED or PEDV or PDCoV or FIPV or FCoV or SADS-CoV or canine or CCov or zoonotic or avian influenza or H1N1 or H5N1 or H5N6 or IBV or murine corona*).mp.
6 ((pneumonia or covid* or coronavirus* or corona virus* or ncov* or 2019-ncov or sars*).mp. or exp pneumonia/) and Wuhan.mp.
7 (2019-ncov or ncov19 or ncov-19 or 2019-novel CoV or sars-cov2 or sars-cov-2 or sarscov2 or sarscov-2 or Sars-coronavirus2 or Sars-coronavirus-2 or SARS-like coronavirus* or coronavirus-19 or covid19 or covid-19 or covid 2019 or ((novel or new or nouveau) adj2 (CoV on nCoV or covid or coronavirus* or corona virus or Pandemi*2)) or ((covid or covid19 or covid-19) and pandemic*2) or (coronavirus* and pneumonia)).mp.
8 6 or 7
9 5 or 8

3. WHO COVID-19 database

The WHO COVID-19 database contained articles on the novel coronavirus from the following sources:

- Web of Science
- Oxford Academic Journals
- Pubmed NIH
- Ishiyaku
- J Stage
- Cinii articles
- Ichushi Web – JAMAS
- Science Direct
- Wiley Online Journals
- JAMA Network
- British Medical Journal
- Mary Ann Liebert
- New England Journal of Medicine
- Sage Publications
- Taylor and Francis Online
- Springer Link
- Biomed Central
- MDPI
- ASM
- PLOS
- The Lancet
- Cell Press
- Cell Press Search Interface
- EMBASE
- KoreaMed

- Global Index Medics
- MMWR
- Epidemiology and Health
- American Chemical Society
- Eurosurvellance
- Cambridge Press
- LWW
- Airiti
- JIMR
- Emerging Infectious Diseases
- Osong Public Health & Research Perspectives
- BASE Bielefeld
- LitCOVID

An additional step using the following search terms was added to the WHO search from 12th May 2020

tw:(newborn* OR mother* OR bab* OR wom* OR pregnan* OR postpart* OR neonat* OR fetus OR fetal OR newborn OR mother OR bab*)

Appendix 3. Summary of time of sample collection, type of samples and test for categorization of timing of vertical transmission for live-born infants (a) and fetal demise (b) in women with documented SARS-CoV-2 infection

(a)

Timing of transmission and categories	Early *in utero* exposure testing							PLUS	Later exposure testing									
**Time of sample collection**	Birth to age <24 hours								24-48 hr	≥48 hr	24-48 hr	≥48 hr	Repeated within 10 d	24 hr-7 d	7-14 d	Repeated within 10 d	>14 d	Repeated within 10 d
**Type of sample and test**	Sterile sample			Non-sterile sample			Serology (IgM/ IgA)		Sterile[4] sample		Non-sterile[5] sample			Serology (IgM/IgA)				
	Neonatal blood	Amniotic fluid	Lower respiratory tract[1]/CSF	Placenta RT-PCR /ISH	Placenta IHC/ microscopy	Upper respiratory tract[2]/other[3]												
***In Utero* (live birth)** (maternal infection anytime during pregnancy)																		
Confirmed	+	+	+	+		+	+		+									
Possible	+	+	+	+	+	+	+				+			+				
Unlikely	+	+	+	+	+	+	+		NEG		NEG			NEG				
	NEG	NEG	NEG	NEG	NEG	NEG	NEG		+		+			+				
Indeterminate	+	+	+	+	+	+	+		ND		ND			ND				
	ND	ND	ND	ND	ND	ND	ND				+			+				
**Intrapartum** (maternal infection near the time of birth)																		
Confirmed	NEG	NEG	NEG	NEG	NEG	NEG	NEG		+		+	+ to 7 d			+	+		
Possible	ND	ND	ND	ND	ND	ND	ND		+		+	+ to 7 d			+	+		
Unlikely	ND	ND	ND	ND	ND	ND	ND		+	NEG	+	NEG			+	NEG		
**Early Postnatal** (maternal infection near the time of birth)																		
Confirmed	NEG	NEG	NEG	NEG	NEG	NEG	NEG		NEG	+	NEG	+	+	NEG	NEG		+	+
Possible	ND	ND	ND	ND	ND	ND	ND		ND	+	ND	+	+	ND	ND		+	+
Unlikely	ND	ND	ND	ND	ND	ND	ND		ND		ND	+	NEG	ND	ND		+	NEG
Indeterminate	ND	ND	ND	ND	ND	ND	ND		ND		ND	+	ND	ND	ND		+	ND

CSF: cerebrospinal fluid; d: age in days; hr: age in hours; ISH: in situ hybridization; IHC: immunohistochemistry; ND: not done; RT-PCR: reverse transcription polymerase chain reaction; [1] Lower respiratory tract: bronchoscopic or non-bronchoscopic bronchoalveolar lavage, broncho- or tracheal aspirate; [2] Upper respiratory tract: nasopharyngeal or oropharyngeal swab or aspirate; [3] Other non-sterile site (e.g. stool); [4] Sterile sample: neonatal blood, cerebrospinal fluid, lower respiratory tract; [5] Non-sterile sample: upper respiratory tract or other non-sterile site; + = positive test (meets category if one or more tests positive for early exposure plus one or more tests positive for later exposure). NEG = negative test (meets category if all tests that were performed are negative, early or later exposure). Colour code: ***In utero* - Blue -**: dark blue: positive test, light blue: negative test; **Intrapartum - Orange**: dark orange positive test, light orange negative test; **Postnatal - Red**: dark red positive test, light red negative test; **Gray**: not done.

(b)

Category	Type of sample and test			
	Fetal tissue[1] RT-PCR or ISH	Fetal tissue IHC or microscopy or fetal swab RT-PCR	Amniotic fluid (sterile)	Placental tissue (RT-PCR, ISH, IHC or microscopy) or placental swab RT-PCR
**Confirmed**	+	+	+	+
	+	NEG or ND	+	+
	+	+	+	NEG or ND
	+	+	NEG or ND	+
	+	+	NEG or ND	NEG or ND
	+	NEG or ND	+	NEG or ND
	+	NEG or ND	NEG or ND	+
	+	NEG or ND	NEG or ND	NEG or ND
**Possible**	ND	+	+	+
	ND	NEG or ND	+	+
	ND	+	NEG or ND	+
	ND	+	+	NEG or ND
	ND	+	NEG or ND	NEG or ND
	ND	NEG or ND	+	NEG or ND
	ND	NEG or ND	NEG or ND	+
	NEG	NEG or ND	+	NEG or ND
**Unlikely**	NEG	+	NEG or ND	+
	NEG	+	NEG or ND	NEG or ND
	NEG	NEG or ND	NEG or ND	+

CS: caesarean section; IHC: immunohistochemistry; ISH: in situ hybridization; ND = not done; RT-PCR: reverse transcription polymerase chain reaction; ROM: rupture of membranes; + = positive test (meets category if one or more tests positive); NEG = negative test (meets category if all tests that were performed are negative); [1] Fetal tissues from sterile site such as fetal organ (e.g. lung, live, brain), testing of multiple specimens recommended; [2] Amniotic fluid from sterile collection prior to rupture of membranes.

Source: Definition and categorization of the timing of mother-to-child transmission of SARS-CoV-2. Scientific brief. 7 February 2021, COVID-19: Scientific briefs. © World Health Organization 2021. WHO reference number: WHO/2019-nCoV/mother-to-child_transmission/2021.1

Appendix 4 Characteristics of cohort studies included in the systematic review: SARS-CoV-2 positivity in babies born to mothers with coronavirus disease (COVID-19) and timing of mother-to-child transmission

Study		Population				Samples taken and tests performed				Outcomes	
						RT-PCR					
Author, Year	Study Design	No. of mothers	No. of foetuses/neonates	Inclusion and exclusion criteria	Risk factors evaluated	Maternal specimens	Foetal/neonatal specimens	Neonatal IgM (peripheral or cord blood)	Others	WHO classification on timing of mother-to-child transmission	Maternal or foetal/neonatal death
**East Asia and Pacific**											
**Arakaki T, 2021 Japan**	Retrospective cohort	72 mothers with confirmed COVID-19 (RT-PCR or clinical and radiological diagnosis)	24 (11 underwent testing)	Pregnant and postpartum women with COVID-19 and their neonates in Japan between 16 January and 30 June 2020	-	-	Pharyngeal swab at any time	-	-	-	1 maternal death<br>3 foetal deaths (abortion)
**Cheng B, 2020 China***	Retrospective cohort	31 mothers with confirmed COVID-19 (RT-PCR)	31 (17 underwent testing)	Pregnant women with COVID-19 and their newborns admitted between January 5 to February 23, 2020	-	-	Pharyngeal swab at birth<br>Pharyngeal swab at any time	-	-	In Utero: Unlikely<br>N = 2	-
**Clemente MJ, 2021 Philippines**	Ambispective cohort	197 mothers with confirmed COVID-19 (RT-PCR)	192	Pregnant women with COVID-19 and their neonates between April to August 2020	Preterm birth	Amniotic fluid<br>Placenta tissue (any side)<br>Breast milk	Pharyngeal swab at birth<br>Pharyngeal swab at any time<br>Cord tissue	-	Anal swab	In Utero: Indeterminate<br>N=2	5 foetal deaths (3 abortions)<br>2 neonatal deaths<br>4 maternal deaths
**Gao J, 2020 China***	Retrospective cohort	24 mothers with confirmed COVID-19 (RT-PCR)	24 (8 underwent testing after 1 month so were excluded)	Mothers with COVID-19 and their neonates admitted between January 19 and April 12, 2020	Preterm birth	Amniotic fluid<br>Placenta (any side)	Pharyngeal swab at any time<br>Stool<br>Cord blood<br>Gastric juice<br>Urine	Cord blood IgM<br>Neonatal blood IgM	Cord blood IgG<br>Neonatal blood IgG<br>Maternal IgM and IgG	In Utero: Possible<br>N = 3<br>Not classifiable<br>N = 4	-
**Gao X, 2020 China**	Retrospective cohort	14 mothers with confirmed COVID-19 (RT-PCR)	14 (12 underwent testing)	Mothers with COVID-19 and their neonates between January 9 and February 27 2020	Mode of delivery	Vaginal fluid<br>Breast milk	Pharyngeal swab at any time<br>Stool	Neonatal blood IgM	Cord blood IgG<br>Neonatal blood IgG	-	-

**Huang W, 2020 China**	Retrospective cohort	8 mothers with confirmed COVID-19 (RT-PCR)	9 (6 underwent testing)	Pregnant women with COVID-19 and their neonates between January 24 to February 19, 2020	-	-	Pharyngeal swab at any time	-	-	-	No maternal death<br>1 foetal death<br>2 neonatal deaths
**Hu X (1), 2020 China***	Retrospective cohort	42 mothers with confirmed COVID-19 (RT-PCR)	42	Neonates born to mothers with COVID-19 admitted between January 23 and March 19, 2020	-	Amniotic fluid<br>Breast milk	Pharyngeal swab at any time<br>Stool<br>Cord blood	Neonatal blood IgM	Neonatal blood IgG	-	-
**Jang WK, 2021 South Korea**	Cohort	7 mothers with confirmed COVID-19 (RT-PCR)				Amniotic fluid<br>Placenta (any side)	Pharyngeal swab at any time<br>Cord blood PCR	Cord blood IgM	Cord blood IgG	-	
**Khan S (1), 2020***	Retrospective cohort	17 mothers with confirmed COVID-19 (RT-PCR)	17	Pregnant women with COVID-19 and their neonates between January 25 to February 15, 2020	-	-	Pharyngeal swab at birth	-	-	-	-
**Liu P, 2020 China***	Retrospective cohort	51 mothers: 7 with confirmed COVID-19 (RT-PCR) and 44 with possible COVID-19 (clinical diagnosis)	51	All neonates born to mothers with COVID-19 admitted from January 20 to March 3, 2020	Mode of delivery, pregnancy status, trimester of infection, ICU admission, maternal severity, preterm birth	-	Pharyngeal swab at birth<br>Blood	Neonatal blood IgM	Neonatal blood IgG	In Utero: Possible N = 1<br>In Utero: Unlikely N = 2<br>Not classifiable N = 2	-
**Luo Q, 2020 China***	Retrospective cohort	23 mothers: 14 with confirmed COVID-19 (RT-PCR), 9 with possible COVID-19 (radiological or clinical diagnosis)	23 (21 underwent testing)	All pregnant women with SARS-CoV-2 infection during the third trimester, and their neonates between February 1 to March 15, 2020	-	Breast milk	Pharyngeal swab at birth<br>Pharyngeal swab at any time	-	-	-	-

**Mattar CNZ, 2020 Singapore**	Prospective cohort	16 mothers with confirmed COVID-19 (RT-PCR)	6	All pregnant women with diagnosed COVID-19 admitted between 15 March and 22 August 2020, and their neonates	-	Amniotic fluid<br>Placenta (any side)<br>Placenta (baby side)<br>Vaginal fluid<br>Stool<br>Breast milk	Pharyngeal swab at any time<br>Cord blood	-	-	-	No maternal deaths<br>2 foetal deaths (miscarriag e)
**Morioka I, 2021 Japan**	Cohort	52 mothers with confirmed COVID-19 (RT-PCR)	52	Pregnant women with COVID-19 and their neonates captured in a survey conducted by the Japan Pediatric Society between September 1 to October 8, 2020	-	-	Pharyngeal swab at any time	-	-	Not classifiable N = 1	-
**Nie R, 2020 China**	Retrospecti ve cohort	33 mothers with confirmed COVID-19 (RT-PCR)	28 (26 underwent testing)	Pregnant women with COVID-19 infection and their neonates admitted to between January 1 and February 20 2020	Mode of delivery	Placenta (any side)	Pharyngeal swab at any time	-	-	-	No maternal deaths<br>1 foetal death (abortion)
**Poon L, 2021 China**	Prospective cohort	20 mothers with confirmed COVID-19 (RT-PCR)	20	Pregnant women with confirmed COVID-19 infection and their neonates from March 27 2020 to January 24 2021	-	Blood IgG	Pharyngeal swab at any time	Cord blood IgM	Cord blood IgG	-	-
**Suyuthi FP, 2021 Indonesia**	Cohort	26 mothers with confirmed COVID-19 (RT-PCR)	24	Pregnant women with COVID-19 and their neonates between 1 April and 12 July 2020	-	-	Pharyngeal swab at any time	-	-	Not classifiable N = 5	-
**Tang F, 2020 China***	Cohort	20 mothers: 8 with confirmed COVID-19 (RT-PCR), 5 with probable COVID-19 (serology) and 7 with possible COVID-19	20	Neonates born to mothers with COVID-19 admitted between 2 February to March 31, 2020	Mode of delivery	Amniotic fluid	Pharyngeal swab at any time<br>Cord blood	Neonatal blood IgM	Maternal Blood IgM and IgG<br>Neonatal blood IgG	In Utero: Possible N = 3<br>In Utero: Unlikely N = 3<br>Not classifiable N = 3	-

		(clinical or radiological diagnosis)									
**Wiyati PS, 2021 Indonesia**	Cohort	45 mothers with confirmed COVID-19 (RT-PCR)	46	Pregnant women with COVID-19 and their neonates between March 2020 and January 2021	-	-	Pharyngeal swab at any time	-	-	Not classifiable N = 5	3 maternal deaths 4 intrauterine deaths 1 stillbirth 1 neonatal death
**Wu H, 2021 China**	Cohort	11 mothers: 3 with confirmed COVID-19 (RT-PCR) and 8 with probable COVID-19 (serology)	11 (9 underwent testing)	Pregnant women who recovered from COVID-19, and their neonates between March 30 and April 24 2020	Maternal severity	Amniotic fluid Placenta (any side)	Pharyngeal swab at any time	Blood IgM Cord blood IgM	Placenta RT-PCR and IHC Blood IgG Cord blood IgG	In Utero: Indeterminate N = 5	-
**Wu YT, 2020 China***	Retrospective cohort	29 mothers: 13 with confirmed COVID-19 (RT-PCR) and 16 with possible COVID-19 (radiological diagnosis)	30 (18 underwent testing)	Pregnant women with COVID-19 and their neonates between January 30 and March 10, 2020	Mode of delivery, preterm birth	-	Pharyngeal swab at any time	Neonatal blood IgM	Neonatal blood IgG	Postpartum: Unlikely N = 2 Postpartum: Indeterminate N = 1	-
**Xu S (1), 2020 China***	Retrospective cohort	34 mothers with confirmed COVID-19 (RT-PCR)	23	Pregnant women with COVID-19 admitted between January 15 and March 15, 2020, and their neonates<br><br>Controls: non-pregnant women with COVID-19	-	-	Pharyngeal swab at any time	-	-	-	-
**Yang H (2), 2020 China**	Retrospective cohort	27 mothers: 11 with confirmed COVID-19 (RT-PCR), 7 with probable COVID-19 (serology) and 8 with possible COVID-19 (clinical or	24 (23 underwent testing)	Pregnant women suspected with COVID-19 and their neonates from January 20 to March 19, 2020	Preterm birth, mode of delivery	-	Pharyngeal swab at birth	Neonatal blood IgM	Neonatal blood IgG	In Utero: Indeterminate N = 1	-

		radiological diagnosis)									
**Yang H (1), 2020 China**	Retrospective cohort	79 mothers: 16 with confirmed COVID-19 (RT-PCR), 3 with probable COVID-19 (serology), 60 with possible COVID-19 infection (clinical or radiological diagnosis)	82 (26 underwent testing)	Pregnant women with confirmed or suspected COVID-19 and their neonates from January 20 to April 15, 2020	-	-	Pharyngeal swab at any time	Blood IgM Cord blood IgM	Blood IgG Cord blood IgG	-	-
**Yu X, 2020 China***	Retrospective cohort	23 mothers with confirmed/possible COVID-19 (RT-PCR or clinical/radiological diagnosis)	24	Pregnant women admitted between February 6 and March 5 2020 and their neonates	-	Amniotic fluid<br>Placenta (any side)<br>Vaginal fluid<br>Stool<br>Breast milk	Pharyngeal swab at any birth<br>Pharyngeal swab at any time<br>Cord blood	-	-	In Utero: Indeterminate N = 3	-
**Zeng L, 2020 China***	Retrospective cohort	33 mothers with confirmed COVID-19 (RT-PCR)	33	Neonates born to mothers with COVID-19 from Wuhan's Children Hospital	Preterm birth, ICU admission, mode of delivery	Amniotic fluid	Pharyngeal swab at any time<br>Cord	-	-	Peripartum: Confirmed N = 2<br>Peripartum: Possible N = 1	-
**Anggraini NWP, 2020 Indonesia**	Case series	9 mothers: 3 with confirmed COVID-19 (RT-PCR), 6 with probable COVID-19 (serology)	8	-	-	-	Pharyngeal swab at any time	-	-	-	-
**Chen S (1), 2020 China***	Case series	3 mothers with confirmed COVID-19 (RT-PCR)	3	-	-	Placenta (any side)	Pharyngeal swab at any time	-	-	-	-
**Corebima B, 2020 Indonesia**	Case series	4 mothers with probable COVID-19 (serology)	4	-	-	-	Pharyngeal swab at any time	-	-	-	-

**Fan C, 2020 China***	Case series	2 mothers with confirmed COVID-19 (RT-PCR)	2	-	-	Amniotic fluid<br>Placenta (any side)<br>Vaginal fluid<br>Breast milk	Pharyngeal swab at birth<br>Cord blood	-	-	-	-
**Li S, 2020 China**	Case series	3 mothers with confirmed COVID-19 (RT-PCR)	2	-	-	-	Pharyngeal swab at birth	-	-	-	-
**Sun M, 2020 China**	Case series	3 mothers with confirmed COVID-19 (RT-PCR)	3	-	-	-	Pharyngeal swab at any time	-	-	Postpartum:<br>Indeterminate<br>N = 1	1 maternal death<br>No foetal/neonatal deaths
**Yu N (2), 2020 China**	Case series	2 mothers with confirmed COVID-19 (RT-PCR)	2 (0 underwent testing)	-	-	Amniotic fluid<br>Blood IgM and IgG	-	-	-	-	-
**Zhu C, 2020 China***	Case series	5 mothers with confirmed COVID-19 (RT-PCR)	5 (0 underwent testing)	-	-	Vaginal fluid<br>Breast milk	-	-	-	-	-
**Bae J, 2020 South Korea**	Case report	1 mother with confirmed COVID-19 (RT-PCR)	1	-	-	Amniotic fluid<br>Placenta (any side)	Pharyngeal swab at any time<br>Blood<br>Cord blood PCR	-	-	-	-
**Bai BL, 2020 China**	Case report	1 mother with confirmed COVID-19 (RT-PCR)	1	-	-	Amniotic fluid<br>Placenta (any side)<br>Breast milk	Pharyngeal swab at birth<br>Pharyngeal swab at any time<br>Blood<br>Cord blood	Blood IgM	-	-	-
**Basyir V, 2021 Indonesia**	Case report	1 mother with confirmed COVID-19 (RT-PCR)	1	-	-	-	Pharyngeal swab at any time	-	-	-	-
**Chen R (1), 2020 China**	Case report	1 mother with confirmed COVID-19 (RT-PCR)	1	-	-	-	Pharyngeal swab at any time	-	-	-	-
**Cui P, 2020 China**	Case report	1 mother with possible COVID-19	1	-	-	Vaginal fluid<br>Maternal stool<br>Breast milk	Pharyngeal swab at any time	-	-	-	-

		(clinical or radiological diagnosis)									
**Dong L, 2020 China***	Case report	1 mother with confirmed COVID-19 (RT-PCR)	1	-	-	Vaginal fluid<br>Breast milk	Pharyngeal swab at any time	Neonatal blood IgM	-	In Utero: Indeterminate<br>N = 1	-
**Du Y, 2020 China**	Case report	1 mother with confirmed COVID-19 (RT-PCR)	1	-	-	-	Pharyngeal swab at any time	-	-	-	-
**Gao J (1), 2020 China***	Case report	1 mother with confirmed COVID-19 (RT-PCR)	1	-	-	Amniotic fluid<br>Placenta (any side)	Pharyngeal swab at any time<br>Stool<br>Anal swab	Neonatal blood IgM	Neonatal blood IgG<br>Maternal blood IgM and IgG	In Utero: Possible<br>N = 1	-
**Gu ZL, 2020 China**	Case report	1 mother with possible COVID-19 (clinical or radiological diagnosis)	1	-	-	-	Pharyngeal swab at any time	-	-	-	-
**Han MS, 2020 South Korea**	Case report	1 mother with confirmed COVID-19 (RT-PCR)	1	-	-	Stool<br>Breast milk<br>Urine	Pharyngeal swab at any time<br>Blood	-	Neonatal stool	Not classifiable<br>N = 1	-
**Harahap A, 2021 Indonesia**	Case report	1 mother with confirmed COVID-19 (RT-PCR)	2	-	-	-	Pharyngeal swab at any time	-	-	-	-
**He SM, 2020 China**	Case report	1 mother with confirmed COVID-19 (RT-PCR)	1	-	-	-	Cord blood	-	-	-	No maternal death<br>1 neonatal death
**Huang J, 2020 China***	Case report	1 mother with confirmed COVID-19 (RT-PCR)	1	-	-	Amniotic fluid<br>Placenta (any side)<br>Maternal stool<br>Breast milk	Pharyngeal swab at any time<br>Neonatal stool<br>Cord blood	-	-	-	-
**Huang LQ, 2020 China**	Case report	1 mother with confirmed COVID-19 (RT-PCR)	1	-	-	-	Pharyngeal swab at any time	-	-	-	-

**Jung J, 2020 South Korea**	Case report	1 mother with confirmed COVID-19 (RT-PCR)	1	-	-	-	Pharyngeal swab at any time	-	-	-	-
**Khan S, 2020 China**	Case report	3 mothers with confirmed COVID-19 (RT-PCR)	3	-	-	-	Pharyngeal swab at birth	-	-	-	-
**Lee E 2020, South Korea**	Case report	1 mother with confirmed COVID-19 (RT-PCR)	1	-	-	Amniotic fluid<br>Placenta (any side)	Pharyngeal swab at any time<br>Blood PCR<br>Cord blood PCR	-	Neonatal stool<br>Gastric lavage fluid<br>Urine<br>Skin swab	-	-
**Li Y, 2020 China***	Case report	1 mother with confirmed COVID-19 (RT-PCR)	1	-	-	Amniotic fluid<br>Placenta (any side)<br>Maternal stool<br>Breast milk	Pharyngeal swab at birth<br>Neonatal stool<br>Blood<br>Cord blood	-	Placenta (RT-PCR)	-	-
**Lowe B, 2020 Australia**	Case report	1 mother with confirmed COVID-19 (RT-PCR)	1	-	-	-	Pharyngeal swab at any time	-	-	-	-
**Lu D, 2020 China**	Case report	1 mother with confirmed COVID-19 (RT-PCR)	1	-	-	-	Pharyngeal swab at birth<br>Pharyngeal swab at any time<br>Blood	-	-	-	-
**NG DCE, 2021 Malaysia**	Case report	1 mother with confirmed COVID-19 (RT-PCR)	1	-	-	-	Pharyngeal swab at any time	Blood IgM	Blood IgG	In Utero: Confirmed N=1	-
**Panichaya P, 2020 Thailand**	Case report	1 mother with confirmed COVID-19 (RT-PCR)	1	-	-	Placenta (any side)	Pharyngeal swab at birth	-	-	-	-
**Peng L 2021 China**	Case report	1 mother with confirmed COVID-19 (RT-PCR)	2	-	-	-	Pharyngeal swab at any time<br>Sputum	-	Neonatal stool	In Utero: Indeterminate N = 2	-
**Peng Z 2021 China**	Case report	1 mother with confirmed COVID-19 (RT-PCR)	1	-	-	Amniotic fluid<br>Placenta (any side)	Pharyngeal swab at birth<br>Blood	-	Neonatal stool	-	-

						Vaginal fluid<br>Maternal stool<br>Breast milk	Cord blood				
**Song L, 2020 China***	Case report	1 mother with probable COVID-19 (serology)	1	-	-	Maternal stool	Pharyngeal swab at any time	-	-	-	-
**Tang J, 2020 China**	Case report	2 mothers with confirmed COVID-19 (RT-PCR)	2	-	-	Blood IgM and IgG	Pharyngeal swab at birth<br>Pharyngeal swab at any time	Blood IgM	Blood IgG	-	
**Tong C, 2020 China**	Case report	1 mother with probable COVID-19 (serology)	1	-	-	-	Pharyngeal swab at any time	Blood IgM	Blood IgG	-	-
**Tran HT (1), 2021**	Case report	1 mother with confirmed COVID-19 (RT-PCR)	1	-	-	Amniotic fluid<br>Placenta (any side)<br>Breast milk	Pharyngeal swab at any time<br>Cord blood	-	-	-	-
**Walczak A, 2020 Australia**	Case report	1 mother with confirmed COVID-19 (RT-PCR)	1	-	-	Placenta (any side)<br>Breast milk<br>Blood IgM and IgG	Pharyngeal swab at any time	-	Neonatal stool	-	-
**Wang J, 2020 China**	Case report	1 mother with confirmed COVID-19 (RT-PCR)	1	-	-	-	Pharyngeal swab at any time	Neonatal blood IgM	Neonatal blood IgG<br>Neonatal stool	Not classifiable<br>N = 1	-
**Wang S, 2020 China**	Case report	1 mother with confirmed COVID-19 (RT-PCR)	1	-	-	Placenta (any side)<br>Breast milk	Pharyngeal swab at any time	-	Anal swab	In Utero: Unlikely<br>N = 1	-
**Wang X, 2020 China**	Case report	1 mother with confirmed COVID-19 (RT-PCR)	1	-	-	Amniotic fluid<br>Placenta (any side)	Pharyngeal swab at birth<br>Cord blood	-	Neonatal stool	-	-
**Xia H 2020, China**	Case report	1 mother with confirmed COVID-19 (RT-PCR)	1	-	-	-	Pharyngeal swab at any time	-	-	-	-
**Xiong X, 2020 China**	Case report	1 mother with confirmed COVID-19 (RT-PCR)	1	-	-	Amniotic fluid<br>Placenta (any side)	Pharyngeal swab at birth<br>Cord blood	Blood IgM	Blood IgG<br>Neonatal stool	-	-

						Vaginal fluid<br>Maternal stool<br>Breast milk					
**Yu ZY, 2020 China**	Case report	1 mother with confirmed COVID-19 (RT-PCR)	1	-	-	-	Pharyngeal swab at any time<br>Cord blood	-	Cord blood IgG	Postpartum: Confirmed<br>N = 1	-
**Zeng L (1), 2020 China**	Case report	1 mother with confirmed COVID-19 (RT-PCR)	1	-	-	-	Pharyngeal swab at any time	-	Anal swab	Not classifiable<br>N = 1	-
**Zhao YW, 2020 China**	Case report	1 mother with confirmed COVID-19 (RT-PCR)	1	-	-	-	Pharyngeal swab at any time	-	-	-	-
**Zheng T, 2020 China**	Case report	2 mothers with confirmed COVID-19 (RT-PCR)	2	-	-	-	Pharyngeal swab at any time<br>Blood	-	-	-	-
**Zhou RB, 2020 China**	Case report	1 mother with confirmed COVID-19 (RT-PCR)	1	-	-	-	Pharyngeal swab at birth<br>Pharyngeal swab at any time<br>Blood	-	-	-	-
**Zhuang SY, 2020**	Case report	1 mother with confirmed COVID-19 (RT-PCR)	1	-	-	Breast milk	Pharyngeal swab at any time	-	-	-	-
**Europe and Central Asia**											
**Alay I, 2020 Turkey**	Retrospective cohort	52 mothers: 27 with confirmed COVID-19 (RT-PCR) and 25 with possible COVID-19 (clinical or radiological diagnosis)	14	Women admitted from 25 March to 25 May 2020, due to suspected or diagnosed COVID-19 infection and their neonates	-	-	Pharyngeal swab at any time	-	-	-	-
**Antsaklis P, 2021 Greece**	Retrospective cohort	40 mothers with confirmed COVID-19 (RT-PCR)	35	All COVID-19 pregnant women and their neonates from March to December 2020	-	-	Pharyngeal swab at any time	-	-	-	-

**Artymuk N, 2021 Russia**	Cohort	8485 mothers with confirmed COVID-19 (RT-PCR)	2373	SARS-CoV-2 positive pregnant women and their neonates during a 10 month period	-	-	Pharyngeal swab at any time	-	-	Not classifiable N = 148	12 maternal deaths 30 foetal deaths and 7 neonatal deaths
**Aslan MM, 2020 Turkey**	Prospective cohort	12 mothers with confirmed COVID-19 (RT-PCR)	12 (0 underwent testing)	All pregnant women hospitalised between April 19 and May 19, 2020 with suspected COVID-19 and their neonates	-	Vaginal fluid	-	-	-	-	-
**Barbero P 2020, Spain**	Retrospective cohort	91 mothers with confirmed COVID-19 (RT-PCR)	38 (24 underwent testing)	All mothers diagnosed with COVID-19 during pregnancy or postpartum period, and their neonates between March 3 and May 21 2020	Pregnancy status	-	Pharyngeal swab at any time	-	-	-	-
**Bertero L (1), 2021 Italy**	Retrospective cohort	10 mothers with confirmed COVID-19 (RT-PCR)	11	Placentas delivered from pregnant women with COVID-19 and their neonates from March 22 to July 17 2021	Preterm birth, mode of delivery	Placenta (any side) Maternal stool	Pharyngeal swab at any time	-	-	In Utero: Unlikely N = 1	1 neonatal death
**Bertino E, 2020 Italy***	Prospective cohort	12 mothers with confirmed COVID-19 (RT-PCR)	12	Breastfeeding mothers with COVID-19 and their neonates between April 1 and May 1 2020	Mode of delivery, trimester of infection, ICU admission, pregnancy status, maternal severity, preterm birth, breast feeding	Breast milk	Pharyngeal swab at any time	-	-	In Utero: Indeterminate N = 1 Not classifiable N = 3	-
**Biasucci G, 2020 Italy***	Prospective cohort	15 mothers with confirmed COVID-19 (RT-PCR)	15	COVID-19 positive pregnant women between February 22 and May 15 2020, and their neonates	Preterm birth, mode of delivery, rooming in, breastfeeding	-	Pharyngeal swab at birth Pharyngeal swab at any time	-	-	Not classifiable N = 2	-
**Bozkurt F, 2021 Turkey**	Retrospective cohort	217 mothers with confirmed COVID-19 (RT-PCR)	92	Pregnant women with COVID-19 and their neonates from March 1 to November 30 2020	Maternal death	-	Pharyngeal swab at birth	-	-	In Utero: Indeterminate N = 1	-

							Pharyngeal swab at any time				
**Briana DD, 2021 Greece**	Prospective cohort	13 mothers: 9 with confirmed COVID-19 (RT-PCR), 4 with possible COVID-19 (radiological or clinical diagnosis)	13	Pregnant women with COVID-19 and their neonates between March and September 2020	-	Breast milk	Pharyngeal swab at any time	-	Neonatal stool	-	
**Brito I, 2021 Portugal**	Cohort	77 mothers with confirmed COVID-19 (RT-PCR)	77 (75 underwent testing)	Pregnant women with COVID-19 and their neonates from April 1 2020 to February 7 2021	Preterm birth, rooming-in, breastfeeding, maternal seveity	-	Pharyngeal swab at any time	-	-	In Utero: Indeterminate N = 2 In Utero: Unlikely N=2	-
**Cakirca TD, 2021**	Retrospective cohort	74 mothers with confirmed COVID-19 (RT-PCR)	37 (8 underwent testing)	Pregnant women with confirmed COVID-19 and their neonates from April to August 2020	-	-	Pharyngeal swab at any time	-	-	-	-
**Colson A, 2021 Belgium**	Cohort	31 mothers with confirmed COVID-19 (RT-PCR)	31 (13 underwent testing)	COVID-19 positive pregnant women between April 1 and December 1 2020, and their neonates	Trimester of infection, mode of delivery	Amniotic fluid Placenta (any side) Vaginal swab Rectal swab Serum	Pharyngeal swab at birth Pharyngeal swab at any time Blood	Blood IgM	Blood IgG Placenta RT-PCR, IHC, ISH	-	No maternal death 1 intrauterine death
**Conti MG, 2021 Italy**	Cohort	37 mothers with confirmed COVID-19 (RT-PCR)	37	SARS-CoV-2-positive mothers and their neonates from April 1 2020 to 18 March 2021	Breastfeeding	-	Pharyngeal swab at birth Pharyngeal swab at any time	-	-	In Utero: Possible	-

**Cosma S (2) , 2021 Italy**	Prospective cohort	17 mothers: 5 with confirmed COVID-19 (RT-PCR) and 12 with probable COVID-19 (serology)	17	12-week pregnant patients admitted for COVID-19-related symptoms between April and June 2020 and their neonates<br><br>Excluded women with last menstrual period less than one month after first positive COVID-19	-	-	Pharyngeal swab at any time	-	Cord blood IgG	-	-
**Cribiu FM (1), 2021 Italy***	Cohort	21 mothers with confirmed COVID-19 (RT-PCR)	21	Pregnant women admitted between March 12 and April 23 2020, and their neonates	-	Placenta (any side)	Pharyngeal swab at any time	-	ISH placenta	In Utero: Indeterminate N = 10	No maternal deaths<br>1 foetal death (miscarriag e)
**de Vasconcelos Gaspar A, 2021 Portugal**	Retrospecti ve cohort	12 mothers with confirmed COVID-19 (RT-PCR)	11	Pregnant women with SARS-CoV-2 detection on admission, and their neonates between March 16 to July 31, 2020	-	-	Pharyngeal swab at any time	-	-	-	No maternal death<br>1 foetal death
**Donadieu D, 2020 France**	Prospective cohort	34 mothers: 26 with confirmed COVID-19 (RT-PCR) and 8 with possible COVID-19 (radiological diagnosis)	18	Pregnant women with COVID-19 at a term greater than 25 weeks of amenorrhoea between March 19 and May 20, 2020, and their neonates	-	-	Pharyngeal swab at any time	-	-	-	-
**Doria M, 2020 Portugal**	Prospective cohort	12 mothers with confirmed COVID-19 (RT-PCR)	13 (11 underwent testing)	Pregnant women with COVID-19 and their neonates between March 25 and April 15, 2020	-	-	Pharyngeal swab at any time	-	-	-	-
**Egerup P, 2020 Denmark**	Prospective cohort	29 mothers: 1 with confirmed COVID-19 (RT-PCR)	29 (22 underwent testing)	All pregnant women, their partners and newborns at between April 4 to July 3, 2020	-	-	-	Neonatal blood IgM	Neonatal blood IgG	-	-

		and 28 with probable COVID-19 (serology)									
**Facchetti F, 2020 Italy***	Retrospective cohort	15 mothers with confirmed COVID-19 (RT-PCR)	15	All pregnant women who delivered between February 7 and May 15, 2020 with placental analysis, and neonates born to mothers with COVID-19	Mode of delivery, trimester of infection, ICU admission, pregnancy status, maternal severity, preterm birth	-	Pharyngeal swab at birth<br>Pharyngeal swab at any time	-	Placenta (IHC, ISH, EM)	In Utero: Possible<br>N = 1	-
**Fenizia C, 2020 Italy***	Prospective cohort	31 mothers with confirmed COVID-19 (RT-PCR)	31	Pregnant women with COVID-19 and their neonates between March 9 and April 14, 2020		Amniotic fluid<br>Placenta (any side)<br>Vaginal fluid<br>Breast milk<br>Blood	Pharyngeal swab at birth<br>Cord blood	Cord blood IgM	Cord blood IgG<br>Maternal blood IgM and IgG<br>Breastmilk IgM and IgG	In Utero: Indeterminate<br>N = 1<br>In Utero: Unlikely<br>N = 1	-
**Ferrazzi E (1), 2020 Italy***	Retrospective cohort	42 mothers with confirmed COVID-19 (RT-PCR)	42	Pregnant women diagnosed with COVID-19 and their neonates between March 1 and March 20	Pregnancy status, breast feeding	-	Pharyngeal swab at any time	-	-	Postpartum: Indeterminate<br>N = 1<br>Not classifiable<br>N = 1	-
**Garcia-Ruiz, I 2021 Spain**	Cohort	44 mothers: 33 mothers with confirmed COVID-19 (RT-PCR), 11 mothers with probably COVID-19 (serology)				Amniotic fluid<br>Placenta (any side)	Pharyngeal swab at birth<br>Pharyngeal swab at any time<br>Cord blood PCR	-	-	In Utero: Unlikely<br>N = 2	-
**Halici-Ozturk F, 2021 Turkey**	Prospective cohort	24 mothers with confirmed COVID-19 (RT-PCR)	24 (21 underwent testing)	Women with early pregnancy loss (<24 weeks of gestation) with COVID-19 between September 1 to December 1, 2020	-	Placenta (any side)	Abortion materials	-	-	-	No maternal deaths<br>24 foetal deaths

**Karasu D, 2021 Turkey**	Prospective cohort	61 mothers with confirmed COVID-19 (RT-PCR)	62	Pregnant women with COVID-19 who underwent caesarean section, and their neonates	-	-	Pharyngeal swab at any time	-	-	-	1 maternal death<br>2 intrauterine deaths
**Kayem G, 2020 France***	Prospective cohort	617 mothers: 597 with confirmed COVID-19 (RT-PCR) and 20 with possible COVID-19 (clinical diagnosis)	190	Pregnant women with COVID-19 and their neonates captured from a research network between March 1 and April 14, 2020	Maternal severity	-	Pharyngeal swab at any time	-	-	Not classifiable N = 1	1 maternal death<br>1 neonatal death
**Knight M, 2020 UK***	Prospective cohort	427 mothers with confirmed COVID-19 (RT-PCR)	272 (265 underwent testing)	Pregnant women with COVID-19 and their neonates between March 1 and April 14, 2020	Mode of delivery	-	Pharyngeal swab at any time	-	-	In Utero: Indeterminate N = 6<br>Not classifiable N = 4	5 maternal deaths<br>2 neonatal deaths<br>7 foetal deaths (3 stillbirths, 4 pregnancy loss)
**Korkmaz MF, 2020 Turkey**	Retrospective cohort	22 mothers: 6 with confirmed COVID-19 (RT-PCR) and 16 with possible COVID-19 (clinical or radiological diagnosis)	22	Children with COVID-19 and their mothers between March 5 and May 5, 2020	-	-	Pharyngeal swab at any time	-	-	Postpartum: Indeterminate N = 2	-
**Llorca J, 2020 Spain**	Cohort	8 mothers with confirmed COVID-19 (RT-PCR)	8 (7 underwent testing)	Pregnant women with COVID-19 and their neonates admitted between March 23 and May 26 2020	-	-	Pharyngeal swab at any time	-	-	-	-
**Mand N, 2021 Germany**	Prospective cohort	435 mothers with confirmed COVID-19 (RT-PCR) or probable COVID-19 (RT-PCR)	431 (323 underwent testing)	Pregnant women with COVID-19 and their neonates, captured from the CRONOS German registry between April 3 and November 27, 2020	-	-	Pharyngeal swab at any time	-	-	Not classifiable N=5	4 foetal deaths (stillbirth)

**Maraschini A, 2020 Italy***	Prospective cohort	146 mothers: 142 with confirmed COVID-19 (RT-PCR) and 4 with possible COVID-19 (radiological diagnosis)	149 (147 underwent testing)	All pregnant women with COVID-19 who delivered, and their neonates, between February 25 to April 22, 2020	Maternal severity, mode of delivery, trimester of infection, ICU admission, preterm birth	-	Pharyngeal swab at any time	-	-	In Utero: Indeterminate N = 1<br>Postpartum: Indeterminate N = 2	No maternal death<br>2 foetal deaths (stillbirth)
**Marin Gabriel MA (2), 2020 Spain***	Retrospective cohort	242 mothers: 222 with confirmed COVID-19 (RT-PCR), 19 with probable COVID-19 (serology) and 1 with possible COVID-19 (clinical or radiological diagnosis)	248 (230 underwent testing)	Newborns of mothers with COVID-19 from March 13 to May 31 2020<br><br>Exclusion: women who were symptomatic but did not have a positive PCR or serological test	Mode of delivery, ICU admission, maternal severity, preterm birth	-	Pharyngeal swab at any time	-	-	-	-
**Martinez Perez O, 2020 Spain***	Retrospective cohort	82 mothers with confirmed COVID-19 (RT-PCR)	82 (72 underwent testing)	Pregnant women with COVID-19 who delivered within the next 14 days, and their neonates between March 12 and April 6, 2020	Mode of delivery, maternal severity	-	Pharyngeal swab at birth	-	-	Not classifiable N = 2	-
**Masmejan S, 2020 Switzerland**	Retrospective cohort	13 mothers: 12 with confirmed COVID-19 (RT-PCR) and 1 with probable COVID-19 (serology)	13	Pregnant women with COVID-19 and their neonates between April 1 and May 6, 2020	-	Placenta (baby side)	Pharyngeal swab at any time<br>Cord blood	-	-	-	-
**Mattern J, 2020 France**	Prospective cohort	20 mothers: 1 with confirmed COVID-19 (RT-PCR) and 19 with probable COVID-19 (serology)	20	All pregnant women with COVID-19 and their neonates admitted between May 4 to May 31, 2020	-	-	Pharyngeal swab at any time	-	-	-	-

**Molina EO, 2020 Spain**	Retrospective cohort	20 mothers: 11 with confirmed COVID-19 (RT-PCR) and 9 with probable COVID-19 (serology)	8	Symptomatic pregnant women with COVID-19 infection and their neonates between 12 March and 17 April 2020	-	-	-	Neonatal blood IgM	Neonatal blood IgG	-	-
**Morhart P, 2021 Germany**	Cohort	27 mothers with confirmed COVID-19 (RT-PCR)	27	Neonates born to women who acquired a SARS-CoV-2 infection during pregnancy	-	-	Pharyngeal swab at any time	-	-	Not classifiable N =2	-
**Murphy C, 2020 Republic of Ireland**	Retrospective cohort	26 mothers with confirmed COVID-19 (RT-PCR)	26 (2 underwent testing)	Infants born to women with SARS-CoV-2 detected during pregnancy from 1 March to 1 July 2020	-	-	Pharyngeal swab at any time	-	-	-	-
**NethOSS Juni, 2020 The Netherlands**	Prospective cohort	241 mothers with confirmed COVID-19 (RT-PCR)	75	All pregnant women with COVID-19 and their neonates in The Netherlands from March 1 to June 19 2020	-	-	Pharyngeal swab at any time	-	-	-	1 maternal death<br>13 foetal deaths
**Nizyaeva NV, 2021 Russia**	Cohort	42 mothers with confirmed COVID-19 (RT-PCR)	42	Pregnant women with COVID-19 and their neonates from March to May 2020<br><br>Control: pregnant women with no COVID-19 based on physical examination and PCR test	-	Placenta (any side)	Pharyngeal swab at birth<br>Pharyngeal swab at any time	-	Placenta IHC	-	1 maternal death<br>1 foetal death
**Norman M, 2021 Switzerland***	Prospective cohort	2286 mothers with confirmed COVID-19 (RT-PCR)	2323	All liveborn infants delivered by pregnant women with COVID-19, captured in the Swedish Pregnancy Register from March 11 to January 31 2021	-	-	Pharyngeal swab at any time	-	-		7 neonatal deaths

**Oncel MY, 2020 Turkey***	Prospective cohort	125 mothers with confirmed COVID-19 (RT-PCR)	125 (120 underwent testing)	Newborns born to mothers with COVID-19 between March 15 and Jun 15, 2020	Trimester of infection, preterm birth, mode of delivery, ICU admission, rooming in, breastfeeding, maternal death	Amniotic fluid<br>Placenta (any side)<br>Breast milk	Pharyngeal swab at any time<br>Stool<br>Blood<br>Deep tracheal aspirate	-	-	In Utero: Indeterminate N = 1<br>Peripartum: Unlikely N = 1<br>Not classifiable N = 2	6 maternal deaths<br>1 neonatal death
**Ozsurmeli M, 2021 Turkey**	Cohort	24 mothers with confirmed COVID-19 (RT-PCR)	10	Pregnant patients with confirmed SARS-CoV-2 admitted between March 11 and July 1 2020 and their neonates	-	-	Pharyngeal swab at any time	-	-	-	1 maternal death<br>1 neonatal death
**Patanè L, 2020 Italy***	Retrospective cohort	22 mothers with confirmed COVID-19 (RT-PCR)	22	Pregnant women with COVID-19 who delivered, and their neonates between March 5 and April 21, 2020	-	Placenta (any side)	Pharyngeal swab at any time	-	-	In Utero: Possible N =1<br>In Utero: Indeterminate N = 1	-
**Pecks U, 2020 Germany***	Cohort	247 mothers with COVID-19	183	Pregnant women with COVID-19 and their neonates, until October 1, 2020	-	-	Pharyngeal swab at any ti	-	-	Not classifiable N = 4	2 foetal deaths
**Pissarra S, 2020 Portugal**	Prospective cohort	10 mothers with confirmed COVID-19 (RT-PCR)	10	Pregnant women with confirmed COVID-19 and their neonates between March 16 to April 22, 2020	-	-	Pharyngeal swab at birth<br>Pharyngeal swab at any time	-	-	-	-
**Preβler J, 2020 Germany**	Prospective cohort	18 mothers: 3 with confirmed COVID-19 (RT-PCR) and 15 with possible COVID-19 (clinical or radiological diagnosis)	18	All deliveries with varying degrees of unprotected parental contact with COVID-19 infected personnel	-	-	Stool<br>Blood	-	Maternal and neonatal serum IgA and IgG	Not classifiable N = 1	-

Rathberger K, 2021 Germany	Retrospective cohort	15 mothers with confirmed COVID-19 (RT-PCR)	15 (10 underwent testing)	All pregnant women who presented with SARS-CoV-2 infection, and their neonates between April to December 2020	-	Breast milk Blood IgG	Pharyngeal swab at birth Pharyngeal swab at any time	-	Blood IgG Cord blood IgG	-	-
**Remaeus K, 2020 Sweden**	Retrospective cohort	67 mothers with confirmed COVID-19 (RT-PCR)	67 (62 underwent testing)	Women who were positive for SARS-CoV-2 and gave birth from March 19 until April 26 2020, and their neonates	-	-	Pharyngeal swab at any time	-	-	In Utero: Indeterminate N = 3	No maternal deaths 1 neonatal death 1 foetal death (stillbirth)
**Resta L, 2021 Italy**	Retrospective cohort	81 mothers with confirmed COVID-19 (RT-PCR)	83	Placentas from pregnant women with COVID-19, and their neonates from September 15 2020 to January 31 2021	-	-	Pharyngeal swab at any time	-	Placenta IHC	In Utero: Indeterminate N = 51	-
**Sanchez-Luna M, 2021 Spain**	Prospective cohort	497 mothers: 465 with confirmed COVID-19 (RT-PCR), 29 with probable CO (serology) and 3 with possible COVID-19 (clinical diagnosis)	503 (469 underwent testing)	COVID-19 positive women and their neonates between March 8 and May 26 2020	Mode of delivery, trimester of infection, ICU admission, maternal severity, preterm birth, rooming in, breast feeding, cord clamping, skin to skin contact	Amniotic fluid Placenta (any side) Vaginal fluid Stool Breast milk	Pharyngeal swab at any time Stool Blood	-	-	-	No maternal deaths 1 neonatal death
**Savasi V, 2020 Italy**	Prospective cohort	77 mothers with confirmed COVID-19 (RT-PCR)	77 (57 underwent testing)	Pregnant women with COVID-19 infection and their neonates admitted between February 23 and March 28 2020	Maternal severity	-	Pharyngeal swab at any time	-	-	-	-
**Semeshkin AA, 2020 Russia**	Prospective cohort	20 mothers with confirmed COVID-19 (RT-PCR)	21	Pregnant women with COVID-19 and their neonates between May 1 and May 20, 2020	-	-	Pharyngeal swab at any time	Neonatal blood IgM	Maternal blood IgM and IgG Neonatal blood IgG	In utero: indeterminate N = 1	-

**Servei Català 29/05, 2020 Spain***	Prospective cohort	153 mothers: 141 with confirmed COVID-19 (RT-PCR) and 12 with possible COVID-19 (clinical or radiological diagnosis)	153 (136 underwent testing)	All pregnant women with COVID-19 and their neonates reported from all centres in Catalonia	Mode of delivery, trimester of infection, ICU admission, preterm birth	-	Pharyngeal swab at any time	-	-	In Utero: Indeterminate N = 2<br>Not classifiable N = 2	-
**Shmakov R, 2020 Russia**	Prospective cohort	66 mothers with confirmed COVID-19 (RT-PCR)	42	Women with confirmed COVID-19 admitted between April and June 2020, and their neonates	-	Amniotic fluid<br>Placenta (any side)<br>Vaginal fluid<br>Breast milk<br>Rectal swab	Pharyngeal swab at birth<br>Pharyngeal swab at any time	-	-	-	1 maternal death<br>No foetal/neonatal deaths
**Sinaci S, 2021 Turkey**	Cross-sectional cohort	48 mothers with confirmed COVID-19 (RT-PCR)	49	Women with confirmed COVID-19 admitted between September 10 and November 23 2020, and their neonates	Mode of delivery, preterm birth	Amniotic fluid<br>Placenta tissue (any side)<br>Vaginal fluid	Pharyngeal swab at birth<br>Pharyngeal swab at any time<br>Cord blood	Blood IgM	Blood IgG	In Utero: Indeterminate N = 2	No maternal death<br>1 neonatal death
**Solis-Garcia G, 2020 Spain**	Prospective cohort	73 mothers with confirmed COVID-19 (RT-PCR)	75	Neonates born to COVID-19 positive mothers between 1 March and 17 August 2020, and their mothers	-	-	Pharyngeal swab at any time	-	-	Not classifiable N = 1	No maternal deaths<br>2 neonatal deaths
**Tallarek A, 2021 Germany**	Cohort	42 mothers with confirmed COVID-19 (RT-PCR)	75	Neonates born to COVID-19 positive mothers between 1 March and 17 August 2020, and their mothers	-	Placenta (any side)<br>Breast milk<br>Blood IgG	Pharyngeal swab at any time<br>Cord blood PCR	-	Placenta RT-PCR<br>Cord blood IgG	-	-
**Tasca C, 2021 Italy***	Prospective cohort	64 mothers with confirmed COVID-19 (RT-PCR)	64	Pregnant women with COVID-19 and their neonates from March to August 2020	-	Placenta (any side)	Pharyngeal swab at birth<br>Pharyngeal swab at any time	-	-	In Utero: Indeterminate N = 7	-

**Vousden N, 2020 UK***	Prospective cohort	1148 mothers with confirmed COVID-19 (RT-PCR)	1003	Hospitalised pregnant women with confirmed SARS-CoV-2 and their neonates from 1 March and 31 August 2020	-	-	Pharyngeal swab at any time	-	-	-	8 maternal deaths<br>4 neonatal deaths<br>9 foetal deaths (stillbirth)
**Yaman A, 2021 Turkey**	Cohort	20 mothers with confirmed COVID-19 (RT-PCR)	20	Neonates born to mothers with COVID-19, and neonates with SARS-CoV-2 infection between April to December 2020	-	-	Pharyngeal swab at any time	-	-	Not classifiable N = 6	-
**Zaharie G, 2020 Romania**	Retrospecti ve cohort	5 mothers with confirmed COVID-19 (RT-PCR)	5	Neonates born to mothers with COVID-19 between April 1 and May 15, 2020	-	-	Pharyngeal swab at any time	-	-	-	-
**Bouachba A, 2021 France**	Case series	5 mothers with confirmed COVID-19 (RT-PCR)	5	-	-	Amniotic fluid<br>Placenta (any side)	Pharyngeal swab at any time	-	Placenta RT_PCR, IHC	In Utero: Indeterminate N=2<br>In Utero (IUFD): Indeterminate N=3	-
**Buonsenso D (1), 2020 Italy***	Case series	7 mothers with confirmed COVID-19 (RT-PCR)	2	-	-	Amniotic fluid<br>Placenta (any side)<br>Breast milk	Pharyngeal swab at any time<br>Stool<br>Cord blood	Neonatal Blood IgM	Neonatal Blood IgG	In Utero: Indeterminate N = 1<br>Not classifiable N = 1	No maternal deaths<br>1 foetal death (abortion)
**Elósegui H, 2020 Spain**	Case series	4 mothers with confirmed COVID-19 (RT-PCR)	4 (0 underwent testing)	-	-	Amniotic fluid<br>Vaginal fluid	-	-	-	-	-
**Garcia-Manau P, 2020 Spain**	Case series	2 mothers with confirmed COVID-19 (RT-PCR)	2 (0 underwent testing)	-	-	-	Pharyngeal swab at any time	-	-	-	-
**Gregorio-Hernández R, 2020 Spain**	Case series	1 mother with confirmed COVID-19 (RT-PCR)	1	-	-	-	Pharyngeal swab at any time	-	-	Not classifiable N = 1	-
**Marin Gabriel MA, 2020 Spain**	Case series	7 mothers with confirmed COVID-19 (RT-PCR)	7	-	-	Breast milk	Pharyngeal swab at any time	-	-	-	-

**Menter T, 2020 Switzerland**	Case series	5 mothers with confirmed COVID-19 (RT-PCR)	5 (1 underwent testing)	-	-	Amniotic fluid<br>Placenta (any side)<br>Breast milk	Pharyngeal swab at any time<br>Cord blood	-	-	In Utero: Indeterminate N = 1	-
**Olivini N, 2020 Italy**	Case series	5 mothers with confirmed COVID-19 (RT-PCR)	5	-	-	Breastmilk	Pharyngeal swab at any time<br>Stool	-	-	Not classifiable N = 4	-
**Perrone S, 2020 Italy***	Case series	4 mothers with confirmed COVID-19 (RT-PCR)	4 (3 underwent testing)	-	-	Placenta (any side)	Pharyngeal swab at any time	-	-	-	-
**Radu MC, 2021 Romania**	Case series	7 mothers with confirmed COVID-19 (RT-PCR)	7	-	-	-	Pharyngeal swab at any time	-	-	-	-
**Saviron-Cornudella R 2020**	Case series	6 mothers with confirmed COVID-19 (RT-PCR)	6	-	-	-	Pharyngeal swab at any time	-	-	-	-
**Vashukova MA, 2020 Russia**	Case series	6 mothers with confirmed COVID-19 (RT-PCR)	6 (5 underwent testing)	-	-	-	Pharyngeal swab at any time	-	-	In Utero: Indeterminate N = 1	-
**Abadía-Cuchi N, 2021 Spain**	Case report	1 mother with confirmed COVID-19 (RT-PCR)	2	-	-	Placenta swab (any side)	Pharyngeal swab at birth<br>Pharyngeal swab at any time	Blood IgM	Blood IgG	In Utero: Unlikely N = 1<br>In Utero: Possible N = 1	-
**Abasse S, 2020 France**	Case report	1 mother with confirmed COVID-19 (RT-PCR)	1	-	-	-	Pharyngeal swab at any time	-	-	In Utero: Indeterminate N = 1	-
**Akdemir Y, 2021 Turkey**	Case report	1 mother with confirmed COVID-19 (RT-PCR)	1	-	-	Amniotic fluid<br>Placenta swab (any side)<br>Placenta swab (foetal side)<br>Vaginal fluid	Pharyngeal swab at birth<br>Pharyngeal swab any time<br>Cord blood PCR	-	-	In Utero: Unlikely N = 1	-

						Endometrium inner surface swab					
**Alario D, 2020 Italy**	Case report	1 mother with possible COVID-19 (RT-PCR)	1	-	-	-	Pharyngeal swab at any time<br>Stool	-	-	Not classifiable<br>N = 1	-
**Banerjee A, 2020**	Case report	1 mother with possible COVID-19 (RT-PCR)	1	-	-	-	Pharyngeal swab at any time	-	-	-	-
**Bastug A, 2020 Turkey**	Case report	1 mother with confirmed COVID-19 (RT-PCR)	1	-	-	Breast milk	Pharyngeal swab at any time<br>Stool<br>Blood	-	-	Postpartum: Confirmed<br>N = 1	-
**Baud D, 2020 Switzerland**	Case report	1 mother with confirmed COVID-19 (RT-PCR)	1 (IUFD)	-	-	Amniotic fluid<br>Placenta (any side)<br>Placenta (baby side)<br>Vaginal fluid	Pharyngeal swab at birth<br>Stool<br>Blood<br>Cord blood<br>Foetal tissues	-	-	In Utero (IUFD): Unlikely<br>N = 1	Foetal death (IUFD)
**Caplliure AP, 2020 Spain**	Case report	1 mother with confirmed COVID-19 (RT-PCR)	1	-	-	-	Pharyngeal swab at any time	Neonatal blood IgM	Neonatal blood IgG	Not classifiable<br>N = 1	-
**Carbayo-Jimenez T, 2021 Spain**	Case report	1 mother with confirmed COVID-19 (RT-PCR)	1	-	-	-	Pharyngeal swab at any time<br>Blood	Neonatal blood IgM	Neonatal blood IgG	In Utero: Possible<br>N = 1	-
**Cavicchiolo ME, 2020 Italy***	Case report	2 mothers with confirmed COVID-19 (RT-PCR)	2	-	-	-	Pharyngeal swab at any time	Neonatal blood IgM	Neonatal blood IgG	-	-
**Cavaliere AF (2), 2020 Italy**	Case report	1 mother with confirmed COVID-19 (RT-PCR)	1	-	-	-	Pharyngeal swab at any time	Neonatal blood IgM	Neonatal blood IgG	-	-

**Correia CR, 2020 Portugal**	Case report	1 mother with confirmed COVID-19 (RT-PCR)	1	-	-	Stool	Pharyngeal swab at any time<br>Stool<br>Blood	Neonatal blood IgM	Neonatal blood IgG	In Utero: Confirmed<br>N = 1	-
**Demirjian A, 2020 UK***	Case report	1 mother with confirmed COVID-19 (RT-PCR)	1	-	-	Blood	Pharyngeal swab at any time<br>Stool<br>Blood<br>Respiratory secretions<br>Cerebrospi nal fluid<br>Urine	-	-	Postpartum: Confirmed<br>N = 1	-
**De Socio GV, 2020 Italy***	Case report	1 mother with confirmed COVID-19 (RT-PCR)	1	-	-	-	Pharyngeal swab at any time	Neonatal stool	-	-	-
**Deveci MF, 2020 Turkey**	Case report	1 mother with confirmed COVID-19 (RT-PCR)	1	-	-	-	Pharyngeal swab at any time	-	-	Not classifiable<br>N = 1	-
**Di Nicola P, 2020 Italy**	Case report	1 mother with confirmed COVID-19 (RT-PCR)	1	-	-	-	Pharyngeal swab at any time	-	-	Not classifiable<br>N = 1	-
**Diaz CA, 2020 Spain**	Case report	1 mother with confirmed COVID-19 (RT-PCR)	1	-	-	-	Pharyngeal swab at any time	-	-	Not classifiable<br>N = 1	-
**Dima M, 2020 Romania**	Case report	3 mothers with confirmed COVID-19 (RT-PCR)	3	-	-	-	Pharyngeal swab at any time	-	-	Not classifiable<br>N = 3	-
**Faure-Bardon V, 2021**	Case report	1 mother with confirmed COVID-19 (RT-PCR)	1 (0 underwent testing)	-	-	Placenta (any side)	-	-	-	-	-
**Ferraiolo A, 2020 Italy***	Case report	1 mother with confirmed COVID-19 (RT-PCR)	1	-	-	Placenta (any side)<br>Placenta (baby side)	Pharyngeal swab at any time	Neonatal blood IgM	Neonatal blood IgG	In Utero: Unlikely<br>N = 1	-

									Maternal IgG and IgM		
**Figueiredo R, 2020 Portugal**	Case report	1 mother with confirmed COVID-19 (RT-PCR)	1	-	-	-	Pharyngeal swab at birth<br>Pharyngeal swab at any time	-	-	-	-
**Filimonovic D, 2020 Serbia**	Case report	1 mother with confirmed COVID-19 (RT-PCR)	2	-	-	Amniotic fluid	Pharyngeal swab at birth<br>Pharyngeal swab at any time<br>Cord blood PCR	Cord blood IgM	Cord blood IgG	-	-
**Gaunt P, 2020 UK**	Case report	1 mother with confirmed COVID-19 (RT-PCR)	2	-	-	-	Pharyngeal swab at any time	-	-	In Utero: Indeterminate N = 2	-
**Grimminck K, 2020 The Netherlands**	Case report	1 mother with confirmed COVID-19 (RT-PCR)	1	-	-	Placenta (any side)<br>Placenta (baby side)<br>Vaginal fluid	Pharyngeal swab at any time	-	-	-	-
**Gordon M, 2020 UK***	Case report	1 mother with confirmed COVID-19 (RT-PCR)	1	-	-	-	Pharyngeal swab at any time	-	-	Postpartum: Confirmed N = 1	-
**Groβ R, 2020 Germany**	Case report	2 mothers with confirmed COVID-19 (RT-PCR)	2	-	-	Breast milk	Pharyngeal swab at any time	-	-	Postpartum: Indeterminate N = 1<br>Not classifiable N = 1	-
**Grimminck K, 2020 The Netherlands**	Case report	1 mother with confirmed COVID-19 (RT-PCR)	1	-	-	Placenta (any side)<br>Placenta (baby side)<br>Vaginal fluid	Pharyngeal swab at any time	-	-	-	-
**Hachem R, 2020 France**	Case report	1 mother with confirmed COVID-19 (RT-PCR)	1 (0 underwent testing)	-	-	Placenta (any side)	-	-	Placenta RT-PCR	-	No maternal death<br>1 foetal death
**Hascoet JM, 2020 France**	Case reports	4 mothers: 3 with confirmed	4	-	-	Stool<br>Breast milk	Pharyngeal swab at	Neonatal blood IgM	Neonatal blood IgG	In Utero: unlikely N = 1	-

		COVID-19 (RT-PCR) and 1 with possible COVID-19 (clinical diagnosis)					birth<br>Pharyngeal swab at any time<br>Stool			In Utero: Indeterminate<br>N = 1	
**Janjua B 2021, Ireland**	Case report	1 mother with confirmed COVID-19 (RT-PCR)	1	-	-	-	Pharyngeal swab at any time	-	-	Not classifiable<br>N = 1	-
**Kalafat E, 2020 Turkey***	Case report	1 mother with confirmed COVID-19 (RT-PCR)	1	-	-	Placenta (any side)<br>Placenta (foetal side)<br>Breast milk	Pharyngeal swab at any time<br>Cord blood PCR	-	-	-	-
**Keklik ESK, 2020 Turkey**	Case report	1 mother with confirmed COVID-19 (RT-PCR)	1	-	-	-	Pharyngeal swab at any time	-	-	-	-
**Komiazyk M, 2021 Poland**	Case report	1 mother with confirmed COVID-19 (RT-PCR)	1	-	-	-	Pharyngeal swab at any time	-	-	Postpartum: confirmed<br>N = 1	-
**Konstantinidou A, 2020 Greece**	Case report	2 mothers with confirmed COVID-19 (RT-PCR)	2	-	-	Placenta (any side)	Pharyngeal swab at any time	-	Neonatal stool	-	-
**Lehner G, 2020 Austria**	Case report	1 mother with confirmed COVID-19 (RT-PCR)	1	-	-	-	Pharyngeal swab at any time<br>Blood	-	Blood IgG	-	-
**Lenoci G, 2020 Italy**	Case report	1 mother with confirmed COVID-19 (RT-PCR)	1	-	-	-	Pharyngeal swab at any time	-	-	Not classifiable<br>N = 1	-
**L'Hullier AG (1), 2020 Switzerland**	Case report	1 mother with confirmed COVID-19 (RT-PCR)	1	Neonates with COVID-19 and their mothers	-	-	Pharyngeal swab at any time	-	-	Postpartum: Indeterminate<br>N=1	-
**Libbrecht S, 2021 Belgium**	Case report	2 mothers with confirmed COVID-19 (RT-PCR)	3	-	-	Placenta (any side)	Pharyngeal swab at any time	-	Placenta RT-PCR, IHC	In Utero (IUFD): possible<br>N=1<br>In Utero: Indeterminate<br>N=2	No maternal death<br>1 intrauterine death

**Lugli L 2020**	Case report	1 mother with confirmed COVID-19 (RT-PCR)	1	-	-	Breast milk	Pharyngeal swab at any time	Blood IgM	Blood IgG Neonatal stool	-	-
**Markovic M, 2020 Serbia**	Case report	1 mother with confirmed COVID-19 (RT-PCR)	1	-	-	Amniotic fluid	Pharyngeal swab at any time Cord blood PCR	Cord blood IgM	Cord blood IgG	-	-
**Martenot A, 2020 France***	Case report from retrospectiv e cohort	26 mothers with confirmed COVID-19 (RT-PCR)	26 (25 underwent testing)	-	-	Breast milk	Pharyngeal swab at birth Pharyngeal swab at any time	-	Neonatal stool	Not classifiable N = 1	-
**McDevitt KEM, 2020 UK**	Case report from retrospectiv e cohort	1 mother with confirmed COVID-19 (RT-PCR)	1	-	-	-	Pharyngeal swab at any time	-	-	Not classifiable N = 1	-
**Michel A, 2021 France***	Case report	1 mother with probable COVID-19 (clinical diagnosis)	1	-	-	Placenta (any side) Pharyngeal swab Stool	Foetal liver and lung tissue	-	Maternal blood IgM and IgG	In Utero (IUFD): Unlikely N = 1	Foetal death (miscarriag e)
**Norbert L, 2020 Germany**	Case report	1 mother with confirmed COVID-19 (RT-PCR)	1	-	-	-	Pharyngeal swab at any time	-	Neonatal stool	Postpartum: Indeterminate N = 1	-
**Ozer E, 2020 Turkey**	Case report	1 mother with confirmed COVID-19 (RT-PCR)	1	-	-	-	Pharyngeal swab at any time	-	-	-	-
**Paramanath an S, 2021 Denmark**	Case report	1 mother with confirmed COVID-19 (RT-PCR)	1	-	-	-	Pharyngeal swab at any time	Blood IgM	Blood IgG Neonatal stool Tracheal aspirate Urine	-	-
**Perrone S (1), 2020 Italy**	Case report	1 mother with confirmed COVID-19 (RT-PCR)	1	-	-	Breast milk	Pharyngeal swab at any time	-	-	-	-
**Piersigilli F, 2020 Belgium**	Case report	1 mother with confirmed COVID-19 (RT-PCR)	1	-	-	Breast milk	Pharyngeal swab at any time	-	-	Not classifiable N = 1	-

**Pinana M, 2021 Spain**	Case report	1 mother with confirmed COVID-19 (RT-PCR)	1	-	-	Amniotic fluid<br>Placenta (any side)	Pharyngeal swab at birth<br>Blood PCR<br>Cord blood PCR	-	Placenta IHC and ISH<br>Blood IgG	In Utero: Indeterminate<br>N = 1	-
**Popescu DE, 2021 Romania**	Case report	1 mother with confirmed COVID-19 (RT-PCR)	1 (0 underwent testing)	-	-	Placenta (any side)	-	-	Placenta (IHC)	In Utero (IUFD): Possible<br>N = 1	1 foetal death (stillbirth)
**Pulinx B, 2020 Belgium**	Case report	1 mother with confirmed COVID-19 (RT-PCR)	2 (0 underwent testing)	-	-	Amniotic fluid<br>Placenta (any side)<br>Blood	-	-	Placenta (IHC)	In Utero (IUFD): Possible<br>N = 2	2 foetal deaths (stillbirth)
**Quigley N 2021, Ireland**	Case report	1 mother with confirmed COVID-19 (RT-PCR)	1	-	-	-	Pharyngeal swab at any time	-	-	-	-
**Resta L (1), 2021 Italy**	Case report	1 mother with confirmed COVID-19 (RT-PCR)	1	-	-	-	Pharyngeal swab at any time	-	Placenta (IHC)	In Utero: Indeterminate<br>N = 2	1 neonatal death
**Rodrigues ML, 2020 Portugal**	Case report	1 mother with confirmed COVID-19 (RT-PCR)	1	-	-	-	Foetal lung (stillbirth)	-	-	In Utero (IUFD): Confirmed<br>N = 1	1 foetal death (stillbirth)
**Saikia B, 2021 UK**	Case report	1 mother with confirmed COVID-19 (RT-PCR)	1	-	-	-	Pharyngeal swab at any time	-	-	Not classifiable<br>N = 1	-
**Santos LM, 2020 Spain**	Case report	1 mother with confirmed COVID-19 (RT-PCR)	1	-	-	-	Pharyngeal swab at any time	-	-	-	-
**Santos RR, 2020 Portugal**	Case report	2 mothers with confirmed COVID-19 (RT-PCR)	2	-	-	-	Pharyngeal swab at any time	-	-	-	-
**Savic D, 2021 Serbia**	Case report	1 mother with confirmed COVID-19 (RT-PCR)	1	-	-	-	Pharyngeal swab at any time	-	-	In Utero: Indeterminate<br>N = 1	-

**Schoenmakers S, 2020<br>Netherlands<br>***	Case report	1 mother with confirmed COVID-19 (RT-PCR)	1	-	-	Placenta (any side)<br>Placenta (baby side)<br>Vaginal fluid<br>Stool<br>Breast milk<br>Blood<br>Urine<br>Faeces	Pharyngeal swab at any time<br>Stool<br>Blood<br>Cord blood<br>Sputum<br>Urine	Neonatal blood IgM<br>Cord blood IgM	Placenta (IHC, ISH and EM)<br>Neonatal blood IgG<br>Neonatal cord blood IgG<br>Maternal IgM and IgG	In Utero: Unlikely<br>N = 1	-
**Sileo FG, 2020<br>Italy**	Case report	1 mother with confirmed COVID-19 (RT-PCR)	1	-	-	Placenta (any side)	Pharyngeal swab at birth<br>Pharyngeal swab at any time	Cord blood IgM	Cord blood IgG	-	-
**Sinelli MT, 2020<br>Italy**	Case report	1 mother with confirmed COVID-19 (RT-PCR)	1	-	-	-	Pharyngeal swab at any time	-	-	Not classifiable<br>N = 1	-
**Sivevski A, 2020<br>Macedonia**	Case report	1 mother with confirmed COVID-19 (RT-PCR)	1	-	-	-	Pharyngeal swab at any time	-	-	-	1 maternal death
**Sukhikh G, 2021<br>Russia**	Case report	1 mother with confirmed COVID-19 (RT-PCR)	1	-	-	Placenta (any side)	Cord blood	-	Placenta IHC<br>Neonatal blood IgG	In Utero: Indeterminate<br>N = 1	1 neonatal death
**Toto V, 2021<br>Italy**	Case report	1 mother with confirmed COVID-19 (RT-PCR)	1	-	-	-	Pharyngeal swab at birth	-	Placenta IHC	In Utero: Indeterminate<br>N = 1	-
**Trombetta A, 2021<br>Italy**	Case report	1 mother with confirmed COVID-19 (RT-PCR)	1	-	-	Placenta (any side)<br>Vaginal fluid	Pharyngeal swab at any time	-	Cord blood IgG	-	-
**Tsatsaris V, 2020 France**	Case report	1 mother with probable COVID-19 (serology)	1	-	-	Foetal tissues	-	-	-	-	No maternal death<br>1 foetal death
**Vivanti A, 2020<br>France***	Case report	1 mother with confirmed COVID-19 (RT-PCR)	1	-	-	Amniotic fluid<br>Placenta (any side)<br>Vaginal fluid<br>Blood	Pharyngeal swab at any time<br>Blood<br>Rectal swab	-	-	In Utero: Possible<br>N = 1	-

**Yildiz H, 2021 Turkey**	Case report	1 mother with confirmed COVID-19 (RT-PCR)	1	-	-	-	Pharyngeal swab at any time	-	Neonatal stool antigen<br>CSF	Postpartum Possible<br>N = 1	-
**Yilmaz R, 2020 Turkey**	Case report	1 mother with confirmed COVID-19 (RT-PCR)	1	-	-	-	Pharyngeal swab at any time	-	-	-	-
**Zaigham M (1), 2021 Sweden**	Case report	1 mother with confirmed COVID-19 (RT-PCR)	1	-	-	-	Pharyngeal swab at any time	-	Placenta IHC	In Utero: Possible<br>N = 1	-
**Zhumabekova A, 2020 Kyrgyzstan**	Case report	1 mother with confirmed COVID-19 (RT-PCR)	1	-	-	-	Pharyngeal swab at birth<br>Pharyngeal swab at any time	-	-	-	-
**Latin America and The Caribbean**											
**Aliaga CD, 2020 Peru**	Retrospective cohort	114 mothers with probable COVID-19 (serology)	114	Pregnant women with COVID-19 and their neonates between April 15 and May 10 2020	Mode of delivery, preterm birth	-	-	Neonatal blood IgM	Neonatal blood IgG	-	No maternal deaths<br>3 neonatal deaths
**Aliaga CD (1), 2020 Peru**	Retrospective cohort	43 mothers with confirmed COVID-19 (RT-PCR)	43	Neonates born to COVID positive mothers between 1 April and 30 June 2020	Mode of delivery, preterm birth	-	Pharyngeal swab at any time	-	-	In Utero: Indeterminate<br>N = 1	-
**Cardona-Perez JA, 2021 Mexico**	Retrospective cohort	70 mothers confirmed with COVID-19 (serology)	63 (30 underwent testing)		-	-	Pharyngeal swab at any time	-	-	Not classifiable<br>N=9	No maternal death<br>5 foetal deaths<br>1 neonatal death
**Cubas JAC (1) 2020, Peru**	Retrospective cohort	13 mothers confirmed with COVID-19 (serology)	13 (12 underwent testing)	Pregnant women with COVID-19 and their neonates between March 6 and June 15, 2020	-	-	-	Neonatal blood IgM	Neonatal blood IgG	-	No maternal deaths<br>1 foetal death (stillbirth)
**de Fatima Yukie**	Cohort	79 mothers with confirmed COVID-19	109 (54 underwent testing)	Pregnant women with COVID-19 and their neonates between	Mode of delivery, breastfeeding	Amniotic fluid<br>Breast milk	Pharyngeal swab at any time	-	-	In Utero: Possible<br>N=2<br>In Utero: Unlikely	4 maternal deaths

**Maeda M, 2021 Brazil**		(RT-PCR) and 30 mothers with probable COVID-19 (serology)		April 12 to September 30 2020			Cord blood			N=3<br>In Utero: Indeterminate<br>N=2	No neonatal deaths
**Diaz-Corvillon P, 2020 Chile**	Prospective cohort	37 mothers with confirmed COVID-19 (RT-PCR)	37	Pregnant women admitted between 27 April and 7 June 2020, and their neonates	Mode of delivery, ICU admission, maternal severity, preterm birth	-	Pharyngeal swab at any time	-	-	In Utero: Possible<br>N = 2	No maternal deaths<br>2 foetal deaths (stillbirth)<br>2 neonatal deaths
**Elenga N, 2021 French Guiana**	Prospective cohort	133 mothers with confirmed COVID-19 (RT-PCR)	132 (32 underwent testing)	All neonates born between May 14 and August 31 2020 to SARS-CoV-2 positive mothers at the time of delivery<br><br>Controls: pregnant women without COVID-19, matched by age	ICU admission	-	Pharyngeal swab at any time	-	-	Postpartum: Indeterminate<br>N = 3	No maternal deaths<br>4 foetal deaths (IUFD)
**Flores-Pliego A, 2021 Mexico***	Cohort	11 mothers with confirmed COVID-19 (RT-PCR)	11	Pregnant women with confirmed COVID-19 and their neonates	Maternal severity, trimester of infection, preterm birth	Placenta (any side)	Pharyngeal swab at any time	-	-	In Utero (IUFD): Possible<br>N = 1<br>Not classifiable<br>N = 4	-
**Haye MT, 2021 Chile**	Cohort	458 mothers with confirmed COVID-19 (RT-PCR)	221 (107 underwent testing)	Pregnant women with confirmed PCR for SARS-CoV-2 infection, and their neonates from April 8 to August 30 2020	-	-	Pharyngeal swab at any time	-	-	In Utero: Possible<br>N=1<br>In Utero: Unlikely<br>N=9	1 maternal death<br>1 neonatal death

**Hcini N, 2020 French Guiana**	Prospective cohort	137 mothers with confirmed COVID-19 (RT-PCR)	137 (127 underwent testing)	Pregnant women admitted for delivery beyond 15 weeks of gestation and tested positive for SARS-CoV-2 infection between 16 June and 16 August 2020, and their neonates<br><br>Control group: women admitted for delivery beyond 15 weeks of gestation and tested negative for SARS-CoV-2	-	Placenta (any side)	Pharyngeal swab at birth<br>Pharyngeal swab at any time	-	-	Peripartum: Possible<br>N = 2<br>In Utero: Indeterminate<br>N = 2	No maternal deaths<br>10 foetal deaths (7 IUFD, 3 abortions)
**Hernández OB, 2020 Chile**	Prospective cohort	661 mothers: 655 with confirmed COVID-19 (RT-PCR), 1 with probable COVID-19 (serology), and 5 with possible COVID-19 (clinical or radiological diagnosis)	389 (316 underwent testing)	Pregnant and postpartum women with SARS-CoV-2 d between March 7 and July 6 2020, and their neonates	-	-	Pharyngeal swab at any time	-	-	In Utero: Indeterminate<br>N = 10<br>Postpartum: Indeterminate<br>N = 4<br>Not classifiable<br>N = 7	No maternal deaths<br>6 perinatal deaths
**Huerta Saenz IH, 2020 Peru***	Prospective cohort	41 mothers: 24 with confirmed COVID-19 (RT-PCR) and 17 with probable COVID-19 (serology)	35	All pregnant women with COVID-19 and their neonates between March 24 and May 7, 2020	Mode of delivery, pregnancy status, maternal severity	-	Pharyngeal swab at any time	-	Maternal blood rapid serological test	Not classifiable<br>N = 1	-
**Lizama O, 2021 Peru**	Retrospective cohort	201 mothers with probable COVID-19 infection (serology)	206	Pregnant women with COVID-19 and their neonates from March 15 to June 30, 2020	Mode of delivery, preterm birth, maternal death, breast feeding	-	Pharyngeal swab at any time	-	-	Not classifiable<br>N = 4	1 maternal death<br>No neonatal death
**Moreira LMO, 2021 Brazil**	Cross-sectional cohort	28 mothers with confirmed or probable COVID-19	28	Pregnant women with confirmed COVID-19 and their neonates between May 24 to July 17 2020	-	Blood IgM and IgG	Pharyngeal swab at any time	-	-	In Utero: Indeterminate<br>N = 2	-

**Rebutini PZ, 2021 Brazil**	Prospective cohort (case -control)	17 mothers with confirmed COVID-19 (RT-PCR) and 2 with probable COVID-19 (serology)	19 (13 underwent testing)	Pregnant women with confirmed and probable COVID-19 and their neonates<br><br>Control group: pregnant women who delivered prior to the SARS-CoV-2 outbreak	Trimester of infection, preterm birth, maternal severity, maternal death	Amniotic fluid<br>Placenta tissue (any side)<br>Blood IgM and IgG	Pharyngeal swab at any time<br>Cord blood	-	Placenta RT-PCR	In Utero: Indeterminate N=4<br>In Utero (IUFD): Possible N=1	3 neonatal deaths
**Sola A, 2020 Latin America***	Prospective cohort	86 mothers with confirmed COVID-19 (RT-PCR)	86	Pregnant women with COVID-19 and their neonates from March 6 to May 30, 2020	-	-	Pharyngeal swab at any time	-	-	In Utero: Indeterminate N = 5	-
**Teixeira MLB, 2021 Brazil**	Cohort	33 mothers: 13 with confirmed COVID-19 (RT-PCR) and 20 with probable COVID-19 (serology)	28 (1 underwent testing)	Pregnant women with COVID-19 and their neonates admitted between April 13 and June 17 2020	-	Placenta (any side)	-	-	Placenta RT-PCR	In Utero (IUFD): Possible	-
**Vera Loyola EM, 2021 Peru**	Cohort	345 mothers with probable COVID-19 infection (serology)	349 (334 underwent testing)	Pregnant women with COVID-19 and their neonates between April to July 2020	Mode of delivery	-	Pharyngeal swab at birth<br>Pharyngeal swab at any time	-	-	In utero: Indeterminate N = 11	No maternal deaths<br>11 foetal deaths (stillbirth)
**2020 July Informe Epidemiológico Embarazadas y Puerperas sem28, 2020 Mexico***	Prospective cohort	76 mothers: 72 with confirmed COVID-19 (RT-PCR) and 4 with possible COVID-19 (clinical or radiological diagnosis)	76	Pregnant and postpartum women diagnosed with COVID-19 and their neonates	-	-	Pharyngeal swab at any time	-	-	Not classifiable N = 56	64 maternal deaths
**Baquero H, 2020 Colombia**	Case series	2 mothers with confirmed COVID-19 (RT-PCR)	2	-	-	-	Pharyngeal swab at any time	-	-	Not classifiable N = 2	-
**dos Reis HLB, 2020 Brazil**	Case series	3 mothers with confirmed COVID-19 (RT-PCR)	3	-	-	-	Pharyngeal swab at any time	-	-	-	1 maternal death<br>No neonatal death

**Dos Santos Beozzo GPN, 2020 Brazil**	Case series	3 mothers: 2 with confirmed COVID-19 (RT-PCR) and 1 with possible COVID-19 (radiological diagnosis)	3	-	-	-	Pharyngeal swab at any time	-	Neonatal blood IgG	Peripartum: Possible N = 2 Not classifiable N = 1	-
**Gorodezky TM, 2020 Chile**	Case series	6 mothers with confirmed COVID-19 (RT-PCR)	6	-	-	-	Pharyngeal swab at any time	-	-	-	-
**Kiappe OP, 2021 Brazil**	Case series from cohort	165 mothers with confirmed or probable COVID-19	165	Pregnant women with COVID-19 and their neonates from April to November 2020	-	-	Pharyngeal swab at any time	-	-	In Utero: Indeterminate N = 5 Not classifiable N = 1	-
**Perez-Chimal LG, 2021 Mexico**	Case series	10 mothers with confirmed COVID-19 (RT-PCR)	10	-	-	-	Pharyngeal swab at any time	-	-	Not classifiable N = 10	-
**Richtmann R, 2020 Brazil**	Case series	5 mothers with confirmed COVID-19 (RT-PCR)	3 (0 underwent testing)	-	-	Amniotic sac Placenta (any side)	-	-	-	In Utero (IUFD): Possible N = 2	No maternal deaths 5 foetal deaths (IUFD)
**Alvarado-Socarras JL, 2021**	Case report	1 mother with confirmed COVID-19 (RT-PCR)	1	-	-	-	Pharyngeal swab at any time	-	-	Not classifiable N = 1	-
**Alzamora M, 2020 Peru***	Case report	1 mother with confirmed COVID-19 (RT-PCR)	1	-	-	-	Pharyngeal swab at birth	Neonatal blood IgM	Neonatal blood IgG	In Utero: Possible N = 1	-
**Behling JAK, 2020 Brazil**	Case report	1 mother with possible COVID-19 (RT-PCR)	1	-	-	Placenta (any side)	Foetal tissues (liver, heart, lung, brain)	-	-	In Utero: Confirmed N = 1	Neonatal death
**Braga LFB, 2020 Brazil**	Case report	1 mother with possible COVID-19 (RT-PCR)	2	-	-	-	Pharyngeal swab at any time	-	-	-	1 neonatal death
**Falcão M, 2020 Brazil**	Case report	1 mother with confirmed COVID-19 (RT-PCR)	1	-	-	-	Pharyngeal swab at any time	-	-	Not classifiable N = 1	-

**Figueiredo de Montalvao Franca AP, 2020 Brazil**	Case report	1 mother with confirmed COVID-19 (RT-PCR)	1	-	-	-	Pharyngeal swab at any time	-	-	-	1 maternal death
**Forera-Pena DA, 2020 Venezuela**	Case report	1 mother with confirmed COVID-19 (RT-PCR)	1	-	-	-	Pharyngeal swab at any time	-		-	
**Fragoso DC, 2021 Brazil**	Case report	1 mother with confirmed COVID-19 (RT-PCR)	1	-	-	-	Pharyngeal swab at any time	-	CSF	Postpartum: Indeterminate N=1	
**Gonzalez R (1), 2021**	Case report	1 mother with confirmed COVID-19 (RT-PCR)	1	-	-	-	-	Blood IgM Cord blood IgM	Blood IgG Cord blood IgG	In Utero: Possible N = 1	-
**Hinojosa-Velasco A, 2020 Mexico***	Case report	1 mother with confirmed COVID-19 (RT-PCR)	1	-	-	Stool Breast milk	Pharyngeal swab at birth Pharyngeal swab at any time Stool	-	-	In Utero: Indeterminate N = 1	-
**Lima ARO, 2020 Brazil**	Case report	1 mother with possible COVID-19 (clinical diagnosis)	1	-	-	Amniotic fluid Placenta (any side)	Pharyngeal swab at any time Blood Chorion	Neonatal blood and cord blood IgM	Neonatal blood IgG Maternal blood IgM and IgG Cord blood IgG	In Utero: Confirmed N = 1	-
**Lima-Rogel V, 2020 Mexico**	Case report	2 mothers with confirmed COVID-19 (RT-PCR)	2	-	-	-	Pharyngeal swab at any time	-	-	Not classifiable N = 2	-
**Machado S, 2021 Brazil**	Case report	1 mother with confirmed COVID-19 (RT-PCR)	1	-	-	Placenta (any side)	Pharyngeal swab at any time	-	-	In Utero (IUFD): Possible N = 1	Foetal death (IUFD)
**Mendoza-Hernandez M, 2021 Mexico**	Case report	1 mother with confirmed COVID-19 (RT-PCR)	1	-	-	Placenta (any side)	Pharyngeal swab at any time	-	-	In Utero: Indeterminate N = 1	-
**Olcese LC, 2020 Peru**	Case report	1 mother with probable COVID-19 (serology)	1	-	-	-	Pharyngeal swab at any time	Neonatal blood IgM	Neonatal blood IgG	-	-

**Orostizaga AA, 2020 Chile**	Case report	1 mother with confirmed COVID-19 (RT-PCR)	1	-	-	-	Pharyngeal swab at any time	-	-	-	-
**Pessoa FS, 2020 Brazil**	Case report	1 mother with confirmed COVID-19 (RT-PCR)	1	-	-	-	Pharyngeal swab at any time	-	-	In Utero: Indeterminate N = 1	-
**Rebello CM, 2020 Brazil**	Case report	1 mother with confirmed COVID-19 (RT-PCR)	1	-	-	-	Pharyngeal swab at any time<br>Cord blood<br>Neonatal skin swab	Neonatal blood IgM	Neonatal blood IgG	In Utero: Unlikely N = 1	-
**Rios DI, 2020 Mexico***	Case report	1 mother with confirmed COVID-19 (RT-PCR)	1	-	-	Placenta (any side)	Pharyngeal swab at any time	-	-	Not classifiable N = 1	-
**Salinas DL, 2021 Mexico**	Case report	1 mother with confirmed COVID-19 (RT-PCR)	1	-	-	-	Pharyngeal swab at any time	-	-	In Utero: Indeterminate N = 1	-
**Sanchez J, 2021 Panama**	Case report	2 mothers with confirmed COVID-19 (RT-PCR)	2	-	-	Placenta (any side)<br>Placenta (baby side)	Pharyngeal swab at any time	-	IHC placenta	In Utero: Indeterminate N = 2	-
**Stonoga ETS, 2020 Brazil**	Case report	1 mother with confirmed COVID-19 (RT-PCR)	1	-	-	Placenta (any side)	Cord blood	-	-	In Utero (IUFD): Unlikely N = 1	1 foetal death (stillbirth)
**Tutiya CT, 2020 Brazil**	Case report	2 mothers with confirmed COVID-19 (RT-PCR)	2	-	-	-	Pharyngeal swab at any time	-	-	-	1 neonatal death
**Valdespino-Vazquez MY, 2021 Mexico**	Case report	1 mother with confirmed COVID-19 (RT-PCR)	2	-	-	Placenta (any side)	Foetal organs	-	Placenta (EM, immunoflu orescence)	In Utero (IUFD): Confirmed N = 2	2 foetal deaths
**Wolf Lebrao C, 2020 Brazil**	Case report	1 mother with probable COVID-19 (serology)	1	-	-	Breast milk<br>Blood IgG and IgM	Pharyngeal swab at any time	-	-	-	
**Middle East and North Africa**											
**Abdulghani SH, 2021 Saudi Arabia**	Retrospecti ve cohort	62 mothers with confirmed	62	All pregnant women with COVID-19 from	Mode of delivery,	-	Pharyngeal swab at any time	Neonatal blood IgM	-	In Utero: Unlikely N=1	

		COVID-19 (RT-PCR)		March to May 2020, and their neonates	preterm birth, breast feeding						
**Abedzadeh-Kalahroudi M, 2021 Iran**	Prospective cohort	56 mothers with confirmed COVID-19 (RT-PCR)	56	Pregnant women diagnosed with COVID-19 and their neonates between March to November 2020	-	-	Pharyngeal swab at any time	-	-	In utero: Indeterminate N = 2	No maternal deaths 1 foetal death (abortion) 2 neonatal deaths
**Al-Matary, A 2021 Saudi Arabia**	Retrospective cohort	288 mothers with confirmed COVID-19 (RT-PCR)	200	Pregnant women with COVID-19 and their neonates between March to November 2020	-	-	Pharyngeal swab at any time	-	-	-	1 maternal death 4 neonatal deaths
**Alnashry LM, 2021 Saudi Arabia**	Retrospective cohort	20 mothers with confirmed COVID-19 (RT-PCR)	20	All pregnant women with COVID-19 and their neonates, from March to December 2020	-	-	Pharyngeal swab at any time	-	-	-	-
**Askary E, 2020 Iran**	Cohort	16 mothers with confirmed COVID-19 (RT-PCR or clinical and radiological diagnosis)	12	Pregnant women with confirmed COVID-19 from March 21 to May 11 2020, and their neonates	-	-	Pharyngeal swab at any time	-	-	-	-
**Ayed A, 2020 Kuwait**	Retrospective cohort	185 mothers with confirmed COVID-19 (RT-PCR)	41	All pregnant women with COVID-19 and their neonates between March 15 and May 31, 2020	ICU admission, mode of delivery, trimester of infection, pregnancy status, maternal severity, preterm birth	-	Pharyngeal swab at any time	-	-	Postpartum: Unlikely N = 1 Postpartum: Possible N = 1	No maternal deaths 4 foetal deaths (1 IUFD, 3 miscarriages)

**Barber E, 2021 Israel**	Prospective cohort	51 mothers with confirmed COVID-19 (RT-PCR)	13	All pregnant women with COVID-19 and their neonates between March and October 2020	-	Vaginal fluid	Pharyngeal swab at any time	-	-	-	-
**Beharier O, 2021 Israel**	Cohort	94 mothers with confirmed COVID-19 (RT-PCR)	94 (65 underwent testing)	Pregnant women with COVID-19 admitted for delivery, and their neonates between from April 2020	-	-	-	Cord blood IgM	Cord blood IgG	In Utero: Indeterminate N = 4	-
**Chaichian S, 2021 Iran**	Cohort	14 mothers: 4 with confirmed COVID-19 (RT-PCR) and 10 with possible COVID-19 (clinical diagnosis)	13	All pregnant women with COVID-19 and their neonates, between March 8 to December 28, 2020	-	-	Pharyngeal swab at any time	-	-	-	-
**ElHalik M, 2020 United Arab Emirates***	Retrospective cohort	35 mothers with confirmed COVID-19 (RT-PCR)	36	Neonates born to mothers who tested positive for SARS-CoV-2 infection between 1 March and 15 August 2020	-	-	Pharyngeal swab at any birth Pharyngeal swab at any time	-	-	In Utero: Possible N = 1 Not classifiable N = 1	1 maternal death 1 neonatal death
**Farhat AS, 2020 Iran***	Prospective cohort	25 mothers: 20 with confirmed COVID-19 (RT-PCR) and 5 with possible COVID-19 (clinical diagnosis)	25 (18 underwent testing)	Mothers suspicious of COVID-19 infection and their neonates admitted between March 15 and April 15, 2020	Mode of delivery, trimester of infection, ICU admission, pregnancy status, maternal severity, preterm birth	-	Pharyngeal swab at any time or tracheal tube sample	-	-	Not classifiable N = 2	-

**Ghema K, 2021 Morocco**	Cohort	30 mothers: 29 with confirmed COVID-19 (RT-PCR), 1 with possible COVID-19 infection (radiological diagnosis)	30	All neonates born to mothers with COVID-19, between January to December 2020	Mode of delivery	-	Pharyngeal swab at any time	-	-	In Utero: Indeterminate N = 1 Postpartum: Indeterminate N = 1	1 maternal death 2 neonatal deaths
**Hadar E, 2021 Israel**	Retrospective cohort	147 mothers with confirmed COVID-19 (RT-PCR)	144 (59 underwent testing)	Pregnant women with COVID-19 and their neonates from April 4 2020 March 8 2021	-	-	Pharyngeal swab at any time	-	-	In Utero: Indeterminate N=6	-
**Hazari K, 2020 United Arab Emirates***	Retrospective cohort	79 mothers with confirmed COVID-19 (RT-PCR)	31	Non-pregnant and pregnant women (and their neonates) with COVID-19 between March and June 2020	Mode of delivery	-	Pharyngeal swab at birth Pharyngeal swab at any time	-	-	In Utero: Unlikely N=1 Not classifiable N = 1	1 maternal death 5 foetal deaths (4 miscarriages, 1 IUFD)
**Hosseini MS, 2021**	Retrospective cohort	13 mothers with confirmed COVID-19 (RT-PCR)	42	Pregnant women with COVID-19 and their neonates between February 21 to November 30 2020	Mode of delivery, preterm birth, maternal death	-	Pharyngeal swab at any time	-	-	In Utero: Indeterminate N = 1	2 maternal death 2 neonatal deaths
**Kamali A, 2021 Iran**	Cohort	13 mothers with confirmed COVID-19 (RT-PCR)	6	Symptomatic pregnant women with COVID-19 admitted from 15 February to 15 June 2020, and their neonates	-	Amniotic fluid	Pharyngeal swab at any time Cord blood	-	-	-	-
**Lopian M, 2020 Israel**	Cohort	21 mothers with confirmed COVID-19 (RT-PCR)	21	Women with laboratory-confirmed COVID-19 who delivered between March 23 and May 8 2020, and their neonates	Mode of delivery, preterm birth	-	Pharyngeal swab at any time	-	-	In Utero: Possible N = 1	-
**Mohaghegh Z, 2021 Iran***	Cohort	263 mothers with confirmed COVID-19 (RT-PCR)	270 (183 underwent testing)	Pregnant women with COVID-19 and their neonates between February to July 2020	-	-	Pharyngeal swab at any time	-	-	-	3 maternal deaths 2 foetal deaths 7 neonatal deaths
			10		-	-		-	-	-	-

**Omrani AS, 2020 Qatar***	Retrospective cohort	26 mothers with confirmed COVID-19 (RT-PCR)		The first consecutive 5000 patients with COVID-19 in Qatar, including pregnant women and their neonates			Pharyngeal swab at any time				
**Rosen H, 2021 Israel**	Prospective cohort	55 mothers with confirmed COVID-19 (RT-PCR)	29 (4 underwent testing)	Pregnant women with laboratory confirmed SARS-CoV-2 infection prior to 26 weeks of gestation, and their neonates between March 2020 to February 2021	-	Amniotic fluid	Blood PCR	Blood IgM<br>Cord blood IgM	Cord blood IgG	-	-
**Santhosh J, 2020 Oman**	Retrospective cohort	60 mothers with confirmed COVID-19 (RT-PCR)	48 (4 underwent testing)	Pregnant and postpartum women from March 24 to July 31 2020, with confirmed SARS-CoV-2 and their neonates	-	-	Pharyngeal swab at any time	-	-	-	No maternal deaths<br>5 foetal deaths (4 miscarriages, 1 stillbirth)
**Sattari M, 2020 Iran**	Retrospective cohort	50 mothers with confirmed COVID-19 (RT-PCR)	25 (7 underwent testing)	Hospitalised pregnant women diagnosed with COVID-19 in 2020 and their neonates	-	-	Pharyngeal swab at any time	-	-	Not classifiable<br>N = 7	2 maternal deaths<br>No foetal/neonatal deaths
**Schwartz DA (3), 2020 Iran***	Retrospective cohort	14 mothers: 9 with confirmed COVID-19 (RT-PCR) and 5 with possible COVID-19 (clinical or radiological diagnosis)	14	Neonates with COVID-19 and their mothers confirmed or suspected with COVID-19	Preterm birth	Amniotic fluid<br>Vaginal fluid	Pharyngeal swab at any time	-	-	In Utero: Indeterminate<br>N = 5<br>In Utero: Possible<br>N = 1<br>Postpartum: Possible<br>N = 1<br>Not classifiable<br>N = 5	1 maternal death<br>4 neonatal deaths
**Vaezi M, 2021 Iran**	Cohort	24: 21 mothers with confirmed COVID-19 (RT-PCR), 3 mothers with	21	All pregnant women with COVID-19 and their neonates from March 10 to April 15, 2020	-	-	Pharyngeal swab at any time	-	-	-	1 foetal death

		possible COVID-19 (radiological or clinical diagnosis)									
**Vizheh M, 2021 Iran***	Cohort	110 mothers with confirmed COVID-19 (RT-PCR)	51	Pregnant women with COVID-19 and their neonates between March and October 2020	-	-	Pharyngeal swab at any time	-	-	Not classifiable N=4	6 maternal deaths<br>8 spontaneous abortions<br>2 neonatal deaths
**Akbarian-Rad Z, 2021 Iran**	Case series	11 mothers: 6 with confirmed COVID-19 (RT-PCR), 5 with possible diagnosis (radiological or clinical diagnosis)	11 (8 underwent testing)	-	-	Amniotic fluid	Pharyngeal swab at any time	-	-	-	-
**Alsharaydeh I, 2020 Jordan**	Case series	4 mothers with confirmed COVID-19 (RT-PCR)	2	-	-	Amniotic fluid	Pharyngeal swab at any time	-	-	-	-
**Alwardi TH, 2020 Oman**	Case series	1 mother with confirmed COVID-19 (RT-PCR)	3	-	-	-	Pharyngeal swab at any time	-	-	In Utero: Possible N = 3	-
**Aouali K, 2021 Morocco**	Case series	3 mothers with confirmed COVID-19 (RT-PCR)	3	-	-	-	Pharyngeal swab at any time	-	-	-	-
**Hantoushzadeh S, 2020 Iran**	Case series	9 mothers with confirmed COVID-19 (RT-PCR)	11 (7 underwent testing)	-	-	-	Pharyngeal swab at any time	-	-	Not classifiable N = 1	7 maternal deaths<br>4 foetal deaths
**Moeindarbary S, 2020 Iran***	Case series	2 mothers with confirmed COVID-19 (RT-PCR)	2	-	-	-	Pharyngeal swab at any time	-	-	In Utero: Indeterminate N = 1<br>Not classifiable N = 1	No maternal deaths<br>1 neonatal death
**Mrazguia C, 2020 Tunisia**	Case series	11 mothers with confirmed COVID-19 (RT-PCR)	6 (2 underwent testing)	-	-	-	Pharyngeal swab at any time	-	-	-	-

**Parsa Y, 2020 Iran**	Case series	9 mothers with confirmed COVID-19 (RT-PCR)	9	-	-	Amniotic fluid	Pharyngeal swab at any time	-	-	In Utero: Indeterminate N = 1	-
**Schwartz A, 2020 Israel**	Case series	5 mothers with confirmed COVID-19 (RT-PCR)	5 (0 underwent testing)	-	-	Vaginal fluid	-	-	-	-	-
**Sheikhahmadi S, 2021 Iran**	Case series	8 mothers with confirmed COVID-19 (RT-PCR)	8	-	-	-	Pharyngeal swab at any time	-	-	In Utero: Indeterminate N=3	No maternal death<br>2 neonatal deaths
**Tabatabaei S, 2021 Iran**	Case series	2 mothers: 1 with confirmed COVID-19 (RT-PCR) and 1 with possible COVID-19 (clinical diagnosis)	2	-	-	-	Pharyngeal swab at any time	-	-	Not classifiable N = 2	1 maternal death<br>No foetal/neonatal deaths
**Abourida Y, 2020 Morocco**	Case report	1 mother with possible COVID-19 (RT-PCR)	1	-	-	-	Pharyngeal swab at any time	-	-	-	1 neonatal death
**Aghdam M, 2020 Iran**	Case report	1 mother with possible COVID-19 (RT-PCR)	1	-	-	-	Pharyngeal swab at any time	-	-	Not classifiable N = 1	-
**Algadeeb KB, 2020 Saudi Arabia**	Case report	1 mother with confirmed COVID-19 (RT-PCR)	1	-	-	-	Pharyngeal swab at any time	-	-	Postpartum: Possible N = 1	1 neonatal death
**Al-Kuraishy HM 2020, Iraq**	Case report	1 mother with confirmed COVID-19 (RT-PCR)	1	-	-	-	Pharyngeal swab at any time	-	-	-	-
**AlZaghal LA, 2020 Jordan**	Case report	1 mother with confirmed COVID-19 (RT-PCR)	1	-	-	Breast milk	Pharyngeal swab at birth<br>Pharyngeal swab at any time	-	-	-	-

**Amiraskari R, 2020 Iran**	Case report	1 mother with confirmed COVID-19 (RT-PCR)	1	-	-	-	Pharyngeal swab at any time	-	-	Postpartum: Indeterminate N = 1	No foetal/neonatal death
**Bordbar A, 2020 Iran**	Case report	1 mother with confirmed COVID-19 (RT-PCR)	1	-	-	-	Pharyngeal swab at any time	-	-	Not classifiable N = 1	-
**Choobdar FA, 2020 Iran**	Case report	1 mother with confirmed COVID-19 (RT-PCR)	1	-	-	Placenta (any side) Placenta (baby side)	Pharyngeal swab at any time	Blood IgM	Blood IgG	In Utero: Possible N=1	-
**Dorgalaleh A, 2020 Iran**	Case report	1 mother with confirmed COVID-19 (RT-PCR)	1	-	-	-	Pharyngeal swab at any time	-	-	Not classifiable N = 1	-
**Eghbalian F, 2020 Iran***	Case report	1 mother with possible COVID-19 (RT-PCR)	1	-	-	-	Pharyngeal swab at any time	-	-	Not classifiable N = 1	-
**Farhadi R, 2021 Iran**	Case report	1 mother with confirmed COVID-19 (RT-PCR)	1	-	-	Amniotic fluid	Pharyngeal swab at any time Cord blood PCR	-	-	In Utero: Possible N=1	1 maternal death No neonatal death
**Farhat A, 2020 Iran**	Case report	1 mother with possible COVID-19 (clinical diagnosis)	1	-	-	-	Pharyngeal swab at any time	-	-	In utero: Indeterminate N = 1	1 neonatal death
**Huseynova RA, 2021 Saudi Arabia**	Case report	1 mother with confirmed COVID-19 (RT-PCR)	1	-	-	-	Pharyngeal swab at any time	-	-	In Utero: Unlikely N = 1	-
**Jafari R, 2020 Iran**	Case report	1 mother with confirmed COVID-19 (RT-PCR)	2	-	-	-	Pharyngeal swab at any time	-	-	-	-
**Khatib MY 2021, Qatar***	Case report	1 mother with confirmed COVID-19 (RT-PCR)	1	-	-	-	Pharyngeal swab at any time	-	-	-	-
**Khorsandi B, 2021 Iran**	Case report	1 mother with possible COVID-19 (radiological or clinical diagnosis)	1	-	-	-	Pharyngeal swab at any time	-	-	-	-

**Mirahmadizadeh A, 2020 Iran**	Case report	1 mother with confirmed COVID-19 (RT-PCR)	1	-	-	-	Pharyngeal swab at any time	-	-	Not classifiable N = 1	-
**Naseh A, 2021 Iran**	Case report	1 mother with confirmed COVID-19 (RT-PCR)	1	-	-	-	Pharyngeal swab at any time	-	-	In Utero: Indeterminate N=1	-
**Rashan N, 2020 Iran**	Case report	1 mother with confirmed COVID-19 (RT-PCR)	1	-	-	-	Pharyngeal swab at any time	-	-	-	-
**Rashidan T, 2020 Iran**	Case report	1 mother with confirmed COVID-19 (RT-PCR)	1	-	-	-	Pharyngeal swab at any time	-	-	-	1 neonatal death
**Sagheb S, 2020 Iran**	Case report	2 mothers: 1 with confirmed COVID-19 (RT-PCR) and 1 with possible COVID-19 (radiological diagnosis)	2	-	-	-	Pharyngeal swab at any time	-	-	Not classifiable N = 2	1 maternal death
**Shaiba LA, 2021 Saudi Arabia**	Case report	2 mothers: 1 with confirmed COVID-19 (RT-PCR)	2	-	-	-	Pharyngeal swab at any time	Blood IgM	-	In Utero: Possible N = 1	-
**Taghizadieh A, 2020 Iran**	Case report	1 mother with confirmed COVID-19 (RT-PCR)	1	-	-	-	Pharyngeal swab at any time	-	-	-	-
**Yaqoub S, 2020 Qatar***	Case report	1 mother with confirmed COVID-19 (RT-PCR)	1	-	-	-	Pharyngeal swab at any time	Blood IgM	Blood IgG	-	-
**North America**											
**Adhikari EH, 2020 USA***	Cohort	252 mothers with confirmed COVID-19 (RT-PCR)	252 (188 underwent testing)	Pregnant women who were tested for SARS-CoV-2 during pregnancy between March 18 and August 22 2020, and their neonates	-	-	Pharyngeal swab at any time	-	-	In Utero: Possible N = 3 In Utero: Indeterminate N = 1 In Utero: Unlikely N = 2	No maternal deaths 7 foetal deaths (abortion)

**Angelidou A, 2021 USA**	Cohort	250 mothers with confirmed COVID-19 (RT-PCR)	255 (225 underwent testing)	Pregnant women with COVID-19 and their neonates, between May 17 to September 4 2021	-	-	Pharyngeal swab at any time	-	-	In Utero: Possible N = 3<br>In Utero: Indeterminate N = 2<br>Postpartum: Indeterminate N=1	-
**Argueta LB, 2021 USA**	Cohort	55 mothers with confirmed COVID-19 (RT-PCR)	57	Pregnant women with COVID-19 and their neonates, between May 17 to September 4 2021	-	Placenta tissue (any side)	Pharyngeal swab at any time	-	Placenta RT-PCR, IHC, ISH	In Utero: Indeterminate N = 23	-
**Bender WR (1), 2020 USA**	Retrospective cohort	8 mothers with confirmed COVID-19 (RT-PCR)	6	Pregnant women with COVID-19 between April 13 and April 26, 2020 and their neonates	-	-	Pharyngeal swab at any time	-	-	-	No maternal deaths<br>1 foetal death (IUFD)
**Berry M, 2021 USA**	Retrospective cohort	91 mothers with confirmed COVID-19 (RT-PCR)	61	Pregnant women with COVID-19 and their neonates between March and July 2020	-	-	Pharyngeal swab at any time	-	-	-	No maternal deaths<br>1 foetal death (IUFD)
**Brandt JS, 2020 USA**	Case control	61 mothers with confirmed COVID-19 (RT-PCR)	61	Pregnant women with COVID-19 who delivered between 16 and 41 weeks' gestation, and their neonates from March 11 to June 11 2020	-	-	Pharyngeal swab at any time	-	-	-	1 neonatal death
**Buhimschi CS, 2020 USA**	Cohort	29 mothers with confirmed COVID-19 (RT-PCR)	20	Pregnant women with COVID-19 and their neonates from April 16 to June 16 2020	-	-	Pharyngeal swab at any time	-	-	-	-
**Cojocaru L, 2020 USA**	Prospective cohort	86 mothers with confirmed COVID-19 (RT-PCR)	31	Pregnant women who were tested for SARS-CoV-2 between March and June 2020, and their neonates	-	-	Pharyngeal swab at any time	-	-	-	-
**Dhuyvetter A, 2020 USA**	Cohort	23 mothers with confirmed COVID-19 (RT-PCR)	23	All pregnant women tested for COVID-19 between April 12 and	-	-	Pharyngeal swab at any time	-	-	-	-

				July 11 2020, and their neonates							
**Dumitriu D, 2020 USA**	Retrospective cohort	100 mothers: 99 with confirmed COVID-19 (RT-PCR) and 1 with possible COVID-19 (clinical and radiological diagnosis)	101	Newborns born to mothers positive for or with suspected SARS-CoV-2 infection between March 13 and April 24 2020	-	-	Pharyngeal swab at any time	-	-	-	-
**Farghaly MAA, 2020 USA**	Retrospective cohort	15 mothers with confirmed COVID-19 (RT-PCR)	15	-	Mode of delivery, preterm birth	-	Pharyngeal swab at any time	Neonatal blood IgM	Neonatal blood IgG	In Utero; Indeterminate N = 1	-
**Flannery DD (1), 2020 USA**	Prospective cohort	83 mothers: 44 with confirmed COVID-19 (RT-PCR) and 39 with probable COVID-19 (serology)	83	Women with COVID-19 and their neonates between April 9 and August 8 2020	-	-	Pharyngeal swab at any time	Cord blood IgM	Neonatal blood IgG Cord blood IgG	-	-
**Grechukina O, 2020 USA***	Retrospective cohort	141 mothers with confirmed COVID-19 (RT-PCR)	73 (60 underwent testing)	Pregnant and postpartum women with COVID-19 between 3 January and 5 November 2020, and their neonates	-	Placenta (any side)	Pharyngeal swab at any time	-	-	-	1 maternal death
**Griffin I, 2020 USA**	Prospective cohort	27 mothers: 25 with confirmed COVID-19 (RT-PCR) and 2 with possible COVID-19 (clinical diagnosis)	25 (15 underwent testing)	Mothers with COVID-19 and their neonates admitted between April 21 and May 5, 2020	-	-	Pharyngeal swab at any time	-	-	-	-
**Gulersen M (1), 2020 USA**	Retrospective cohort	50 mothers with confirmed COVID-19 (RT-PCR)	50	Placentas from all women with COVID-19 and their neonates from April 9 and April 27, 2020	-	-	Pharyngeal swab at any time	-	-	-	-
**Hecht J, 2020 USA***	Retrospective cohort	20 mothers: 19 with confirmed COVID-19 (RT-PCR) and 1 with possible COVID-19	22 (20 underwent testing)	Pregnant women with COVID-19 and their neonates captured from March 1 to May 15, 2020	Mode of delivery, preterm birth, pregnancy status, maternal	-	Pharyngeal swab at any time	-	Placenta IHC and ISH	-	-

		(clinical diagnosis)			severity, ICU admission						
**Jani S, 2021 USA**	Retrospective cohort	34 mothers with confirmed COVID-19 (RT-PCR)	34 (31 underwent testing)	COVID-19 positive women and their neonates between March 11 and July 31 2020	-	-	Pharyngeal swab at any time	-	-	-	-
**Janssen O, 2020 USA**	Retrospective cohort	180 mothers with confirmed COVID-19 (RT-PCR)	180	Pregnant women who tested positive for SARS-CoV-2 infection between March 25 and May 15 2020, and their neonates	-	-	Pharyngeal swab at any time	-	-	Not classifiable N = 3	No maternal deaths 14 foetal deaths (IUFD)
**Joseph NT (3), 2021 USA**	Prospective cohort	32 mothers with confirmed COVID-19 (RT-PCR)	32	Women who tested positive for COVID-19 who delivered between April and December 2020	-	Trimester of infection, mode of delivery	- Pharyngeal swab at any time	Cord blood IgM	Cord blood IgG	In Utero: Indeterminate N = 3	-
**Kest H, 2020 USA**	Retrospective cohort	49 mothers with confirmed COVID-19 (RT-PCR)	49	Neonates born to SARS-CoV-2 positive mothers at >36 weeks gestational age between May 1 and June 12 2020	-	-	Pharyngeal swab at any time	-	-	In Utero: Unlikely N = 1	-
**Khoury R, 2020 USA***	Prospective cohort	241 mothers with confirmed COVID-19 (RT-PCR)	247 (236 underwent testing)	Pregnant women with COVID-19 and their neonates between March 13 and April 12, 2020	Mode of delivery, ICU admission, maternal severity, preterm birth	-	Pharyngeal swab at any time	-	-	In Utero: Indeterminate N = 6	No maternal death 2 foetal deaths (IUFD)
**Lang LK, 2020 USA**	Cohort	1120 mothers with confirmed COVID-19 (RT-PCR)	257	Pregnant women diagnosed with COVID-19 up to August 8 2020 and their neonates	-	-	Pharyngeal swab at any time	-	-	-	-
**Levitan D, 2021 USA**	Cohort	65 mothers with confirmed COVID-19 (RT-PCR)	65	Placentas from all women with COVID-19 and their neonates between March 25 and May 4 2020	-	-	Pharyngeal swab at any time	-	-	-	-
**Lokken EM (1), 2021 USA**	Cohort	240 mothers with confirmed COVID-19 (RT-PCR)	156 (45 underwent testing)	Pregnant patients with confirmed SARS-CoV-2 infection between March 1 and June 30 2020, and their neonates	-	-	Pharyngeal swab at any time	-	-	-	3 maternal deaths 4 foetal deaths (2 stillbirths and 2

											spontaneous abortions)
**Mourad M (1) 2021**	Cohort	66 mothers with confirmed COVID-19 (RT-PCR)	66	Pregnant women with COVID-19 with biospecimens available, and their neonates	Maternal severity	Placenta (any side)	Pharyngeal swab at any time	Cord blood IgM	Cord blood IgG	In Utero: Indeterminate N = 2	-
**Ogamba I, 2020 USA**	Retrospective cohort	40 mothers with confirmed COVID-19 (RT-PCR)	23 (20 underwent testing)	Pregnant women, 18 years or older, with COVID-19 between March 17 and June 4 2020, and their neonates	-	-	Pharyngeal swab at any time	-	-	-	No maternal deaths<br>2 foetal deaths (miscarriage)
**Oxana Z, 2021 USA**	Retrospective cohort	10 mothers with confirmed COVID-19 (RT-PCR)	10 (7 underwent testing)	SARS-CoV-2 positive pregnant women from March to August 2020, and their neonates	-	-	Pharyngeal swab at any time	-	-	-	-
**Patberg ET, 2020 USA**	Retrospective cohort	77 mothers with confirmed COVID-19 (RT-PCR)	77 (71 underwent testing)	Pregnant women diagnosed with COVID-19 between 31 march and 17 June 2020, and their neonates<br><br>Exclusion criteria: delivery at <37 weeks gestation	-	-	Pharyngeal swab at any time	-	-	-	-
**Penfield C, 2020 USA***	Prospective cohort	32 mothers with confirmed COVID-19 (RT-PCR)	32 (11 underwent testing)	Pregnant women diagnosed with COVID-19 and their neonates between March 1 and April 20, 2020	-	Placenta (baby side)<br>Amniotic membranes	Pharyngeal swab at any time	-	-	-	-
**Pineles BL, 2020 USA**	Retrospective cohort	77 mothers with confirmed COVID-19 (RT-PCR)	71	Pregnant women at ≥ 20 weeks of gestation with COVID-19 between April 22 and July 22, 2020 and their neonates	Mode of delivery, ICU admission, preterm birth	-	Pharyngeal swab at any time	-	-	In Utero: Unlikely N = 1	-
**Qadri F, 2020 USA**	Prospective cohort	16 mothers with confirmed COVID-19 (RT-PCR)	16 (12 underwent testing)	Pregnant women with COVID-19 and their neonates between March 26 to April 10, 2020	-	-	Pharyngeal swab at any time	-	-	-	-

**Romagano MP, 2020 USA**	Retrospective cohort	8 mothers with confirmed COVID-19 (RT-PCR)	8 (7 underwent testing)	Mothers and their newborns requiring critical care for severe COVID-19 between March and April 2020	-	-	Pharyngeal swab at any time	-	-	-	-
**Salvatore CM, 2020 USA**	Retrospective cohort	116 mothers with confirmed COVID-19 (RT-PCR)	120	All neonates born to mothers with COVID-19 between March 22 and May 17, 2020	-	-	Pharyngeal swab at birth<br>Pharyngeal swab at any time	-	-	-	-
**Sastry SR, 2020 USA**	Case series from prospective cohort	5 mothers with confirmed COVID-19 (RT-PCR)	1	Patients admitted between April 27 and May 18 2020 (includes pregnant women and their neonates)	-	-	Pharyngeal swab at any time	-	-	Not classifiable N = 1	-
**Shook (1) LL, 2021 USA***	Cohort	354 mothers with confirmed COVID-19 (RT-PCR)	369 (159 underwent testing)	Pregnant women with COVID-19 and their neonates between March 22 and December 20, 2020	-	-	Pharyngeal swab at any time	-	-	-	-
**Song D, 2021 USA**	Prospective cohort	145 mothers with confirmed COVID-19 (RT-PCR)	147 (144 underwent testing)	Pregnant women with COVID-19 and their neonates between April 15 2020 and March 31, 2020	-	Placenta (any side)	Pharyngeal swab at any time<br>Cord blood PCR	Neonatal blood IgM<br>Cord blood IgM	Neonatal blood IgG<br>Cord blood IgG<br>Neonatal stool	In Utero: Possible N=1<br>In Utero: indeterminate N = 3<br>Intrapartum: indeterminate N=2	-
**Steffen HA, 2021 USA**	Prospective cohort	61 mothers: 23 with confirmed COVID-19 infection (RT-PCR), 38 with probable COVID-19 (serology)	65 (17 underwent testing)	All women with COVID-19 and their neonates, between May 1 and September 22 2020	-	-	Pharyngeal swab at any time	-	-	-	-
**Zgutka K, 2021 USA**	Case-control	60 mothers with confirmed COVID-19 (RT-PCR)	62	Neonates born to mothers with COVID-19 between March 15 and June 15, 2020<br><br>Controls: neonates born to non-COVID-19 mothers	-	-	Pharyngeal swab at any time	-	-	-	-
**Zhang P, 2020 USA**	Retrospective cohort	74 mothers with confirmed	74	Placentas from mothers tested for	-	-	Pharyngeal swab at any time	-	In situ hybridizatio	In Utero: Possible N = 1	-

		COVID-19 (RT-PCR)		COVID-19, and their neonates					n of placenta	In Utero: indeterminate N = 1	
**Zlochiver V, 2021 USA**	Cohort	85 mothers with confirmed COVID-19 (RT-PCR)	92	Pregnant women with COVID-19 who delivered, and their neonates from March 23 to October 3 2020	-	-	Pharyngeal swab at any time	-	-	-	-
**Douedi S, 2020**	Case series	3 mothers with confirmed COVID-19 (RT-PCR)	3	-	-	-	Pharyngeal swab at any time	-	-	-	-
**Fashner J, 2020 USA**	Case series	7 mothers with confirmed COVID-19 (RT-PCR)	7	-	-	-	Pharyngeal swab at any time	-	-	-	No maternal deaths 2 neonatal deaths
**McCoy JA, 2020**	Case series	5 mothers with confirmed COVID-19 (RT-PCR)	5 (4 underwent testing)	-	-	-	Pharyngeal swab at any time	-	-	-	-
**Mei-Dan E 2020, Canada**	Case series	4 mothers with confirmed COVID-19 (RT-PCR)	4	-	-	-	Pharyngeal swab at any time	-	-	-	-
**Reddy A, 2020 USA**	Case series	2 mothers with confirmed COVID-19 (RT-PCR)	2	-	-	-	Pharyngeal swab at any time	-	-	-	-
**Shanes ED, 2020 2020**	Case series	16 mothers with confirmed COVID-19 (RT-PCR)	2	-	-	-	Pharyngeal swab at any time	-	-	-	-
**Valk JE, 2020 USA**	Case series	2 mothers with confirmed COVID-19 (RT-PCR)	2	-	-	Placenta (any side)	Pharyngeal swab at any time Cord Foetal lungs, heart, liver, kidneys, intestines, spleen and adrenal	-	Placenta IHC and ISH	In Utero (IUFD): Unlikely N = 2	No maternal deaths 1 neonatal death

**Verma S, 2021 USA**	Case series	5 mothers with confirmed COVID-19 (RT-PCR)	5	-	-	Placenta (any side)	-	-	Placenta IHC and ISH	In Utero: Indeterminate N = 5	-
**Wardell H, 2020 USA**	Case series	2 mothers with possible COVID-19 (RT-PCR)	2	-	-	-	Pharyngeal swab at any time	-	-	Not classifiable N = 2	-
**Algarroba G, 2020 USA**	Case report	1 mother with confirmed COVID-19 (RT-PCR)	1	-	-	-	Pharyngeal swab at any time	-	Electron microscopy of placenta	In Utero: Unlikely N = 1	-
**Chambers CD, 2020 USA**	Case report from prospective cohort	2 mothers with possible COVID-19 (clinical diagnosis)	2 (1 underwent testing)	Mothers recruited for the Mommy's Milk Human Milk Research Biorepository	-	Breast milk	Pharyngeal swab at any time	-	Breast milk viral culture	Not classifiable N = 1	-
**Clough BM, 2021 USA**	Case report	1 mother with confirmed COVID-19 (RT-PCR)	1	-	-	-	Pharyngeal swab at any time	-	-	-	-
**Elkafrawi D, 2020**	Case report	1 mother with confirmed COVID-19 (RT-PCR)	1	-	-	-	Pharyngeal swab at birth	-	-	-	-
**Fang NZ, 2020 USA**	Case report	1 mother with confirmed COVID-19 (RT-PCR)	1	-	-	Placenta (any side)	-	-	-	-	1 foetal death (abortion)
**Gonzalez MR, 2020**	Case report	1 mother with confirmed COVID-19 (RT-PCR)	1	-	-	Amniotic fluid Breast milk	Pharyngeal swab at any time	-	-	-	-
**Grundman JB, 2021 USA**	Case report	1 mother with confirmed COVID-19 (RT-PCR)	1	-	-	-	Pharyngeal swab at any time	-	-	-	-
**Gulersen M (2), 2020 USA**	Case report	1 mother with confirmed COVID-19 (RT-PCR)	1	-	-	-	Pharyngeal swab at any time	-	-	-	-
**Gupta A, 2020 USA**	Case report	1 mother with confirmed COVID-19 (RT-PCR)	1	-	-	-	Pharyngeal swab at any time	Neonatal blood IgM	Neonatal blood IgG	In Utero; Possible N = 1	-

**Hansen JN, 2020**	Case report	1 mother with confirmed COVID-19 (RT-PCR)	1	-	-	-	Pharyngeal swab at any time	-	-	-	-
**Hansra R, 2020 USA**	Case report	1 mother with confirmed COVID-19 (RT-PCR)	2	-	-	-	Pharyngeal swab at any time	-	-	-	-
**Hopwood AJ, 2020 USA**	Case report	1 mother with confirmed COVID-19 (RT-PCR)	1	-	-	-	Pharyngeal swab at any time	-	Neonatal blood IgG	In Utero: Indeterminate N = 1	-
**Hosier H, 2020 USA***	Case report	1 mother with confirmed COVID-19 (RT-PCR)	1	-	-	Placenta (any side) Saliva Urine Umbilical cord	Foetal tissues	-	Maternal blood IgM and IgG Placenta (IHC, ISH and EM)	In Utero (IUFD): Unlikely N = 1	-
**Hsu AL, 2020 USA**	Case report	1 mother with confirmed COVID-19 (RT-PCR)	1	-	-	-	Pharyngeal swab at any time	-	Placenta IHC	In Utero: Indeterminate N = 1	-
**Iqbal SN, 2020 USA**	Case report	1 mother with confirmed COVID-19 (RT-PCR)	1	-	-	-	Pharyngeal swab at any time	-	-	-	-
**Jacobson J, 2020 USA**	Case report	1 mother with confirmed COVID-19 (RT-PCR)	1	-	-	Amniotic fluid Placenta (any side)	Pharyngeal swab at any time	-	-	-	-
**Kelly JC, 2020 USA**	Case report	1 mother with confirmed COVID-19 (RT-PCR)	1	-	-	-	Pharyngeal swab at any time	-	-	-	-
**Kirtsman M, 2020 Canada**	Case report	1 mother with confirmed COVID-19 (RT-PCR)	1	-	-	Placenta (any side) Placenta (baby side) Vagina Breast milk Rectal swab	Pharyngeal swab at birth Stool Blood	-	-	In Utero: Unlikely N = 1	-
**Kumar (2) V, 2021 USA**	Case report	1 mother with confirmed COVID-19 (RT-PCR)	1	-	-	-	Pharyngeal swab at any time	-	-	In Utero: Possible N = 1	-

**Majachani N, 2020 USA**	Case report	1 mother with confirmed COVID-19 (RT-PCR)	1	-	-	-	Pharyngeal swab at any time	-	-	In Utero: Possible N = 1	-
**Marton T, 2021 USA**	Case report	1 mother with probable COVID-19 (clinical diagnosis)	1	-	-	Placenta tissue (any side)	-	-	Placenta ISH, IHC	In Utero (IUFD): Possible N = 1	1 intrauterine foetal death
**McCarty K, 2020 USA**	Case report	1 mother with confirmed COVID-19 (RT-PCR)	1	-	-	-	Pharyngeal swab at any time	-	-	-	-
**Mehta H, 2020 USA**	Case report	1 mother with confirmed COVID-19 (RT-PCR)	2	-	-	-	Pharyngeal swab at any time	-	-	Postpartum: Indeterminate N = 1	-
**Narang (2) K, 2020 USA**	Case report	1 mother with confirmed COVID-19 (RT-PCR)	1	-	-	-	Pharyngeal swab at any time	-	-	-	-
**Needleman JS, 2020 USA**	Case report	1 mother with confirmed COVID-19 (RT-PCR)	1	-	-	-	Pharyngeal swab at any time	-	-	Not classifiable N = 1	-
**Pelayo J, 2020 USA**	Case report	1 mother with confirmed COVID-19 (RT-PCR)	1	-	-	-	Pharyngeal swab at any time	-	-	-	-
**Rabah R, 2021 USA**	Case report	2 mothers with confirmed COVID-19 (RT-PCR) and 1 with probable COVID-19 (serology)	3 (2 underwent testing)	-	-	Placenta (any side)	-	-	Placenta ISH	In Utero; Indeterminate N = 1 In Utero (IUFD): Possible N=1	-
**Rehana R, 2021 USA**	Case report	1 mother with confirmed COVID-19 (RT-PCR)	1	-	-	-	Pharyngeal swab at any time	-	-	-	-
**Rivera-Hernandez P, 2020 USA**	Case report	1 mother with confirmed COVID-19 (RT-PCR)	1	-	-	-	Pharyngeal swab at any time	-	Neonatal blood IgG	In Utero; Indeterminate N = 1	-
**Roberts J, 2021 USA**	Case report	1 mother with confirmed COVID-19 (RT-PCR)	1	-	-	Placenta tissue (any side)	Pharyngeal swab at any time	-	Placenta IHC	In Utero: Unlikely N = 1	-

**Rong Q, 2020 USA**	Case report	1 mother with confirmed COVID-19 (RT-PCR)	1	-	-	-	Pharyngeal swab at any time	-	-	Not classifiable N = 1	-
**Sainathan S, 2021 USA**	Case report	1 mother with confirmed COVID-19 (RT-PCR)	1	-	-		Pharyngeal swab at any time	-	-	Postpartum: Indeterminate N = 1	-
**Salik I, 2020 USA**	Case report	1 mother with confirmed COVID-19 (RT-PCR)	1	-	-	-	Pharyngeal swab at any time	-	-	Not classifiable N = 1	-
**Schnettler WT, 2020**	Case report	1 mother with confirmed COVID-19 (RT-PCR)	1	-	-	Amniotic fluid	Pharyngeal swab at birth	-	-	-	-
**Suresh CS, 2020**	Case report	1 mother with confirmed COVID-19 (RT-PCR)	1	-	-	-	Pharyngeal swab at any time	-	-	-	-
**Trieu C, 2020 USA**	Case report	1 mother with confirmed COVID-19 (RT-PCR)	1	-	-	-	Pharyngeal swab at any time	-	-	Not classifiable N = 1	-
**Vallejo V, 2020 USA**	Case report	1 mother with confirmed COVID-19 (RT-PCR)	1	-	-	-	Pharyngeal swab at any time	-	-	-	1 maternal death No neonatal death
**Von Kohorn I, 2020 USA**	Case report	1 mother with confirmed COVID-19 (RT-PCR)	1	-	-	Placenta (any side)	Pharyngeal swab at any time Blood Cord blood Neonatal urine	-	-	In Utero: Indeterminate N = 1	-
**South Asia**											
**Agarwal N, 2021 India**	Prospective cohort	65 mothers with confirmed COVID-19 (RT-PCR)	60	Pregnant women with COVID-19 and their neonates from April 15 to June 30, 2020	-	Amniotic fluid Placenta (any side)	Pharyngeal swab at any time	-	-	In Utero: Indeterminate N=1	No maternal deaths 2 neonatal deaths
**Ajith S, 2021 India**	Retrospective cohort	350 mothers with confirmed COVID-19 (RT-PCR or	221	COVID-19 positive mothers and their neonates from April 15 to October 15 2020	Mode of delivery, trimester of infection, ICU admission,	-	Pharyngeal swab at any time	-	-	In Utero: Indeterminate N = 32	No maternal deaths 11 foetal deaths (2

		rapid antigen test)			pregnancy status, maternal severity, preterm birth						IUFD, 9 miscarriage s)
**Anand P, 2020 India**	Retrospecti ve cohort	69 mothers with confirmed COVID-19 (RT-PCR)	65	All neonates born to mothers with COVID-19 between April 1 and July 10, 2020	Mode of delivery, preterm birth, trimester of infection, ICU admission, maternal severity, pregnancy status, rooming in	-	Pharyngeal swab at any time	-	-	In Utero: Indeterminate N = 2 Postpartum: Indeterminate N = 1 Not classifiable N = 4	2 maternal deaths 2 neonatal deaths
**Arora D, 2021 India**	Cohort	54 mothers with confirmed COVID-19 (RT-PCR)	54	Pregnant women with COVID-19 and their neonates between June 1 to October 15, 2020	-	Amniotic fluid	Pharyngeal swab at any time Cord blood PCR	-	-	-	-
**Bachani S, 2020 India**	Retrospecti ve cohort	57 mothers with confirmed COVID-19 (RT-PCR)	56	COVID-19 positive women who delivered between May 5 and June 5 2020, and their neonates	Maternal death	-	Pharyngeal swab at any time	-	-	-	3 maternal deaths 2 neonatal deaths
**Charki S, 2021 India**	Prospective cohort	26 mothers with confirmed COVID-19 (RT-PCR)	28	Neonates born to mothers with COVID-19 from May to October 2020	Preterm delivery, mode of delivery	-	Pharyngeal swab at any time	-	-	Not classifiable N = 1	-
**Chowdhury L 2021 Bangladesh**	Retrospecti ve cohort	81 mothers with confirmed COVID-19 (RT-PCR)	47	Pregnant women with COVID-19 and their neonates, between Mayto July 2020	-	-	Pharyngeal swab at any time	-	-	Not classifiable N = 4	1 maternal death 3 intrauterine deaths 2 neonatal deaths
**Hassan N, 2020 India**	Prospective cohort	38 mothers with confirmed COVID-19 (RT-PCR)	38	Pregnant women with COVID-19 in the third trimester and their neonates, between March to June, 2020	-	-	Pharyngeal swab at any time	-	-	Not classifiable N = 1	No maternal deaths 1 foetal death (IUFD)

											1 neonatal death
**Joshi SD, 2020 India**	Cohort	133 mothers with confirmed COVID-19 (RT-PCR)	137	All pregnant women who tested positive for COVID-19 from June to September 2020, and their neonates	-	-	Pharyngeal swab at any time	-	-	In Utero: Indeterminate N = 7	1 maternal death<br>4 foetal deaths (IUFD)
**Kalamdani P, 2020 India**	Retrospective cohort	185 mothers with confirmed COVID-19 (RT-PCR)	185	SARS-CoV-2 positive neonates born between 1 April and 31 May 2020, and their mothers	Rooming in	-	Pharyngeal swab at any time	-	-	In Utero: Indeterminate N = 6<br>Postpartum: Indeterminate N = 4<br>Not classifiable N = 2	-
**Khushdil A, 2021 Pakistan**	Prospective cohort	106 mothers with confirmed COVID-19 (RT-PCR)	106	Pregnant women who tested positive for SARS-CoV-2 between April and August 2020, and their neonates	Mode of delivery	-	Pharyngeal swab at any time	-	-	In Utero: Possible N = 1<br>In Utero: Unlikely N = 4	1 maternal death
**Kumari K, 2021 India**	Cross-sectional cohort	28 mothers with confirmed COVID-19 (RT-PCR)	29	All pregnant women with COVID-19 admitted for delivery, and their neonates between April to December 2020	-	-	Pharyngeal swab at birth<br>Pharyngeal swab at any time	-	-	-	1 foetal death (stillbirth)
**Mahajan N (2), 2020 India**	Retrospective cohort	879 mothers with confirmed COVID-19 (RT-PCR)	660 (633 underwent testing)	COVID-19 positive pregnant women from April 4 and September 10 2020, and their neonates<br><br>Controls: pre-pandemic pregnant women attending between April 1 2019 and March 31 2020	-	-	Pharyngeal swab at birth	-	Neonatal blood IgG	-	7 maternal deaths<br>1 neonatal death<br>29 foetal deaths (spontaneous abortion, stillbirth)
**Malik S, 2021 India***	Retrospective cohort	514 mothers with confirmed COVID-19 (RT-PCR)	524 (423 underwent testing)	Neonates born to mothers with COVID-19 between April 14 to July 31, 2020	-	-	Pharyngeal swab at any time	-	-	Not classifiable N = 33	13 neonatal deaths

**Malshe N, 2021**	Cohort	57 mothers with confirmed COVID-19 (RT-PCR)	59 (56 underwent testing)	Pregnant women with COVID-19 and their neonates between June and September 2020	Trimester of infection, rooming-in	Blood IgG	Pharyngeal swab at any time<br>Cord blood	Blood IgM	-	In Utero: Indeterminate N = 2	No maternal death<br>1 neonatal death
**Nanavati R, 2021 India**	Retrospective cohort	122 mothers with confirmed COVID-19 (RT-PCR)	125	Neonates with suspected SARS-CoV-2 infection admitted from 15 April to 31 July 2020, and their mothers	Rooming in, preterm birth, breastfeeding	-	Pharyngeal swab at any time	Neonatal blood IgM	Neonatal blood IgG	In Utero: Indeterminate N = 1<br>Peripartum: Unlikely N = 5<br>Not classifiable N = 12	No maternal deaths<br>4 neonatal deaths
**Nayak M, 2021 India**	Prospective cohort	162 mothers with confirmed COVID-19 (RT-PCR)	165 (32 underwent testing)	Neonates born to mothers with COVID-19 between May 1 to October 20 2020	Preterm birth, rooming-in	-	Pharyngeal swab at any time	-	-	In Utero: Indeterminate N=3	1 maternal death<br>4 neonatal deaths
**Pathak S, 2020 India**	Prospective cohort	60 mothers with confirmed COVID-19 (RT-PCR)	60	All neonates born to mothers who tested positive for SARS-CoV2 during pregnancy from 1 June to 20 September, 2020	-	-	Pharyngeal swab at any time	-	-	Not classifiable N = 3	No maternal deaths<br>2 neonatal deaths
**Pawar R, 2021 India**	Cohort	12 mothers: 3 with confirmed COVID-19 (RT-PCR), 3 with probable COVID-19 (serology), 6 with possible COVID-19 (radiological or clinical diagnosis)	13	Neonates born to mothers with COVID-19 who were admitted to seven NICUs between September 1 2020 and April 30 2021	-	-	-	Blood IgM	Blood IgG	-	2 neonatal deaths
**Prasad A, 2021 India**	Prospective cohort	50 mothers with confirmed COVID-19 (RT-PCR)	51	Pregnant women with COVID-19 who delivered and their neonates	Mode of delivery, breastfeeding	Breast milk	Pharyngeal swab at any time	-	-	In Utero: Indeterminate N = 2	1 maternal death<br>1 neonatal death
**Puneet G, 2021 India**	Retrospective cohort	108 mothers with confirmed COVID-19 (RT-PCR)	108	Pregnant women with COVID-19 and their neonates, between September 1 and November 30, 2020	-	-	Pharyngeal swab at any time	-	-	-	1 maternal death<br>2 foetal deaths
**Sayeed SK, 2021 Bangladesh**	Prospective cohort	68 mothers with confirmed COVID-19 (RT-PCR)	51 (41 underwent testing)	Pregnant women with COVID-19 between March 15 to August 15	-	-	Pharyngeal swab at any time	-	-	-	2 maternal deaths

				2020, and their neonates							2 foetal deaths (abortion)
**Sehra R, 2021 India**	Retrospective cohort	120 mothers with confirmed COVID-19 (RT-PCR)	120	Pregnant women with COVID-19 and their neonates between April 13 to July 31 2020	Mode of delivery, preterm birth	-	Pharyngeal swab at any time	-	-	Not classifiable N = 5	-
**Shah PT, 2020 India**	Prospective cohort	125 mothers with confirmed COVID-19 (RT-PCR)	97 (96 underwent testing)	Pregnant women with COVID-19 and their neonates between April 15 to June 10, 2020	-	-	Pharyngeal swab at any time	-	-	Not classifiable N = 16	No maternal deaths<br>2 neonatal deaths<br>6 foetal deaths (abortion, IUFD)
**Sharma N, 2021 India**	Prospective cohort	125 mothers with confirmed COVID-19 (RT-PCR)	97	COVID-19 positive pregnant women admitted from April to September 2020, and their neonates	-	-	Pharyngeal swab at birth	-	-	-	-
**Sharma R, 2021 India**	Ambispective cohort	57 mothers with confirmed COVID-19 (RT-PCR)	44 (30 underwent testing)	Pregnant women with COVID-19 and their neonates between April 1 and August 31 2020	Mode of delivery, preterm birth	Amniotic fluid<br>Placenta (any side)<br>Vaginal fluid<br>Breast milk	Pharyngeal swab at any time<br>Cord blood	-	-	In Utero: Indeterminate N = 1<br>Not classifiable N = 1	-
**Sibia P, 2021 India**	Prospective cohort	150 mothers with confirmed COVID-19 (RT-PCR)	80 (76 underwent testing)	Pregnant women with COVID-19 and their neonates, between March 20 to September 30, 2020	-	-	Pharyngeal swab at any time	-	-	-	1 maternal death<br>4 intrauterine deaths
**Singh V (1), 2021 India**	Retrospective cohort	132 mothers with confirmed COVID-19 (RT-PCR)	125 (121 underwent testing)	COVID-19 positive pregnant women admitted from 15 May to 15 November 2020, and their neonates	Mode of delivery, rooming in	-	Pharyngeal swab at any time	-	-	In Utero: Indeterminate N = 2	No maternal deaths<br>3 neonatal deaths<br>7 foetal deaths (4 IUFD, 3

											miscarriage s)
**Sri Sri G, 2020 India**	Prospective cohort	730 mothers with confirmed/possi ble COVID-19 (RT-PCR or RAT)	500	All pregnant women admitted between July and September 2020, and their neonates	-	-	Pharyngeal swab at any time	-	-	-	-
**Tadas MP, 2021 India**	Cohort	181 mothers with confirmed COVID-19 (RT-PCR)	187	All pregnant women with COVID-19 who delivered, and their neonates between May 1 to August 31, 2020	-	-	Pharyngeal swab at any time	-	-	In Utero: Indeterminate N = 4	1 maternal death<br>1 neonatal death<br>7 foetal deaths (stillbirth)
**Thanigainat han S, 2021 India**	Cohort	30 mothers with confirmed COVID-19 (RT-PCR)	30	Mothers admitted for delivery who tested positive for SARS-CoV-2 between April and August 2020, and their neonates	-	Breast milk	Pharyngeal swab at any time	-	-	-	-
**Yadav V, 2020 India**	Retrospecti ve cohort	28 mothers with confirmed COVID-19 (RT-PCR)	28	COVID-19 positive pregnant women between 23 March and 23 July 2020, and their neonates<br>Controls: COVID-19 negative pregnant women who delivered during the same time frame	-	-	Pharyngeal swab at any time	-	-	-	No maternal deaths<br>1 neonatal death
**Saha J, 2021 India**	Case series	3 mothers with confirmed COVID-19 (RT-PCR)	3	-	-	-	Pharyngeal swab at any time	-	-	Not classifiable N = 2	-
**Saha MM, 2020 Bangladesh**	Case series	10 mothers: 6 with confirmed COVID-19 (RT-PCR), 4 with possible COVID-19 (clinical or radiological diagnosis)	10	-	-	-	Pharyngeal swab at any time	-	-	-	-
**Saha S, 2020 Bangladesh**	Case series	10 mothers: 6 with confirmed COVID-19 (RT-PCR) and 4	10	-	-	-	Pharyngeal swab at any time	-	-	Not classifiable N = 10	No maternal deaths

		with possible COVID-19 (clinical diagnosis)									4 neonatal deaths
**Aatif M, 2020 Pakistan**	Case report	1 mother with confirmed COVID-19 (RT-PCR)	1	-	-	-	Pharyngeal swab at any time	Neonatal blood IgM	-	In Utero: Possible N = 1	-
**Agarwal M, 2020 India**	Case report	1 mother with confirmed COVID-19 (RT-PCR)	1	-	-	-	Pharyngeal swab at birth	-	-	In Utero (IUFD): Possible N = 1	1 foetal death (IUFD)
**Bajwa S, 2020 India**	Case report	1 mother with confirmed COVID-19 (RT-PCR)	1	-	-	CSF	Pharyngeal swab at any time	-	-	-	-
**Bandyopadhyay, 2020 India**	Case report	1 mother with confirmed COVID-19 (RT-PCR)	1	-	-	-	Pharyngeal swab at any time	Neonatal blood IgM	Neonatal blood IgG	In Utero: Unlikely N = 1	-
**Borkotoky KR, 2021 India**	Case report	1 mother with probable COVID-19 (radiological or clinical diagnosis)	1	-	-	-	Pharyngeal swab at any time Tracheal aspirate	Blood IgM	Blood IgG	-	-
**Dhawan S, 2020 India**	Case report	1 mother with confirmed COVID-19 (RT-PCR)	1	-	-	-	Pharyngeal swab at any time	-	-	In Utero: Indeterminate N = 1	-
**Jain P, 2020 India**	Case report	1 mother with confirmed COVID-19 (RT-PCR)	1	-	-	-	Pharyngeal swab at any time	-	-	-	-
**Kappanayil M, 2021 India**	Case report	1 mother with possible COVID-19 (radiological or clinical diagnosis)	1	-	-	-	Pharyngeal swab at any time	Peripheral blood IgM	Peripheral blood IgG Neonatal stool	-	-
**Karade S, 2021 India**	Case report	1 mother with confirmed COVID-19 (RT-PCR)	1	-	-	Amniotic fluid Placental swab (any side) Breast milk	Pharyngeal swab at any time	-	-	In Utero: Possible N=1	-

**Kulkarni R, 2020 India**	Case report	1 mother with probable COVID-19 (serology)	1	-	-	Placenta (any side)	Pharyngeal swab at birth<br>Pharyngeal swab at any time<br>Cord stump	Neonatal blood IgM	Maternal blood antibodies<br>Neonatal blood IgG and IgA	In Utero: Possible<br>N = 1	-
**Kumar V, 2020 India**	Case report	1 mother with confirmed COVID-19 (RT-PCR)	1	-	-	-	Pharyngeal swab at any time	-	-	-	-
**Sharma KA, 2020 India**	Case report	1 mother with confirmed COVID-19 (RT-PCR)	1	-	-	-	Pharyngeal swab at any time	-	-	-	
**Shende P 2020, India**	Case report	1 mother with confirmed COVID-19 (RT-PCR)	1	-	-	Amniotic fluid<br>Placenta (any side)	-	-	-	In Utero (IUFD): Possible<br>N = 1	1 foetal death (miscarriage)
**Singh MV, 2020 India**	Case report	1 mother with confirmed COVID-19 (RT-PCR)	1	-	-	-	Pharyngeal swab at any time	-	-	In Utero: Indeterminate<br>N = 1	-
**Singh P, 2020 India**	Case report	1 mother with possible COVID-19 (radiological or clinical diagnosis)	1	-	-	-	Pharyngeal swab at any time	-	-	-	
**Thapa B, 2021 Nepal**	Case report	1 mother with confirmed COVID-19 (RT-PCR)	1	-	-	-	Pharyngeal swab at any time	-	-	In Utero: Indeterminate<br>N=1	-
**Sub-Saharan Africa**											
**Dingom MAN 2020, Cameroon**	Cohort	25 mothers with confirmed COVID-19 infection (RT-PCR or RAT)	9 (7 underwent testing)	Pregnant women with confirmed COVID-19 and their neonates between April 1 to June 30, 2020	-	-	Pharyngeal swab at any time	-	-	-	No maternal deaths<br>4 foetal deaths (2 IUFD, 2 abortions)
**Ngalame AN, 2020 Cameroon**	Prospective cohort	18 mothers with confirmed COVID-19 (RT-PCR)	13 (3 underwent testing)	All mothers with COVID-19 diagnosed in pregnancy or postpartum and their neonates, between	-	-	Pharyngeal swab at any time	-	-	-	4 maternal deaths<br>No foetal/neonatal deaths

				March 24 and July 24 2020							
**Birindwa EK, 2020 DR Congo**	Case report	1 mother with confirmed COVID-19 (RT-PCR)	1	-	-	-	Pharyngeal swab at any time	-	-	In Utero: Indeterminate N = 1	Neonatal death
**Makwe CC, 2020 Nigeria**	Case report	1 mother with confirmed COVID-19 (RT-PCR)	1	-	-	-	Pharyngeal swab at any time	-	-	-	-
**Nakstad B, 2021 Botswana**	Case report	2 mothers with confirmed COVID-19 (RT-PCR)	2	-	-	-	Pharyngeal swab at any time	-	-	Postpartum: Possible N=1	-

* RT-PCR: reverse transcription-polymerase chain reaction; RAT: rapid antigen test; IUFD: intrauterine foetal demise

References of included studies

1. Aatif M, Basheer F, Jalil J, Jahanzaib S, Khan FS. Vertical Transmission of Corona Virus – Possibly First Case From Pakistan. Pak Armed Forces Med. 2020 Aug 8; 70(1), S427-29. Available from: https://www.pafmj.org/index.php/PAFMJ/article/view/4947
2. Abadía-Cuchí N, Ruiz-Martínez S, Fabre M, et al. SARS-CoV-2 congenital infection and pre-eclampsia-like syndrome in dichorionic twins: A case report and review of the literature. *Int J Gynecol Obstet*. 2021;154(2):370-372. doi:10.1002/ijgo.13749. PMID: 34009656
3. Abasse S, Essabar L, Costin T, Mahistra V, Kaci M, Braconnier A, et al. Neonatal COVID 19 Pneumonia: Report of the First Case in a Preterm Neonate in Mayotte, an Overseas Department of France. Children (Basel). 2020 Aug;7(8):87. doi: 10.3390/children7080087 pmid: 32756337
4. Abdulghani S., Shaiba L., Bukhari M. et al. Consequences of SARS-CoV-2 disease on maternal, perinatal and neonatal outcomes: a retrospective observational cohort study. *Clin. Exp. Obstet. Gynecol.* 2021, 48(2), 353–358. doi: 10.31083/j.ceog.2021.02.2361
5. Abedzadeh-Kalahroudi M, Sehat M, Vahedpour Z, Talebian P. Maternal and neonatal outcomes of pregnant patients with COVID-19: A prospective cohort study. Int J Gynaecol Obstet. 2021 Jun; 153(3):449-456.doi: 10.1002/ijgo.13661 pmid: 33638200
6. Abourida Y, Rebahi H, Oussayeh I, et al. Case Report Management of Severe COVID-19 in Pregnancy. 2020; 2020:1-5. PMID: 32733724
7. Adhikari EH, Moreno W, Zofkie AC, MacDonald L, McIntire DD, Collins RRJ, et al. Pregnancy Outcomes among Women with and without Severe Acute Respiratory Syndrome Coronavirus 2 Infection. JAMA Netw Open. 2020 Nov 19;3(11):e2029256.doi: 10.1001/jamanetworkopen.2020.29256. pmid: 33211113
8. Agarwal M, Basumatary S, Kant B, Kumar S. Intrauterine Transmission of SARS-CoV-2 (COVID-19 Virus). J Obstet Gynaecol India. 2021 Mar 3; 1-3. doi: 10.1007/s13224-021-01431-y. pmid: 33679015
9. Agarwal N, Garg R, Singh S, Agrawal A. Coronavirus disease 2019 in pregnancy: Maternal and perinatal outcome. J Educ Health Promot. 2021 May;10(1):194. doi: 10.4103/jehp.jehp_954_20. PMID: 34250128.
10. Aghdam M, Jafari N, Eftekhari K. Novel coronavirus in a 15-day-old neonate with clinical signs of sepsis, a case report. Infect Dis (Lond). 2020 Jun 2;52(6):427–9. doi: 10.1080/23744235.2020.1747634 pmid: 32233816
11. AlZaghal LA, AlZaghal N, Alomari SO, Obeidat N, Obeidat B, Hayajneh WA. Multidisciplinary team management and cesarean delivery for a Jordanian woman infected with SARS-COV-2: A case report. Case Rep Womens Health. 2020 May 1;27:e00212. doi: 10.1016/j.crwh.2020.e00212. PMID: 32523874
12. Ajith S, Reshmi VP, Nambiar S, Naser A, Athulya B. Prevalence and Risk Factors of Neonatal Covid-19 Infection: A Single-Centre Observational Study. J Obstet Gynaecol India. 2021 Feb 4:1–4. doi: 10.1007/s13224-021-01436-7 pmid: 33564217
13. Akbarian-Rad Z, Mojaveri MH, Bouzari Z, et al. Neonatal Outcomes in Pregnant Women Infected with COVID-19 in Babol, North of Iran: A Retrospective Study with Short-Term Follow-Up. *Infect Dis Obstet Gynecol*. 2021;2021:4-9. doi:10.1155/2021/9952701. PMID: 34188437
14. Akdemir Y, Haciseyitoglu D, Celebi G, et al. Probable viremia and positive placental swabs for SARS-CoV-2 in a preterm pregnant woman with mild COVID-19. *J Med Virol*. 2021;93(12):6788-6793. doi:10.1002/jmv.27202. PMID: 34260081
15. Al-Kuraishy H, Al-Maiahy T, Al-Gareeb A, Musa R, Ali Z. COVID-19 pneumonia in an Iraqi pregnant woman with preterm delivery. *Asian Pacific J Reprod*. 2020;9(3):156-158. doi:10.4103/2305-0500.282984.
16. Al-Matary A, Almatari F, Al-Matary M, AlDhaefi A, et al. Clinical outcomes of maternal and neonate with COVID-19 infection - Multicenter study in Saudi Arabia. J Infect Public Health. 2021 Jun;14(6):702-708. doi: 10.1016/j.jiph.2021.03.013. PMID: 34020209
17. Alario D, Bracaglia G, Franceschini G, Arcangeli F, Mecarini F. Orange discoloration of the skin in mother and newborn with SARS-CoV-2 infection: is hypercarotenosis a sign of COVID-19? Journal of Pediatric and Neonatal Individualized Medicine. 2021 Apr;10(1):e100101. doi: 10.7363/100101
18. Alay I, Yildiz S, Kaya C, Yasar KK, Aydin OA, Karaosmanoglu HK, et al. The clinical findings and outcomes of symptomatic pregnant women diagnosed with or suspected of having coronavirus disease 2019 in a tertiary pandemic hospital in Istanbul, Turkey. J Obstet Gynaecol Res. 2020 Sep 21: 10.1111/jog.14493. doi: 10.1111/jog.14493 pmid: 32954601
19. Algadeeb KB, AlMousa HH, AlKadhem SM, Alduhilan MO, Almatawah Y. A Novel Case of Severe Respiratory Symptoms and Persistent Pulmonary Hypertension in a Saudi Neonate With SARS-CoV-2 Infection. Cureus. 2020 Sep 15; 12(9): e10472. doi: 10.7759/cureus.10472 pmid: 33083174

20. Algarroba GN, Rekawek P, Vahanian SA, Khullar P, Palaia T, Peltier MR, et al. Visualization of SARS-CoV-2 virus invading the human placenta using electron microscopy. Am J Obstet Gynecol. 2020 Aug; 223(2): 275–278. doi: 10.1016/j.ajog.2020.05.023 pmid: 32405074
21. Dávila-Aliaga C, Hinojoza-Pérez R, Espinola-Sánchez M, Torres-Marcos E, Guevara-Ríos E, Espinoza-Vivas Y, et al. Resultados materno-perinatales en gestantes con COVID-19 en un hospital nivel III del Perú. Revista Peruana de Medicina Experimental y Salud Pública. 2021 Mar 26;38(1):58–63. doi: https://doi.org/10.17843/rpmesp.2021.381.6358
22. Dávila-Aliaga, C, Espínola-Sánchez, M, Mendoza-Ibáñez, E, et al. Resultados perinatales y serológicos en neonatos de gestantes seropositivas para SARS-CoV-2: estudio transversal descriptivo. *Medwave* 2020;20(11):e8084 doi: 10.5867/medwave.2020.11.8084
23. Alnashry ML, Webair H, Alzabed A, Fatma A. Covid-19 in pregnancy-does it related to intrauterine growth restriction (IUGR)? RCOG Virtual World Congress 2021- Top 500 abstract
24. Alsharaydeh I, Rawashdeh H, Saadeh N, Obeidat B, Obeidat N. Challenges and solutions for maternity and gynecology services during the COVID-19 crisis in Jordan. *Int J Gynecol Obstet*. 2020;150(2):159-162. doi:10.1002/ijgo.13240. PMID: 32470178.
25. Alvarado-Socarras JL, Theurel-Martin D, Cruz-Hernandez M, Rodriguez-Morales AJ. Community-Acquired Neonatal SARS-CoV-2 Infection Associated with Neurological Symptoms in Colombia. *J Trop Pediatr*. 2021;67(1). doi:10.1093/tropej/fmab022. PMID: 33823049.
26. Alwardi TH, Ramdas V, al Yahmadi M, al Aisari S, Bhandari S, Saif Al Hashami H, et al. Is Vertical Transmission of SARS-CoV-2 Infection Possible in Preterm Triplet Pregnancy? A Case Series. Pediatr Infect Dis J. 2020 Dec;39(12):e456-e458. doi: 10.1097/INF.0000000000002926. pmid: 33006879
27. Alzamora MC, Paredes T, Caceres D, Webb CM, Webb CM, Valdez LM, et al. Severe COVID-19 during Pregnancy and Possible Vertical Transmission. Am J Perinatol. 2020 Jun 1;37(8):861-865. doi: 10.1055/s-0040-1710050 pmid: 32305046
28. Amiraskari R, Sayarifard E, Kharrazi H, Naserfar N, Sayarifard A. Neonatal sars-cov-2 infection and congenital myocarditis: A case report and literature review. Arch Pediatr Infect Dis. 2020 Jun 15; 8(3): e103504. doi: 10.5812/pedinfect.103504.
29. Anand P, Yadav A, Debata P, Bachani S, Gupta N, Gera R. Clinical profile, viral load, management and outcome of neonates born to COVID 19 positive mothers: a tertiary care centre experience from India. Eur J Pediatr. 2020 Sep 10 : 1–13. doi: 10.1007/s00431-020-03800-7 pmid: 32910210
30. Angelidou A, Sullivan K, Melvin PR, Shui JE, Goldfarb IT, Bartolome R, Chaudhary N, Vaidya R, Culic I, Singh R, Yanni D, Patrizi S, Hudak ML, Parker MG, Belfort MB. Association of Maternal Perinatal SARS-CoV-2 Infection with Neonatal Outcomes during the COVID-19 Pandemic in Massachusetts. *JAMA Netw Open*. 2021;4(4):1-14. doi:10.1001/jamanetworkopen.2021.7523. PMID: 33890989
31. Anggraini NWP, Sulistyowati S. Low neutrophil-to-lmphocyte ratio decreases risk of coronavirus disease in pregnant women. *Universa Med*. 2020;39(2):88-96. doi:10.18051/univmed.2020.v39.88-96.
32. Antsaklis P, Tasias K, Psarris A, Theodora M, Daskalakis G, Rodolakis A. Covid-19 and pregnancy: The experience of a tertiary maternity hospital. J Perinat Med. 2021;49(6):686-690. doi:10.1515/jpm-2021-0070. PMID: 34187133
33. Aouali K, Moustaid H, Moustaid H, Benkirane S, Benkirane S. COVID-19 and pregnancy: the fruitful experience of the university hospital of Tangier Morocco with hydroxychloroquine and anti-retroviral treatment. *Int J Reprod Contraception, Obstet Gynecol*. 2021;10(3):1143. doi:10.18203/2320-1770.ijrcog20210749.
34. Arakaki T, Hasegawa St J, Kinoshita Seijyo Kinoshita Hospital K. Clinical Characteristics of Pregnant Women With COVID-19 In Japan: A Nationwide Questionnaire Survey. 2020 Oct. Available at SSRN: https://ssrn.com/abstract=3700896
35. Argueta LB, Lacko LA, Bram Y, Tada T, Carrau L, Zhang T et al. SARS-CoV-2 Infects Syncytiotrophoblast and Activates 2 Inflammatory Responses in the Placenta. bioRxiv 2021.06.01.446676. doi: 10.1101/2021.06.01.446676.
36. Arora D, Rajmohan KS, Dubey S, Dey M, Singh S, Nair VG, Tiwari RP, Tiwari S. Assessment of materno-foetal transmission of SARS-CoV-2: A prospective pilot study. Med J Armed Forces India. 2021 Jul;77(Suppl 2):S398-S403. doi: 10.1016/j.mjafi.2021.01.007. PMID: 34334910.
37. Artymuk N v., Belokrinitskaya TE, Filippov OS, Frolova NI, Surina MN. Perinatal outcomes in pregnant women with COVID-19 in Siberia and the Russian Far East. J Matern Fetal Neonatal Med. 2021 Feb 2;1-4. doi: 10.1080/14767058.2021.1881954. pmid: 33530804
38. Askary E, Poordast T, Shiravani Z, Ashraf MA, Hashemi A, Naseri R, et al. Coronavirus disease 2019 (COVID-19) manifestations during pregnancy in all three trimesters: A case series. Int J Reprod Biomed. 2021 Feb; 19(2): 191–204. doi: 10.18502/ijrm.v19i2.8477 pmid: 33718763

39. Aslan MM, Uslu Yuvacı H, Köse O, Toptan H, Akdemir N, Köroğlu M, et al. SARS-CoV-2 is not present in the vaginal fluid of pregnant women with COVID-19. J Matern Fetal Neonatal Med. 2020 Jul 16;1-3. doi: 10.1080/14767058.2020.1793318 pmid: 32669014
40. Ayed A, Embaireeg A, Benawath A, Al-Fouzan W, Hammoud M, Al-Hathal M, et al. Maternal and perinatal characteristics and outcomes of pregnancies complicated with COVID-19 in Kuwait. BMC Pregnancy Childbirth. 2020 Dec 2;20(1):754. doi: 10.1186/s12884-020-03461-2 pmid: 33267785
41. Bachani, S., Arora, R., Dabral, A. et al. Clinical Profile, Viral Load, Maternal-Fetal Outcomes of Pregnancy With COVID-19: 4-Week Retrospective, Tertiary Care Single-Centre Descriptive Study. Journal of Obstetrics and Gynaecology Canada. 2020; 43(4): 474 – 482. doi:10.1016/j.jogc.2020.09.021
42. Bae JG, Ha J, Kwon M, Park HY, Seong WJ, Hong SY. A case of delivery of a pregnant woman with COVID-19 infection in Daegu, Korea. *Obstet Gynecol Sci*. 2020;63(6):745-749. doi:10.5468/OGS.20106. PMID: 33012160
43. Bai, BL., Zhongliang, G., Shuangying, H., et al. A case of multi-site etiological detection of novel coronavirus pneumonia in mother and her newborn. Chinese journal of neonatology. 2020 Apr 24; 35(2):85-86. doi: 10.3760/cma.j.issn.2096-2932.2020.02.003
44. Bajwa SJS, Gupta R, Wahi A, Goraya SPS. Exploring the unknown territories in the new normal world of COVID. J Anaesthesiol Clin Pharmacol. 2020 ;36(Suppl 1):S77-S80. doi: 10.4103/joacp.JOACP_350_20. PMID: 33100651
45. Bandyopadhyay T, Sharma A, Kumari P, Maria A, Choudhary R. Possible Early Vertical Transmission of COVID-19 from an Infected Pregnant Female to Her Neonate: A Case Report. J Trop Pediatr. 2021 Jan 29;67(1):fmaa094. doi: 10.1093/tropej/fmaa094 pmid: 33221859
46. Banerjee A, Arrandale LA, Sankaran S, Glover GW, Nelson-Piercy CF. Approach to dyspnoea in pregnancy in the COVID-19 era. *Acute Med*. 2020;19(4):230-234. doi:10.52964/amja.083. PMID: 33215176
47. Baquero H, Venegas ME, Velandia L, Neira F, Navarro E. Neonatal late-onset infection with SARS CoV-2. Biomedica. 2020 Oct 30;40(Supl. 2):44-49. doi: 10.7705/biomedica.5609 pmid: 33152187
48. Barber E, Kovo M, Leytes S, Sagiv R, Weiner E, Schwartz O et al. Evaluation of SARS-CoV-2 in the Vaginal Secretions of Women with COVID-19: A Prospective Study. J. Clin. Med. 2021, 10, 2735. doi: 10.3390/jcm10122735
49. Barbero P, Mugüerza L, Herraiz I, García Burguillo A, San Juan R, Forcén L, Mejía I, Batllori E, Montañez MD, Vallejo P, Villar O, García Alcazar D, Galindo A. SARS-CoV-2 in pregnancy: characteristics and outcomes of hospitalized and non-hospitalized women due to COVID-19. J Matern Fetal Neonatal Med. 2020 Jul 20:1-7. doi: 10.1080/14767058.2020.1793320. pmid: 32689846.
50. Bastug A, Hanifehnezhad A, Tayman C, Ozkul A, Ozbay O, Kazancioglu S, et al. Virolactia in an Asymptomatic Mother with COVID-19. Breastfeed Med. 2020 Aug;15(8):488-491. doi: 10.1089/bfm.2020.0161 pmid: 32614251
51. Basyir V, Adnani SR, Fauziah PN. Caeserean delivery of women with COVID-19 in padang, indonesia: A case report. *Syst Rev Pharm*. 2021;12(3):698-700. doi:10.31838/srp.2021.3.97.
52. Baud D, Greub G, Favre G, Gengler C, Jaton K, Dubruc E, et al. Second-Trimester Miscarriage in a Pregnant Woman with SARS-CoV-2 Infection. JAMA. 2020 Jun 2; 323(21): 2198–2200. doi: 10.1001/jama.2020.7233 pmid: 32352491
53. Beharier O, Mayo RP, Raz T, Sacks KN, Schreiber L, Suissa-Cohen Y, Chen R, Gomez-Tolub R, Hadar E, Gabbay-Benziv R, Moshkovich YJ, Biron-Shental T, Shechter-Maor G, Farladansky-Gershnabel S, Sela HY, Raischer HB, Sela ND, Goldman-Wohl D, Shulman Z, Many A, Barr H, Yagel S, Neeman M, Kovo M. Efficient maternal to neonatal transfer of SARS-CoV-2 and BNT162b2 antibodies. *medRxiv*. 2021:1-26. doi:10.1101/2021.03.31.21254674.
54. Behling JAK, Zanirati G, Rodrigues FVF, Grahl MVC, Krimberg FD, Pinzetta G, et al. Placental maternal vascular malperfusion affecting late fetuses development and multi-organ infection caused by SARS-CoV-2. Research Square. 2020 Oct 9. doi: 10.21203/rs.3.rs-88599/v1
55. Bender WR, Hirshberg A, Coutifaris P, Acker AL, Srinivas SK. Universal testing for severe acute respiratory syndrome coronavirus 2 in 2 Philadelphia hospitals: carrier prevalence and symptom development over 2 weeks. Am J Obstet Gynecol MFM. 2020 Nov;2(4):100226. doi: 10.1016/j.ajogmf.2020.100226 pmid: 32954248
56. Berry M, Wang A, Clark SM, Harirah HM, Jain S, Olson GL, et al. Clinical Stratification of Pregnant COVID-19 Patients based on Severity: A Single Academic Center Experience. Am J Perinatol. 2021 Apr;38(5):515-522. doi: 10.1055/s-0041-1723761 pmid: 33548937
57. Bertero L, Borella F, Botta G, Carosso A, Cosma S, Bovetti M et al. Placenta histopathology in SARS-CoV-2 infection: analysis of a consecutive series and comparison with control cohorts. Virchows Archiv. 479, 715–728. doi: 10.1007/s00428-021-03097-3.

58. Bertino E, Moro GE, de Renzi G, Viberti G, Cavallo R, Coscia A, et al. SARS-CoV-2 in Human Breast Milk and Neonatal Outcome: A Collaborative Study. 2020 Jun. Available at http://dx.doi.org/10.2139/ssrn.3611974
59. Biasucci G, Cannalire G, Raymond A, Capra ME, Benenati B, Vadacca G, et al. Safe Perinatal Management of Neonates Born to SARS-CoV-2 Positive Mothers at the Epicenter of the Italian Epidemic. Front Pediatr. 2020; 8: 565522. doi: 10.3389/fped.2020.565522 pmid: 33194893
60. Birindwa EK, Mulumeoderhwa GM, Nyakio O, Mbale GQM, Mushamuka SZ, Materanya JM, et al. A case study of the first pregnant woman with COVID-19 in Bukavu, eastern Democratic Republic of the Congo. Matern Health Neonatol Perinatol. 2021 Jan 20;7(1):6. doi: 10.1186/s40748-021-00127-5 pmid: 33472696
61. Bordbar A, Kashaki M, Rezaei F, Jafari R. Vertical transmission of COVID-19 in a 1-day-old neonate. Travel Med Infect Dis. 2020 November-December; 38: 101879. doi: 10.1016/j.tmaid.2020.101879 pmid: 32950662
62. Borkotoky RK, Barua PB, Paul SP, Heaton PA. COVID-19-Related Potential Multisystem Inflammatory Syndrome in Childhood in a Neonate Presenting as Persistent Pulmonary Hypertension of the Newborn. Pediatr Infect Dis J. 2021;40(4):e162-e164. doi: 10.1097/INF.0000000000003054. PMID: 33464010.
63. Bouachba A, Allias F, Nadaud B, Massardier J, Mekki Y, Bouscambert Duchamp M, Fourniere B, Huissoud C, Trecourt A, Collardeau-Frachon S. Placental lesions and SARS-Cov-2 infection: Diffuse placenta damage associated to poor fetal outcome. Placenta. 2021;112:97-104. doi: 10.1016/j.placenta.2021.07.288. PMID: 34329973

64. Bozkurt F, Coskun O, Yelec S, Bekcibasi M, Asena M, Bagli I. Comparison of the Clinical and Laboratory Findings in COVID-19 Positive 2 Pregnants without Comorbidity. Turk J Med Sci. 2021:7;8. doi: 10.3906/sag-2105-116. PMID: 34233388
65. Braga LFB, Sass N. Coronavirus 2019, Thrombocytopenia and HELLP Syndrome: Association or Coincidence? *Rev Bras Ginecol e Obstet*. 2020;42(10):669-671. doi:10.1055/s-0040-1718437. PMID: 33129222
66. Brandt JS, Hill J, Reddy A, Schuster M, Patrick HS, Rosen T, Sauer M V., Boyle C, Ananth C V. Epidemiology of coronavirus disease 2019 in pregnancy: risk factors and associations with adverse maternal and neonatal outcomes. *Am J Obstet Gynecol*. 2021;224(4):389.e1-389.e9. doi:10.1016/j.ajog.2020.09.043. PMID: 32986989.
67. Briana DD, Papadopoulou A, Syridou G, Marchisio E, Kapsabeli E, Daskalaki A, Papaevangelou V. Early human milk lactoferrin during SARS-CoV-2 infection. J Matern Neonatal Med. 2021;0(0):1-4. doi:10.1080/14767058.2021.1920010. PMID: 33969775.

68. Brito I, Sousa R, Saaches B, Franco J, Marcellino S, Costa A. Rooming-in, Breastfeeding with Neonatal Follow-up of Infants Born to Mothers with COVID-19. Acta Med Port 2021 Jul-Aug;34(7-8):507-516. doi: 10.20344/amp.1544.
69. Buhimschi CS, Elam GL, Locher SR, Norris-Stojak D, Aldasoqi H, Stephenson MD, et al. Prevalence and Neighborhood Geomapping of COVID-19 in an Underserved Chicago Pregnant Population. AJP Rep. 2020 Oct; 10(4): e413–e416. doi: 10.1055/s-0040-1721416 pmid: 33294287
70. Buonsenso D, Costa S, Sanguinetti M, Cattani P, Posteraro B, Marchetti S, et al. Neonatal Late Onset Infection with Severe Acute Respiratory Syndrome Coronavirus 2. Am J Perinatol. 2020 Jun;37(8):869-872. doi: 10.1055/s-0040-1710541 pmid: 32359227
71. Çakırca TD, Torun A, Hamidanoğlu M, Portakal RD, Ölçen M, Çakırca G et al. COVID-19 infection in pregnancy: a single center experience with 75 cases COVID-19 infection in pregnancy. Ginekologia Polska. 2021;7:15. doi: 10.5603/GP.a2021.0118
72. Caplliure AP, Almela MP, Albert AA, Vicente EM, Roig BM. Utilidad de la ecografía torácica en infección neonatal por SARS-CoV-2. An Pediatr (Barc). 2021 Jun; 94(6): 412–413. doi: 10.1016/j.anpedi.2020.04.027 pmid: 32736924
73. Carbayo-Jiménez T, Carrasco-Colom J, Epalza C, Folgueira D, Pérez-Rivilla A, Barbero-Casado P, et al. Severe Acute Respiratory Syndrome Coronavirus 2 Vertical Transmission from an Asymptomatic Mother. Pediatr Infect Dis J. 2021 Mar 1;40(3):e115-e117. doi: 10.1097/INF.0000000000003028 pmid: 33565817
74. Cardona-Pérez JA, Villegas-Mota I, Helguera-Repetto AC, et al. Prevalence, clinical features, and outcomes of SARS-CoV-2 infection in pregnant women with or without mild/moderate symptoms: Results from universal screening in a tertiary care center in Mexico City, Mexico. *PLoS One*. 2021;16(4 April):1-19. doi:10.1371/journal.pone.0249584. PMID: 33886590
75. Cavaliere AF, Marchi L, Aquilini D, Brunelli T, Vasarri PL. Passive immunity in newborn from SARS-CoV-2-infected mother. *J Med Virol*. 2021;93(3):1810-1813. doi:10.1002/jmv.26609. PMID: 33073377

76. Cavicchiolo ME, Trevisanuto D, Lolli E, et al. Universal screening of high-risk neonates, parents, and staff at a neonatal intensive care unit during the SARS-CoV-2 pandemic. *Eur J Pediatr*. 2020;179(12):1949-1955. doi:10.1007/s00431-020-03765-7. PMID: 32767137.
77. Chaichian S, Mehdizadehkashi, Mirgalobayat S, Hashemi N, Farzaneh F, Derakhshan R et al. Maternal and Fetal Outcomes of Pregnant Women Infected with Coronavirus Based on Tracking the Results of 90-Days Data in Hazrat-E-Rasoul Akram Hospital, Iran University of Medical Sciences. Bull Emerg trauma. 2021;9(3):145-150. doi:10.30476/BEAT.2021.90434.1254.
78. Chambers C, Krogstad P, Bertrand K, Contreras D, Tobin N, Bode L, et al. Evaluation of SARS-CoV-2 in Breastmilk from 18 Infected Women. JAMA. 2020 Oct 6;324(13):1347-1348. doi: 10.1001/jama.2020.15580 pmid: 32822495
79. Charki S, Gamini BS, Biradar V, et al. Experience of covid-19 infections in neonates in tertiary care centre in North Karnataka, India: A prospective cohort study. *Curr Pediatr Res*. 2021;25(3):421-425.
80. Chen, R., Chen, J. & Meng, QT. Chest computed tomography images of early coronavirus disease (COVID-19). Can J Anesth/J Can Anesth. 2020; 67, 754-755. doi: 10.1007/s12630-020-01625-4
81. Chen S, Huang B, Luo DJ, Li X, Yang F, Zhao Y, Nie X, Huang BX. Pregnancy with new coronavirus infection: clinical characteristics and placental pathological analysis of three cases. Zhonghua Bing Li Xue Za Zhi. 2020 May 8;49(5):418-423. Chinese. doi: 10.3760/cma.j.cn112151-20200225-00138. pmid: 32114744.
82. Cheng B, Jiang T, Zhang L, Hu R, Tian J, Jiang Y, et al. Clinical Characteristics of Pregnant Women With Coronavirus Disease 2019 in Wuhan, China. Open Forum Infect Dis. 2020 Aug; 7(8): ofaa294. doi: 10.1093/ofid/ofaa294 pmid: 32760752
83. Choobdar FA, Ghassemzadeh M, Attarian M, et al. Transplacental transmission of SARS-CoV-2 infection: A case report from Iran. *Arch Pediatr Infect Dis*. 2021;9(2):1-6. doi:10.5812/pedinfect.108582. doi: 10.5812/pedinfect.108582.
84. Chowdhury, L., Jahan, I., Sharmin, M. I., Ferdaushi, J., Tasnim, T., & Nahar, S Fetomaternal Outcome of Pregnancy with COVID-19: An Observational Study in A Tertiary Care Hospital of Bangladesh. *Journal of Bangladesh College of Physicians and Surgeons*. 2021; *39*(2), 100–105. https://doi.org/10.3329/jbcps.v39i2.52390
85. Clemente MJ, Amosco MD, Octavio MBR, Bravo SLR, Villanueva-Uy E. Maternal and Neonatal Outcomes of Pregnant Women with Clinically Confirmed COVID-19 Admitted at the Philippine General Hospital. Acta Medica Philippina. 2021; 55:2.
86. Clough BM. Triple Threat: Postpartum, Coronavirus Disease 2019 Positive, and Requiring Extracorporeal Membrane Oxygenation. *Air Med J*. 2021;40(2):124-126. doi:10.1016/j.amj.2020.12.009. PMID: 33637276
87. Cojocaru L, Crimmins S, Sundararajan S, Goetzinger K, Elsamadicy E, Lankford A, et al. An initiative to evaluate the safety of maternal bonding in patients with SARS-CoV-2 infection. J Matern Fetal Neonatal Med. 2020 Sep 30;1-7. doi: 10.1080/14767058.2020.1828335 pmid: 32998572
88. Colson A, Depoix CL, Dessilly G, et al. Clinical and in Vitro Evidence against Placenta Infection at Term by Severe Acute Respiratory Syndrome Coronavirus 2. Am J Pathol. 2021;191(9):1610-1623. doi:10.1016/j.ajpath.2021.05.009. PMID: 34111431
89. Conti MG, Terreri S, Mortari EP, Albano C, Natale F, Boscarino G et al. Immune Response of Neonates Born to Mothers Infected With SARS-CoV-2. JAMA Network Open. 2021;4(11):e2132563. doi:10.1001/jamanetworkopen.2021.32563
90. Resita BI, Corebima V, Sulistijono E, et al. CASE REPORT Clinical features and neonatal outcomes of neonatus with mother suspected COVID-19 in Malang, Indonesia: a serial-cases, single-centre. *Pediatr Sci J*. 2020;1(1):27-34. http://pedscij.org.

91. Correia CR, Marçal M, Vieira F, Santos E, Novais C, Maria AT et al. Congenital SARS-CoV-2 Infection in a Neonate With Severe Acute Respiratory Syndrome. Pediatr Infect Dis J. 2020 Dec;39(12):e439-e443. doi: 10.1097/INF.0000000000002941 pmid: 33060519
92. Cosma S, Carosso AR, Corcione S, Cusato J, Borella F, Antonucci M, et al. Longitudinal analysis of antibody response following SARS-CoV-2 infection in pregnancy: From the first trimester to delivery. J Reprod Immunol. 2021 Apr;144:103285. doi: 10.1016/j.jri.2021.103285 pmid: 33582489
93. Cribiù FM, Erra R, Pugni L, Rubio-Perez C, Alonso L, Simonetti S, et al. Severe SARS-CoV-2 placenta infection can impact neonatal outcome in the absence of vertical transmission. J Clin Invest. 2021 Mar 15;131(6):e145427. doi: 10.1172/JCI145427 pmid: 33497369
94. Cubas J, Ventura S, Montes O, Julcamoro G, Aguilar G, Pajares, et al. Características clínicas de gestantes en trabajo de parto con infección SARSCoV- 2 en la altura: serie de casos. Revista Peruana de Ginecología y Obstetricia. 2020 Nov 9;66(3). doi: https://doi.org/10.31403/rpgo.v66i2276

95. Cui P, Chen Z, Wang T, et al. Severe acute respiratory syndrome coronavirus 2 detection in the female lower genital tract. *Am J Obstet Gynecol*. 2020;223(1):131-134. doi:10.1016/j.ajog.2020.04.038. PMID: 32395222
96. Maeda M de FY, Brizot M de L, Gibelli MABC, Ibidi SM et al. Vertical transmission of SARS-CoV2 during pregnancy: A high-risk cohort. Prenat Diagn. 2021;41(8):998-1008. doi:10.1002/pd.5980. PMID: 34101871
97. De Socio GV, Malincarne L, Arena S, et al. Delivery in asymptomatic Italian woman with SARS-CoV-2 infection. *Mediterr J Hematol Infect Dis*. 2020;12(1):2-4. doi:10.4084/MJHID.2020.033. PMID: 32395222
98. Gaspar DVA, Silva SI. SARS-CoV-2 in Pregnancy-The First Wave. Medicina (Kaunas). 2021;57(3):241. doi:10.3390/medicina57030241. PMID: 33807607
99. Demirjian A, Singh C, Tebruegge M, Herbert R, Draz N, Mirfenderesky M, et al. Probable Vertical Transmission of SARS-CoV-2 Infection. Pediatr Infect Dis J. 2020 Sep;39(9):e257-e260. doi: 10.1097/INF.0000000000002821 pmid: 32658096
100. Deveci M, Gokce I, Ozdemir R. Neonatal COVID-19 case from Turkey; a Case report of neonatal. Ann Med Res. 2020;27(11):3037-40. doi: 10.5455/annalsmedres.2020.08.804
101. Dhawan S, Pandey M. SARS-CoV-2 Vertical Transmission: Rare But a Potential Possibility. Indian J Pediatr. 2020 Oct 8:1. doi: 10.1007/s12098-020-03498-3 pmid: 33034001
102. Dhuyvetter A, Cejtin HE, Adam M, Patel A. Coronavirus Disease 2019 in Pregnancy: The Experience at an Urban Safety Net Hospital. J Community Health. 2021 Apr;46(2):267-269. doi: 10.1007/s10900-020-00940-7 pmid: 33128667
103. Di Nicola P, Ceratto S, Dalmazzo C, Roasio L, Castagnola E, Sannia A. Concomitant SARS-CoV-2 infection and severe neurologic involvement in a late-preterm neonate. Neurology. 2020 Nov 3;95(18):834-835. doi: 10.1212/WNL.0000000000010729 pmid: 32934169
104. Díaz CA, Maestro ML, Pumarega MTM, Antón BF, Pallás Alonso CP. Primer caso de infección neonatal por COVID-19 en España. Anales de Pediatria. 2020 Apr 1;92(4):237–8. doi: 10.1016/j.anpede.2020.03.002 pmid: 32363217
105. Díaz-Corvillón P, Mönckeberg M, Barros A, Illanes SE, Soldati A, Nien JK, et al. Routine screening for SARS CoV-2 in unselected pregnant women at delivery. PLoS One. 2020 Sep 29;15(9):e0239887. doi: 10.1371/journal.pone.0239887 pmid: 32991621
106. Dima M, Enatescu I, Craina M, Petre I, Iacob ER, Iacob D. First neonates with severe acute respiratory syndrome coronavirus 2 infection in Romania: Three case reports. Medicine (Baltimore). 2020 Aug 14; 99(33): e21284. doi: 10.1097/MD.0000000000021284 pmid: 32871986
107. Dingom MAN, Sobngwi E, Essiben F, Assiga AN, Wasnyo YF, Ngate A, et al. Maternal and Fetal Outcomes of COVID-19 Pregnant Women Followed Up at a Tertiary Care Unit: A Descriptive Study. Open Journal of Obstetrics and Gynecology. 2020 Oct;10(10):1482–91. doi: 10.4236/ojog.2020.10100135
108. Donadieu D, Fremont G, Scetbun E, Gerin M, Nivose P, Renevier B. Impact du SARS-CoV-2 sur la grossesse. Med Mal Infect. 2020 Sep; 50(6): S103. doi: 10.1016/j.medmal.2020.06.209
109. Dong L, Tian J, He S, Zhu C, Wang J, Liu C, et al. Possible Vertical Transmission of SARS-CoV-2 from an Infected Mother to Her Newborn. JAMA. 2020 May 12;323(18):1846-1848.doi: 10.1001/jama.2020.4621 pmid: 32215581
110. Dorgalaleh A, Baghaipour MR, Tabibian S, Ghazizadeh F, Dabbagh A, Bahoush G, et al. Gastrointestinal bleeding in a newborn infant with congenital factor X deficiency and COVID-19—A common clinical feature between a rare disorder and a new, common infection. Int J Lab Hematol. 2020 Dec;42(6):e277-e279. doi: 10.1111/ijlh.13318 pmid: 32845081
111. Doria M, Peixinho C, Laranjo M, Varejão AM, Silva PT. Covid-19 during pregnancy: A case series from an universally tested population from the north of Portugal. Eur J Obstet Gynecol Reprod Biol. 2020 Jul; 250: 261-262. doi:https://doi.org/10.1016/j.ejogrb.2020.05.029
112. Dos Reis HLB, Boldrini NAT, Caldas JVJ, da Paz APC, Ferrugini CLP, Miranda AE. Severe coronavirus infection in pregnancy: Challenging cases report. *Rev Inst Med Trop Sao Paulo*. 2020;62(July):1-5. doi:10.1590/S1678-9946202062049. PMID: 32667391
113. Dos Santos Beozzo GPN, de Carvalho WB, Krebs VLJ, Gibelli MABC, Zacharias RSB, Rossetto LES, et al. Neonatal manifestations in COVID-19 patients at a Brazilian tertiary center. Clinics (Sao Paulo). 2020 Nov 27;75:e2407.doi: 10.6061/clinics/2020/e2407 pmid: 33263625
114. Douedi S, Albayati A, Alfraji N, Mazahir U, Costanzo E. Successful maternal and fetal outcomes in COVID-19 pregnant women: An institutional approach. *Am J Case Rep*. 2020;21:1-3. doi:10.12659/AJCR.925513. PMID: 32716009

115. Du Y, Wang L, Wu G, Lei X, Li W, Lv J. Anesthesia and protection in an emergency cesarean section for pregnant woman infected with a novel coronavirus: case report and literature review. *J Anesth*. 2020;34(4):613-618. doi:10.1007/s00540-020-02796-PMID: 3291747.
116. Dumitriu D, Emeruwa UN, Hanft E, Liao G v., Ludwig E, Walzer L, et al. Outcomes of Neonates Born to Mothers with Severe Acute Respiratory Syndrome Coronavirus 2 Infection at a Large Medical Center in New York City. JAMA Pediatr. 2021 Feb 1;175(2):157-167. doi: 10.1001/jamapediatrics.2020.4298 pmid: 33044493
117. Egerup P, Olsen LF, Christiansen A-MH, Westergaard D, Severinsen ER, Hviid KVR, et al. Impact of SARS-CoV-2 antibodies at delivery in women, partners and newborns. Obstetrics & Gynecology. 2021 Jan; 137(1):49-55. doi: 10.1097/AOG.0000000000004199
118. Eghbalian F, Esfahani AM, Jenabi E. COVID-19 Virus in a 6-Day-Old Girl Neonate: A Case Report. Clin Pediatr (Phila). 2020 Dec;59(14):1288-1289. doi: 10.1177/0009922820946010 pmid: 32698615
119. Elenga N, Marie-Josephine W, Siban J, Nacher M, Demar M. Neonatal COVID-19 in French Guiana, a Case-Control study. Research Square. 2021 Jan 18. doi:10.21203/rs.3.rs-147493/v1
120. Elkafrawi D, Joseph J, Schiattarella A, Rodriguez B, Sisti G. Intrauterine transmission of COVID-19 in pregnancy: Case report and review of literature. *Acta Biomed*. 2020;91(3):1-5. doi:10.23750/abm.v91i3.9795. PMID: 32182347.
121. Hijona Elósegui JJ, Carballo García AL, Fernández Risquez AC, Bermúdez Quintana M, Expósito Montes JF. Does the maternal-fetal transmission of SARS-CoV-2 occur during pregnancy *Rev Clin Esp*. 2021;221(2):93-96. doi:10.1016/j.rce.2020.06.001.? PMID: 32546368
122. Elhalik M, Dash S, EL-Atawi K, Mahfouz R, Mabrouk A, D'Souza D, et al. Clinical profile of neonates delivered from mothers with confirmed COVID-19 infection: An experience from a Tertiary Perinatal Care Center in Dubai, UAE. Journal of Pediatrics & Neonatal Care. 2020 Nov 11;10(4). doi:10.15406/jpnc.2020.10.00427
123. Facchetti F, Bugatti M, Drera E, Tripodo C, Sartori E, Cancila V, et al. SARS-CoV2 vertical transmission with adverse effects on the newborn revealed through integrated immunohistochemical, electron microscopy and molecular analyses of Placenta. EBioMedicine. 2020 Sep;59:102951. doi: 10.1016/j.ebiom.2020.102951 pmid: 32818801
124. Falcão MB, de Góes Cavalcanti LP, Filgueiras Filho NM, Antunes de Brito CA. Case report: Hepatotoxicity associated with the use of hydroxychloroquine in a patient with COVID-19. Am J Trop Med Hyg. 2020 Jun;102(6):1214-1216. doi: 10.4269/ajtmh.20-0276. pmid: 32314698
125. Fan C, Lei D, Fang C, et al. Perinatal Transmission of 2019 Coronavirus Disease-Associated Severe Acute Respiratory Syndrome Coronavirus 2: Should We Worry? *Clin Infect Dis*. 2021;72(5):862-864. doi:10.1093/cid/ciaa226. PMID: 32430561
126. Fang NZ, Castaño PM, Davis A. A hospital-based COVID-19 abortion case in the early phase of the pandemic. Contraception. 2020;102(2):137-138. doi: 10.1016/j.contraception.2020.05.005. PMID: 32416144
127. Farghaly MAA, Kupferman F, Castillo F, Kim RM. Characteristics of Newborns Born to SARS-CoV-2-Positive Mothers: A Retrospective Cohort Study. Am J Perinatol. 2020 Nov;37(13):1310-1316. doi: 10.1055/s-0040-1715862 pmid: 32882743
128. Farhadi R, Mehrpisheh S, Ghaffari V, Haghshenas M, Ebadi A. Clinical course, radiological findings and late outcome in preterm infant with suspected vertical transmission born to a mother with severe COVID-19 pneumonia: a case report. *J Med Case Rep*. 2021;15(1):1-5. doi:10.1186/s13256-021-02835-0. PMID: 33892788
129. Farhat A, Eshkil S, Bolourian M, Mohamadzadeh A, Saeedi R. A 900-Gram Preterm Infant with Coronavirus (COVID-19) Infection: A Case Report. J Cardiothorac Med. 2020; 8(4):718-22. doi: 10.22038/JCTM.2020.52621.1298
130. Farhat AS, Sayedi SJ, Akhlaghi F, Hamedi A, Ghodsi A. Coronavirus (COVID-19) Infection in Newborns. Int. J. Pediatr. 2020 Jun; 8(6): 11513-17. doi: 10.22038/IJP.2020.48004.3871
131. Fashner J, Cintron C. Nine SARS-CoV-2 Positive Pregnant Women and Their Infant Delivery Outcomes. *Cureus*. 2020;12(12):10-13. doi:10.7759/cureus.11946. PMID: 33425525
132. Faure-Bardon V, Isnard P, Roux N, et al. Protein expression of angiotensin-converting enzyme 2, a SARS-CoV-2-specific receptor, in fetal and placental tissues throughout gestation: new insight for perinatal counseling. *Ultrasound Obstet Gynecol*. 2021;57(2):242-247. doi:10.1002/uog.22178. PMID: 32798244.
133. Fenizia C, Biasin M, Cetin I, Vergani P, Mileto D, Spinillo A, et al. IN-UTERO MOTHER-TO-CHILD SARS-CoV-2 TRANSMISSION: viral detection and fetal immune response. medRxiv 2020.07.09.20149591; doi: https://doi.org/10.1101/2020.07.09.20149591

134. Ferraiolo A, Barra F, Kratochwila C, Paudice M, Vellone VG, Godano E, et al. Report of positive placental swabs for sars-cov-2 in an asymptomatic pregnant woman with covid-19. Medicina (Kaunas). 2020 Jun; 56(6): 306. doi: 10.3390/medicina56060306 pmid: 32580461
135. Ferrazzi E, Frigerio L, Savasi V, Vergani P, Prefumo F, Barresi S, et al. Mode of Delivery and Clinical Findings in COVID-19 Infected Pregnant Women in Northern Italy. SSRN Electronic Journal. 2020 Apr 8. Available at http://dx.doi.org/10.2139/ssrn.3562464
136. Figueiredo de Montalvao Franca AP, do Vale Pereira D, Valéria Rodrigues E, Nunes Vieira F, Santos Machado K, Aleixo Nogueira P et al. Severe COVID-19 in cardiopath young pregnant without vertical transmission: a case report. 2020. doi: 10.21203/rs.3.rs-40095/v1
137. Figueiredo R, Tavares S, Moucho M, Ramalho C. Systematic screening for SARS-CoV-2 in pregnant women admitted for delivery in a Portuguese maternity. J. Perinat. Med. 2020 doi: 10.1515/jpm-2020-0387
138. Filimonovic D, Lackovic M, Karadzov Orlic N, Djukic V, Pesi Stevanovic I, Mihajlovic S. Intrauterine transfusion in COVID-19 positive mother vertical transmission risk assessment. European Journal of Obstetrics & Gynecology and Reproductive Biology. 2020 doi: 10.1016/j.ejogrb.2020.07.039 03
139. Flannery D, Gouma S, Dhudasia M, Mukhopadhyay S, Pfeifer M, Woodford E, et al. Transplacental Transfer of SARS-CoV-2 Antibodies. medRxiv Preprint. 2020 Oct 11. doi:https://doi.org/10.1101/2020.10.07.20207480
140. Flores-Pliego A, Miranda J, Vega-Torreblanca S, Valdespino-Vázquez Y, Helguera-Repetto C, Espejel-Nuñez A, et al. Molecular Insights into the Thrombotic and Microvascular Injury in Placental Endothelium of Women with Mild or Severe COVID-19. Cells. 2021 Feb 10;10(2):364. doi: 10.3390/cells10020364 pmid: 33578631
141. Forera-Pena DA, Rodriguez MI, Flora-Noda DM, Maricto AL, Velasquez VL, Soto LM et al. The first pregnant woman with COVID-19 in Venezuela: Pre-symptomatic transmission. Travel Medicine and Infectious Disease. 2020. doi: 10.1016/j.tmaid.2020.101805
142. Fragoso DC, Mars C, Dutra BG, da Silva CJ, da Slva PM, Martins Maia Junior AC et al. Covid-19 As A Cause Of Acute Neonatal Encephalitis And Cerebral Cytotoxic Edema. The Pediatric Infectious Disease Journal. 2021. DOI: 10.1097/INF.0000000000003145
143. Gao J, Hu X, Sun X, Luo X, Chen L. Possible intrauterine SARS-CoV-2 infection: Positive nucleic acid testing results and consecutive positive SARS-CoV-2-specific antibody levels within 50 days after birth. Int J Infect Dis. 2020 Oct;99:272-275. doi: 10.1016/j.ijid.2020.07.063 pmid: 32771637
144. Gao J, Li W, Hu X, Wei Y, Wu J, Luo X et al. Disappearance of SARS-CoV-2 Antibodies in Infants Born to Women with COVID-19, Wuhan, China. Emerg Infect Dis. 2020 Oct;26(10):2491-2494.doi: 10.3201/eid2610.202328. doi: 10.3201/eid2610.202328 pmid: 32620180
145. Gao X, Wang S, Zeng W, Chen S, Wu J, Lin X, et al. Clinical and immunologic features among COVID-19–affected mother–infant pairs: antibodies to SARS-CoV-2 detected in breast milk. New Microbes New Infect. 2020 Sep;37:100752. doi: 10.1016/j.nmni.2020.100752 pmid: 32904990
146. Garcia-Manau P, Garcia-Ruiz I, Rodo C, Sulleiro E, Maiz N, Catalan M et al. Fetal Transient Skin Edema in Two Pregnant Women With Coronavirus Disease 2019 (COVID-19). Obstetrics & Gynecology. 2020;00:1–5. DOI: 10.1097/AOG.0000000000004059
147. Garcia-Ruiz I, Sulleiro E, Serrano B, Fernandez-Buhigas I, Rodriguez-Gomez L, Sanchez-Nieves D et al. Congenital infection of SARS-CoV-2 in live-born neonates: a population-based descriptive study. Clinical Microbiology and Infection. doi: 10.1016/j.cmi.2021.06.016.
148. Gaunt P, Ahmed I, Geethanath R, Abu-Harb M, Onwuneme C. Transmission of SARS-CoV-2 to premature twins from an asymptomatic mother. Case Reports in Perinatal Medicine. 2020 Nov 18;9(1). doi: https://doi.org/10.1515/crpm-2020-0064
149. Ghema K, Lehlimi M, Toumi H, Badre A, Chemsi M, Habzi A, et al. Outcomes of newborns to mothers with COVID-19. Infect Dis Now. 2021 Mar 17;S2666-9919(21)00065-8. doi: 10.1016/j.idnow.2021.03.003 pmid: 33748807
150. Gonzalez MR, Villarreal Morales ML, Rodriguez AN, Tapia ES, Cacho WR, Maldonado MO. Third trimester pregnancy and cesarean delivery of a patient with COVID-19: a case report. 2020. DOI: 10.21203/rs.3.rs-29614/v1
151. Gonzalez R, Correa P, Orchard F, Sumonte R, Vial MT, Bitar P e al. COVID-19 and pregnancy: Clinical case of critical presentation, placental inflammation and demonstrated fetal vertical transmission. 2019, Revista Médica Clínica Las Condes. 0716-8640 doi: 10.1016/j.rmclc.2020.12.011
152. Gordon M, Kagalwala T, Rezk K, Rawlingson C, Ahmed MI, Guleri A. Rapid systematic review of neonatal COVID-19 including a case of presumed vertical transmission. BMJ Paediatr Open. 2020 May 25;4(1). doi: 10.1136/bmjpo-2020-000718
153. Gorodezky TM, Amin JM. Interruption of pregnancy in times of COVID-19, Carlos Van Buren Hospital, Valparaiso: about 6 clinical cases. 2020, Rev Chil Obstet Ginecol.

154. Grechukhina O, Greenberg V, Lundsberg LS, Deshmukh U, Cate J, Lipkind HS, et al. Coronavirus disease 2019 pregnancy outcomes in a racially and ethnically diverse population. Am J Obstet Gynecol MFM. 2020 Nov;2(4):100246.doi: 10.1016/j.ajogmf.2020.100246 pmid: 33047100
155. Gregorio-Hernández R, Escobar-Izquierdo AB, Cobas-Pazos J, Martínez-Gimeno A. Point-of-care lung ultrasound in three neonates with COVID-19. Eur J Pediatr. 2020 Jun 5:1–7. doi: 10.1007/s00431-020-03706-4 pmid: 32504135
156. Griffin I, Benarba F, Peters C, Oyelese Y, Murphy T, Contreras D, et al. The Impact of COVID-19 Infection on Labor and Delivery, Newborn Nursery, and Neonatal Intensive Care Unit: Prospective Observational Data from a Single Hospital System. Am J Perinatol. 2020 Aug;37(10):1022-1030. doi: 10.1055/s-0040-1713416 pmid: 32534458
157. Grimminck K, Santegoets LAM, Siemens FC, Fraaij PLA, Reiss IKM, Schoenmakers S. et al. BMJ Case Rep 2020;13:e235581. doi:10.1136/bcr-2020- 235581
158. Groß R, Conzelmann C, Müller JA, Stenger S, Steinhart K, Kirchhoff, et al. Detection of SARS-CoV-2 in human breastmilk. 2020 Jun 6; 395(10239):1757-58. doi: https://doi.org/10.1016/ S0140-6736(20)31209-5
159. Grundman JB, Persky RW, Shanker RK. Late-Onset Neonatal Hypocalcemia Due to Transient Hypoparathyroidism in Infant of Mother With COVID19 at Delivery. J Endocrine Soc 2021;5:e1. m https://academic.oup.com/jes/article/5/Supplement_1/A204/6240516
160. Gu, Z., Duan, Z., Liu, M., et al. Experience of infection protection during cesarean section of pregnant women with new coronavirus pneumonia. *China Family Planning and Obstetrics and Gynecology*. 2020;12:93–95. doi: 10.3969/j.issn.1674-4020.2020.02.25
161. Gulersen M, Prasannan L, Tam Tam H, Metz CN, Rochelson B, Meirowitz N, et al. Histopathologic evaluation of placentas after diagnosis of maternal severe acute respiratory syndrome coronavirus 2 infection. Am J Obstet Gynecol MFM. 2020 Nov;2(4):100211. doi: 10.1016/j.ajogmf.2020.100211 pmid: 32838277
162. Gulersen M, Staszewski C, Grayver E, Tam Tam H, Gottesman E, Isseroff D et al. Coronavirus Disease 2019 (COVID-19)–Related Multisystem Inflammatory Syndrome in a Pregnant Woman. 2020, Obstet Gynecol 2020;00:1–5. DOI: 10.1097/AOG.0000000000000425
163. Gupta A, Malhotra Y, Patil U, Muradas AR, Lee WT, Krammer F, et al. In Utero Vertical Transmission of Coronavirus Disease 2019 in a Severely Ill 29-week Preterm Infant. AJP Rep. 2020 Jul;10(3):e270-e274. doi: 10.1055/s-0040-1715177 pmid: 33094016
164. Hachem R, Marku GA, Veluppillai C, Poncelet C. et al., Late miscarriage as a presenting manifestation of COVID-19, Eur J Obstet Gynecol (2020), doi: 10.1016/j.ejogrb.2020.07.024
165. Hadar E, Dollinger S and Wiznitzer A. Coronavirus disease and vaccination during pregnancy and childbirth: a review of the Israeli perspective and experience. The Journal of Maternal-Fetal & Neonatal Medicine. doi: 10.1080/14767058.2021.1937110
166. Halici-Oztruk F, Ocal FD, Aydin S, Tanacan A, Ayhan SG, Altinhboga O et al. Investigating the risk of maternal-fetal transmission of SARS-CoV-2 in early pregnancy. 2021, Placenta. 0143-4004. doi: 10.1016/j.placenta.2021.02.006
167. Han MS, Seong MW, Heo EY, Park JH, Kim N, Shin S, et al. Sequential Analysis of Viral Load in a Neonate and Her Mother Infected With Severe Acute Respiratory Syndrome Coronavirus 2. Clin Infect Dis. 2020 Nov 19;71(16):2236-2239. doi: 10.1093/cid/ciaa447 pmid: 32297925
168. Hansen JN, Hine J, Strout TD. COVID-19 and preeclampsia with severe features at 34-weeks gestation. American Journal of Emergency Medicine (2020). doi: 10.1016/j.ajem.2020.06.052
169. Hansra R, Hankins M, Nasim M. Safety of Remdesivir and Inhaled Nitric Oxide in a Pregnant Patient With Twins: A Case Report. 2021, Critical Care Medicine. 49;1:8. doi: 10.1097/01.ccm.0000726088.11325.8b
170. Hantoushzadeh S, Shamshirsaz AA, Aleyasin A, Seferovic MD, Aski SK, Arian SE, et al. Maternal death due to COVID-19. Am J Obstet Gynecol. 2020 Jul;223(1):109.e1-109.e16. doi: 10.1016/j.ajog.2020.04.030 pmid: 32360108
171. Harahap A, Harianto A, Etika R, Utomo MT, Angelika D, Handayani KD et al. Spontaneous Ileum Perforation in a premature twin with Coronavirus-19 positive mother. Journal of Pediatric Surgery Case Reports (2021) doi: 10.1016/j.epsc.2021.101807.
172. Hascoët JM, Jellimann JM , Hartard C, Wittwer A , Jeulin H, Franck P, et al. Case Series of COVID-19 Asymptomatic Newborns With Possible Intrapartum Transmission of SARS-CoV-2. Front. Pediatr. 2020 Sep 29; 8:632. doi: https://doi.org/10.3389/fped.2020.568979
173. Hassan N, Muzamil M, Banday D. COVID-19 infection during pregnancy - maternal and perinatal outcomes: a tertiary care centre study. Int J Reprod Contracept Obstet Gynecol. 2020Sep;9(9):3764-3769. doi:http://dx.doi.org/10.18203/2320-1770.ijrcog20203853

174.Haye MT, Cartes G, Gutiérrez J, Ahumada P, Krause B, Merialdi M et al. Maternal and perinatal outcomes in pregnant women with confirmed severe and mild COVID-19 at one large maternity hospital in Chile. 2021, The Journal of Maternal-Fetal & Neonatal Medicine. DOI: 10.1080/14767058.2021.1902498
175.Hazari K, Abdeldayem R, Paulose L, Kurien N, Almahloul Z, Mohammad H, et al. Covid-19 infection in pregnant women in Dubai: A case-control study. Research Square. 2021 Jan 27. doi: 10.21203/rs.3.rs-149240/v1
176.Hcini N, Maamri F, Picone O, Carod JF, Lambert V, Mathieu M, et al. Maternal, fetal and neonatal outcomes of large series of SARS-CoV-2 positive pregnancies in peripartum period: A single-center prospective comparative study. Eur J Obstet Gynecol Reprod Biol. 2021 Feb;257:11-18. doi: 10.1016/j.ejogrb.2020.11.068 pmid: 33310656
177.He M, Skaria P, Kreutz K, Chen L, Hagemann I, Carter EB. et al. Histopathology of Third Trimester Placenta from SARS-CoV-2-Positive Women, 2020. doi: 10.1101/2020.08.11.20173005.
178.Hecht, J.L., Quade, B., Deshpande, V. *et al.* SARS-CoV-2 can infect the placenta and is not associated with specific placental histopathology: a series of 19 placentas from COVID-19-positive mothers. *Mod Pathol*. 2020; 33: 2092–2103 doi: 10.1038/s41379-020-0639-4
179.Hernández OB, Honorato S. M, Silva G MC, Sepúlveda-Martínez Á, Fuenzalida C. J, Abarzúa C. A, et al. COVID 19 y embarazo en Chile: Informe preliminar del estudio multicéntrico GESTACOVID. Rev.chil.obstet.ginecol. 2020; 85; Suplemento Nº1: S75 – S89. doi: http://dx.doi.org/10.4067/S0717-75262020000700011
180.Hinojosa-Velasco A, de Oca PVBM, García-Sosa LE, Mendoza-Durán JG, Pérez-Méndez MJ, Dávila-González E, et al. A case report of newborn infant with severe COVID-19 in Mexico: Detection of SARS-CoV-2 in human breast milk and stool. Int J Infect Dis. 2020 Nov; 100: 21–24. doi: 10.1016/j.ijid.2020.08.055 pmid: 32860950
181.Hopwood AJ, Jordan-Villegas A, Gutierrez LD, Cowart MC, Vega-Montalvo W, Cheung WL, et al. SARS-CoV-2 pneumonia in a newborn treated with remdesivir and COVID-19 convalescent plasma. J Pediatric Infect Dis Soc. 2021 May 28;10(5):691-694. doi: 10.1093/jpids/piaa165 pmid: 33306130
182.Hosier H, Farhadian S, Morotti RA, Deshmukh U, Lu-Culligan A, Campbell KH, et al. SARS-CoV-2 infection of the placenta. J Clin Invest. 2020 Sep 1; 130(9): 4947–4953. doi: 10.1172/JCI139569 pmid: 32573498
183.Hosseini MS, Hosseini A, Ghaffari E, Radfar M, Shirvani F, Tabatabai S, Abtahi D, Mirhadi M, Mortazavi M, Ariana S. Evaluation of clinical outcomes of neonates born to mothers with coronavirus (COVID-19) in Shahid Beheshti Hospitals. J Educ Health Promot. 2021 May;10(1):173. doi: 10.4103/jehp.jehp_20_21. PMID: 34250107.
184.Hsu AL, Guan M, Johannesen E, Stephens AJ, Khaleel N, Kagan N, et al. Placental SARS-CoV-2 in a patient with mild COVID-19 disease. 2020 Aug 4; 93(2):1038-44. doi: 10.1101/2020.07.11.20149344
185.Hu X, Gao J, Wei Y, Chen H, Sun X, Chen J, et al. Managing Preterm Infants Born to COVID-19 Mothers: Evidence from a Retrospective Cohort Study in Wuhan, China. Neonatology. 2020; 117(5):592-598. doi: 10.1159/000509141 pmid: 32799197
186.Huang J, Zhou X, Lu S, Xu Y, Hu J, Huang M et al. Dialectical behavior therapy-based psychological intervention for woman in late pregnancy and early postpartum suffering from COVID-19: a case report. Journal of Zhejiang University-SCIENCE B, 2020. doi: 10.1631/jzus.B2010012
187.Huang Liqun WJ, Chumei X, Dianxing S. Successful treatment of the first pregnancy with severe new coronavirus pneumonia. *Med Pharm J Chin People's Lib Army.* 2020 doi: 10.3969/j.issn.2095-140X.2020.04.001.
188.Huang W, Zhao Z, He Z, Liu S, Wu Q, Zhang X, et al. Unfavorable outcomes in pregnant patients with COVID-19. J Infect. 2020 Aug; 81(2): e99–e101. doi: 10.1016/j.jinf.2020.05.014 pmid: 32417313
189.Huerta Saenz IH, Elias Estrada JC, Campos Del Castillo K, Muñoz Taya R, Cristina Coronado J. Características materno perinatales de gestantes COVID-19 en un hospital nacional de Lima, Perú. Revista Peruana de Ginecología y Obstetricia. 2020 Jun 11;66(2). doi: http://dx.doi.org/10.31403/rpgo.v66i2245
190.Huseynova RA, A. Bin Mahmoud L, Huseynov O, Almalkey M, Amer Almotiri A, Sumaily HH, et al. A neonate born to an infected COVID-19 mother was tested positive just 24 hours after its birth. Clin Case Rep. 2021 Feb 10;9(4):1954-1957. doi: 10.1002/ccr3.3913 pmid: 33821189
191.Iqbal SN, Overcash R, Mokhtari N, Saeed H, Gold S, Auguste T et al. An Uncomplicated Delivery in a Patient with Covid-19 in the United States. The New England Journal of Medicine, 2020.
192.Jacobson J, Antony K, Beninati M, Alward W, Hoppe KK. Use of dexamethasone, remdesivir, convalescent plasma and prone positioning in the treatment of severe COVID-19 infection in pregnancy: A case report. Case Reports in Women's Health, 2021. e00273. doi: 10.1016/j.crwh.2020.e00273.

193. Jafari R, Jonaidi-Jafari N, Dehghanpoor F, Saburi A. Convalescent plasma therapy in a pregnant COVID-19 patient with a dramatic clinical and imaging response: A case report. World J Radiol 2020; 12(7): 137-141. DOI: 10.4329/wjr.v12.i7.137
194. Jain P, Thakur A, Kler N, Garg P. Manifestations in Neonates Born to COVID-19 Positive Mothers. Indian J Pediatr. 2020 Aug;87(8):644. doi: 10.1007/s12098-020-03369-x. pmid: 32504454
195. Jang WK, Lee SY, Park S, Ryoo NH, Hwang I, Park JM et al. Pregnancy Outcome, Antibodies, and Placental Pathology in SARS-CoV-2 Infection during Early Pregnancy. Int. J. Environ. Res. Public Health 2021, 18, 5709. https:// doi.org/10.3390/ijerph18115709
196. Jani S, Jacques SM, Qureshi F, Natarajan G, Bajaj S, Velumula P, et al. Clinical Characteristics of Mother-Infant Dyad and Placental Pathology in COVID-19 Cases in Predominantly African American Population. AJP Rep. 2021 Jan;11(1):e15-e20. doi: 10.1055/s-0040-1721673 pmid: 33542856
197. Janjua BN, Petch S, Akthar Birmani S, Seyal S, Elhassadi E, Azam et al. Vertical transmission, maternal thrombocytopenia, & postpartum haemorrhage in coronavirus infection – a case report. RCOG Virtual World Congress 2021 – Top 500 Abstracts, 2021
198. Janssen O, Thompson M, Milburn S, Green R, Wagner B, Bianco A, et al. The impact of perinatal severe acute respiratory syndrome coronavirus 2 infection during the peripartum period. Am J Obstet Gynecol MFM. 2021 Jan;3(1):100267. doi: 10.1016/j.ajogmf.2020.100267 pmid: 33103098
199. Joseph NT, Dude CM, Verkerke HP, Irby LS, Dunlop AD, Patel RM et al. Maternal Antibody Response, Neutralizing Potency, and Placental Antibody Transfer After Severe Acute Respiratory Syndrome Coronavirus (SARS-CoV-2) Infection. Obstet Gynecol 2021;00:1–9. doi: 10.1097/AOG.0000000000004440.
200. Joshi SD., Vijayalakshmi G, Kavya N, Rajeswari R, Banu R, Jayasakthi. Severe acute respiratory syndrome coronavirus 2 in pregnant women: an observational study. IJRCOG. 2020 Nov 26;9(12):4892. doi:http://dx.doi.org/10.18203/2320-1770.ijrcog20204963
201. Jung J, Hong MJ, Kim EO, Lee J, Kim MN, Kim SH. Investigation of a nosocomial outbreak of COVID-19 in a pediatric ward in South Korea: Successful control by early detection and extensive contact tracing with testing. Clinical Microbiology and Infection, 2020. doi: 10.1016/j.cmi.2020.06.021.
202. Kalafat E, Yassa M, Koc A, Tug N; TULIP collaboration. Utility of lung ultrasound assessment for probable SARS-CoV-2 infection during pregnancy and universal screening of asymptomatic individuals. Ultrasound Obstet Gynecol. 2020 Oct;56(4):624-626. doi: 10.1002/uog.23099. pmid: 32916004.
203. Kalamdani P, Kalathingal T, Manerkar S, Mondkar J. Clinical Profile of SARS-CoV-2 Infected Neonates From a Tertiary Government Hospital in Mumbai, India. Indian Pediatr. 2020 Dec 15;57(12):1143-1146. doi: 10.1007/s13312-020-2070-9 pmid: 33043888
204. Kamali A, Mahmodiyeh B, Almasi-Hashiani A, Mousavi-Hasanzadeh M, Shokrpour M. Epidemiological study of COVID-19 pneumonia in pregnant woman and their neonates; report of thirteen confirmed COVID-19 pregnant women. Eur. J. Mol. Clin. Med. 2020; 7(1):4455-6.
205. Kappanayil M, Balan S, Alawani S, Mohanty S, Leeladharan SP, Gangadharan S et al. Multisystem inflammatory syndrome in a neonate, temporally associated with prenatal exposure to SARS-CoV-2: a case report. Lancet Child Adolesc Health, 2021. doi: 10.1016/ S2352-4642(21)00055-9
206. Karade S, Vishal AK, Sen S, Bewal N, Gupta RM. Probable vertical transmission of severe acute respiratory syndrome coronavirus 2 infection from mother to neonate. Medical Journal Armed Forces India, 2021. doi: 10.1016/j.mjafi.2020.11.026
207. Karasu D, Kilicarslan N, Ozugunay SE, Gurbuz H. Our anesthesia experiences in COVID-19 positive patients delivering by cesarean section: A retrospective single-center cohort study. J. Obstet. Gynaecol. Res. doi: 10.1111/jog.14852.
208. Kayem G, Lecarpentier E, Deruelle P, Bretelle F, Azria E, Blanc J, et al. A snapshot of the Covid-19 pandemic among pregnant women in France. J Gynecol Obstet Hum Reprod. 2020 Sep;49(7):101826. doi: 10.1016/j.jogoh.2020.101826 pmid: 32505805
209. Keklik ESK, Dal H, Bozok S. Cytokine Hemoadsorption in the Management of a Pregnant Woman with COVID-19 Pneumonia: Case Report. SN Comprehensive Clinical Medicine, 2020. doi: 10.1007/s42399-020-00508-5
210. Kelly JC, Raghuraman N, Palanisamy A, Stout MJ, Carter EB. Pre-procedural asymptomatic COVID-19 in obstetric and surgical units, American Journal of Obstetrics and Gynecology (2020), doi: 10.1016/j.ajog.2020.09.023.
211. Kest H, Kaushik A, Skroce L, Bogusz J, Datta-Bhutada S. Rooming-in for well term infants born to asymptomatic mothers with COVID-19. Pediatr. Infect. Dis. J. 2020 Nov 5; 10(1):60-61. doi: https://doi.org/10.1093/jpids/piaa120

212. Khan S, Jun L, Nawsherwan MS, Siddique R, Li Y, Han G et al. Association of COVID-19 with pregnancy outcomes in health-care workers and general women, Clinical Microbiology and Infection. doi: 10.1016/j.cmi.2020.03.034
213. Khan S, Peng L, Siddique R, Nabi G, Nawsherwan MS, Xue M et al. Impact of COVID-19 infection on pregnancy outcomes and the risk of maternal-to-neonatal intrapartum transmission of COVID-19 during natural birth. Infection Control & Hospital Epidemiology, 2020. doi: 10.1017/ice.2020.84
214. Khatib MY, Olagundoye VO, Elshafei MS, El Khatib FM, Mohamed AS, Nashwan AJ. Management considerations for a critically ill 26-gestational week patient with COVID-19: A case report. Clin Case Rep. 2021;00:1–4. doi: 10.1002/ccr3.3886
215. Khorsandi B, Nasirzadeh N, Asadi L. Normal Vaginal Delivery in a Woman with COVID-19. jcbr. 2020; 4 (4) :34-40. DOI: 10.29252/Jcbr.4.4.34
216. Khoury R, Bernstein PS, Debolt C, Stone J, Sutton DM, Simpson LL, et al. Characteristics and outcomes of 241 births to women with severe acute respiratory syndrome coronavirus 2 (SARS-CoV-2) infection at Five New York City Medical Centers. Obstet Gynecol. 2020 Aug;136(2):273-282. doi: 10.1097/AOG.0000000000004025 pmid: 32555034
217. Khushdil A, Ahmed Z, Waqar T, Haque KN, Sultana R, Sughra U et al. Outcome of Neonates Born to Mothers Who Are COVID-19 Positive; An Observation Cohort Study from Pakistan (preprint). SSRN; 2021. doi:10.2139/ssrn.3748372.
218. Kiappe OP, Santos da Cruz HF, Cervii Rosa PA, Arrais L, de Moraes NSB. Ocular Assessments of a Series of Newborns Gestationally Exposed to Maternal COVID-19 Infection. JAMA Ophthalmol, 2021. doi:10.1001/jamaophthalmol.2021.1088
219. Kirtsman M, Diambomba Y, Poutanen SM, Malinowski AK, Vlachodimitropoulou E, Parks WT, et al. Probable congenital SARS-CoV-2 infection in a neonate born to a woman with active SARS-CoV-2 infection. CMAJ. 2020 Jun 15;192(24):E647-E650. doi: 10.1503/cmaj.200821 pmid: 32409520
220. Knight M, Bunch K, Vousden N, Morris E, Simpson N, Gale C, et al. Characteristics and outcomes of pregnant women admitted to hospital with confirmed SARS-CoV-2 infection in UK: National population based cohort study. BMJ. 2020 Jun 8;369:m2107. doi: 10.1136/bmj.m2107 pmid: 32513659
221. Komiazyk M, Aptowicz A, Książek I, Sitkiewicz I, Baraniak A. An asymptomatic carriage of severe acute respiratory syndrome coronavirus 2 by a pregnant woman and her newborn. Pol Arch Intern Med. 2021 Feb 26;131(2):182-183. doi: 10.20452/pamw.15777 pmid: 33491941
222. Konstantinidou A, Papaevangelou V, Tsakris A, Spanakis NE, Syrdiou G, Pergaris A et al. Further placental pathology in mild COVID-19 of term pregnancy with pathophysiological correlates. 2020, DOI: 10.21203/rs.3.rs-40375/v1
223. Korkmaz MF, Türe E, Dorum BA, Kiliç ZB. The epidemiological and clinical characteristics of 81 children with COVID-19 in a pandemic hospital in Turkey: An observational cohort study. J Korean Med Sci. 2020 Jun 29; 35(25): e236. doi: 10.3346/jkms.2020.35.e236 pmid: 32597047
224. Kulkarni R, Rajput U, Dawre R, Valvi C, Nagpal R, Magdum N, et al. Early-onset symptomatic neonatal COVID-19 infection with high probability of vertical transmission. Infection. 2020 Aug 2:1–5. doi: 10.1007/s15010-020-01493-6 pmid: 32743723
225. Kumar VHS, Prasath A, Blanco C, Kenney PO, Ostwald CM, Meyer TS et al. Respiratory Failure in an Extremely Premature Neonate with COVID-19. Children, 2021. 8, 477. doi: 10.3390/children806047
226. Kumar V, Narayanan P, Shetty S Mohammed AP. Lower motor neuron facial palsy in a postnatal mother with COVID-19. BMJ Case Rep, 2021. 14:e240267. doi:10.1136/bcr-2020- 240267
227. Kumari K, Yadav R, Mittra S, Kumar A, Bajpai PK, Srivastava DK, Kumar R. Pregnancy outcomes and vertical transmission capability of SARS-CoV-2 infection among asymptomatic females: A cross-sectional study in a tertiary care rural hospital. J Family Med Prim Care. 2021 Sep;10(9):3247-3251. doi: 10.4103/jfmpc.jfmpc_23_21. PMID: 34760738.
228. L'Huillier AG, Torriani G, Pigny F, Kaiser L, Eckerle I. Culture-Competent SARS-CoV-2 in Nasopharynx of Symptomatic Neonates, Children, and Adolescents. Emerg Infect Dis. 2020 Oct;26(10):2494-2497. doi: 10.3201/eid2610.202403 pmid: 32603290
229. Lang LK, Lang QA. Covid-19 Maternal-Neonate Vertical Transmission Rates in Los Angeles County. J NATL MED ASSOC. 2020 Oct; 112(5):S36-37. doi: https://doi.org/10.1016/j.jnma.2020.09.093.
230. Lee, E. K., Kim, W. D., Lee, D. W., & Lee, S. A. Management of the first newborn delivered by a mother with COVID-19 in South Korea. *Clinical and experimental pediatrics*. 2020; *63*(9), 373–375. https://doi.org/10.3345/cep.2020.00850
231. Léhner G, Krugluger W, Aberle SW, Weseslindtner L, Hartmann B. A Case of a 33- Week Pregnant Woman with COVID-19 and Term Birth. Obstet Gynecol Cases, 2020. Rev 7:168. doi. 10.23937/2377-9004/1410168

232. Lenoci G, Galante D, Ceci E, Manzulli V, Moramarco AM, Chiaromonte A, et al. Sars-CoV-2 isolation from a 10-day-old newborn in Italy: A case report. IDCases. 2020;22:e00960. doi: 10.1016/j.idcr.2020.e00960 pmid: 32963961
233. Levitan D, London V, McLaren RA, Mann JD, Cheng K, Silver M, et al. Histologic and Immunohistochemical Evaluation of 65 Placentas from Women with Polymerase Chain Reaction–Proven Severe Acute Respiratory Syndrome Coronavirus 2 (SARS-CoV-2) Infection. Arch Pathol Lab Med. 2021 Jun 1;145(6):648-656.doi: 10.5858/arpa.2020-0793-SA pmid: 33596304
234. Li, S., Zhang, LX., Fei ANX., He S., Jiang H. Analysis of clinical and laboratory indicators in cases of combined pregnancy with novel coronavirus pneumonia. International Journal of Laboratory Medicine. May 2020; 4(10):1-3. doi: 10.3969/j.issn.1673-4130.2020.09.001
235. Li Y, Zhao R, Zheng S, Chen X, Wang J, Sheng X et al. Lack of Vertical Transmission of Severe Acute Respiratory Syndrome Coronavirus 2, China. Emerging Infectious Diseases, 2020. DOI: 10.3201/eid2606.200287
236. Libbrecht S, Van CLeemput J, Vanderkerckhove L, Colman S, Padalko E, Verhasselt B et al. A rare but devastating cause of twin loss in a near-term pregnancy highlighting the features of severe SARS-CoV2 placentitis. Histopathology. 2021 Oct;79(4):674-676. doi: 10.1111/his.14402. Epub 2021 Jul 8. PMID: 33982813.
237. Lima ARO, Cardoso CC, Bentim PRB, Voloch CM, Rossi ÁD, da Costa RM, et al. Maternal SARS-CoV-2 infection associated to systemic inflammatory response and pericardial effusion in the newborn: a Case-Report. J Pediatric Infect Dis Soc. 2021 Apr 30;10(4):536-539. doi: 10.1093/jpids/piaa133. pmid: 33125068
238. Lima-Rogel V, Villegas-Silva R, Coronado-Zarco A, Estrada-Ruelas I, la Vega ARS de, Muro-Flores RH, et al. Perinatal covid-19: A case report, literature review, and proposal of a national system for case record. Bol Med Hosp Infant Mex. 2021;78(1):34-40. doi: 10.24875/BMHIM.20000230 pmid: 33661873
239. Liu P, Zheng J, Yang P, Wang X, Wei C, Zhang S, et al. The immunologic status of newborns born to SARS-CoV-2–infected mothers in Wuhan, China. J Allergy Clin Immunol. 2020 Jul; 146(1): 101–109.e1. doi: 10.1016/j.jaci.2020.04.038 pmid: 32437740
240. Lizama O, Mucha J, Chincarol MC, Giraldo G, Salazar J, Aguero K et al. Epidemiological, clinical, pre- and postnatal characteristics of neonates born to mothers with Covid-19, and follow-up up to 14 days post-discharge, in Lima-Peru. Rev Med Hered. 2021; 32:5-11. doi: 10.20453/rmh.v32i1.3942.
241. Llorca J, Lechosa-Muñiz C, Gortázar P, Fernández-Ortiz M, Jubete Y, Cabero MJ, et al. COVID-19 in a cohort of pregnant women and their descendants. Cohort profile in the MOACC-19 study. medRxiv. 2020 Aug 20. doi: https://doi.org/10.1101/2020.08.20.20178657
242. Lokken EM, Huebner EM, Taylor GG, Hendrickson S, Vanderhoeven J, Kachikis A, et al. Disease severity, pregnancy outcomes, and maternal deaths among pregnant patients with severe acute respiratory syndrome coronavirus 2 infection in Washington State. Am J Obstet Gynecol. 2021 Jan 27;225(1):77.e1-77.e14. doi: 10.1016/j.ajog.2020.12.1221 pmid: 33515516
243. Lopian M, Kashani-Ligumsky L, Czeiger S, Cohen R, Schindler Y, Lubin D, et al. Safety of vaginal delivery in women infected with COVID-19. Pediatr Neonatol. 2021 Jan;62(1):90-96. doi: 10.1016/j.pedneo.2020.10.010. pmid: 33218936
244. Lowe B, Bopp B. Covid-19 Vaginal Delivery – A Case Report. Aust N Z J Obstet Gynaecol. 2020 Jun;60(3):465-466. doi: 10.1111/ajo.13173. PMID: 32294229
245. Lu D, Sang L, Du S, Li T, Chang Y, Yang XA. Asymptomatic COVID-19 infection in late pregnancy indicated no vertical transmission. J Med Virol. 2020;1–5. doi: 10.1002/jmv.25927
246. Lugli, L., Bedetti, L., Lucaccioni, L. et al. An Uninfected Preterm Newborn Inadvertently Fed SARS-CoV-2–Positive Breast Milk. *Pediatrics*. 2020; *146*(6). https://doi.org/10.1542/peds.2020-004960
247. Luo, Q., Yao, D., Xia, L. et al. Characteristics and Pregnancy Outcomes of Asymptomatic and Symptomatic Women with COVID-19: Lessons from Hospitals in Wuhan. *The Journal of Infection in Developing Countries*. 2021; *15*(04), 463–469.
248. Machado S, de Sousa Tavares W, de Quental I, Lopes J, Cruz A, Feitosa P et al. Analysis of Placenta with SARS-CoV-2 and Fetal Death: Case Report. Journal of Psychiatry Research Reviews & Reports. 2021 Jan 6; 3(1):2-4. doi: https://doi.org/10.47363/JPSR/2021(3)114.
249. Mahajan NN, Ansari M, Gaikwad C, Jadhav P, Tirkey D, Pophalkar MP, et al. Impact of SARS-CoV-2 on multiple gestation pregnancy. International Journal of Gynecology and Obstetrics. 2021 Feb 1;152(2):220–5. doi: https://doi.org/10.1002/ijgo.13508
250. Majachani N, Francois JLM, Fernando AK, Zuberi J. A case of a newborn baby girl infected with SARS-CoV-2 due to transplacental viral transmission. Am J Case Rep. 2020 Oct 25; 21: e925766-1–e925766-5. doi: 10.12659/AJCR.925766 pmid: 33099570

251. Makwe, C. C., Okunade, K. S., Rotimi, M. K., Ekor, O. E., Oyeleke, O. G., Bello, Q. O., Oluwole, A. A., Akase, I. E., Ezenwa, B. N., Fajolu, I. B., Dada, R. W., Oshodi, Y., Olatosi, J. O., Opanuga, O. O., Omilabu, S., Ezeaka, V. C., & Afolabi, B. B. Caesarean delivery of first prediagnosed COVID-19 pregnancy in Nigeria. *Pan African Medical Journal*, 2020; *36*. Doi: https://doi.org/10.11604/pamj.2020.36.100.23892
252. Malik S, Surve S, Wade P, Kondekar S, Sawant V, Shaikh M, Kuppusamy P, Bharmal R, Mahale SD, Modi DN, Gajbhiye RK. Clinical Characteristics, Management, and Short-Term Outcome of Neonates Born to Mothers with COVID-19 in a Tertiary Care Hospital in India. J Trop Pediatr. 2021 Jul 2;67(3):fmab054. doi: 10.1093/tropej/fmab054. PMID: 34114628.
253. Malshe N, Patnaik SK, Lalwani S, Suryawanshi P, Kulkarni R, Mhaske S, Mishra AC, Arankalle V. Perinatal transmission of SARS-CoV-2 and transfer of maternal IgG/neutralizing anti-SARS-CoV-2 antibodies from mothers with asymptomatic infection during pregnancy. Infection. 2021;(0123456789). doi:10.1007/s15010-021-01650-5. PMID: **34232457**
254. Mand N, Iannaccone A, Longardt AC, Hutten M, Mense L, Oppelt P, Maier RF, Pecks U, Rüdiger M; CRONOS Network; CRONOS-Network. Neonatal outcome following maternal infection with SARS-CoV-2 in Germany: COVID-19-Related Obstetric and Neonatal Outcome Study (CRONOS). Arch Dis Child Fetal Neonatal Ed. 2021 Jul:fetalneonatal-2021-322100. doi: 10.1136/archdischild-2021-322100. PMID: 34281935.
255. Maraschini A, Corsi E, Salvatore MA, Donati S. Coronavirus and birth in Italy: results of a national population-based cohort study. Ann Ist Super Sanita. 2020 Jul-Sep;56(3):378-389. doi:10.4415/ANN_20_03_17 pmid: 32959805
256. Marín Gabriel MA, Cuadrado I, Álvarez Fernández B, González Carrasco E, Alonso Díaz C, Llana Martín I, et al. Multicentre Spanish study found no incidences of viral transmission in infants born to mothers with COVID-19. Acta Paediatr. 2020 Nov;109(11):2302-2308. doi: 10.1111/apa.15474 pmid: 32649784
257. Marín Gabriel MA, Reyne Vergeli M, Caserío Carbonero S, Sole L, Carrizosa Molina T, Rivero Calle I, et al. Maternal, Perinatal and Neonatal Outcomes with COVID-19: A Multicenter Study of 242 Pregnancies and Their 248 Infant Newborns during Their First Month of Life. Pediatr Infect Dis J. 2020 Dec;39(12):e393-e397 pmid: 32947599
258. Filimonovic, D., Lackovic, M., Filipovic, I., Orlic, N. K., Markovic, V. M. et al. Intrauterine transfusion in COVID-19 positive mother vertical transmission risk assessment. *European Journal of Obstetrics & Gynecology and Reproductive Biology*. 2020; *252*, 617–618. https://doi.org/10.1016/j.ejogrb.2020.07.039
259. Martenot A, Labbassi I, Delfils-Stern A, Monroy O, Langlet C, Pichault-Klein V, et al. Favorable outcomes among neonates not separated from their symptomatic SARS-CoV-2-infected mothers. Pediatr Res. 2020 Nov 3;1-4. doi: 10.1038/s41390-020-01226-3 pmid: 33144706
260. Martínez-Perez O, Vouga M, Cruz Melguizo S, Forcen Acebal L, Panchaud A, Muñoz-Chápuli M, et al. Association between Mode of Delivery among Pregnant Women with COVID-19 and Maternal and Neonatal Outcomes in Spain. JAMA. 2020 Jul 21;324(3):296-299. doi: 10.1001/jama.2020.10125 pmid: 32511673
261. Marton, T., Hargitai, B., Hunter, K., Pugh, M., & Murray, P. Massive Perivillous Fibrin Deposition and Chronic Histiocytic Intervillositis a Complication of SARS-CoV-2 Infection. *Pediatric and Developmental Pathology*. 2021; *24*(5), 450–454. https://doi.org/10.1177/10935266211020723
262. Masmejan S, Pomar L, Favre G, Panchaud A, Giannoni E, Greub G, et al. Vertical transmission and materno-fetal outcomes in 13 patients with coronavirus disease 2019. Clin Microbiol Infect. 2020 Nov; 26(11): 1585–1587. doi: 10.1016/j.cmi.2020.06.035 pmid: 32652239
263. Mattar CN, Kalimuddin S, Sadarangani SP, Tagore S, Thain S, Thoon KC, et al. Pregnancy Outcomes in COVID-19: A Prospective Cohort Study in Singapore. Ann Acad Med Singap. 2020 Nov;49(11):857-869. doi: 10.47102/annals-acadmedsg.2020437. pmid: 33381779
264. Mattern J, Vauloup-Fellous C, Zakaria H, Benachi A, Carrara J, Letourneau A, et al. Post lockdown COVID-19 seroprevalence and circulation at the time of delivery, France. PLoS One. 2020; 15(10): e0240782. doi: 10.1371/journal.pone.0240782 pmid: 33057392
265. McCarty, K. L., Tucker, M., Lee, G., & Pandey, V. Fetal Inflammatory Response Syndrome Associated With Maternal SARS-CoV-2 Infection. *Pediatrics*. 2021; *147*(4). doi: 10.1542/peds.2020-010132
266. McCoy, J. A., Short, W. R., Srinivas, S. K., Levine, L. D., & Hirshberg, A. Compassionate use of remdesivir for treatment of severe coronavirus disease 2019 in pregnant women at a United States academic center. *American Journal of Obstetrics & Gynecology MFM*. 2020; *2*(3), 100164. https://doi.org/10.1016/j.ajogmf.2020.100164

267. McDevitt KEM, Ganjoo N, Mlangeni D, Pathak S. Outcome of universal screening of neonates for COVID-19 from asymptomatic mothers. J Infect. 2020 Sep; 81(3): 452–482. doi: 10.1016/j.jinf.2020.06.037 pmid: 32565072
268. Mehta H, Ivanovic S, Cronin A, VanBrunt L, Mistry N, Miller R, et al. Novel coronavirus-related acute respiratory distress syndrome in a patient with twin pregnancy: A case report. Case Rep Womens Health. 2020 Jul; 27:e00220. doi: 10.1016/j.crwh.2020.e00220 pmid: 32426242
269. Mei-Dan, E., Satkunaratnam, A., Cahan, T., Leung, M., Katz, K., & Aviram, A. (2021). Questionnaire-based vs universal PCR testing for SARS-CoV-2 in women admitted for delivery. *Birth*, *48*(1), 96–103. https://doi.org/10.1111/birt.12520
270. Mendoza-Hernández, M., Huerta-Niño de Rivera, I., Yoldi-Negrete, M., Saviñon-Tejeda, P., Franco-Cendejas, R., López-Jácome, L. E., & Navarro-Castellanos, I. Probable Case of Vertical Transmission of SARS-CoV-2 in a Newborn in Mexico. *Neonatology*. 2021; *118*(3), 364–367. https://doi.org/10.1159/000514710
271. Menter T, Mertz KD, Jiang S, Chen H, Monod C, Tzankov A, et al. Placental Pathology Findings during and after SARS-CoV-2 Infection: Features of Villitis and Malperfusion. Pathobiology 2021 Jan;88(1):69-77. doi: 10.1159/000511324 pmid: 32950981
272. Michel AS, de Logiviere V, Schnuriger A, Lefebvre M, Maisonneuve E, Kayem G. Description of a late miscarriage case at 16 Weeks of Gestation associated with a SARS-CoV-2 infection. J Gynecol Obstet Hum Reprod. 2021 Mar;50(3):102064. doi: 10.1016/j.jogoh.2021.102064 pmid: 33453449
273. Mirahmadizadeh A, Borazjani R, Ebrahimi M, Haghighi LH, Kamali K, Hamzavi SS, et al. COVID-19 presented with gastrointestinal manifestations in an 11-days-old neonate: A case report and review of the literature. Arch Pediatr Infect Dis. 2020 Jul 11;8(3):1–3. doi:10.5812/pedinfect.104508
274. Moeindarbary S, Pourhoseini SA, Layegh P, Shahriari Z, Fayyaz F, Bahrami M, et al. Two infants with COVID-19 acquired from infected mothers: The incompatibility of maternal intensity and infant lung involvement: A case report. Research Square. 2020 Jun 22 (Preprint). doi: 10.21203/rs.3.rs-37088/v1
275. Mohaghegh Z, Abedi P, Taghizdeh Z, Leyli EK, Valiani FH, Kasvaie MS, Tavakolikia N. Incidence Rate of COVID-19 and Pregnancy Outcomes in Women Affected With COVID-19: A Cross-Sectional Study in Tehran, Iran. The Lancet Preprint. 2021 Apr. doi: 10.2139/ssrn.3831848

276. Molina EO, Pailos RH, Guillen MP, Pedreno AP, Rodriguez ER, Martinez AH. Covid-19 Infection in Symptomatic Pregnant Women at the Midpoint of the Pandemic in Spain: A Retrospective Analysis. Ginekol Pol. 2020;91(12):755-763. doi: 10.5603/GP.a2020.0130 pmid: 33447995
277. Moreira LMO, Patiño J, Oliveira PR de, Costa MHM, Santiago AC, Campos GS, Sardi SI, Menezes C, Lyra PPR, Netto EM. SARS-CoV-2 infection in pregnant women and newborns in two maternity hospitals in Salvador-Bahia, Brazil. Brazilian J Infect Dis. 2021;25(3):101591. doi:10.1016/j.bjid.2021.101591. PMID: 34133953.
278. Morhart P, Mardin C, Rauh M, Jüngert J, Hammersen J, Kehl S, Schuh W, Hermes K, Schneider M, Hein A, Woele J, Schneider H. Microphthalmia and Optic Nerve Hypoplasia Induced by Maternal Coronavirus Infection? Research Square. 2021:1-11. doi: 10.21203/rs.3.rs-615203/v1.
279. Morioka I, Toishi S, Kusaka T, Wada K, Mizuno K. Medical care of newborns born to mothers with confirmed or suspected severe acute respiratory syndrome coronavirus 2 infections in Japan/Survey on newborns of SARS-CoV-2 infected mothers. Ped Intl. doi: 10.1111/PED.14855.
280. Mourad M, Jacob T, Sadovsky E, Bejerano S, Salazar-De Simone G, Bagalkot TR et al. Placental response to maternal SARS-CoV-2 infection. Nat Port. (2021) 11:14390. doi: 10.1038/s41598-021-93931-0.
281. Mrazguia, C., Aloui, H., Jaouad, H., & Jaouad, F. SARS-CoV-2 infection in pregnant women: Tunisian series of 11 cases. *The Pan African Medical Journal*. 2020; *37*(Suppl 1), 50. https://doi.org/10.11604/pamj.supp.2020.37.50.27185
282. Murphy C, O'Reilly, McCallion N, Drew R, Ferguson W. Infants Born to Mothers with COVID-19 During Pregnancy: The First Four Months of the Pandemic. Ir Med J. 2020; 113(9):193. Available from: http://imj.ie/infants-born-to-mothers-with-covid-19-during-pregnancy-the-first-four-months-of-the-pandemic/
283. Nakstad, B., Kaang, T., Gezmu, A. M., & Strysko, J. Nosocomial SARS-CoV-2 transmission in a neonatal unit in Botswana: chronic overcrowding meets a novel pathogen. *BMJ Case Reports*. 2021; *14*(6), e242421. https://doi.org/10.1136/bcr-2021-242421
284. Nanavati R, Mascarenhas D, Goyal M, Haribalakrishna A, Nataraj G. A single-center observational study on clinical features and outcomes of 21 SARS-CoV-2-infected neonates from India. Eur J Pediatr. 2021 Jun;180(6):1895-1906. doi: 10.1007/s00431-021-03967-7 pmid: 33544233

285. Narang, K., Szymanski, L. M., Kane, S. v., & Rose, C. H. Acute Pancreatitis in a Pregnant Patient With Coronavirus Disease 2019 (COVID-19). *Obstetrics & Gynecology*. 2021; *137*(3), 431–433. https://doi.org/10.1097/AOG.0000000000004287
286. Naseh, A., & Ashrafzadeh, S. Possible Vertical Transmission From an Unsuspected SARS-CoV-2-Infected Mother to Her Newborn. *Cureus*. 2021. https://doi.org/10.7759/cureus.15717
287. Nayak MK, Panda SK, Panda SS, Rath S, Ghosh A, Mohakud NK. Neonatal outcomes of pregnant women with COVID-19 in a developing country setup. Pediatr Neonatol. 2021;62(5):499-505. doi:10.1016/j.pedneo.2021.05.004. PMID: 34147430
288. Needleman JS, Hanson AE. COVID-19-associated apnea and circumoral cyanosis in a 3-week-old. BMC Pediatr. 2020 Aug 12;20(1):382. doi: 10.1186/s12887-020-02282-8 pmid: 32787956
289. Nederlandse Vereniging Voor Obstetrie en Gynaecologie. Update registratie COVID-19 positieve zwangeren in NethOSS. 2021 Jan 22. Available at: https://www.nvog.nl/actueel/registratie-van-covid-19-positieve-zwangeren-in-nethoss/
290. NG, D. C., Chin, L., Choo, P. P. L., & Paramasivam, U. COVID-19 in a premature infant. *BMJ Case Reports*. 2021; *14*(5), e243783. https://doi.org/10.1136/bcr-2021-243783
291. Ngalame AN, Neng HT, Inna R, Djomo DT, Kamdem DEM, Moustapha B, et al. Materno-Fetal Outcomes of COVID-19 Infected Pregnant Women Managed at the Douala Gyneco-Obstetric and Pediatric Hospital—Cameroon. OJOG. 2020 Sep;10(09):1279–94. doi:10.4236/ojog.2020.1090118
292. Nie R, Wang SS, Yang Q, Fan CF, Liu YL, He WC, et al. Clinical features and the maternal and neonatal outcomes of pregnant women with coronavirus disease 2019. medRxiv. 2020 Mar 27 (Preprint).doi: 10.1101/2020.03.22.20041061
293. Nizyaeva N, Lomova NA, Dolgopolova EL, Petrova U, Karapetyan TE, Shmakov RG. et al. The impact of the novel coronavirus infection COVID-19 on the mother-placenta-fetus system. Bulletin of Russian State Medical University. doi:10.24075/brsmu.2021.020.
294. Norbert L, Treptow A, Schmidt S, Hofmann R, Raumer-Engler M, Heubner G, et al. Neonatal Early-Onset Infection With SARS-CoV-2 in a Newborn Presenting With Encephalitic Symptoms. Pediatr Infect Dis J. 2020 Aug; 39(8):p e212. doi: 10.1097/INF.0000000000002735
295. Norman M, Navér L, Söderling J, Ahlberg M, Askling HH, Aronsson B et al. Association of Maternal SARS-CoV-2 Infection in Pregnancy With Neonatal Outcomes. JAMA. doi: 10.1001/jama.2021.5775.
296. Ogamba I, Kliss A, Rainville N, Panarelli E, Petrini J, Chuang L, et al. Initial review of pregnancy and neonatal outcomes of pregnant women with COVID-19 infection. J Perinat Med. 2020 Nov 3;49(3):263-268. doi: 10.1515/jpm-2020-0446 pmid: 33141109
297. Campodonico Olcese, L., Paredes Salas, J. R., Campodonico Olcese, D., Chang Vargas, C., Acuña Barrueto, L., & Marchena Arias, J. Atención de parto eutócico en gestante con COVID-19 en Lima - Perú. *Revista Peruana de Ginecología y Obstetricia*. 2020; *66*(2). https://doi.org/10.31403/rpgo.v66i2251
298. Olivini N, Calò Carducci FI, Santilli V, de Ioris MA, Scarselli A, Alario D, et al. A neonatal cluster of novel coronavirus disease 2019: clinical management and considerations. Ital J Pediatr. 2020 Dec 7;46, 180. doi:https://doi.org/10.1186/s13052-020-00947-9
299. Omrani A, Almaslamani M, Daghfal J, Alattar R, Elgara M, Shaar S, et al. The First Consecutive 5000 Patients with COVID-19 in Qatar; a Nation-wide Cohort Study. BMC Infect Dis. 2020 Oct 19;20(1):777. doi: 10.1186/s12879-020-05511-8 pmid: 33076848
300. Oncel MY, Akın IM, Kanburoglu MK, Tayman C, Coskun S, Narter F, et al. A multicenter study on epidemiological and clinical characteristics of 125 newborns born to women infected with COVID-19 by Turkish Neonatal Society. Eur J Pediatr. 2021 Mar;180(3):733-742. doi: 10.1007/s00431-020-03767-5 pmid: 32776309
301. Orostizaga A., A., Apablaza R., F., Garrido L., B., Zúñiga F., P., Vaccarezza P., I., & Wittkopf, D. Reporte de caso: Manejo multidisciplinario en gestante con embarazo de 31 semanas y COVID-19 positivo con evolución grave. *Revista Chilena de Obstetricia y Ginecología*. 2020; *85*, S97–S100. https://doi.org/10.4067/S0717-75262020000700013
302. Zarudskaya O, Tsolakian I, Roth J, Bitto A, Khurshid N. Neonatal outcomes of COVID-19 positive mothers. Am J Obstet Gynecol. 2021 Feb; 224(2): S346. doi: 10.1016/j.ajog.2020.12.568
303. Ozer, E., Cagliyan, E., Yuzuguldu, R. I., Cevizci, M. C., & Duman, N. Villitis of unknown etiology in the placenta of a pregnancy complicated by covid-19. *Turkish Journal of Pathology*. 2020. https://doi.org/10.5146/tjpath.2020.01506
304. Ozsurmeli M, Terzi H, Hocaoglu M, Bilir RA, Gunay T, Unsal D, et al. Clinical characteristics, maternal and neonatal outcomes of pregnant women with SARS-CoV-2 infection in Turkey. Bratisl Lek Listy. 2021;122(2):152-157. doi: 10.4149/BLL_2021_023 pmid: 33502885

305. Panichaya, P., Thaweerat, W., & Uthaisan, J. Prolonged viral persistence in COVID-19 second trimester pregnant patient. *European Journal of Obstetrics & Gynecology and Reproductive Biology*. 2020; *250*, 263. https://doi.org/10.1016/j.ejogrb.2020.05.030
306. Paramanathan, S., Kyng, K. J., Laursen, A. L., Jensen, L. D., Grejs, A. M., & Jain, D. COVID-19 with severe acute respiratory distress in a pregnant woman leading to preterm caesarean section: A case report. *Case Reports in Women's Health*. 2021; *30*, e00304. https://doi.org/10.1016/j.crwh.2021.e00304
307. Parsa Y, Shokri N, Jahedbozorgan T, Naeiji Z, Zadehmodares S, Moridi A. Possible Vertical Transmission of COVID-19 to the Newborn; a Case Report. Arch Acad Emerg Med. 2020 Nov 14;9(1):e5. doi: 10.22037/aaem.v9i1.923 pmid: 33313572
308. Patanè L, Morotti D, Giunta MR, Sigismondi C, Piccoli MG, Frigerio L, et al. Vertical transmission of coronavirus disease 2019: severe acute respiratory syndrome coronavirus 2 RNA on the fetal side of the placenta in pregnancies with coronavirus disease 2019-positive mothers and neonates at birth. Am J Obstet Gynecol MFM. 2020 Aug;2(3):100145. doi: 10.1016/j.ajogmf.2020.100145 pmid: 32427221
309. Patberg ET, Adams T, Rekawek P, Vahanian SA, Akerman M, Hernandez A, et al. Coronavirus disease 2019 infection and placental histopathology in women delivering at term. Am J Obstet Gynecol. 2021 Apr;224(4):382.e1-382.e18. doi: 10.1016/j.ajog.2020.10.020 pmid: 33091406
310. Pathak S, Lazarus M, Tiwari A. Short term outcome of neonates born to mothers with SARS-CoV2 infection. IJCP. 2021 Jan;8(1):134-7 doi: http://dx.doi.org/10.18203/2349-3291.ijcp20205519
311. Pawar R, Gavade V, Patil N, Mali V, Girwalkar A, Tarkasband V, Loya S, Chavan A, Nanivadekar N, Shinde R, Patil U, Lakshminrusimha S. Neonatal multisystem inflammatory syndrome (Mis-n) associated with prenatal maternal sars-cov-2: A case series. Children. 2021;8(7):1-15. doi:10.3390/children8070572. PMID: 34356552
312. Pecks, U., Kuschel, B., Mense, L., Oppelt, P., & Rüdiger, M. Pregnancy and SARS-CoV-2 Infection in Germany—the CRONOS Registry. *Deutsches Ärzteblatt International*. 2020. https://doi.org/10.3238/arztebl.2020.0841
313. Pelayo, J., Pugliese, G., Salacup, G., Quintero, E., Khalifeh, A., Jaspan, D., & Sharma, B. Severe COVID-19 in Third Trimester Pregnancy: Multidisciplinary Approach. *Case Reports in Critical Care*. *2020*, 1–8. https://doi.org/10.1155/2020/8889487
314. Penfield CA, Brubaker SG, Limaye MA, Lighter J, Ratner AJ, Thomas KM, et al. Detection of severe acute respiratory syndrome coronavirus 2 in placental and fetal membrane samples. Am J Obstet Gynecol MFM. 2020 Aug;2(3):100133.doi: 10.1016/j.ajogmf.2020.100133 pmid: 32391518
315. Peng, L., Khan, S., Ali, A., Ahmed, S., Ali, L., Han, G., & Jing, Y. Vertical transmission potential of SARS-CoV-2 from infected mother to twin neonates. *Future Virology*. 2021; *16*(6), 379–382. doi: 10.2217/fvl-2020-0324
316. Peng, Z., Wang, J., Mo, Y., Duan, W., Xiang, G., Yi, M., Bao, L., & Shi, Y. Unlikely SARS-CoV-2 vertical transmission from mother to child: A case report. *Journal of Infection and Public Health*. 2020; *13*(5), 818–820. https://doi.org/10.1016/j.jiph.2020.04.004
317. Pérez-Chimal LG, Cuevas GG, Di-Luciano A, Chamartín P, Amadeo G, Martínez-Castellanos MA. Ophthalmic manifestations associated with SARS-CoV-2 in newborn infants: a preliminary report. J AAPOS. 2021 Apr;25(2):102-104. doi: 10.1016/j.jaapos.2020.11.007 pmid: 33601042
318. Perrone, S., Deolmi, M., Giordano, M., D'Alvano, T., Gambini, L., Corradi, M., Frusca, T., Ghi, T., & Esposito, S. Report of a series of healthy term newborns from convalescent mothers with COVID-19. *Acta Bio-Medica : Atenei Parmensis*. 2020; *91*(2), 251–255. doi: 10.23750/abm.v91i2.9743
319. Perrone, S., Giordano, M., Meoli, A., Deolmi, M., Marinelli, F., Messina, G., Lugani, P., Moretti, S., & Esposito, S. Lack of viral transmission to preterm newborn from a COVID-19 positive breastfeeding mother at 11 days postpartum. *Journal of Medical Virology*. 2020; *92*(11), 2346–2347. https://doi.org/10.1002/jmv.26037
320. Pessoa FS, do Vale MS, Marques PF, da Silva Figueira S, da Silva Cruz Salgado IA, de Sousa Wernz Cancian Mochel R. Probable vertical transmission identified within six hours of life. Rev Assoc Med Bras (1992). 2020 Dec;66(12):1621-1624. doi: 10.1590/1806-9282.66.12.1621 pmid: 33331566
321. Piersigilli F, Carkeek K, Hocq C, van Grambezen B, Hubinont C, Chatzis O, et al. COVID-19 in a 26-week preterm neonate. Lancet Child Adolesc Health. 2020 Jun;4(6):476-478. doi: 10.1016/S2352-4642(20)30140-1 pmid: 32386562
322. Piñana, M., Abril, J. F., Andrés, C., Silgado, A., Navarro, A., Suy, A., Sulleiro, E., Pumarola, T., Quer, J., & Antón, A. Viral populations of SARS-CoV-2 in upper respiratory tract, placenta, amniotic fluid and umbilical cord blood support viral replication in placenta. *Clinical Microbiology and Infection*. 2021; *27*(10), 1542–1544. https://doi.org/10.1016/j.cmi.2021.07.008
323. Pineles BL, Alamo IC, Farooq N, Green J, Blackwell SC, Sibai BM, et al. Racial-ethnic disparities and pregnancy outcomes in SARS-CoV-2 infection in a universally-tested cohort in Houston, Texas. Eur J

Obstet Gynecol Reprod Biol. 2020 Nov;254:329-330. doi: 10.1016/j.ejogrb.2020.09.012 pmid: 32950276
324. Pissarra S, Rosário M, Moucho M, Soares H. Perinatal management of SARS-CoV-2 infection in a level III University Hospital. J Matern Fetal Neonatal Med. 2020 Jul 23;1-4. doi: 10.1080/14767058.2020.1786526 pmid: 32698646
325. Poon, L. C., Leung, B. W., Ma, T., Yu, F. N. Y. et al. Relationship between viral load, infection-to-delivery interval and mother-to-child transfer of anti-SARS-CoV-2 antibodies. *Ultrasound in Obstetrics & Gynecology*. 2021; *57*(6), 974–978. https://doi.org/10.1002/uog.23639
326. Popescu, D. E., Cioca, A., Muresan, C., Navolan, D. et al. A Case of COVID-19 Pregnancy Complicated with Hydrops Fetalis and Intrauterine Death. *Medicina*. 2021; *57*(7), 667. https://doi.org/10.3390/medicina57070667
327. Prasad A, Yankappa N, Kumar P, Chaudhary BK, Pati BK, Anant M, Tiwari LK. Excretion of SARS-CoV-2 in breast milk: A single-centre observational study. BMJ Paediatr Open. 2021;5(1):1-5. doi:10.1136/bmjpo-2021-001087. PMID: 34192204
328. Preßler J, Malfertheiner SF, Kabesch M, Buntrock-Döpke H, Häusler S, Ambrosch A, et al. Postnatal SARS-CoV-2 infection and immunological reaction: A prospective family cohort study. Pediatr Allergy Immunol. 2020 Oct;31(7):864-867. doi: 10.1111/pai.13302 pmid: 32515518
329. Pulinx B, Kieffer D, Michiels I, Petermans S, Strybol D, Delvaux S, et al. Vertical transmission of SARS-CoV-2 infection and preterm birth. Eur J Clin Microbiol Infect Dis. 2020 Jul 13:1–5. doi: 10.1007/s10096-020-03964-y pmid: 32661809
330. Gupta P, Kumar S, Sharma SS. SARS-CoV-2 prevalence and maternal-perinatal outcomes among pregnant women admitted for delivery: Experience from COVID-19-dedicated maternity hospital in Jammu, Jammu and Kashmir (India). J Med Virol. 2021;93(9):5505-5514. doi:10.1002/jmv.27074. pmid: 33974301
331. Qadri F, Mariona F. Pregnancy affected by SARS-CoV-2 infection: a flash report from Michigan. J Matern Fetal Neonatal Med. 2020 May 20;1-3. doi: 10.1080/14767058.2020.1765334 pmid: 32434403
332. Quigley, N., Keating, N., Rooney, G., Sheil, O. et al. Management of an unstable preterm covid-19 pregnant woman with emergency caesarean delivery. *Irish Medical Journal*. 2021; *114*(1), 246.
333. Rabah, R. SARS-CoV-2 Placental Infection With Characteristic Histologic Features and Poor Outcome. *Pediatric and Developmental Pathology*. 2021; *24*(3), 280.
334. Radu, M. C., Boeru, C., Marin, M., & Manolescu, L. S. SARS-CoV-2 Infection in Seven Childbearing Women at the Moment of Delivery, a Romanian Experience. *Cureus*. 2021; *13*(1), e12811. https://doi.org/10.7759/cureus.12811
335. Rashan, N., Bahmani, M., & Direkvand-Moghadam, A. A Case Report of COVID-19 in a Pregnant Woman with Clinical Manifestations of Chorioamnionitis in Ilam, Iran. *International Journal of High Risk Behaviors and Addiction*; 2020; *9*(3). doi: 10.5812/ijhrba.105178
336. Rashidian, T., Sharifi, N., Fathnezhad-Kazemi, A., Mirzamrajani, F., Nourollahi, S., & Ghaysouri, A. Death of a neonate with suspected coronavirus disease 2019 born to a mother with coronavirus disease 2019 in Iran: a case report. *Journal of Medical Case Reports*. 2020; *14*(1), 186. doi: 10.1186/s13256-020-02519-1
337. Rathberger K, Häusler S, Wellmann S, Weigl M, Langhammer F, Bazzano MV, Ambrosch A, Fill Malfertheiner S. SARS-CoV-2 in pregnancy and possible transfer of immunity: Assessment of peripartal maternal and neonatal antibody levels and a longitudinal follow-up. J Perinat Med. 2021;49(6):702-708. doi:10.1515/jpm-2021-0166. PMID: 34116588.
338. Rebello CM, Fascina LP, Annicchino G, Pinho JRR, Yoshida R de AM, Zacharias RSB. Vertical transmission of SARS-CoV-2 from infected pregnant mother to the neonate detected by cord blood real-time polymerase chain reaction (RT-PCR). Pediatr Res. 2020 Oct 26. doi: 10.1038/s41390-020-01193-9 pmid: 33106558
339. Rebutini PZ, Zanchettin AC, Stonoga ETS, Prá DMM, de Oliveira ALP, Dezidério F da S, Fonseca AS, Dagostini JCH, Hlatchuk EC, Furuie IN, Longo J da S, Cavalli BM, Dino CLT, Dias VM de CH, Percicote AP, Nogueira MB, Raboni SM, de Carvalho NS, Machado-Souza C, de Noronha L. Association Between COVID-19 Pregnant Women Symptoms Severity and Placental Morphologic Features. Front Immunol. 2021;12(May). doi:10.3389/fimmu.2021.685919. PMID: 34122449.
340. Reddy, A., Engelhardt, K., & Jain, D. Air Leak Syndrome in Two Very Preterm Infants Born to Mothers with Coronavirus Disease 2019: An Association or a Coincidence? *AJP Reports*. 2020; *10*(3), e266–e269. https://doi.org/10.1055/s-0040-1715180
341. Rehana, R., Jamoor, K., & Huda, N. 273: SARS-CoV-2-Induced Emergency Cesarian Section. *Critical Care Medicine*. 2021; *49*(1), 123–123. https://doi.org/10.1097/01.ccm.0000726980.06985.10
342. Remaeus K, Savchenko J, Brismar Wendel S, Brusell Gidlöf S, Graner S, Jones E, et al. Characteristics and short-term obstetric outcomes in a case series of 67 women test-positive for SARS-CoV-2 in

Stockholm, Sweden. Acta Obstet Gynecol Scand. 2020 Dec;99(12):1626-1631. doi: 10.1111/aogs.14006 pmid: 32981033
343. Resta L, Vimercati A, Sablone S, Marzullo A, Cazzato G, Ingravallo G et al. Is the First of the Two Born Saved? A Rare and Dramatic Case of Double Placental Damage from SARS-CoV-2. Viruses 2021, 13, 995. doi: 10.3390/v13060995
344. Resta, L.; Vimercati, A.; Cazzato, G.; Mazzia, G.; Cicinelli, E.;Colagrande, A.; Fanelli, M.; Scarcella,S.V.; Ceci, O.; Rossi, R. SARS-CoV-2and Placenta: New Insights and Perspectives.Viruses. 2021;13, 723. doi:10.3390/v13050723
345. Richtmann R, Torloni MR, Oyamada Otani AR, Levi JE, Crema Tobara M, de Almeida Silva C, et al. Fetal deaths in pregnancies with SARS-CoV-2 infection in Brazil: A case series. Case Rep Womens Health. 2020 Jul 12;27:e00243. doi: 10.1016/j.crwh.2020.e00243. pmid: 32704477
346. Ríos D, García D, Solano E, García A, Valdespino-Vázquez M, Repetto A, et al. Lung ultrasound and neonatal COVID-19 pneumonia: A case report. Research Square. 2020 Sep 16 (Preprint) (Version 2). doi: 10.21203/rs.3.rs-33182/v2
347. Rivera-Hernandez P, Nair J, Islam S, Davidson L, Chang A, Elberson V. Coronavirus Disease 2019 in a Premature Infant: Vertical Transmission and Antibody Response or Lack Thereof. AJP Rep. 2020 Jul;10(3):e224-e227. doi: 10.1055/s-0040-1715176 pmid: 33094009
348. Roberts, J., Cheng, J. D., Moore, E., Ransom, C., Ma, M., & Rogers, B. B. Extensive Perivillous Fibrin and Intervillous Histiocytosis in a SARS-CoV-2 Infected Placenta From an Uninfected Newborn: A Case Report Including Immunohistochemical Profiling. *Pediatric and Developmental Pathology*. 2021; *24*(6), 581–584. https://doi.org/10.1177/10935266211025122
349. Rodrigues ML, Gasparinho G, Sepúlveda F, Matos T. Signs suggestive of congenital SARS-CoV-2 infection with intrauterine fetal death: A case report. Eur J Obstet Gynecol Reprod Biol. 2021 Jan;256:508-509. doi: 10.1016/j.ejogrb.2020.11.042 pmid: 33250220
350. Romagano MP, Guerrero K. Perinatal outcomes in critically ill pregnant women with coronavirus disease 2019. Am J Obstet Gynecol MFM. 2020 Aug; 2(3): 100151. doi: 10.1016/j.ajogmf.2020.100151
351. Rong Q, Abubakar K. A newborn with coronavirus (COVID-19) disease: A brief report. J Neonatal Perinatal Med. 2020;13(4):593-595.doi: 10.3233/NPM-200489 pmid: 32925114
352. Rosen H, Bart Y, Zlatkin R, Ben-Sira L, Ben Bashat D, Amit S et al. Fetal and Perinatal Outcome Following First and Second Trimester COVID-19 Infection: Evidence from a Prospective Cohort Study. J. Clin. Med. 2021, 10, 2152. doi: 10.3390/jcm10102152
353. Sagheb S, Lamsehchi A, Jafary M, Atef-Yekta R, Sadeghi K. Two seriously ill neonates born to mothers with COVID-19 pneumonia- a case report. Ital J Pediatr. 2020; 46,137. doi: https://doi.org/10.1186/s13052-020-00897-2
354. Saha J, Das T, Nayek K. Neonatal Coronavirus 2019 Infection in a tertiary setup of India: A case series. Asian J Med Sci. 2021 Mar 1;12(3):110–3. doi: 10.3126/ajms.v12i3.33617
355. Saha, M. M., Das, S. K., Sarkar, N., Chaudhuri, R., Gaikowad, S., & Shirsath, S. E. SARS-CoV-2/COVID-19 infection in pregnancy and its outcome in a rural tertiary care centre of West Bengal. *Indian Journal of Biochemistry & Biophysics*. 2020; *57*, 694–700.
356. Saha S, Ahmed ANU, Sarkar PK, Bipul MRA, Ghosh K, Rahman SW, et al. The Direct and Indirect Impact of SARS-CoV-2 Infections on Neonates: A Series of 26 Cases in Bangladesh. Pediatr Infect Dis J. 2020 Dec;39(12):e398-e405. doi: 10.1097/INF.0000000000002921 pmid: 33031143
357. Saikia B, Tang J, Robinson S, Nichani S, Lawman KB, Katre M, et al. Neonates with SARS-CoV-2 infection and pulmonary disease safely treated with remdesivir. Pediatr Infect Dis J. 2021 May 1;40(5):e194-e196. doi: 10.1097/INF.0000000000003081 pmid: 33847299
358. Sainathan, S., Heal, M. E., Frantz, E., Johnston, P., Smith, R., & Sharma, M. Use of VA ECMO and percutaneous palliation of ductal dependent coarctation in a neonate with trisomy 21 and COVID-19 pneumonia. *Indian Journal of Thoracic and Cardiovascular Surgery*. 2021; *37*(6), 698–701. https://doi.org/10.1007/s12055-021-01204-2
359. Salik I, Mehta B. Tetralogy of Fallot palliation in a COVID-19 positive neonate. J Clin Anesth. 2020 Nov;66:109914. doi: 10.1016/j.jclinane.2020.109914 pmid: 32474332
360. Lopez Salinas, D., Barba Leon, F., Rodriguez Garcia, L., Jimenez-Baez, M. V., Garma Montiel, F., & Sanchez Castuera, M. E. Vertical Transmission of COVID-19 in Children of Sero-positive Mothers to SARS-CoV-2 in Southeast Mexico: A Case Report. *Respiratory Case Reports*. 2021; *10*(1), 1–7. https://doi.org/10.5505/respircase.2021.05826
361. Salvatore CM, Han JY, Acker KP, Tiwari P, Jin J, Brandler M, et al. Neonatal management and outcomes during the COVID-19 pandemic: an observation cohort study. The Lancet Child and Adolescent Health. 2020 Oct 1;4(10):721–7. doi: https://doi.org/10.1016/ S2352-4642(20)30235-2

362. Sánchez J, Espinosa J, Caballero LC, Campana BS, Quintero A, Luo C, et al. NEW CORONAVIRUS IN PREGNANT WOMEN. Maternal and perinatal outcomes. medRxiv. 2021 Jun 6. doi: 10.1101/2021.06.03.21258328
363. Sánchez-Luna M, Fernández Colomer B, de Alba Romero C, Alarcón Allen A, Baña Souto A, Camba Longueira F, Cernada Badía M, Galve Pradell Z, González López M, López Herrera MC, Ribes Bautista C, Sánchez García L, Zamora Flores E; SENEO COVID-19 Registry Study Group. Neonates Born to Mothers With COVID-19: Data From the Spanish Society of Neonatology Registry. Pediatrics. 2021 Feb;147(2):e2020015065. doi: 10.1542/peds.2020-015065. PMID: 33479162.
364. Santhosh J, al Salmani M, Khamis F, Ali Al Ubaidani S, Al-Zakwani I. Clinical characteristics of COVID-19 in pregnant women: A retrospective descriptive single-center study from a tertiary hospital in Muscat, Oman. Int J Gynaecol Obstet. 2021 Feb;152(2):270-274. doi: 10.1002/ijgo.13427 pmid: 33075146
365. Martínez Santos, L., Olabarrieta Zarain, U., García Trancho, A., Serna de la Rosa, R. M., Vallinas Hidalgo, I., Maroño Boedo, M. J., & Martínez Ruiz, A. Planificación y manejo anestésico para cesárea en gestante con neumonía por SARS-CoV-2. *Revista Española de Anestesiología y Reanimación*. 2021; *68*(1), 46–49. https://doi.org/10.1016/j.redar.2020.08.006
366. Rosado Santos, R., Martins, I., & Ayres-de-Campos, D. Prevalence of SARS-CoV-2 infection in asymtomatic pregnant women and their partners in a tertiary care hospital in Portugal. *The Journal of Maternal-Fetal & Neonatal Medicine*. 2020; 1–2. https://doi.org/10.1080/14767058.2020.1793323
367. Sastry SR, Pryor R, Raybould JE, Reznicek J, Cooper K, Patrick A, et al. Universal screening for the SARS-CoV-2 virus on hospital admission in an area with low COVID-19 prevalence. Infect Control Hosp Epidemiol. 2020 Oct;41(10):1231-1233. doi: 10.1017/ice.2020.358 pmid: 32698924
368. Sattari M, Bashirian S, Masoumi SZ, Shayan A, Jenabi E, Ghelichkhani S, et al. Evaluating clinical course and risk factors of infection and demographic characteristics of pregnant women with COVID-19 in Hamadan Province, West of Iran. J Res Health Sci. 2020 Aug 17;20(3):e00488. doi: 10.34172/jrhs.2020.22 pmid: 33169720
369. Savasi VM, Parisi F, Patanè L, Ferrazzi E, Frigerio L, Pellegrino A, Spinillo A, Tateo S, Ottoboni M, Veronese P, Petraglia F, Vergani P, Facchinetti F, Spazzini D, Cetin I. Clinical Findings and Disease Severity in Hospitalized Pregnant Women With Coronavirus Disease 2019 (COVID-19). Obstet Gynecol. 2020 Aug;136(2):252-258. doi: 10.1097/AOG.0000000000003979. pmid: 32433453.
370. Savić, D., Simović, A., Ristić, D., Stojković, T., Živojinović, S., Prodanović, T., Pavlović, S., Stojković, A., Igrutinović, Z., & Pavlović, R. Fatal Outcome of COVID-19 in a Newborn. *Indian Journal of Pediatrics*. 2021; *88*(9), 949–949. doi: 10.1007/s12098-021-03860-z
371. Savirón-Cornudella, R., Villalba, A., Zapardiel, J., Andeyro-Garcia, M., Esteban, L. M., & Pérez-López, F. R. Severe acute respiratory syndrome coronavirus 2 (SARS-CoV-2) universal screening in gravids during labor and delivery. *European Journal of Obstetrics & Gynecology and Reproductive Biology*. 2021; *256*, 400–404. https://doi.org/10.1016/j.ejogrb.2020.11.069
372. Sayeed SK, Rahman M, Humayon Kabir A, Moniruzzaman Md, Mahmud R, Yusuf M, et al. Clinical, Laboratory Characteristics and Pregnancy Outcome of COVID-19 Patients Admitted in the Largest COVID Dedicated Hospital of Bangladesh. American Journal of Internal Medicine. 2021;9(1):11-16. doi: 10.11648/j.ajim.20210901.12
373. Schnettler, W. T., al Ahwel, Y., & Suhag, A. Severe acute respiratory distress syndrome in coronavirus disease 2019–infected pregnancy: obstetric and intensive care considerations. *American Journal of Obstetrics & Gynecology MFM*. 2020; *2*(3), 100120. https://doi.org/10.1016/j.ajogmf.2020.100120
374. Schoenmakers S, Snijder P, Verdijk RM, Kuiken T, Kamphuis SSM, Koopman LP, et al. Severe acute respiratory syndrome coronavirus 2 placental infection and inflammation leading to fetal distress and neonatal multi-organ failure in an asymptomatic woman. J Pediatric Infect Dis Soc. 2021 May 28;10(5):556-561. doi: 10.1093/jpids/piaa153 pmid: 33367801
375. Schwartz DA, Mohagheghi P, Beigi B, Zafaranloo N, Moshfegh F, Yazdani A. Spectrum of neonatal COVID-19 in Iran: 19 infants with SARS-CoV-2 perinatal infections with varying test results, clinical findings and outcomes. J Matern Fetal Neonatal Med. 2020 Aug 12;1-10. doi: 10.1080/14767058.2020.1797672 pmid: 32783494
376. Schwartz, D. A. An Analysis of 38 Pregnant Women With COVID-19, Their Newborn Infants, and Maternal-Fetal Transmission of SARS-CoV-2: Maternal Coronavirus Infections and Pregnancy Outcomes. *Archives of Pathology & Laboratory Medicine*. 2020; *144*(7), 799–805. doi: 10.5858/arpa.2020-0901-SA
377. Sehra, R. N., Goyal, A. K., Saini, R., Verma, S., Gothwal, S., Gupta, R. K., & Gupta, M. L. Clinicolaboratory Profile of and Outcomes in Neonates Born to COVID-19–Positive Mothers. *Perinatology*. 2021; *21*(4).

378. Semeshkin AA, Vechorko VI, Silaev BV, Levchuk NN, Polikarpova SV, Akerkov OV. IgM and IgG antibodies against SARS-CoV-2 in neonates born to mothers with COVID-19. Bull. Russ. State Med. Univ. 2020 Jun 26; 3: 28-30. doi:10.24075/brsmu.2020.036
379. Servei Català de la Salut, Àrea de Sistemes d'Informació. Informe Seguiment Gestants. Barcelona; Servei Català de la Salut; 2020 May 18. Available at: https://scientiasalut.gencat.cat/handle/11351/4940
380. Shah PT, Shah SR, Shah SR, Yadava PA, Patel BS, Chudasama TJ. Fetomaternal outcome in COVID-19 infected pregnant women: a preliminary clinical study. IJRCOG. 2020 Sep;9(9):3704-10. doi: http://dx.doi.org/10.18203/2320-1770.ijrcog20203843
381. Shaiba, L. A., Hadid, A., Altirkawi, K. A., Bakheet, H. M. et al. Case Report: Neonatal Multi-System Inflammatory Syndrome Associated With SARS-CoV-2 Exposure in Two Cases From Saudi Arabia. *Frontiers in Pediatrics*. 2021; *9*. https://doi.org/10.3389/fped.2021.652857
382. Shanes, E. D., Mithal, L. B., Otero, S., Azad, H. A., Miller, E. S., & Goldstein, J. A. Placental Pathology in COVID-19. *American Journal of Clinical Pathology*. 2020; *154*(1), 23–32. doi: 10.1093/ajcp/aqaa089
383. Sharma, K. A., Kumari, R., Kachhawa, G., Chhabra, A., Agarwal, R., Sharma, A., Kumar, S., & Bhatla, N. Management of the first patient with confirmed COVID-19 in pregnancy in India: From guidelines to frontlines. *International Journal of Gynecology & Obstetrics*. 2020; *150*(1), 116–118. https://doi.org/10.1002/ijgo.13179
384. Sharma N, Seehra N, Kabra S. Pregnancy with Covid-19 Infection and Fetomaternal Outcomes. Journal of Evolution of Medical and Dental Sciences. 2021 Jan 4;10(1):23–7. doi: 10.14260/jemds/2021/5
385. Sharma R, Seth S, Sharma R, Yadav S, Mishra P, Mukhopadhyay S. Perinatal outcome and possible vertical transmission of coronavirus disease 2019: experience from North India. Clin Exp Pediatr. 2021 May;64(5):239-246. doi: 10.3345/cep.2020.01704 pmid: 33592686
386. Sheikhahmadi, S., Behzadi, S., karimi, A., & Zakaryaei, F. (2021). Evaluating Vertical Transmission of COVID-19 from Mothers to Neonates: An Iranian Case Series of 8 Patients. *Archives of Iranian Medicine*. 2021; *24*(5), 405–408. https://doi.org/10.34172/aim.2021.58
387. Shende P, Gaikwad P, Gandhewar M, Ukey P, Bhide A, Patel V, et al. Persistence of SARS-CoV-2 in the first trimester placenta leading to vertical transmission and fetal demise from an asymptomatic mother. Hum Reprod. 2021 Mar 18;36(4):899-906. doi: 10.1093/humrep/deaa367 pmid: 33346816
388. Shmakov RG, Prikhodko A, Polushkina E, Shmakova E, Pyregov A, Bychenko V, et al. Clinical course of novel COVID-19 infection in pregnant women. J Matern Fetal Neonatal Med. 2020 Nov 29;1-7. doi:10.1080/14767058.2020.1850683 pmid: 33249969
389. Shook LL, Collier AY Goldfarb IT, Diouf K, Akinwunmi BO, Young N et al. Vertical transmission of SARS-CoV-2: consider the denominator. Amer Jour Obs & Gyne. 2021;3:4: 100386. doi: 10.1016/j. ajogmf.2021.100386
390. Sibia P, Kaur N, Sibia RS, Kaur S, Kaur A. Impact of COVID-19 on Maternal and Neonatal Outcomes in Pregnancy: A Prospective Study. J Clin Diagnostic Res. Published online 2021:1-4. doi:10.7860/jcdr/2021/49342.14965.
391. Sileo, F. G., Tramontano, A. L., Leone, C., Meacci, M., et al. Pregnant woman infected by Coronavirus disease (COVID-19) and calcifications of the fetal bowel and gallbladder. *Minerva Obstetrics and Gynecology*. 2021; *73*(1), 121–124. https://doi.org/10.23736/S2724-606X.20.04717-6
392. Sinaci S, Ocal DF, Seven B, Anuk AT, Besimoglu B, Keven MC et al. Vertical transmission of SARS-CoV-2: A prospective cross- sectional study from a tertiary center. J Med Virol. 2021;1-9. doi: 10.1002/jmv.27128.
393. Sinelli M, Paterlini G, Citterio M, di Marco A, Fedeli T, Ventura ML. Early neonatal SARS-CoV-2 infection manifesting with hypoxemia requiring respiratory support. Pediatrics. 2020 Jul;146(1):e20201121. doi: 10.1542/peds.2020-1121 pmid: 32366612
394. Singh MV, Shiravastava A, Maurya M, Tripathi A, Sachan R, Siddiqui SA. Vertical Transmission of SARS-CoV-2 from an Asymptomatic Pregnant Woman in India. J Trop Pediatr. 2020 Sep 25;fmaa048. doi: 10.1093/tropej/fmaa048 pmid: 32974677
395. Singh, P., Acharya, N., & Verma, N. Experience of managing covid-19 suspected case in labour in a green zone district tertiary care hospital. *International Journal of Research in Pharmaceutical Sciences*. 2020; *11*(SPL1), 500–505. https://doi.org/10.26452/ijrps.v11iSPL1.2838
396. Singh V, Choudhary A, Datta MR, Ray A. Maternal and Neonatal Outcomes of COVID-19 in Pregnancy: A Single-Centre Observational Study. Cureus. 2021 Feb 6;13(2):e13184. doi: 10.7759/cureus.13184 pmid: 33717728
397. Sivevski, A., Karadzova, D., Davceva, N., Aleksioska-Papestiev, I., Kadriu, R., Velickovic, I., Markovic, I., Pejcic, N., & Baysinger, C. L. Post Partum Death in a Patient Diagnosed With COVID-19. *Frontiers in Global Women's Health*. 2020; *1*. https://doi.org/10.3389/fgwh.2020.567810

398. Sola A, Rodríguez S, Cardetti M, Dávila C. COVID-19 perinatal en América Latina. Rev Panam Salud Publica. 2020 Jul 31;44:e47. doi: 10.26633/RPSP.2020.47 pmid: 32754205
399. Solís-García G, Gutiérrez-Vélez A, Pescador Chamorro I, Zamora-Flores E, Vigil-Vázquez S, Rodríguez-Corrales E, et al. Epidemiology, management and risk of SARS-CoV-2 transmission in a cohort of newborns born to mothers diagnosed with COVID-19 infection. An Pediatr (Engl Ed). 2021 Mar;94(3):173-178. doi: 10.1016/j.anpede.2020.12.006 pmid: 33521167
400. Song D, Prahl M, Gaw SL, Narasimhan S, Rai D, Huang A et al. Passive and active immunity in infants born to mothers with SARS-CoV-2 infection during pregnancy: Prospective cohort study. medRxiv 2021.05.01.2125587. doi: 10.1101/2021.05.01.21255871.
401. Song L, Xiao W, Ling K, Yao S, Chen X. Anesthetic Management for Emergent Cesarean Delivery in a Parturient with Recent Diagnosis of Coronavirus Disease 2019 (COVID-19): A Case Report. Transl Perioper & Pain Med 2020; 7(3):234-237. doi: 10.31480/2330-4871/118
402. Sri Sri G, Aravind R, Isukapalli V, Begum S. Feto-maternal outcome in Covid infected pregnancies over 3 month period from semi-urban Medical Colleges-A prospective study. International Archives of Integrated Medicine. 2020 ; 7(10):158-161
403. Steffen HA, Swartz SR, Jackson JB, Kenne KA, ten Eyck PP, Merryman AS, et al. SARS-CoV-2 Infection during Pregnancy in a Rural Midwest All-delivery Cohort and Associated Maternal and Neonatal Outcomes. Am J Perinatol. 2021 May;38(6):614-621. doi: 10.1055/s-0041-1723938 pmid: 33611783
404. Stonoga E, Lanzoni L, Rebutini P, de Oliveira A, Chiste J, Fugaça C, et al. Intrauterine Transmission of SARS-CoV-2. Emerg Infect Dis. 2021 Feb;27(2):638-641. doi: 10.3201/eid2702.203824 pmid: 33185524
405. Sukhikh G, Petrova U, Prikhodko A, Starodubtseva N, Chingin K, Chen H, et al. Vertical transmission of SARS-CoV-2 in second trimester associated with severe neonatal pathology. Viruses. 2021 Mar; 13(3): 447. doi: 10.3390/v13030447 pmid: 33801923
406. Sun M, Xu G, Yang Y, Tao Y, Pian-Smith M, Madhavan V, et al. Evidence of mother-to-newborn infection with COVID-19. Br J Anaesth. 2020 Aug; 125(2): e245–e247. doi: 10.1016/j.bja.2020.04.066 pmid: 32386820
407. Suresh, S. C., MacGregor, C. A., & Ouyang, D. W. Urgent Cesarean Delivery Following Nonstress Test in a Patient with COVID-19 and Pregestational Diabetes. *NeoReviews*. 2020 *21*(9), e625–e630. https://doi.org/10.1542/neo.21-9-e625
408. Suyuthi FP, Chalid MT, Padjalangi AN, Djaharuddin I, Massi MN. Maternal and Perinatal Outcome on Pregnancy with Covid-19 Infection at Dr. Wahidin Sudirohusodo Hospital Makassar During the Period of April-July 2020. Indian Journal of Forensic Medicine & Toxicology. 2020 Oct 29; 14(4):7519-22 doi: https://doi.org/10.37506/ijfmt.v14i4.12836
409. Rafiei Tabatabaei S, Fallahi M, Boskabadi A, Taslimi Taleghani N, Pajouhandeh F, Tabatabaee S, et al. COVID-19 in Neonates, A Case Series Study From Tertiary Neonatal Centers in Iran. Archives of Pediatric Infectious Diseases. 2021 Jan 23; 9 (4); e110603. doi: 10.5812/pedinfect.110603
410. Tadas MP, Prashanthi S, Waikar M. Maternal and neonatal outcomes of pregnant women with covid-19: A case–control study at a tertiary care center in India. JSAFOG. 2021;13(1):44-49. doi:10.5005/jp-journals-10006-1850a.
411. Taghizadieh, A., Mikaeili, H., Ahmadi, M., & Valizadeh, H. Acute kidney injury in pregnant women following SARS-CoV-2 infection: A case report from Iran. *Respiratory Medicine Case Reports*. 2020; *30*, 101090. https://doi.org/10.1016/j.rmcr.2020.101090
412. Tallarek AC, Urbschat C, Fonseca Brito L, Stanelle-Bertram S, Krasemann S, Frascaroli G, Thiele K, Wieczorek A, Felber N, Lütgehetmann M, Markert UR, Hecher K, Brune W, Stahl F, Gabriel G, Diemert A, Arck PC. Inefficient Placental Virus Replication and Absence of Neonatal Cell-Specific Immunity Upon Sars-CoV-2 Infection During Pregnancy. Front Immunol. 2021;12(June):1-13. doi:10.3389/fimmu.2021.698578. PMID: 34149740
413. Tang F, Luo W, Wang X, Chen Z, Li H, Liu W, et al. An Observational Study of Intrauterine Vertical Transmission of SARS-CoV-2 in 20 Neonates. Preprints with The Lancet. 2020 Jul 16 (Preprint). doi: http://dx.doi.org/10.2139/ssrn.3618210
414. Tang, J., Song, W., Xu, H., & Wang, N. No evidence for vertical transmission of SARS-CoV-2 in two neonates with mothers infected in the second trimester. *Infectious Diseases*. 2020; *52*(12), 913–916. https://doi.org/10.1080/23744235.2020.1798499
415. Tasca, C., Rossi, R. S., Corti, S., Anelli, G. M., Savasi, V., Brunetti, F. et al. Placental pathology in COVID-19 affected pregnant women: A prospective case-control study. *Placenta*. 2021; *110*, 9–15. https://doi.org/10.1016/j.placenta.2021.04.002

416. Teixeira M de LB, da Costa Ferreira Júnior O, João E, Fuller T, Silva Esteves J, Mendes-Silva W, Carvalho Mocarzel C, Araújo Maia R, Theodoro Boullosa L, Gonçalves CCA, Frankel PP, Gouvêa MIF da S. Maternal and neonatal outcomes of sars-cov-2 infection in a cohort of pregnant women with comorbid disorders. Viruses. 2021;13(7):1-10. doi:10.3390/v13071277. PMID: 34208954.

417. Thanigainathan S, Kaliyaperumal V, Sivanandan S, Rengaraj S, Dhodapkar R, Bethou A. Is SARS-CoV-2 Transmitted Through Breastfeeding? Indian J Pediatr. 2021 Feb 8;1-2. doi: 10.1007/s12098-021-03681-0 pmid: 33555566
418. Thapa, B., Acharya, S., & Karki, S. Vertical Transmission of COVID-19: A Case Report and Review of Literature. *Journal of Nepal Health Research Council*. 2021; *19*(1), 203–205. doi: 10.33314/jnhrc.v19i1.3312
419. Tong, C., Wang, L., Liu, Y., Tan, J., Li, Q., & Chen, Y. Premature infant born to a convalescent mother with COVID-19 in mid-term pregnancy . *Chinese Journal of Perinatal Medicine*. 2020; *23*(5), 321–323.
420. Toto, V., Tosi, D., de Vitis, L. A., Marconi, A. M., & Bulfamante, G. Finding of Severe Acute Respiratory Syndrome Coronavirus 2 (SARS-CoV-2) Within Placental Tissue 11 Weeks After Maternal Infection. *Archives of Pathology & Laboratory Medicine*. 2021; *145*(8), 920–921. doi: 10.5858/arpa.2021-0076-LE
421. Tran, H. T., Huynh, L. T., Le, C. H. M., et al Early Essential Newborn Care can still be used with mothers who have COVID-19 if effective infection control measures are applied. *Acta Paediatrica*. 2021; *110*(7), 1991–1994. https://doi.org/10.1111/apa.15837
422. Trieu C, Poole C, Cron RQ, Hallman M, Rutledge C, Bliton K, et al. Severe neonatal coronavirus disease 2019 presenting as acute respiratory distress syndrome. Pediatr Infect Dis J. 2020 Nov;39(11):e367-e369. doi: 10.1097/INF.0000000000002864 pmid: 33021595
423. Trombetta, A., Comar, M., Tommasini, A., Canton, M., Campisciano, G., Zanotta, N., Cason, C., Maso, G., & Risso, F. M. SARS-CoV-2 Infection and Inflammatory Response in a Twin Pregnancy. *International Journal of Environmental Research and Public Health*. 2021; *18*(6), 3075. https://doi.org/10.3390/ijerph18063075
424. Tsatsaris, V., Mariaggi, A.-A., Launay, O., et al. SARS-COV-2 IgG antibody response in pregnant women at delivery. *Journal of Gynecology Obstetrics and Human Reproduction*. 2021; *50*(7), 102041. https://doi.org/10.1016/j.jogoh.2020.102041
425. Tutiya, C. T., Siaulys, M. M., Kondo, M. M., et al. Possible formation of pulmonary microthrombi in the early puerperium of pregnant women critically ill with COVID-19: Two case reports. *Case Reports in Women's Health*. 2020; *27*, e00237. https://doi.org/10.1016/j.crwh.2020.e00237
426. Vaezi M, Mirghafourvand M, Hemmatzadeh S. Characteristics, clinical and laboratory data and outcomes of pregnant women with confirmed SARS-CoV-2 infection admitted to Al-Zahra tertiary referral maternity center in Iran: a case series of 24 patients. BMC Pregnancy Childbirth. 2021;21(1):1-8. doi:10.1186/s12884-021-03764-y. PMID: 34001013

427. Valdespino-Vázquez M, Helguera-Repetto C, León-Juárez M, Villavicencio-Carrisoza O, Flores-Pliego A, Moreno-Verduzco E, et al. Fetal and placental infection with SARS-CoV-2 in early pregnancy. J Med Virol. 2021 Jul;93(7):4480-4487. doi: 10.1002/jmv.26965 pmid: 33764543
428. Valk JE, Chong AM, Uhlemann AC, Debelenko L. Detection of SARS-CoV-2 in placental but not fetal tissues in the second trimester. J Perinatol. 2021 May;41(5):1184-1186. doi: 10.1038/s41372-020-00877-8 pmid: 33257773
429. Vallejo, V., & Ilagan, J. G. A Postpartum Death Due to Coronavirus Disease 2019 (COVID-19) in the United States. *Obstetrics & Gynecology*. 2020; *136*(1), 52–55. https://doi.org/10.1097/AOG.0000000000003950
430. Vashukova MA, Zinserling VA, Semenova NY, Lugovskaya NA, Narkevich TA, Sukhanova Y v. Возможна ли перинатальная COVID-19: первые результаты. Jurnal Infektologii. 2020 Jan 1;12(3):51–5. doi: 10.22625/2072-6732-2020-12-3-51-55
431. Vera Loyola EM, Montenegro Cruz I, Cruzate Cabrejos V, Marcelo Pacheco H, Arce Benitez M, Pelaez Chomba M. Gestación en tiempos de pandemia COVID-19. Hospital Nacional Docente Madre Niño San Bartolomé, Lima, Perú. Revista Peruana de Ginecología y Obstetricia. 2020 Nov 6;66(3). doi:http://dx.doi.org/10.31403/rpgo.v66i2265
432. Verma, S., Joshi, C. S., Silverstein, R. B., He, M., Carter, E. B., & Mysorekar, I. U. SARS-CoV-2 colonization of maternal and fetal cells of the human placenta promotes alteration of local renin-angiotensin system. *Med*. 2021; *2*(5), 575-590.e5. https://doi.org/10.1016/j.medj.2021.04.009

433. Vivanti A, Vauloup-Fellous C, Prevot S, Zupan V, Suffee C, Cao J do, et al. Transplacental transmission of SARS-CoV-2 infection. Research Square. 2020 Jul 14 (Preprint). doi: 10.21203/rs.3.rs-28884/v1
434. Vizheh, M., Muhidin, S., Aghajani, F., Maleki, Z., Bagheri, F., Hosamirudsari, H., Aleyasin, A., & Tehranian, A. Characteristics and outcomes of COVID-19 pneumonia in pregnancy compared with infected nonpregnant women. *International Journal of Gynecology & Obstetrics*. 2021; *153*(3), 462–468. https://doi.org/10.1002/ijgo.13697
435. Von Kohorn I, Stein S, Shikani B, Ramos-Benitez MJ, Vannella KM, Hewitt SM, et al. In Utero Severe Acute Respiratory Syndrome Coronavirus 2 Infection. J Pediatric Infect Dis Soc. 2020 Dec 31;9(6):769-771. doi: 10.1093/jpids/piaa127 pmid: 33089311
436. Vousden N, Bunch K, Morris E, Simpson N, Gale C, O'Brien P, et al. The incidence, characteristics and outcomes of pregnant women hospitalized with symptomatic and asymptomatic SARS-CoV-2 infection in the UK from March to September 2020: A national cohort study using the UK Obstetric Surveillance System (UKOSS). PLoS One. 2021 May 5;16(5):e0251123. doi: 10.1371/journal.pone.0251123 pmid: 33951100
437. Walczak, A., Wilks, K., Shakhovskoy, R., Baird, T., Schlebusch, S., Taylor, C., Reid, D., & Choong, K. COVID-19 in a complex obstetric patient with cystic fibrosis. *Infection, Disease & Health*. 2020; *25*(4), 239–241. https://doi.org/10.1016/j.idh.2020.07.002
438. Wang J, Wang D, Chen G, Tao X, Zeng L. SARS-CoV-2 infection with gastrointestinal symptoms as the first manifestation in a neonate. Zhongguo Dang Dai Er Ke Za Zhi. 2020 Mar;22(3):211-214. doi: 10.7499/j.issn.1008-8830.2020.03.006 pmid: 32204755
439. Wang S, Guo L, Chen L, Liu W, Cao Y, Zhang J, et al. A case report of neonatal 2019 coronavirus disease in China. Clin Infect Dis. 2020 Jul 28;71(15):853-857. doi: 10.1093/cid/ciaa225 pmid: 32161941
440. Wang, X., Zhou, Z., Zhang, J., Zhu, F., Tang, Y., & Shen, X. A Case of 2019 Novel Coronavirus in a Pregnant Woman With Preterm Delivery. *Clinical Infectious Diseases*. 2020; *71*(15), 844–846. https://doi.org/10.1093/cid/ciaa200
441. Wardell H, Campbell JI, Vanderpluym C, Dixit A, Mbi MM. SARS-CoV-2 Infection in Febrile Neonates. Pediatr Infect Dis J. 2020 Jul 9; 9(5):630-5. doi: https://doi.org/10.1093/jpids/piaa084 pmid: 32645175
442. Wiyati PS, Adawiyah R, Cahyanti RD, Pramono BA, Hadijono RS, Trisanto AT, Kurube IM. High fatality rate maternal with COVID-19: lesson learned tertiary hospital in Indonesia. Top Scoring Abstracts of the RCOG Virtual World Congress 2021. BJOG. 2021 Jun;128 Suppl 2:4-281. doi: 10.1111/1471-0528.16715. PMID: 34096154.
443. Lebrão, C. W., Cruz, M. N., Silva, M. H. da, Dutra, L. V., Cristiani, C., Affonso Fonseca, F. L., & Suano-Souza, F. I. Early Identification of IgA Anti-SARSCoV-2 in Milk of Mother With COVID-19 Infection. *Journal of Human Lactation*. 2020; *36*(4), 609–613. https://doi.org/10.1177/0890334420960433
444. Wu H, Liao S, Wang Y, Guo M, Lin X, Wu J, Wang R, Lv D, Wu D, He M, Hu B, Long R, Peng J, Yang H, Yin H, Wang X, Huang Z, Lan K, Zhou Y, Zhang W, Xiao Z, Zhao Y, Deng D, Wang H. Molecular evidence suggesting the persistence of residual SARS-CoV-2 and immune responses in the placentas of pregnant patients recovered from COVID-19. Cell Prolif. 2021;54(9):1-13. doi:10.1111/cpr.13091. PMID: 34291856
445. Wu YT, Liu J, Xu JJ, Chen YF, Yang W, Chen Y, et al. Neonatal outcome in 29 pregnant women with COVID-19: A retrospective study in Wuhan, China. PLoS Med. 2020 Jul; 17(7): e1003195. doi: 10.1371/journal.pmed.1003195 pmid: 32722722
446. Xia, H., Zhao, S., Wu, Z., Luo, H., Zhou, C., & Chen, X. Emergency Caesarean delivery in a patient with confirmed COVID-19 under spinal anaesthesia. *British Journal of Anaesthesia*. 2020; *124*(5), e216–e218. doi: 10.1016/j.bja.2020.02.016
447. Xiong, X., Wei, H., Zhang, Z., Chang, J., Ma, X., Gao, X., Chen, Q., & Pang, Q. Vaginal delivery report of a healthy neonate born to a convalescent mother with COVID-19. *Journal of Medical Virology*. 2020; *92*(9), 1657–1659. https://doi.org/10.1002/jmv.25857
448. Xu S, Shao F, Bao B, Ma X, Xu Z, You J, et al. Clinical Manifestation and Neonatal Outcomes of Pregnant Patients With Coronavirus Disease 2019 Pneumonia in Wuhan, China. Open Forum Infect Dis. 2020 Jul 5;7(7):ofaa283. doi: 10.1093/ofid/ofaa283 pmid: 32743014
449. Yadav V, Goel N, Afreen N, Chutani N, Agarwal S. COVID 19 in pregnancy; obstetrical and neonatal outcomes: A retrospective comparative study. IJOGR. 2020 Dec 28;7(4):584–9. doi: https://doi.org/10.18231/j.ijogr.2020.124

450. Yaman A, Kandemir I, Varkal MA. Infants infected with SARS-CoV-2 and newborns born to mother diagnosed with COVID-19: clinical experience. Irish Journal of Medical Science. doi: 10.1007/s11845-021-02662-8.
451. Yang H, Hu B, Zhan S, Yang LY, Xiong G. Effects of Severe Acute Respiratory Syndrome Coronavirus 2 Infection on Pregnant Women and Their Infants. Arch Pathol Lab Med. 2020 Oct 1;144(10):1217-1222. doi: 10.5858/arpa.2020-0232-SA pmid: 32422078
452. Yang H, Sun G, Tang F, Peng M, Gao Y, Peng J, et al. Clinical features and outcomes of pregnant women suspected of coronavirus disease 2019. J Infect. 2020 Jul;81(1):e40-e44. doi: 10.1016/j.jinf.2020.04.003 pmid: 32294503
453. Yaqoub, S., Ahmad, S., Mansouri, Z. et al. Management of life-threatening acute respiratory syndrome and severe pneumonia secondary to COVID-19 in pregnancy: A case report and literature review. *Clinical Case Reports*. 2021; *9*(1), 137–143. https://doi.org/10.1002/ccr3.3485
454. Yildiz, H., Yarci, E., Bozdemir, S. E., Ozdinc Kizilay, N., et al. COVID-19-Associated Cerebral White Matter Injury in a Newborn Infant With Afebrile Seizure. *Pediatric Infectious Disease Journal*. 2021;*40*(7), e268–e269. doi:10.1097/INF.0000000000003143
455. Yilmaz, R., Kiliç, F., Arican, Ş., Hacibeyoğlu, G., Süslü, H., Koyuncu, M., & Tuncer Uzun, S. Anesthetic management for cesarean birth in pregnancy with the novel coronavirus (COVID-19). *Journal of Clinical Anesthesia*. 2020; *66*, 109921. doi:10.1016/j.jclinane.2020.109921
456. Yu, N., Li, W., Kang, Q., Zeng, W., Feng, L., & Wu, J. No SARS-CoV-2 detected in amniotic fluid in mid-pregnancy. *The Lancet Infectious Diseases*. 2020; *20*(12), 1364. https://doi.org/10.1016/S1473-3099(20)30320-0
457. Yu X, Chen H, Luo F, Guo J, Qiao Y, Zhang W, et al. Further Evaluation of the Mother-to-Child Transmission Potential of SARS-CoV-2 Infection During Pregnancy: A Retrospective Study. Research Square. 2020 Oct 6 (Preprint). doi:10.21203/rs.3.rs-77490/v1
458. Yu, Z.-Y., Xue, W., Feng, Y., Bai, Q., et al. Transport, diagnosis and treatment of a newborn with severe SARS-CoV-2 infection: A case report. *Chinese Journal of Evidence-Based Pediatrics*. 2020; *15*(1), 37–41.
459. Zaharie G, Muresan D, Matyas M. Diagnosis Challenges, Management, and Outcome of Infants Born to Mothers With COVID 19. Research Square. 2020 Sep 8 (Preprint). doi:https://doi.org/10.21203/rs.3.rs-65377/v1
460. Zaigham M, Holmberg A, Karlberg ML, Lindsjö OK, Jokubkiene L, Sandblom J, et al. Intrauterine vertical SARS-CoV-2 infection: a case confirming transplacental transmission followed by divergence of the viral genome. BJOG. 2021 Jul;128(8):1388-1394. doi: 10.1111/1471-0528.16682 pmid: 33638908
461. Zeng L, Tao X, Yuan W, Wang J, Liu X, Liu Z. First case of neonate infected with novel coronavirus pneumonia in China. Zhonghua Er Ke Za Zhi. 2020 Feb 17;58(0):E009. doi: 10.3760/cma.j.issn.0578-1310.2020.0009 pmid: 10.3760/cma.j.issn.0578-1310.2020.0009
462. Zeng L, Xia S, Yuan Q, Yan K, Xiao F, Shao J, et al. Neonatal Early-Onset Infection With SARS-CoV-2 in 33 Neonates Born to Mothers With COVID-19 in Wuhan, China. JAMA Pediatr. 2020 Jul 1;174(7):722-725. doi: 10.1001/jamapediatrics.2020.0878 pmid: 32215598
463. Zgutka K, Prasanth K, Pinero-Bernardo S, Lew LQ, Cervellione K, Rhythm R et al. Infant outcomes and maternal COVID-19 status at delivery. J. Perinat. Med. 2021; 3:15. doi: 10.1515/jpm-2020-0481.
464. Zhang P, Salafia C, Heyman T, Salafia C, Lederman S, Dygulska B. Detection of severe acute respiratory syndrome coronavirus 2 in placentas with pathology and vertical transmission. Am J Obstet Gynecol MFM. 2020 Nov; 2(4): 100197. doi: 10.1016/j.ajogmf.2020.100197 pmid: 32838273
465. Zhao, Y.W, et al. COVID-19 infection in pregnant woman giving birth to a newborn with dyspnea: a case report. Chin J Neonatal (Zhonghua Xin Sheng Er Ke Za Zhi). 2020; 35(2):84-85
466. Zheng, T., Guo, J., He, W., Wang, H., Yu, H., & Ye, H. Coronavirus disease 2019 (COVID-19) in pregnancy: 2 case reports on maternal and neonatal outcomes in Yichang city, Hubei Province, China. *Medicine*. 2020; *99*(29), e21334. https://doi.org/10.1097/MD.0000000000021334
467. Zhou, R., Chen, Y., Lin, C., Li, H., Cai, X., Cai, Z., & Lin, G. Asymptomatic COVID-19 in pregnant woman with typical chest CT manifestation: a case report. *Chinese Journal of Perinatal Medicine*. 2020; *23*(3).
468. Zhu, C., Liu, W., Su, H., Li, S., et al. Breastfeeding Risk from Detectable Severe Acute Respiratory Syndrome Coronavirus 2 in Breastmilk. *Journal of Infection*. 2020; *81*(3), 452–482. https://doi.org/10.1016/j.jinf.2020.06.001
469. Zhuang, S., Guo, L., Cao, Y., Chen, H., Xu, D., Li, J., & Zhang, Y. Perinatal COVID-19: a case report. *Chinese Journal of Perinatal Medicine*. 2020; *23*(2), 85–90.

470. Zhumabekova, A., Tagaev, T., Yethindra, V., Zhumabaeva, S., Ysabaeva, D., & Imankulova, B. A case report of a pregnant woman with coronavirus disease 2019 (COVID-19) and her live-born infant. *Biomedicine*. 2021; *40*(4), 551–553. https://doi.org/10.51248/.v40i4.341
471. Zlochiver V, Tilkens B, Perez Moreno AC, Aziz F, Jan MF. COVID-19 deliveries: maternal features and neonatal outcomes. J Patient Cent Res Rev. 2021;8:286-9. doi: 10.17294/2330-0698.1848.
472. Gobierno de México. Informes epidemiológicos de embarazadas y puérperas estudiadas, ante sospecha de COVID 19. 2020 Jul 6. Available at: https://www.gob.mx/salud/documentos/informes-epidemiologicos-de-embarazadas-y-puerperas-estudiadas-ante-sospecha-de-covid-19

Appendix 5: Quality assessment for risk of bias in non-comparative cohort studies using the tool by Hoy et al

	External Validity				Internal validity						Summary
Study	Representativeness	Sampling frame	Selection	Non-response	Data collection	Case definition	Measurement	Differential verification	Adequate follow up	Appropriate numerator and denominator	Summary
2020 July Informe Epidemiológico Embarazadas y Puerperas sem28, 2020	LOW	HIGH	LOW	LOW	LOW	HIGH	LOW	LOW	HIGH	LOW	LOW
Abdulghani SH 2021	HIGH	HIGH	LOW	LOW	LOW	LOW	LOW	LOW	LOW	LOW	LOW
Abedzadeh-Kalahroudi M 2021	HIGH	HIGH	LOW	LOW	LOW	LOW	LOW	LOW	LOW	LOW	LOW
Adhikari EH 2020	HIGH	HIGH	LOW	LOW	LOW	LOW	LOW	LOW	LOW	LOW	LOW
Agarwal N 2021	HIGH	HIGH	LOW	LOW	LOW	LOW	LOW	LOW	HIGH	LOW	LOW
Ajith S 2021	HIGH	HIGH	LOW	LOW	LOW	HIGH	LOW	LOW	LOW	HIGH	MODERATE
Al-Matary A 2021	HIGH	HIGH	LOW	LOW	LOW	LOW	LOW	LOW	HIGH	LOW	LOW
Alay I 2020	HIGH	HIGH	LOW	LOW	LOW	LOW	LOW	LOW	HIGH	LOW	LOW
Aliaga CD (1) 2020	HIGH	LOW	LOW	LOW	LOW	LOW	LOW	LOW	LOW	LOW	LOW
Alnashry LM 2021	HIGH	HIGH	LOW	LOW	LOW	HIGH	LOW	LOW	LOW	LOW	LOW
Anand P 2020	HIGH	HIGH	LOW	LOW	LOW	LOW	LOW	LOW	LOW	LOW	LOW
Angelidou A 2021	HIGH	HIGH	LOW	LOW	LOW	LOW	LOW	LOW	LOW	LOW	LOW
Antsaklis P 2021	HIGH	HIGH	LOW	LOW	LOW	LOW	LOW	LOW	HIGH	LOW	LOW
Arakaki T 2021	LOW	LOW	LOW	HIGH	LOW	LOW	LOW	LOW	HIGH	LOW	LOW
Argueta LB 2021	HIGH	HIGH	LOW	LOW	LOW	HIGH	LOW	LOW	LOW	LOW	LOW
Arora D 2021	HIGH	LOW	LOW	LOW	LOW	HIGH	LOW	LOW	LOW	LOW	LOW
Artymuk N 2021	HIGH	HIGH	HIGH	LOW	LOW	HIGH	LOW	LOW	HIGH	LOW	MODERATE

Askary E 2020	HIGH	HIGH	LOW	LOW	LOW	LOW	LOW	LOW	HIGH	LOW	LOW
Aslan MM 2020	HIGH	HIGH	LOW	LOW	LOW	LOW	LOW	LOW	HIGH	LOW	LOW
Ayed A 2020	HIGH	HIGH	LOW	LOW	LOW	LOW	LOW	LOW	HIGH	LOW	LOW
Barber E 2021	HIGH	HIGH	LOW	LOW	LOW	HIGH	LOW	LOW	HIGH	LOW	MODERATE
Beharier O 2021	LOW	HIGH	LOW	LOW	LOW	HIGH	LOW	LOW	LOW	LOW	LOW
Bender WR (1) 2020	HIGH	LOW	LOW	LOW	LOW	LOW	LOW	LOW	HIGH	LOW	LOW
Berry M 2021	HIGH	LOW	LOW	LOW	LOW	LOW	LOW	LOW	HIGH	LOW	LOW
Bertero L (1) 2021	HIGH	HIGH	LOW	LOW	LOW	HIGH	LOW	LOW	LOW	LOW	LOW
Bertino E 2020	HIGH	HIGH	HIGH	LOW	LOW	LOW	LOW	LOW	LOW	LOW	LOW
Biasucci G 2020	HIGH	HIGH	HIGH	LOW	LOW	LOW	LOW	LOW	LOW	LOW	LOW
Bozkurt F 2021	HIGH	HIGH	HIGH	LOW	LOW	LOW	LOW	LOW	HIGH	LOW	MODERATE
Brandt JS 2020	HIGH	HIGH	LOW	LOW	LOW	LOW	LOW	LOW	HIGH	LOW	LOW
Briana DD 2021	HIGH	HIGH	LOW	LOW	LOW	HIGH	LOW	LOW	LOW	LOW	LOW
Brito I 2021	HIGH	HIGH	LOW	LOW	LOW	LOW	LOW	LOW	LOW	LOW	LOW
Buhimschi CS 2020	HIGH	LOW	LOW	LOW	LOW	LOW	LOW	LOW	LOW	LOW	LOW
Cakirca TD 2021	HIGH	HIGH	LOW	LOW	LOW	LOW	LOW	LOW	HIGH	HIGH	MODERATE
Cardona-Perez JA 2021	HIGH	LOW	LOW	LOW	LOW	LOW	LOW	LOW	HIGH	HIGH	LOW
Chaichian S 2021	HIGH	HIGH	LOW	LOW	LOW	LOW	LOW	LOW	HIGH	LOW	LOW
Charki S 2021	HIGH	LOW	LOW	LOW	LOW	LOW	LOW	LOW	LOW	LOW	LOW
Cheng B 2020	HIGH	HIGH	HIGH	LOW	LOW	LOW	LOW	HIGH	HIGH	LOW	MODERATE
Chowdhury L 2021	HIGH	HIGH	LOW	LOW	LOW	LOW	LOW	LOW	HIGH	LOW	LOW
Clemente MJ 2021	HIGH	HIGH	LOW	LOW	LOW	HIGH	LOW	LOW	HIGH	LOW	MODERATE
Cojocaru L 2020	HIGH	HIGH	LOW	LOW	LOW	LOW	LOW	LOW	HIGH	LOW	LOW
Colson A 2021	HIGH	HIGH	LOW	LOW	LOW	HIGH	LOW	LOW	LOW	LOW	LOW
Conti MG 2021	HIGH	LOW	LOW	LOW	LOW	LOW	LOW	LOW	LOW	LOW	LOW
Cosma S (2) 2021	HIGH	HIGH	LOW	LOW	LOW	LOW	LOW	LOW	LOW	LOW	LOW
Cribiu FM (1) 2021	HIGH	LOW	LOW	LOW	LOW	HIGH	LOW	LOW	LOW	HIGH	LOW
Cubas JAC (1) 2020	HIGH	HIGH	LOW	LOW	LOW	HIGH	LOW	LOW	LOW	LOW	LOW
de Fatima Yukie Maeda M 2021	HIGH	HIGH	LOW	LOW	LOW	HIGH	LOW	LOW	LOW	LOW	LOW

de Vasconcelos Gaspar A 2021	HIGH	HIGH	LOW	LOW	LOW	HIGH	LOW	LOW	LOW	LOW	LOW
Dhuyvetter A 2020	HIGH	HIGH	LOW	LOW	LOW	LOW	LOW	LOW	HIGH	LOW	LOW
Diaz-Corvillon P 2020	HIGH	LOW	LOW	LOW	LOW	LOW	LOW	LOW	LOW	LOW	LOW
Dingom MAN 2020	HIGH	HIGH	LOW	LOW	LOW	LOW	LOW	LOW	HIGH	LOW	LOW
Donadieu D 2020	HIGH	HIGH	LOW	LOW	LOW	HIGH	LOW	LOW	HIGH	LOW	MODERATE
Doria M 2020	HIGH	LOW	LOW	LOW	LOW	HIGH	LOW	LOW	HIGH	LOW	LOW
Dumitriu D 2020	HIGH	HIGH	LOW	LOW	LOW	LOW	LOW	LOW	LOW	LOW	LOW
Egerup P 2020	HIGH	LOW	LOW	LOW	LOW	LOW	LOW	LOW	LOW	LOW	LOW
Elenga N 2021	HIGH	LOW	LOW	LOW	LOW	LOW	LOW	LOW	HIGH	LOW	LOW
ElHalik M 2020	HIGH	LOW	LOW	LOW	LOW	LOW	LOW	LOW	LOW	LOW	LOW
Facchetti F 2020	HIGH	HIGH	HIGH	LOW	LOW	HIGH	LOW	LOW	LOW	LOW	MODERATE
Farghaly MAA 2020	HIGH	HIGH	HIGH	LOW	LOW	LOW	LOW	LOW	LOW	LOW	LOW
Farhat AS 2020	HIGH	HIGH	HIGH	LOW	LOW	HIGH	LOW	LOW	LOW	LOW	MODERATE
Fenizia C 2020	HIGH	HIGH	LOW	LOW	LOW	LOW	LOW	LOW	LOW	LOW	LOW
Ferrazzi E (1) 2020	HIGH	HIGH	LOW	LOW	LOW	LOW	LOW	LOW	LOW	LOW	LOW
Flannery DD (1) 2020	HIGH	LOW	LOW	LOW	LOW	HIGH	LOW	LOW	LOW	LOW	LOW
Flores-Pliego A 2021	HIGH	LOW	HIGH	LOW	LOW	HIGH	LOW	LOW	LOW	LOW	LOW
Gao J 2020	HIGH	HIGH	LOW	LOW	LOW	LOW	LOW	LOW	LOW	LOW	LOW
Garcia-Ruiz I 2021	HIGH	HIGH	LOW	LOW	LOW	LOW	LOW	LOW	LOW	LOW	LOW
Ghema K 2021	HIGH	HIGH	LOW	LOW	LOW	LOW	LOW	LOW	LOW	LOW	LOW
Grechukina O 2020	HIGH	HIGH	LOW	LOW	LOW	LOW	LOW	LOW	HIGH	LOW	LOW
Griffin I 2020	HIGH	HIGH	LOW	LOW	LOW	HIGH	LOW	LOW	HIGH	LOW	MODERATE
Gulersen M (1) 2020	HIGH	LOW	LOW	LOW	LOW	LOW	LOW	LOW	LOW	LOW	LOW
Hadar E 2021	HIGH	HIGH	HIGH	HIGH	LOW	HIGH	LOW	LOW	HIGH	LOW	MODERATE
Halici-Ozturk F 2021	HIGH	LOW	LOW	LOW	LOW	HIGH	LOW	LOW	LOW	LOW	LOW
Hassan N 2020	HIGH	HIGH	LOW	LOW	LOW	LOW	LOW	LOW	LOW	LOW	LOW
Haye MT 2021	HIGH	HIGH	LOW	LOW	LOW	LOW	LOW	LOW	HIGH	HIGH	MODERATE
Hazari K 2020	HIGH	LOW	LOW	LOW	LOW	LOW	LOW	LOW	HIGH	HIGH	LOW
Hcini N 2020	HIGH	LOW	LOW	LOW	LOW	LOW	LOW	LOW	HIGH	LOW	LOW

Hernandez OB 2020	LOW	HIGH	LOW	LOW	LOW	LOW	LOW	LOW	HIGH	LOW	LOW
Hosseini MS 2021	HIGH	HIGH	LOW	LOW	LOW	HIGH	LOW	LOW	LOW	LOW	LOW
Hu X (1) 2020	HIGH	HIGH	LOW	LOW	LOW	LOW	LOW	LOW	LOW	LOW	LOW
Huang W 2020	HIGH	HIGH	HIGH	HIGH	LOW	HIGH	LOW	LOW	HIGH	LOW	MODERATE
Huerta Saenz IH 2020	HIGH	HIGH	LOW	LOW	LOW	LOW	LOW	LOW	HIGH	LOW	LOW
Jang WK 2021	HIGH	HIGH	LOW	LOW	LOW	LOW	LOW	LOW	LOW	LOW	LOW
Jani S 2021	HIGH	HIGH	LOW	LOW	LOW	HIGH	LOW	LOW	LOW	LOW	LOW
Janssen O 2020	HIGH	LOW	LOW	LOW	LOW	LOW	LOW	LOW	LOW	LOW	LOW
Joseph NT (3) 2021	HIGH	LOW	LOW	LOW	LOW	LOW	LOW	LOW	LOW	LOW	LOW
Joshi SD 2020	HIGH	HIGH	LOW	LOW	LOW	LOW	LOW	LOW	LOW	LOW	LOW
Kalamdani P 2020	HIGH	HIGH	LOW	LOW	LOW	LOW	LOW	LOW	LOW	LOW	LOW
Kamali A 2021	HIGH	HIGH	LOW	LOW	LOW	LOW	LOW	LOW	HIGH	LOW	LOW
Karasu D 2021	HIGH	HIGH	LOW	LOW	LOW	HIGH	LOW	LOW	LOW	LOW	LOW
Kayem G 2020	HIGH	HIGH	LOW	LOW	LOW	LOW	LOW	LOW	HIGH	LOW	LOW
Kest H 2020	HIGH	HIGH	LOW	LOW	LOW	HIGH	LOW	LOW	LOW	LOW	LOW
Khan S (1) 2020	HIGH	HIGH	LOW	LOW	LOW	HIGH	LOW	LOW	LOW	LOW	LOW
Khoury R 2020	HIGH	HIGH	LOW	LOW	LOW	LOW	LOW	LOW	LOW	LOW	LOW
Khushdil A 2021	HIGH	HIGH	LOW	LOW	LOW	LOW	LOW	LOW	LOW	LOW	LOW
Knight M 2020	LOW	HIGH	LOW	LOW	LOW	LOW	LOW	LOW	HIGH	LOW	LOW
Korkmaz MF 2020	HIGH	HIGH	LOW	LOW	LOW	LOW	LOW	LOW	LOW	LOW	LOW
Kumari K 2021	HIGH	LOW	LOW	LOW	LOW	LOW	LOW	LOW	LOW	LOW	LOW
Lang LK 2020	HIGH	HIGH	LOW	LOW	LOW	HIGH	LOW	LOW	HIGH	LOW	MODERATE
Levitan D 2021	HIGH	HIGH	LOW	LOW	LOW	HIGH	LOW	LOW	LOW	LOW	LOW
Liu P 2020	HIGH	HIGH	LOW	LOW	LOW	LOW	LOW	LOW	LOW	LOW	LOW
Lizama O 2021	HIGH	HIGH	LOW	LOW	LOW	LOW	LOW	LOW	LOW	LOW	LOW
Llorca J 2020	HIGH	HIGH	LOW	LOW	LOW	LOW	LOW	LOW	HIGH	LOW	LOW
Lokken EM (1) 2021	HIGH	HIGH	LOW	LOW	LOW	HIGH	LOW	LOW	HIGH	HIGH	MODERATE
Luo Q 2020	HIGH	HIGH	LOW	LOW	LOW	HIGH	LOW	LOW	LOW	LOW	LOW
Mahajan N (2) 2020	HIGH	HIGH	LOW	LOW	LOW	LOW	LOW	LOW	HIGH	LOW	LOW
Malik S 2021	HIGH	HIGH	LOW	LOW	LOW	LOW	LOW	LOW	LOW	LOW	LOW

Malshe N 2021	HIGH	HIGH	LOW	LOW	LOW	HIGH	LOW	LOW	LOW	LOW	LOW
Mand N 2021	LOW	HIGH	LOW	LOW	LOW	HIGH	LOW	LOW	LOW	LOW	LOW
Maraschini A 2020	LOW	HIGH	LOW	LOW	LOW	LOW	LOW	HIGH	LOW	LOW	LOW
Marin Gabriel MA (2) 2020	HIGH	HIGH	LOW	LOW	LOW	LOW	LOW	LOW	LOW	LOW	LOW
Martinez-Perez O 2020	HIGH	HIGH	LOW	LOW	HIGH	LOW	LOW	HIGH	LOW	LOW	MODERATE
Masmejan S 2020	HIGH	HIGH	LOW	LOW	LOW	HIGH	LOW	LOW	LOW	LOW	LOW
Mattar CNZ 2020	LOW	LOW	LOW	LOW	LOW	LOW	LOW	LOW	HIGH	LOW	LOW
Mattern J 2020	HIGH	LOW	LOW	LOW	LOW	LOW	LOW	LOW	HIGH	LOW	LOW
Mohaghegh Z 2021	HIGH	HIGH	LOW	LOW	LOW	LOW	LOW	LOW	LOW	LOW	LOW
Molina EO 2020	HIGH	HIGH	LOW	LOW	LOW	LOW	LOW	LOW	HIGH	LOW	LOW
Moreira LMO 2021	HIGH	LOW	LOW	LOW	LOW	HIGH	LOW	LOW	LOW	LOW	LOW
Morhart P 2021	HIGH	HIGH	HIGH	LOW	LOW	HIGH	LOW	LOW	LOW	LOW	LOW
Morioka I 2021	LOW	HIGH	LOW	LOW	LOW	LOW	LOW	LOW	HIGH	LOW	LOW
Mourad M (1) 2021	HIGH	HIGH	LOW	LOW	LOW	HIGH	LOW	LOW	LOW	LOW	LOW
Murphy C 2020	HIGH	HIGH	LOW	LOW	LOW	HIGH	LOW	LOW	LOW	LOW	LOW
Nanavati R 2021	HIGH	HIGH	LOW	LOW	LOW	LOW	LOW	LOW	LOW	LOW	LOW
Nayak M 2021	HIGH	HIGH	LOW	LOW	LOW	LOW	LOW	LOW	LOW	LOW	LOW
Nethoss Juni 2020	LOW	HIGH	LOW	LOW	HIGH	HIGH	LOW	HIGH	HIGH	LOW	MODERATE
Ngalame AN 2020	HIGH	HIGH	LOW	LOW	LOW	LOW	LOW	LOW	HIGH	LOW	LOW
Nizyaeva NV 2021	HIGH	HIGH	LOW	LOW	LOW	HIGH	LOW	LOW	HIGH	LOW	MODERATE
Norman M 2021	LOW	HIGH	LOW	LOW	LOW	LOW	LOW	LOW	LOW	LOW	LOW
Ogamba I 2020	HIGH	LOW	LOW	LOW	LOW	LOW	LOW	LOW	HIGH	LOW	LOW
Omrani AS 2020	LOW	HIGH	LOW	LOW	LOW	HIGH	LOW	LOW	HIGH	LOW	LOW
Oncel MY 2020	LOW	HIGH	LOW	LOW	LOW	LOW	LOW	LOW	LOW	LOW	LOW
Oxana Z 2021	HIGH	HIGH	LOW	LOW	LOW	HIGH	LOW	LOW	LOW	LOW	LOW
Ozsurmeli M 2021	HIGH	HIGH	LOW	LOW	LOW	LOW	LOW	LOW	HIGH	LOW	LOW
Patanè L 2020	HIGH	LOW	LOW	LOW	LOW	HIGH	LOW	LOW	LOW	LOW	LOW
Patberg ET 2020	HIGH	LOW	LOW	LOW	LOW	HIGH	LOW	LOW	LOW	LOW	LOW
Pathak S 2020	HIGH	HIGH	HIGH	LOW	LOW	HIGH	LOW	LOW	LOW	LOW	MODERATE

Pawar R 2021	HIGH	HIGH	LOW	LOW	LOW	HIGH	LOW	LOW	LOW	LOW	LOW
Pecks U 2020	LOW	HIGH	LOW	LOW	LOW	HIGH	LOW	LOW	HIGH	LOW	LOW
Penfield C 2020	HIGH	LOW	HIGH	LOW	LOW	HIGH	LOW	LOW	HIGH	LOW	MODERATE
Pineles BL 2020	HIGH	LOW	LOW	LOW	LOW	HIGH	LOW	LOW	LOW	LOW	LOW
Pissarra S 2020	HIGH	HIGH	LOW	LOW	LOW	HIGH	LOW	LOW	LOW	LOW	LOW
Poon L 2021	HIGH	HIGH	LOW	LOW	LOW	LOW	LOW	LOW	HIGH	HIGH	MODERATE
Prasad A 2021	HIGH	HIGH	LOW	LOW	LOW	HIGH	LOW	LOW	LOW	LOW	LOW
Preßler J 2020	HIGH	HIGH	LOW	LOW	LOW	HIGH	LOW	LOW	HIGH	LOW	MODERATE
Puneet G 2021	HIGH	LOW	LOW	LOW	LOW	LOW	LOW	LOW	LOW	LOW	LOW
Qadri F 2020	HIGH	HIGH	LOW	LOW	LOW	HIGH	LOW	LOW	HIGH	LOW	MODERATE
Rathberger K 2021	HIGH	LOW	LOW	LOW	LOW	LOW	LOW	LOW	LOW	LOW	LOW
Rebutini PZ 2021	HIGH	HIGH	LOW	LOW	LOW	LOW	LOW	LOW	LOW	LOW	LOW
Remaeus K 2020	HIGH	HIGH	LOW	LOW	LOW	HIGH	LOW	LOW	LOW	LOW	LOW
Resta L 2021	HIGH	LOW	LOW	LOW	LOW	HIGH	LOW	LOW	LOW	LOW	LOW
Romagano MP 2020	HIGH	HIGH	LOW	LOW	LOW	LOW	LOW	LOW	HIGH	HIGH	MODERATE
Rosen H 2021	HIGH	LOW	LOW	LOW	LOW	LOW	LOW	LOW	HIGH	LOW	LOW
Salvatore CM 2020	HIGH	HIGH	LOW	LOW	LOW	LOW	LOW	LOW	HIGH	LOW	LOW
Santhosh J 2020	HIGH	HIGH	LOW	LOW	LOW	HIGH	LOW	LOW	HIGH	HIGH	MODERATE
Sastry SR 2020	HIGH	LOW	LOW	LOW	LOW	HIGH	LOW	LOW	HIGH	LOW	LOW
Sattari M 2020	HIGH	HIGH	LOW	LOW	LOW	LOW	LOW	LOW	HIGH	LOW	LOW
Sayeed SK 2021	HIGH	HIGH	LOW	LOW	LOW	LOW	LOW	LOW	HIGH	LOW	LOW
Schwartz DA (3) 2020	HIGH	HIGH	HIGH	LOW	LOW	LOW	LOW	LOW	LOW	LOW	LOW
Sehra R 2021	HIGH	HIGH	LOW	LOW	LOW	LOW	LOW	LOW	LOW	LOW	LOW
Semeshkin AA 2020	HIGH	LOW	HIGH	LOW	LOW	LOW	LOW	LOW	LOW	LOW	LOW
Servei Catala 29/05	LOW	HIGH	LOW	LOW	LOW	HIGH	LOW	LOW	HIGH	HIGH	MODERATE
Shah PT 2020	HIGH	HIGH	HIGH	LOW	LOW	LOW	LOW	LOW	HIGH	LOW	MODERATE
Sharma N 2021	HIGH	LOW	LOW	LOW	LOW	LOW	LOW	LOW	HIGH	LOW	LOW
Sharma R 2021	HIGH	LOW	LOW	LOW	LOW	HIGH	LOW	LOW	LOW	LOW	LOW
Shmakov R 2020	HIGH	LOW	LOW	LOW	LOW	LOW	LOW	LOW	HIGH	LOW	LOW
Shook LL (1) 2021	HIGH	HIGH	LOW	LOW	LOW	HIGH	LOW	LOW	LOW	LOW	LOW

Sibia P 2021	HIGH	HIGH	LOW	LOW	LOW	HIGH	LOW	LOW	HIGH	HIGH	MODERATE
Sinaci S 2021	HIGH	HIGH	LOW	LOW	LOW	LOW	LOW	LOW	LOW	LOW	LOW
Singh V (1) 2021	HIGH	HIGH	LOW	LOW	LOW	LOW	LOW	LOW	HIGH	HIGH	MODERATE
Sola A 2020	LOW	HIGH	HIGH	LOW	LOW	LOW	LOW	LOW	LOW	LOW	LOW
Solis-Garcia G 2020	HIGH	HIGH	LOW	LOW	LOW	LOW	LOW	LOW	LOW	LOW	LOW
Song D 2021	HIGH	LOW	LOW	LOW	LOW	LOW	LOW	LOW	LOW	LOW	LOW
Sri Sri G 2020	HIGH	LOW	LOW	LOW	LOW	HIGH	LOW	LOW	HIGH	LOW	LOW
Steffen HA 2021	HIGH	LOW	LOW	LOW	LOW	HIGH	LOW	LOW	LOW	LOW	LOW
Suyuthi FP 2021	HIGH	HIGH	LOW	LOW	LOW	LOW	LOW	LOW	LOW	LOW	LOW
Tadas MP 2021	HIGH	HIGH	LOW	LOW	LOW	LOW	LOW	LOW	LOW	LOW	LOW
Tallarek A 2021	HIGH	HIGH	HIGH	LOW	LOW	HIGH	LOW	LOW	LOW	LOW	LOW
Tang F 2020	HIGH	HIGH	LOW	LOW	LOW	LOW	LOW	LOW	LOW	LOW	LOW
Tasca C 2021	HIGH	HIGH	LOW	LOW	LOW	LOW	LOW	LOW	LOW	LOW	LOW
Teixeira MLB 2021	HIGH	HIGH	LOW	LOW	LOW	HIGH	LOW	LOW	HIGH	LOW	MODERATE
Thanigainathan S 2021	HIGH	HIGH	LOW	LOW	LOW	LOW	LOW	LOW	LOW	LOW	LOW
Vaezi M 2021	HIGH	HIGH	LOW	LOW	LOW	LOW	LOW	LOW	HIGH	LOW	LOW
Vera Loyola EM 2021	HIGH	LOW	LOW	LOW	LOW	LOW	LOW	LOW	LOW	LOW	LOW
Vizheh M 2021	HIGH	HIGH	LOW	LOW	LOW	LOW	LOW	LOW	HIGH	HIGH	MODERATE
Vousden N 2020	LOW	HIGH	LOW	LOW	HIGH	LOW	LOW	LOW	HIGH	HIGH	MODERATE
Wiyati PS 2021	HIGH	HIGH	LOW	LOW	LOW	LOW	LOW	LOW	LOW	LOW	LOW
Wu H 2021	HIGH	HIGH	HIGH	LOW	LOW	HIGH	LOW	LOW	LOW	LOW	LOW
Wu YT 2020	HIGH	HIGH	LOW	LOW	LOW	LOW	LOW	LOW	LOW	LOW	LOW
Xu S (1) 2020	HIGH	HIGH	HIGH	LOW	LOW	LOW	LOW	LOW	HIGH	LOW	MODERATE
Yadav V 2020	HIGH	HIGH	LOW	LOW	LOW	LOW	LOW	LOW	LOW	LOW	LOW
Yaman A 2021	HIGH	HIGH	LOW	LOW	LOW	HIGH	LOW	LOW	LOW	LOW	LOW
Yang H (1) 2020	HIGH	HIGH	LOW	LOW	LOW	LOW	LOW	LOW	LOW	LOW	LOW
Yang H (2) 2020	HIGH	HIGH	HIGH	LOW	LOW	LOW	LOW	HIGH	HIGH	LOW	MODERATE
Yu X 2020	HIGH	HIGH	HIGH	LOW	LOW	HIGH?	LOW	LOW	LOW	LOW	MODERATE
Zaharie G 2020	HIGH	LOW	LOW	LOW	LOW	LOW	LOW	LOW	LOW	LOW	LOW
Zeng L 2020	HIGH	HIGH	HIGH	LOW	LOW	HIGH	LOW	LOW	LOW	LOW	MODERATE

Zgutka K 2021	HIGH	HIGH	LOW	LOW	LOW	LOW	LOW	LOW	LOW	LOW	LOW
Zhang P 2020	HIGH	LOW	LOW	LOW	LOW	HIGH	LOW	LOW	LOW	LOW	LOW
Zlochiver V 2021	HIGH	HIGH	LOW	LOW	LOW	HIGH	LOW	LOW	LOW	LOW	LOW

Appendix 5: Quality assessment for risk of bias in comparative cohort studies using the Newcastle-Ottawa Scale

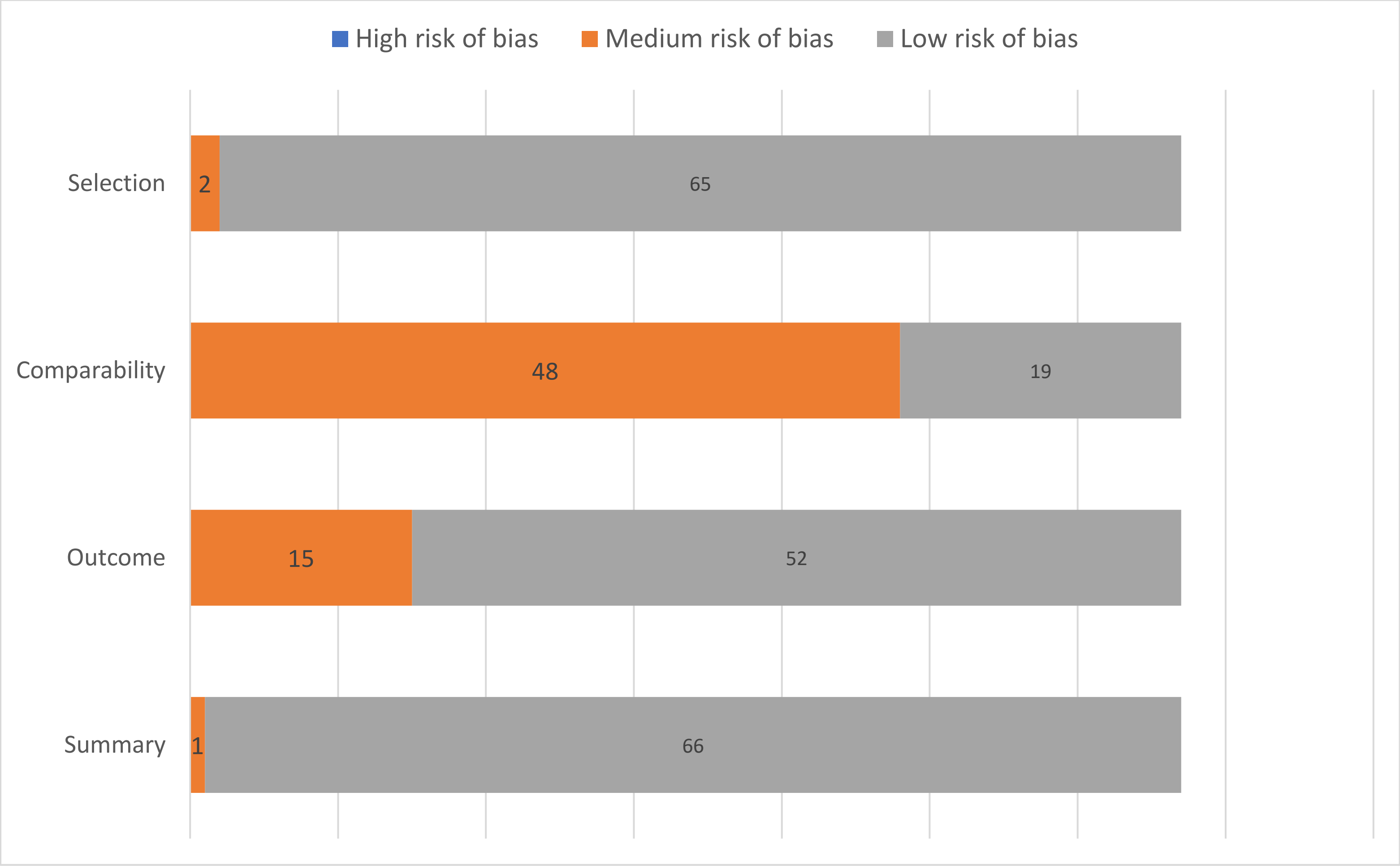

Appendix 6

a: Subgroup analysis by World Bank Region for rates of SARS-CoV-2 positivity in babies by RT-PCR and RT-PCR or anti-SARS-CoV-2 IgM

World Bank Region	Studies	events/N	Proportion (95% CI)	Tau^2	I^2
**North America**					
RT-PCR only	32	19/2229	0.06 (0.00, 0.34)	0.0000	0
RT-PCR or/and IgM	32	25/2364	0.16 (0.00, 0.56)	0.0022	14
**South Asia**					
RT-PCR only	29	126/3365	2.87 (1.18, 5.12)	0.0696	89
RT-PCR or/and IgM	30	126/3378	2.75 (1.09, 4.95)	0.0690	88
**Europe and Central Asia**					
RT-PCR only	38	231/6079	1.50 (0.54, 2.76)	0.0234	75
RT-PCR or/and IgM	39	233/6101	1.59 (0.61, 2.86)	0.0232	75
**Latin America and Caribbean**					
RT-PCR only	14	83/1461	5.68 (3.21, 8.67)	0.0248	71
RT-PCR or/and IgM	15	83/1473	5.34 (2.97, 8.23)	0.0241	69
**East Asia and Pacific**					
RT-PCR only	12	22/502	3.15 (0.47, 7.31)	0.0500	65
RT-PCR or/and IgM	12	28/502	4.27 (0.78, 9.55)	0.0741	74
**Middle east and North Africa**					
RT-PCR only	15	20/635	2.16 (0.26, 5.19)	0.0372	58
RT-PCR or/and IgM	16	24/700	2.42 (0.47, 5.32)	0.0353	59
**Overall**					
RT-PCR only	140	501/14271	1.80 (1.18, 2.51)	0.0348	77
RT-PCR or/and IgM	144	519/14518	1.94 (1.31, 2.66)	0.0352	77

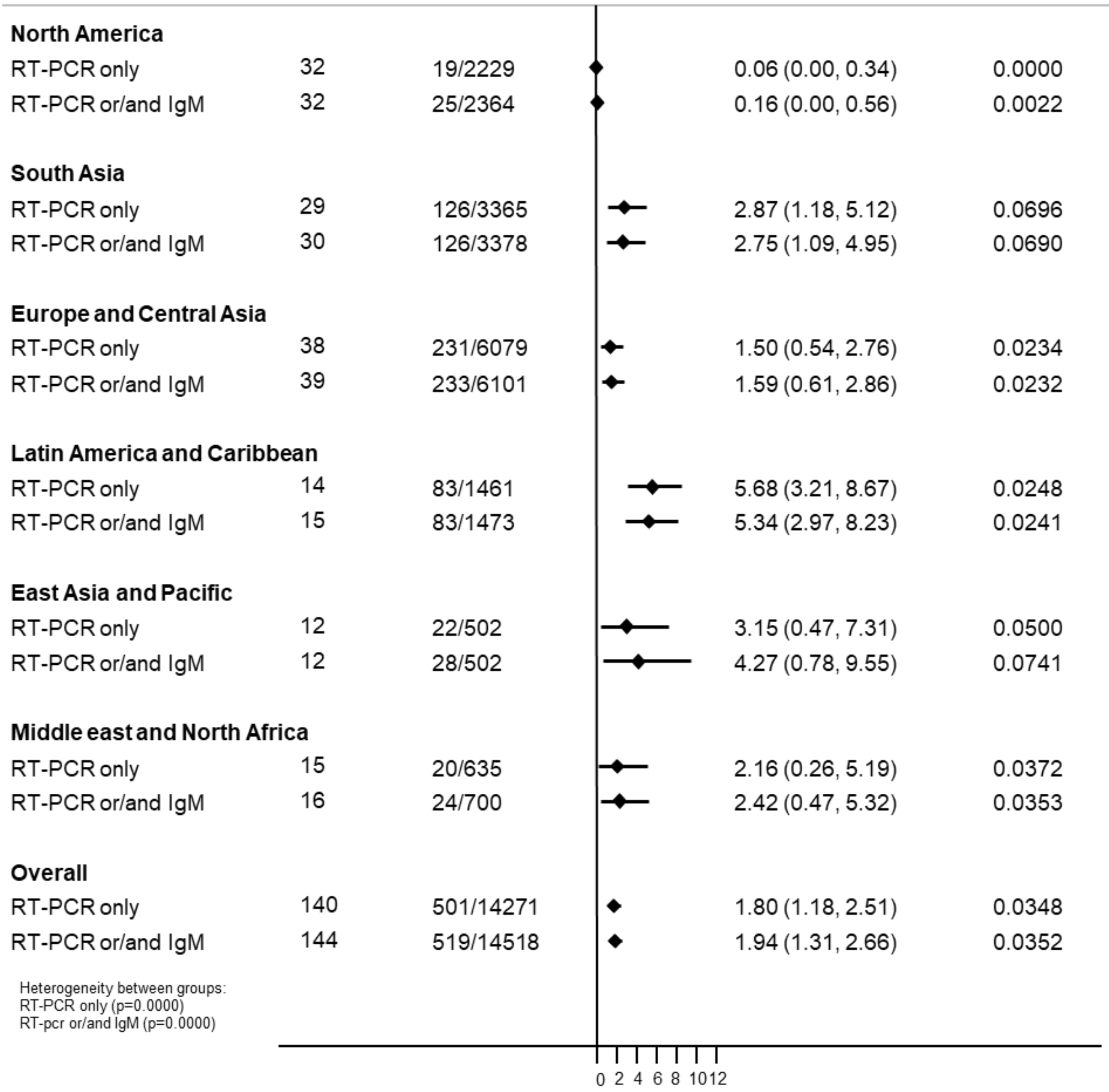

Heterogeneity between groups:
RT-PCR only (p=0.0000)
RT-pcr or/and IgM (p=0.0000)

b: Individual study estimates of SARS-CoV-2 positivity in babies by RT-PCR

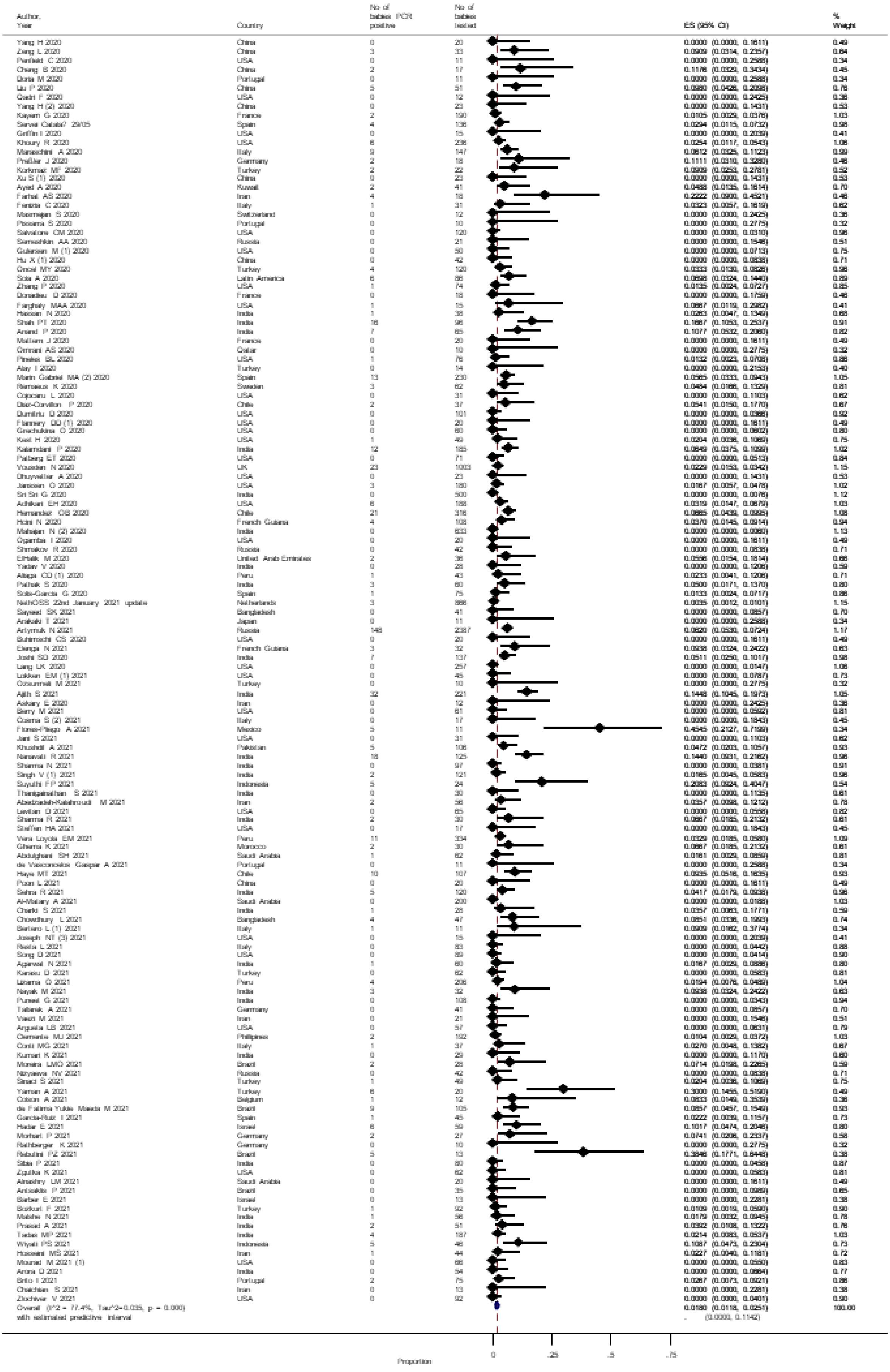

c: Individual study estimates of SARS-CoV-2 positivity in babies by RT-PCR or anti-SARS-CoV-2 IgM

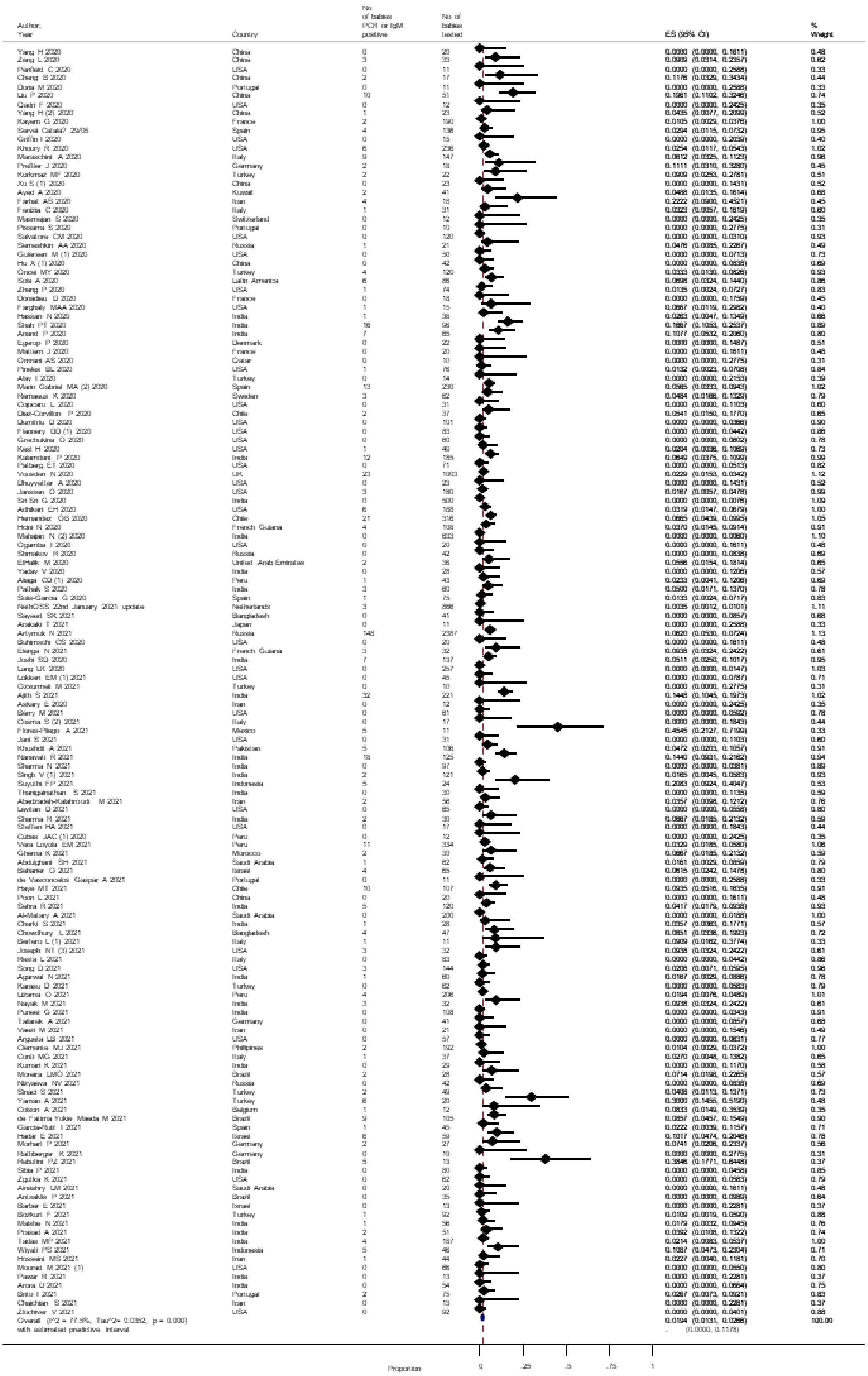

d: Individual study for maternal and perinatal factors associated with SARS-CoV-2 positivity in babies

Trimester of infection in pregnancy (3rd vs 1st or 2nd)

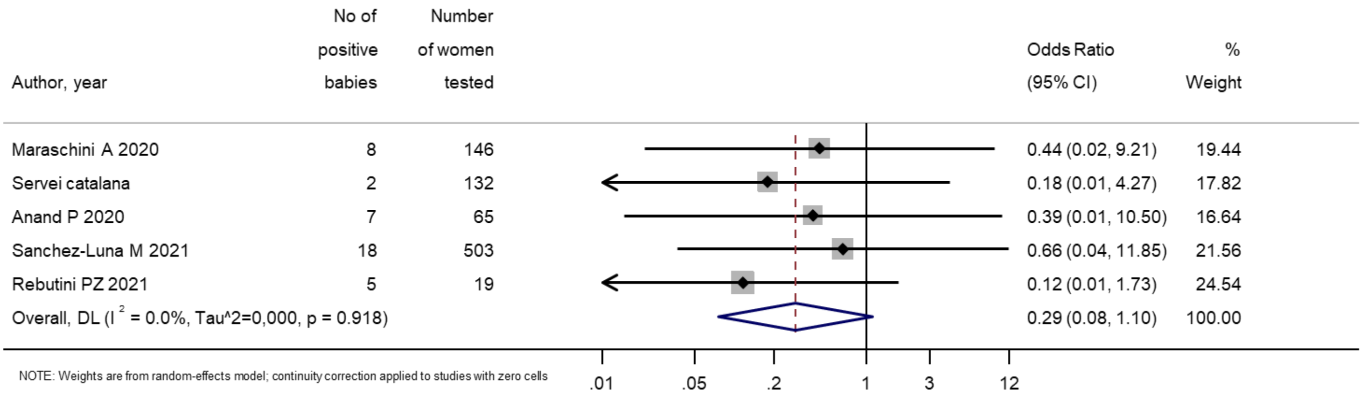

Postnatal vs antenatal infection

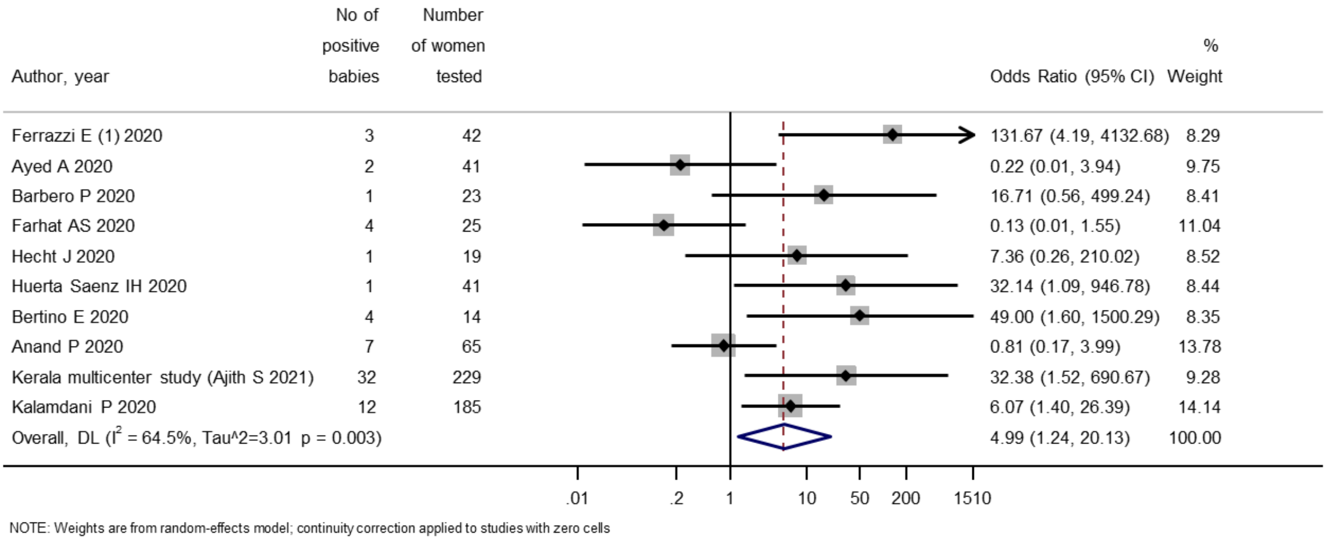

Maternal death

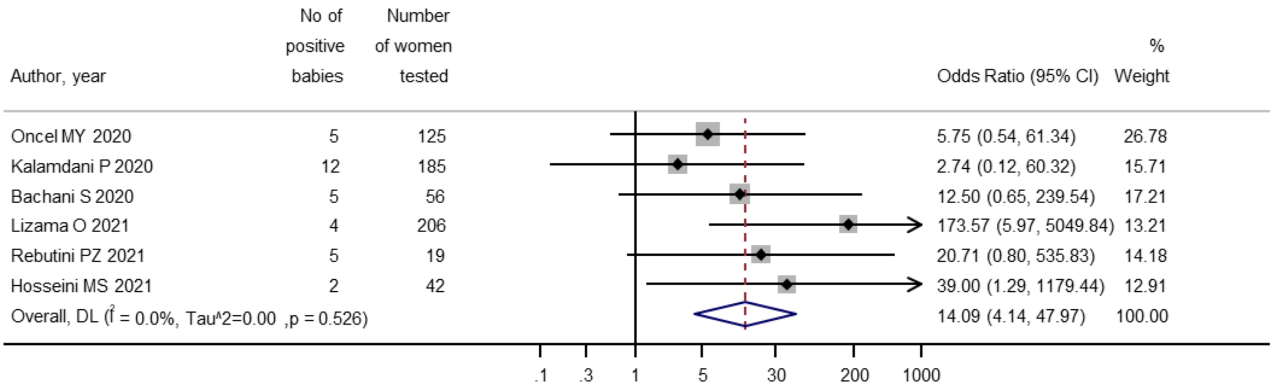

NOTE: Weights are from random-effects model; continuity correction applied to studies with zero cells

Severe COVID-19 in mother

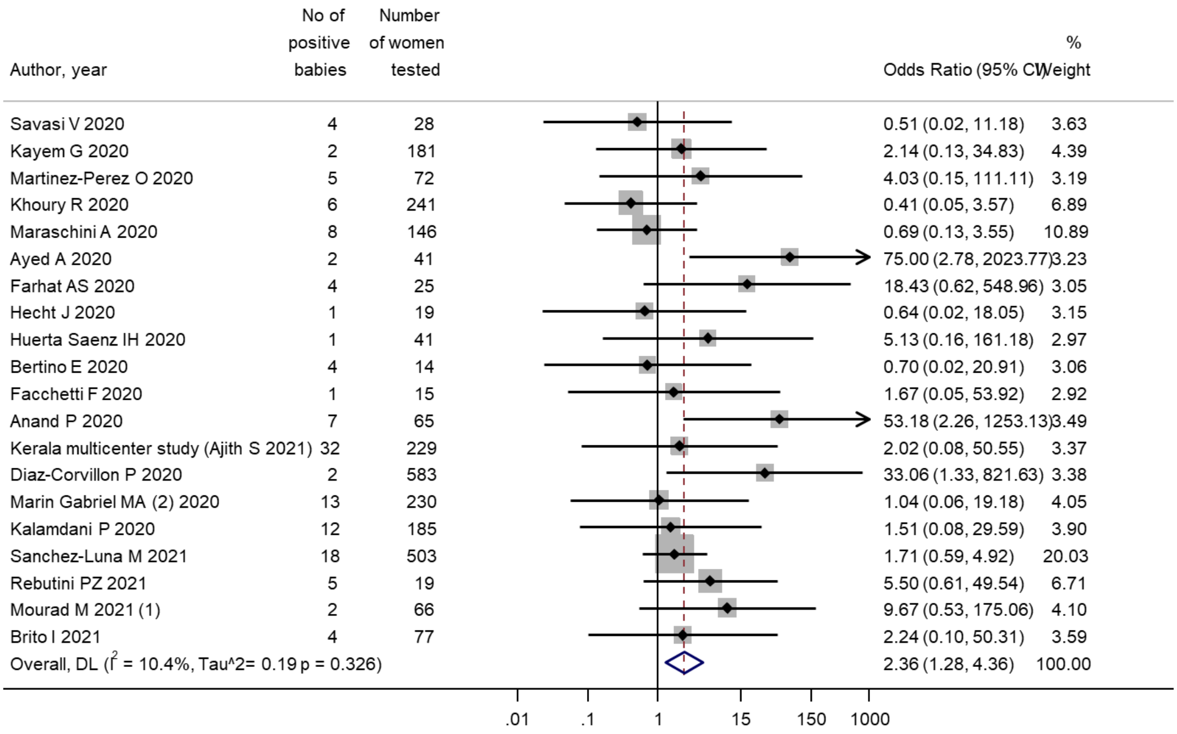

NOTE: Weights are from random-effects model; continuity correction applied to studies with zero cells

ICU admission

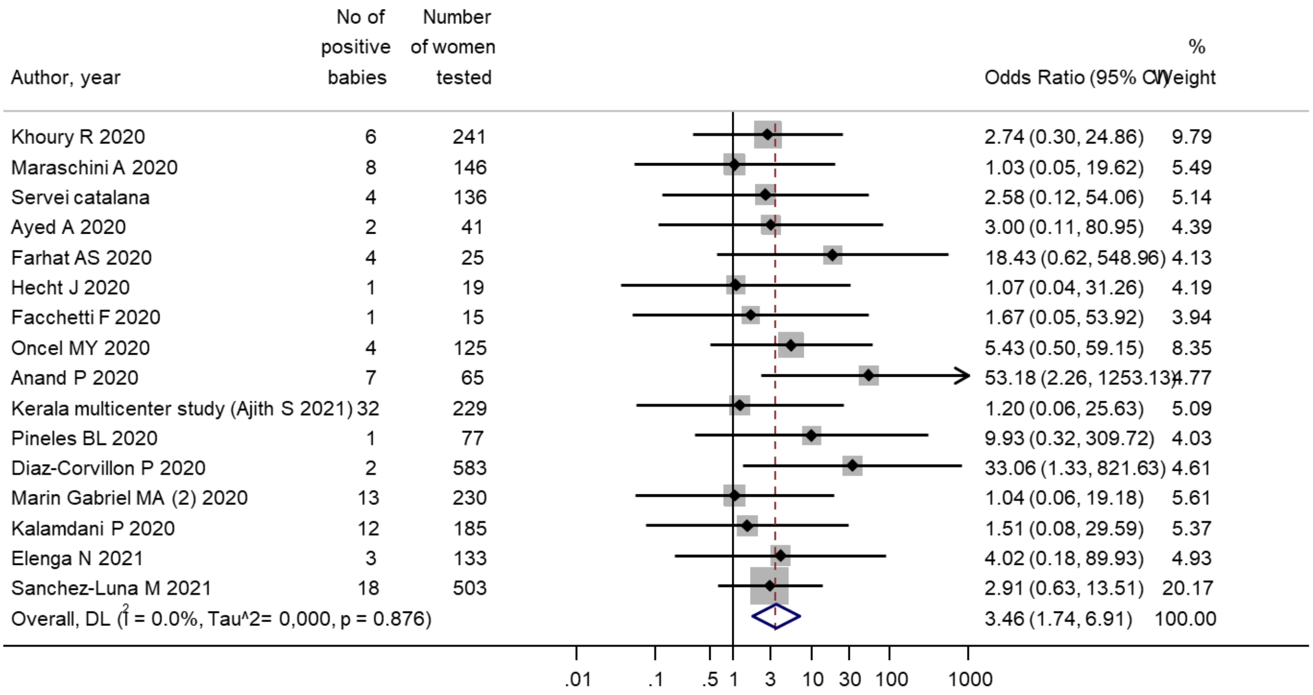

NOTE: Weights are from random-effects model; continuity correction applied to studies with zero cells

Separated at birth

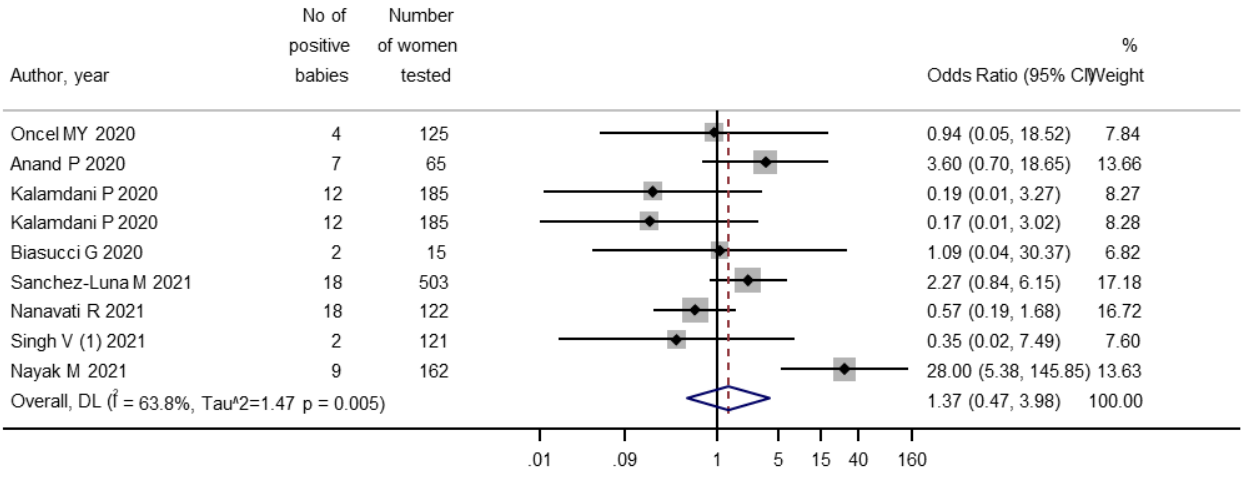

NOTE: Weights are from random-effects model; continuity correction applied to studies with zero cells

Preterm

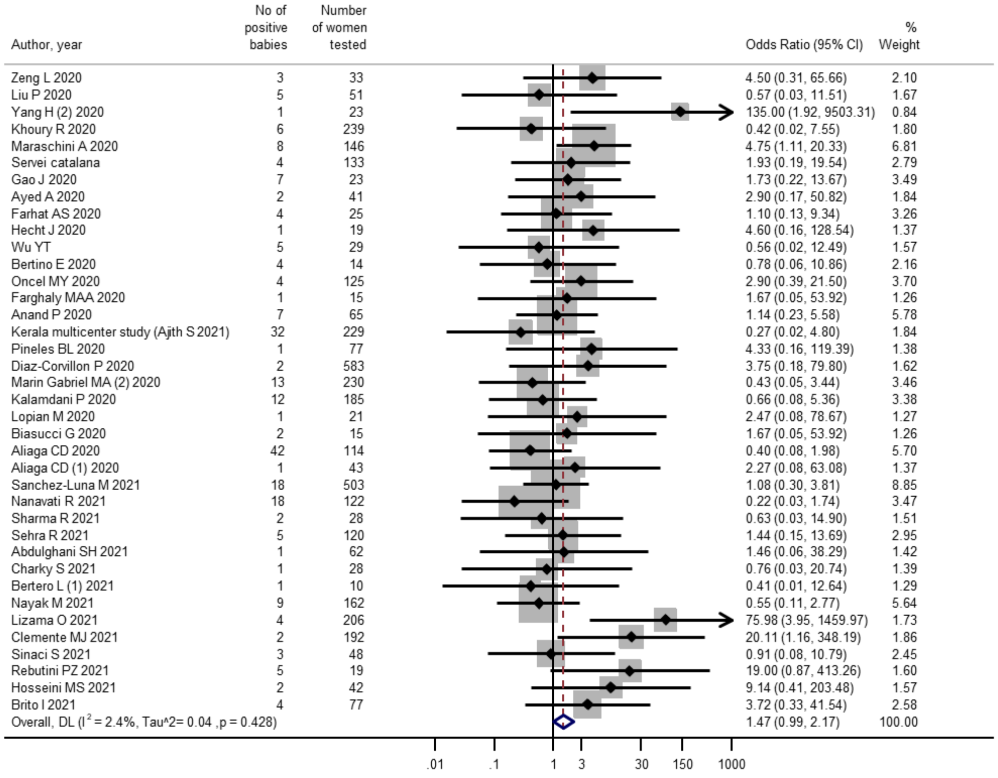

NOTE: Weights are from random-effects model; continuity correction applied to studies with zero cells

Breast fed

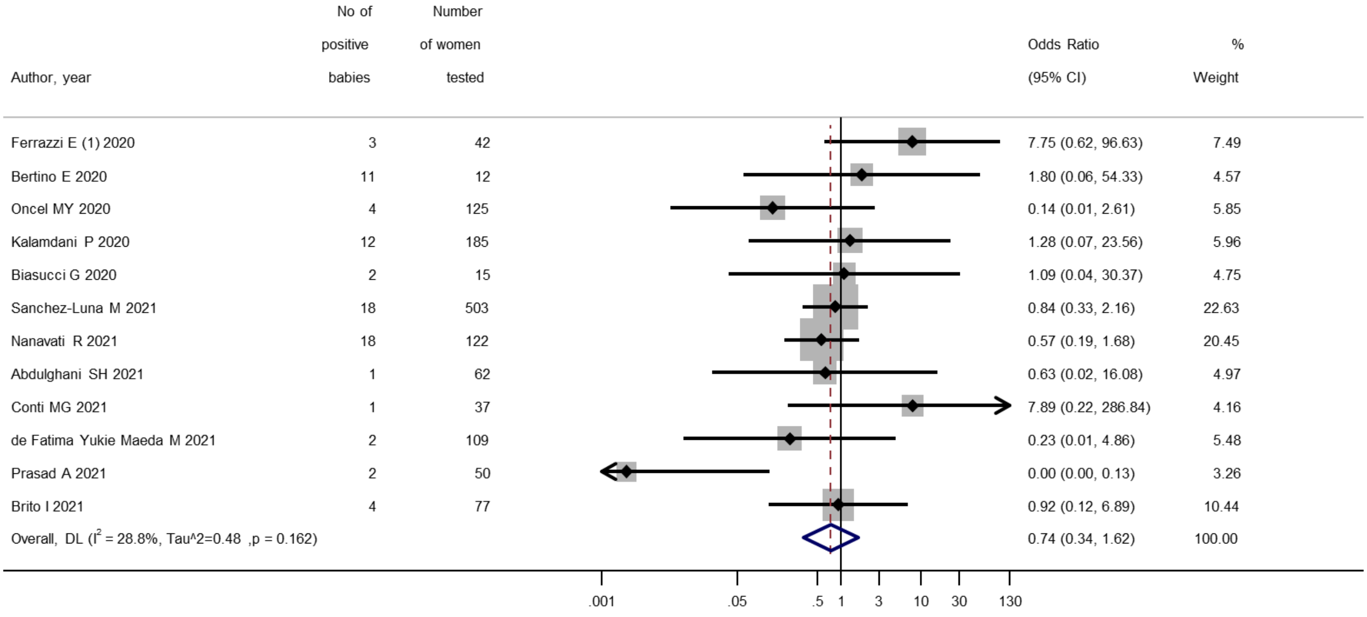

NOTE: Weights are from random-effects model; continuity correction applied to studies with zero cells

Appendix 7: Maternal and perinatal characteristics of babies with possible in utero transmission

Author, year	Maternal characteristics	Mode of delivery	Measures to prevent SARS-CoV-2 MTCT	Tests for SARS-CoV-2 MTCT		Fetal and neonatal characteristics
				**Initial test**	**Further tests**	
**Possible in utero (IUFD) MTCT**						
Agarwal, 2020	30-years-old, G2P1<br>Symptoms of mild fever, cough and sore throat.<br>NP PCR+ at 20 weeks	Induction at 20 weeks due to IUFD	Not reported	Fetal tissue testing (PCR/ISH): not done	Other testing: Fetal swab PCR+	Fetal demise at 20 weeks<br>BW: 340g
Bouachba A, 2021<br><br>(Baby 1)	33-years-old, G2P1.<br>Symptoms of fever.<br>NP PCR+ late pregnancy	Natural	Not reported	Fetal tissue testing (PCR/ISH): not done	Other testing: Placental tissue PCR+ and IHC+.	Fetal demise at 30 weeks
Bouachba A, 2021<br><br>(Baby 2)	30-years-old, G1P0.<br>Symptoms of ageusia and fever.<br>NP PCR+ late pregnancy.	Natural	Not reported	Fetal tissue testing (PCR/ISH): not done	Other testing: Placental tissue IHC+.	Fetal demise at 22 weeks
Bouachba A, 2021<br><br>(Baby 3)	43-years-old, G11P8.<br>Symptoms of fever.<br>IUGR at 26 GW.<br>NP PCR+ late pregnancy	Natural	Not reported	Fetal tissue testing (PCR/ISH): not done	Other testing: Placental tissue PCR+ and IHC+.	Fetal demise at 26 weeks
Flores-Pliego A, 2021	28-years-old, no comorbidities, presented with cough and headache at 13 weeks. NP PCR+ at delivery.	Curettage (abortion) at 13 weeks	No details published	Fetal swab PCR+	Placenta tissue PCR negative	Termination at 13 weeks<br><br>No details published
Libbrecht S, 2021<br><br>(Twin 1)	22-years-old, primipara.<br>Dichorionic diamniotic twin pregnancy.<br>Flu like symptoms and high temperature.<br>NP PCR+ at 35 GW	IUFD at 36 weeks	Not reported	Fetal tissue testing (PCR/ISH): not done.	Placenta PCR+ and ISH+.<br>NP PCR neg (no timing)	Fetal demise at 36 weeks<br><br>(Twin 2 classified as in utero: indeterminate)

Machado S, 2021	39-years-old, G1P0. Symptoms of watery diarrhoea. NP PCR+ after induction	Induction at 35 weeks due to IUFD	Not reported	Fetal tissue testing (PCR/ISH): not done	Other testing: Placental swab PCR+ Fetal NP PCR negative	GA: 35 weeks BW: 1990g Fetal demise
Marton T, 2021	23-years-old, G2P1. Symptomatic NP PCR+ at 25 GW	Induction at 25 weeks due to IUFD	Not reported	Placenta IHC+ and ISH+	Nasal swab not done	BW: 780g Fetal demise at 25 weeks
Popescu DE, 2021	43-years-old Symptoms of fever, fatigue, taste and smell loss/ NP PCR+ at 18 weeks	Vaginal	Not reported	Fetal tissue testing (PCR/ISH): not done	Other testing: Placental tissue IHC+.	Fetal demise at 28 weeks
Pulinx B, 2020 **(twins)**	30-years-old, G2P1, history of gestational diabetes. Symptoms of rhinitis and fever. NP PCR+ at 22 GW. Blood PCR+ at time of delivery	Preterm labour at 24 weeks due to IUFD	Not reported	Fetal tissue testing (PCR/ISH): not done	Other testing: Placental tissues PCR+ and IHC+. Amniotic fluid PCR+ (not sterile collection), amniotic membranes PCR negative	Intrauterine death of twin fetuses at 24 weeks
Rabah R, 2021	Symptom of loss of taste and smell NP PCR+	IUFD at 23 weeks	Not reported	Fetal tissue testing (PCR/ISH): not done	Other testing: Placental tissue IHC+. Fetal NP PCR+	Fetal demise at 23 weeks
Rebutini PZ 2021	42-years-old, severe symptoms. Hypertensive disorder in pregnancy. NP PCR+ during 2nd trimester. Blood IgM+ and Ig+	IUFD at 28 weeks	Not reported	Fetal tissue testing (PCR/ISH): not done	Other testing: Placenta PCR+ Umbilical cord blood PCR+	Fetal demise at 28 weeks
Richtmann R, 2020	32-years-old, nulliparous. Symptoms of rhinorrhea, myalgia and fever. NP	Induction at 29 weeks due to IUFD	Not reported	Fetal tissue testing (PCR/ISH): not done	Other testing: Placental fragments PCR+	Fetal demise at 29 weeks

(baby 1)	PCR+ 14 days before induction					
Richtmann R, 2020<br><br>(baby 2)	30-years-old, nulliparous. Symptoms of rhinorrhoea, fever, anosmia, dysgeusia and headache. NP PCR+ 5 days before induction	Induction at 31 weeks due to IUFD	Not reported	Fetal tissue testing (PCR/ISH): not done	Other testing:<br>Placental PCR+<br>Amniotic fluid (“collected before Fetal extraction”) PCR+	Fetal demise at 31 weeks
Shende P, 2020	Late twenties, third gravida.<br>Asymptomatic, NP PCR+ at 8 weeks	IUFD at 14 weeks	Not reported	Fetal tissue testing (PCR/ISH): not done	Other testing:<br>Placental PCR+ and IHC+.<br>Amniotic membrane PCR+	Fetal demise at 14 weeks
Teixeira MLB, 2021	NP PCR inconclusive, blood IgG+<br>HIV positive	IUFD	Not reported	Fetal tissue testing (PCR/ISH): not done	Other testing:<br>Placental PCR+	Fetal demise
**Possible in utero (liveborn) MTCT**						
Aatif M, 2020	23-years-old, G3 and 2 previous abortions, presented with fever and cough at 37 weeks. NP PCR+ before delivery.	Caesarean section	Mother delivered in negative pressure room whilst wearing N95 mask. Separated from mother until D2. Breastfed from D2.	NP PCR+ at 24h	NP PCR+ on D7 and D14, negativeon D18 and D19<br><br>Peripheral blood IgM+ (rapid antibody testing) on D7	GA: 37 weeks<br>BW: 2800g<br>Apgar: 7, 9<br><br>Neonate observed in NICU, and developed tachypnea (72 breaths per minute) and grunting at 1 hour, but normal CXR. Symptoms resolved on D2. Alive.

Abadía-Cuchi N, 2021	Dichorionic diamniotic twin pregnancy. Symptoms of fever.<br>NP PCR+<br>Blood IgM and IgG +<br>Bilateral interstitial infiltrates on CXR.	Caesarean section	Separated from mother at birth.	NP PCR neg at 0hr<br><br>Placenta swab PCR+	NP PCR+ on D2 and D8<br><br>Blood IgM neg on D2. Blood IgG neg on D2 and D8, + on D19	GA: 38 weeks<br>BW: 2845g<br>APGAR: 2, 3, 8<br>Asymptomatic<br>Normal CXR<br>Alive
Adhikari EH, 2020<br><br>(baby 1)<br><br>(Sisman J 2020)	37-years-old, G4P3 with comorbid diabetes, obesity (BMI: 55) and recent treatment of late latent syphilis. Presented with 1 day history of fever, diarrhoea, and back pain. NP PCR+ before delivery.	Vaginal delivery	Mother wore mask during hospitalisation. Had premature rupture of membranes, delivered 8 hours later. No skin-to-skin contact after delivery, neonate separated from mother and transferred to NICU. Fed expressed colostrum for first three days.	Placenta IHC+ and EM+<br><br>NP PCR+ at 24h	NP PCR+ at 48h and D14	GA: 34 weeks<br>BW: 3280g<br>Apgar: 7, 9<br><br>Managed in NICU for respiratory distress and fever. Normal CXR. Test for syphilis negative.<br>Alive.
Adhikari EH, 2020<br><br>(baby 2)	NP PCR+ during third trimester. No further details published.	Delivered at term, mode unknown	Not reported	NP PCR+ at 24h	NP PCR+ at 48h	Alive. No other details published.
Adhikari EH, 2020<br><br>(baby 3)	NP PCR+ during third trimester. No further details published.	Delivered at term, mode unknown	Not reported	NP PCR+ at 24h	NP PCR+ at 48h	Alive. No other details published.
Alwardi TH, 2020*<br><br>(triplets)	31-years-old female with trichorionic triamniotic triplets conceived by IVF and history of gestational thrombocytopaenia and	Caesarean section	Delivered in negative pressure room. Neonates separated from	All three triplets NP PCR+ at 20h	All three triplets NP PCR+ on D5	GA: 32 weeks<br>BW: 1910g, 1390g, 1630g<br>Apgar: 8, 9 (all triplets)

	hypothyroidism, presented with fever and flu-like symptoms at 32 weeks. NP PCR+ the day before delivery.		mother after birth and not breastfed.			Neonates admitted to NICU for isolation. Twin B required 3 days of intermittent nasal CPAP. Otherwise, all asymptomatic and alive.
Alzamora M, 2020*	41-years-old, G3P2 with obesity and diabetes, presented with a 4 day history of fever, fatigue and malaise at 32 weeks. NP PCR+ and abnormal radiology before delivery. Admitted to ICU and intubated due to severe respiratory distress before delivery.	Caesarean section	Separated from mother after birth, not breastfed.	NP PCR+ at 16h<br><br>IgM negative (immunochromatographic assay) at brith	NP PCR+ at 64h<br><br>IgM negative on D4 and D5	GA: 33 weeks<br>BW: 2970g<br>Apgar: 6, 8<br><br>Neonate intubated after birth in NICU. Normal CXR. Extubated after 12 hours.<br>Alive.
Angelidou A, 2021<br><br>(baby 1)	Symptomatic<br>NP PCR+ during 3rd trimester	Caesarean section	No rooming-in with mother.<br>Not breastfed.	NP PCR+ on D1<br>Placenta IHC+	NP PCR+ on D2 and D5	GA: 35 weeks<br>Symptomatic, respiratory distress<br>Alive
Angelidou A, 2021<br><br>(baby 2)	Symptomatic<br>NP PCR+ during 3rd trimester	Caesarean section	None (rooming-in and breastfeeding was permitted)	NP PCR neg on D1<br>Placenta IHC+	NP PCR+ on D2, neg on D5	GA: 38 weeks<br>Asymptomatic<br>Alive
Angelidou A, 2021	Asymptomatic<br>NP PCR+ during 3rd trimester	Vaginal delivery	None (rooming-in and breastfeeding was permitted)	Placenta IHC+	NP PCR+ on D2	GA: 41 weeks<br>Asymptomatic<br>Alive

(baby 3)						
Carbayo-Jimenez T, 2021	28-years-old, no comorbidities, asymptomatic but partner was positive so was tested. NP PCR+ 8 days before delivery. Also serum PCR+.	Vaginal delivery	Skin-to-skin contact permitted after birth whilst mother wearing mask. Roomed together with 2m separation. Breastfed whilst mother wore mask.	NP PCR+ at 5h	NP PCR+ at 28h and D23.<br><br>Blood IgM+, IgA+ & IgG+ (Chemiluminescent immunoassay) on D23	GA: 38 weeks<br>BW: 'Appropriate for gestational age'<br>Apgar: 9, 10<br><br>Asymptomatic. Alive.
Choobdar FA, 2020	Symptoms of COVID-19<br>Died<br>NP PCR+ at 23 weeks<br>Blood IgM and IgG neg<br>Abnormal radiology	Caesarean section		Placenta PCR+<br>NP PCR+ at 24hr	NP PCR+ on D3, neg on D21 and D28.<br>Peripheral blood IgM and IgG neg on D5 and D14.	GA: 31<br>BW: 1440g<br>Symptomatic, admitted to NICU<br>Alive
Conti MG, 2021	NP PCR+ during 3rd trimester	Not reported	Not reported	NP PCR+ at birth	NP PCR+ on D3, D7 and D10	Alive
de Fatima Yukie Maeda M, 2021<br><br>(Baby 1)	Severe symptoms<br>NP PCR+ AN<br>Blood PCR +<br>Breastmilk PCR neg	Caesarean section	Not reported	Amniotic fluid PCR+<br>Cord blood PCR+	NP PCR + at 48hr, 72hr and on D17; neg on D22<br><br>Blood IgG+ on D23	GA: 33 weeks<br>BW: 2130g<br>APGAR at 5 mins: 9<br>Symptomatic<br>Opacities and atelectasis in right upper lobe on CT.<br>Alive
de Fatima Yukie Maeda M, 2021	Mild symptoms<br>NP PCR+ AN<br>Blood PCR neg<br>Breastmilk PCR+	Vaginal delivery	Not reported	Amniotic fluid PCR neg<br>Cord blood PCR+	NP PCR+ on D2 and at 72hr; neg on D7	GA: 38 weeks<br>BW: 2980g<br>APGAR at 5 mins: 9

(Baby 2)						Alive
Diaz-Corvillon P, 2020*<br><br>(Baby 1)	39-years-old. Asymptomatic, delivered at 40 GW. NP PCR+ due to universal screening.	Not reported	Separated from mother at birth	NP PCR+ at 6hr	NP PCR+ at 72hr	GA: 40 weeks<br>BW: 3840g<br>Apgar: 9, 9<br>Asymptomatic and alive.
Diaz-Corvillon P, 2020*<br><br>(Baby 2)	27-years-old. Asymptomatic, delivered at 38 GW. NP PCR+ due to universal screening.	Not reported	Separated from mother at birth	NP PCR+ at 6hr	NP PCR+ at 72hr	GA: 38 weeks<br>BW: 3510g<br>APGAR: 9, 10<br>Asymptomatic and alive
El Halik M, 2020	Asymptomatic, NP PCR+ 1 day before delivery due to screening at admission for delivery.<br><br>No other details published.	Caesarean section	Neonate roomed-in with mother and directly breastfed.	NP PCR+ at birth	NP PCR+ on D2, D3, D4, D5, then negative on D6 and D7	GA: 36 weeks<br>BW: 2460g<br>Alive.<br><br>No other details published.
Facchetti F, 2020	29-year-old, background of idiopathic thrombocytopaenia, developed a fever at 37 weeks. NP PCR+ and abnormal CXR.	Vaginal delivery	Not reported	Placenta ISH+, IHC+<br><br>NP PCR swab was equivocal at birth	NP PCR+ at 36h, 72h and D17	GA: 37 weeks<br>BW: 2840g<br>Apgar: 9, 10<br><br>Neonate developed fever, difficulty breathing, vomiting, abdominal distension, hypotonia and mild erythema after 24 hours.

						abnormal radiology. Improved and alive.
Farhadi R, 2021	22-years-old, primigravid. Severe symptoms of fever, non-productive cough and myalgia.<br>NP PCR + at 32 GW, COVID-19 pneumonia on lung CT scan.<br>Admitted to ICU, died D15 PN	Caesarean section	Separated from mother at birth. Not breastfed.	NP PCR neg at 2hr, NP PCR+ at 24hr<br><br>Amniotic fluid (sterile collection) PCR+<br>Cord blood PCR neg	NP PCR + on D14, NP PCR neg on D16 and D18	GA: 32 weeks<br>BW: 2350g<br>APGAR: 8, 9<br>Neonate symptomatic and admitted to NICU, required CPAP<br>Features of RDS on CXR<br>Alive
Gao J, 2020<br><br>(Baby 1)	NP PCR+ at 30 weeks<br>IgG+, IgM+	Not reported	Not reported	Peripheral blood IgM+ and IgG+ on D1	Peripheral blood IgM+ on D7, D14, D28<br><br>NP PCR negative (timing unclear)	GA: 38 weeks<br><br>Asymptomatic<br>Alive
Gao J, 2020<br><br>(Baby 2)	NP PCR+ at 30 weeks<br>IgG+, IgM+	Not reported	Not reported	Peripheral blood IgM+ and IgG+ on D1	Peripheral blood IgM+ on D7, then negative on D14, and D28<br><br>NP PCR negative (timing unclear)	GA: 30 weeks<br><br>Asymptomatic<br>Alive
Gao J, 2020<br><br>(Baby 3)	NP PCR+ at 32 weeks<br>IgG+, IgM+	Not reported	Not reported	Peripheral blood IgM+ and IgG+ on D1	Peripheral blood IgM+ on D7, then negative on D14 and D28	GA: 32 weeks<br><br>Asymptomatic<br>Alive

					NP PCR negative (timing unclear)	
Gao J (1), 2020	30-years-old, G2P1. Symptoms of Covid-19, NP PCR+ at 33 GW IgG+, IgM+	Caesarean section	Neonate separated from mother for first 14 days. Breastfed from D14	Cord IgM+ and IgG+ D1 Peripheral blood IgM+ and IgG+ on D1<br><br>Amniotic membranes PCR+, but cord blood PCR negative.<br><br>Peripheral blood and NP swab PCR negative on D1	Peripheral blood IgM+ on D3, D7, D14, and D28.<br><br>NP PCR negative on D1 and D3, PCR+ on D7, then negative on D14.<br><br>Anal swab PCR negative (timing unclear)	GA: 38 weeks BW: 2700g Apgar: 8, 9<br><br>Asymptomatic Abnormal CT Alive
Gonzalez R (1), 2021	34-years-old, multiparous with 3 previous term deliveries.<br>Symptoms of fever, cough and general malaise.<br>NP PCR+ at 25 weeks Abnormal CT	Vaginal delivery	Not reported	Cord blood IgM+ and Ig+ Peripheral blood IgM+ and IgG+ at 24 hr	Not done	GA: 37 weeks BW: 3180g Apgar:9, 9 Asymptomatic Alive
Gupta A, 2020*	31-years-old, G2P1, presented with 10 day history of fever, respiratory distress, cough and 20 hours of decreased fetal movements. NP PCR+ just after delivery. Abnormal radiology. Alive.	Caesarean section	Mother wore N95 during delivery. Neonate separated from mother after delivery. Neonate admitted to negative pressure room in NICU. Fed expressed breastmilk from D4.	NP PCR+ at 24h	NP PCR+ on D4, D6 and D25.<br><br>Endotracheal aspirate PCR+ D12 and D18<br><br>IgM+ on D12 and D18	GA: 29 weeks BW: 1330g Apgar: 3, 5, 7<br><br>Baby admitted to NICU at birth and intubated. Abnormal CXR. Grade III intraventricular

						haemorrhage. Improvement in symptoms after surfactant administered. Alive.
Haye MT, 2021	NP PCR+	Not reported	Not reported	NP PCR+ <24hr	NP PCR+ at 48hr	Not reported
Karade S, 2021	31-years-old, asymptomatic<br>NP PCR+ at 39 GW<br>Breast milk PCR neg	Vaginal delivery	Not reported	Amniotic fluid PCR+ (sterile collection)<br>Placental swab PCR+<br>NP PCR on D1	NP PCR neg on D5	GA: 39 weeks<br>Alive
Khushdil A, 2021	Infection during antenatal period, exact timing and further details not published.	Mode not reported.<br>Delivered in 3rd trimester	Not reported	NP PCR+ at 24h	NP PCR+ at 48h	BW: 'Appropriate for gestational age'<br>Admitted to NICU for observation.<br>Asymptomatic.<br>Normal CXR.<br>Alive.
Kulkarni R, 2020*	24-years-old, G1P0<br>Symptoms of fever and body ache<br>NP PCR negative, but IgG+, IgM+ before delivery.	Vaginal delivery	Mother wore surgical mask during delivery.<br>Neonate separated from mother until D11, not breastfed	NP PCR+ at 0h	NP PCR+ on D5 and D10, then negative on D14<br><br>IgM negative on D10, then positive on D21	GA: 38 weeks<br>BW: 3200g<br>APGAR: 6, 9<br><br>Symptomatic on D2<br>Normal CXR<br>Raised CRP, PCT and D-dimer.<br>Alive

Kumar V (2), 2021	32-years-old, G5P4L4.<br>Asymptomatic<br>NP PCR+ at 27 GW<br>Patchy bilateral opacities on CXR	Vaginal delivery	No direct contact with mother at birth.	NP PCR+ at 24hr	NP PCR+ at 48hr, D14, D22, D30, D42.<br>NP PCR neg on D50, D57	GA: 27 weeks<br>BW: 1185g<br>APGAR: 1, 5, 7<br>Symptomatic, impaired respiratory status, received PPV.<br>Transferred to NICU.<br>Extensive opacities on CXR.<br>Alive
Liu P, 2020	NP PCR negative, antenatal radiological changes	Caesarean section	Mother wore PPE during delivery. Neonate separated from mother and not breastfed	NP PCR+ on D1	NP PCR+ on D2, then negative on D3, D4, and D5	Asymptomatic<br>Normal radiology<br>Alive
Lopian M, 2020	G2 P2, presented at 37 weeks with fever, cough and malaise. NP PCR+ 2 days before delivery.	Vaginal delivery (vacuum-assisted)	Mother wore a face mask during delivery. Neonate isolated from mother several minutes after birth. Not breastfed.	NP PCR+ at 24h	NP PCR+ at 48h, then negative on D7, D14, D17	GA: 37 weeks<br>BW: 2770g<br>Apgar: 9, 10<br><br>Asymptomatic and alive.
Majachani N, 2020	31-years-old, G2P1, with past history of HIV and gestational diabetes. Presented with shortness of breath and myalgia. NP PCR+ 2 days before delivery. CXR abnormal.	Caesarean section due to oligohydramnios	Not reported	NP PCR+ at 24h	NP PCR+ at 48h & D7	GA: 34 weeks<br>Apgar: 9, 9<br><br>Admitted to NICU for monitoring. Given zidovudine for HIV

						prophylaxis.<br>Remained asymptomatic and alive.
Patanè L, 2020	Symptoms of cough and fever<br>NP PCR+ at 37 GW	Vaginal delivery	Mother wore mask during delivery.	Placenta IHC+ and ISH+<br>NP PCR+ at 0hr and 24hr	NP PCR+ on D7	GA: 37 weeks<br>BW: 2660g<br>APGAR: 9, 10<br>Asymptomatic<br>Alive
Schwartz DA (3), 2020*	NP PCR+<br>Severe symptoms of fever and severe respiratory distress, admitted to ICU<br>Delivered at 32 GW<br>Vaginal fluid PCR+	Caesarean section	Not reported	Amniotic fluid+<br>NP PCR- at 1hr	NP PCR+ on D4	GA: 32 weeks<br>BW: 2300g<br>APGAR: 5<br><br>Symptomatic on D1: respiratory distress<br>Required nasal (CPAP)<br>Abnormal CXR<br>Alive
Shaiba LA, 2021	30-years-old, primigravida.<br>NP PCR+ at 32 GW	Caesarean section	Separated from mother at birth.<br>Not breastfed	NP PCR+ at 10hr	4x NP PCR+ without timing, NP PCR neg on D29<br>Peripheral IgM and IgG neg	GA: 32 weeks<br>BW: 1700g<br>Symptomatic, Abnormal CXR
Song D, 2021	NP PCR+ and negative antibodies at delivery.<br>Asymptomatic.	Vaginal delivery	Separated from mother at birth and isolated for 14 days.	Cord blood PCR neg<br>NP PCR+ at 24hr	NP PCR+ on discharge.<br>Peripheral blood IgM and IgG neg on D2, D4 and D8;<br>IgM+ and IgG+ on D16	GA: 31 weeks<br>Asymptomatic

Tang F, 2020<br><br>(Baby 1)	Suspected case detected antenatally, near time of delivery.<br>NP PCR negative, IgM negative, IgG+ before delivery.	Caesarean section	Not reported	Peripheral blood IgM+ and IgG+ at birth<br>NP PCR- on D1<br>Cord PCR neg, amniotic fluid PCR neg	Peripheral blood IgM+ on D2	GA: 37 weeks<br>BW: 2600g<br>APGAR: 9 at 5 mins<br>Asymptomatic<br>Abnormal CXR<br>Alive
Tang F, 2020<br><br>(Baby 2)	Suspected case detected antenatally, near time of delivery.<br>NP PCR negative, peripheral blood IgM IgG+ before delivery.	Caesarean section	Not reported	Amniotic fluid PCR neg, cord blood PCR neg.<br>NP PCR neg on D1.<br>Peripheral blood IgM+ on D1	Peripheral blood IgM+ on D2, D5, D7	GA: 36 weeks<br>Asymptomatic, abnormal radiology.<br>Alive
Tang F, 2020<br><br>(Baby 3)	Suspected case detected antenatally, near time of delivery<br>NP PCR negative, IgM+, IgG+ before delivery	Caesarean section	Not reported	Peripheral blood IgM+ and IgG+ at birth<br><br>NP PCR negative on D1<br><br>Cord blood PCR neg, amniotic fluid PCR neg	Peripheral blood IgM+ on D2, D5	BW: 3090g<br>APGAR: 10 at 5 mins<br>Asymptomatic<br>Abnormal CXR<br>Alive
Vivanti A, 2020*	23-years-old, G1P0. Symptoms of fever and severe cough.<br>NP and blood PCR+ at 35 weeks<br>Vaginal fluid PCR+	Caesarean section	Delivery performed “in full isolation”	Amniotic fluid and placenta PCR+<br><br>Tracheal aspirate and neonatal blood PCR+ at 6hr<br><br>Anal swab PCR+ at 1hr	NP PCR+ on D3 and D18<br>Anal swab PCR+ on D3 and D18	GA: 35 weeks<br>BW: 2540g<br>APGAR: 4, 2<br><br>Required intubation, admitted to NICU<br>Alive
Zaigham M (1), 2021	27-years-old, G2P1, presented with three day history of fever, one day history of cough,	Caesarean section	Delivered in negative pressure room. No skin-to-skin contact.	Placenta IHC+	NP PCR+ at 48h, D5, then negative on D20	GA: 34 weeks<br>Apgar: 1, 4, 8

	abdominal pain and reduced fetal movements. NP PCR+ at delivery. Also blood PCR+, IgM weakly positive, IgG negative.		Neonate separated from mother for 60h. Then breastfed from D3 (breast milk PCR neg)		Blood IgM+ on D14 and D20. IgG negative on D7 but positive on D14 and D20	No initial signs of life, but spontaneous breathing at 6 mins.<br>Alive.
Zamaniyan M 2020	22-year-old, with controlled hypothyroidism, had severe symptoms of dyspnea, myalgia, anorexia, nausea and cough at 32 weeks. NP PCR+ 4 days before delivery and abnormal CT scan. Admitted to ICU. Mother developed ARDS postnatally and died D15PN	Caesarean section	Mother cared for in a negative pressure room. Neonate separated from mother and not breastfed.<br><br>Vaginal fluids PCR neg.	Amniotic fluid PCR+<br><br>But cord blood PCR negative and NP PCR negative at birth.	NP PCR+ at 24h and D7	GA: 32 weeks<br>BW: 2350g<br>Apgar: 8, 9<br><br>Neonate isolated in NICU and developed fever after birth, but improved.<br>Alive.
Zhang P, 2020<br><br>(Alamar I, 2020)	32-years-old G2P0, presented with vaginal bleeding due to placenta previa. Had 1 day history of fever, fatigue, dysgeusia and anosmia. NP PCR+ D1PN.	Caesarean section	Mother wore mask during hospitalisation. Neonate roomed-in with mother, 2m away in closed incubator. Directly breastfed from D2.	Placenta ISH+<br><br>NP PCR+ at 24h	NP PCR+ at 48h and D7	GA: 35 weeks<br>BW: 2630g<br>Apgar: 9, 9<br><br>Asymptomatic and alive.

NP=Nasopharyngeal Dx=Day x GA= Gestational age BW= Birth weight EM= Electron microscopy CPAP= continuous positive airway pressure NICU= neonatal intensive care unit IUFD= Intra-uterine fetal demise *classified as ‘possible’ in utero, despite not meeting WHO criteria of positive test 24-48 h but had further positive tests in first 7 days with no negative tests in between.

Appendix 8: Neonatal symptoms and radiological abnormalities of SARS-CoV-2 positive babies

	**SARS-CoV-2 positive term babies (≥37 weeks)**				**SARS-CoV-2 positive preterm babies and early pregnancy (<37 weeks)**				**SARS-CoV-2 positive babies (Gestational age not known)**				**All SARS-CoV-2 positive babies**			
	Mild maternal disease (n=152)	Severe maternal disease (n=10)	Severity not known (n=55)	Total (n=217)	Mild maternal disease (n=95)	Severe maternal disease (n=26)	Severity not known (n=22)	Total (n=143)	Mild maternal disease (n=95)	Severe maternal disease (n=0)	Severity not known (n=533)	Total (n=628)	Mild maternal disease (n=342)	Severe maternal Disease (n=36)	Severity not known (n=610)	Total (n=988)
**Neonatal symptoms**																
Yes	59	5	6	70	37	16	5	58	9	0	9	18	105	21	20	146
No	67	2	11	80	18	4	6	28	6	0	60	66	91	6	77	174
NK	26	3	38	67	40	6	11	57	80	0	464	544	146	9	513	668
**Radiological findings in newborn**																
Abnormal	27	2	11	40	15	8	5	28	2	0	1	3	44	10	17	71
Normal	26	1	0	27	9	4	1	14	0	0	9	9	35	5	10	50
NK	99	7	44	150	71	14	16	101	93	0	523	616	263	21	583	867

NK – Not known

Appendix 9. SARS-Cov-2 positivity in maternal and perinatal biological samples in cohort studies, women and their offspring

Samples assessed	No. of studies	No. of mothers with SARS-CoV-2 infection	No. of babies tested for SARS-CoV-2	No. samples tested	No. of samples positive
**Maternal samples**					
Vaginal fluid RT-PCR	9	1215	1050	315	2
Maternal stool RT-PCR	4	123	72	48	0
Breast milk RT-PCR	14	830	675	328	8
**Amniotic fluid, placenta, cord blood**					
Amniotic fluid RT-PCR	18	1882	1672	486	11
Placental swab any side RT-PCR	13	596	473	225	4
Placental tissue (any test) #	13	1223	1036	355	59
**Neonatal samples**					
Neonatal Stool/anal swab sample RT-PCR	6	560	552	44	2

Tests include IHC – Immunohistochemical evaluation; ISH - In situ hybridization; EM – Electron microscopy
