# Supplementary material for: SARS-CoV-2 positivity in offspring and timing of mother-to-child transmission: living systematic review and meta-analysis
Source: BMJ. 2022 Mar 16;376:e067696. doi: 10.1136/bmj-2021-067696 (PMC8924705; doi:10.1136/bmj-2021-067696)
Supplement: Supplementary file 1 [file allj067696.wf1.pdf]

Figure 1. Study selection process in the systematic review: SARS-CoV-2 positivity in babies born to mothers with coronavirus disease (COVID-19) and timing of mother-to-child transmission

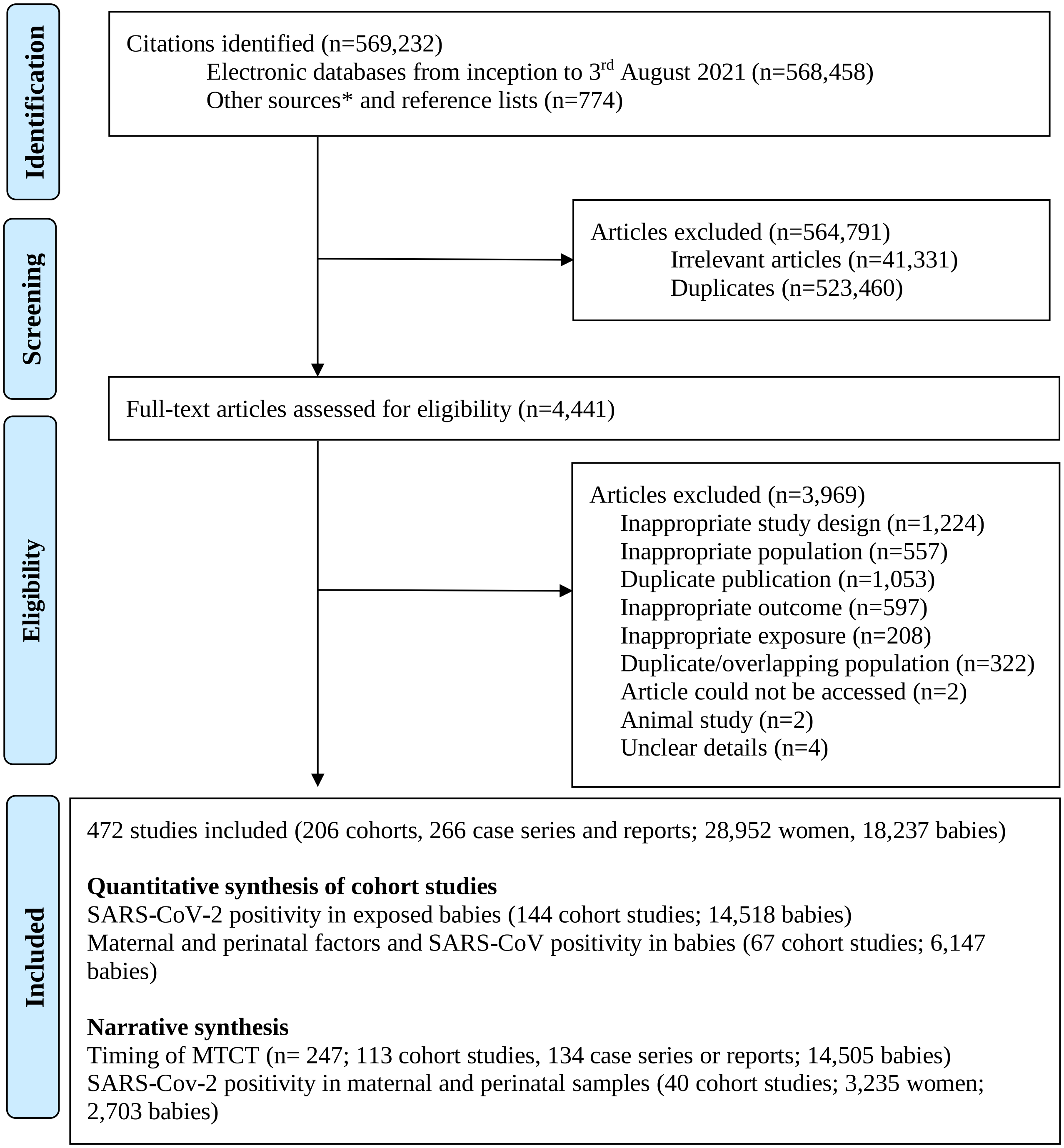

*Twitter, national reports, blog Thornton J, ObG Project, COVID-19 & Pregnancy Cases, https://www.obgproject.com/2020/04/07/covid-19-research-watch-with-dr-jim-thornton/ (Accessed May 12th 2020)
EPPI-Centre, COVID-19: a living systematic map of evidence, http://eppi.ioe.ac.uk/cms/Projects/DepartmentofHealthandSocialCare/Publishedreviews/COVID-19Livingsystematicmapoftheevidence/tabid/3765/Default.aspx (Accessed May 12th 2020)
Norwegian Institute of Public Health, NIPH systematic and living map on COVID-19 evidence, https://www.nornesk.no/forskningskart/NIPH_mainMap.html (Accessed May 19th 2020)
John Hopkins University Center for Humanitarian Health; COVID-19, Maternal and Child Health, Nutrition, http://hopkinshumanitarianhealth.org/empower/advocacy/covid-19/covid-19-children-and-nutrition/ (Accessed June 2nd 2020)
ResearchGate, COVID-19 research community, https://www.researchgate.net/community/COVID-19 (Accessed June 2nd 2020)
Living Overview of the Evidence, Coronavirus disease (COVID-19), https://app.iloveevidence.com/loves/5e6fdb9669c00e4ac072701d?population=5d062d5fc80dd41e58ba8459 (Accessed June 16th 2020)
